# A Mixed Flavonoid-Fish Oil Supplement Induces Immune-Enhancing and Anti-Inflammatory Transcriptomic Changes in Adult Obese and Overweight Women—A Randomized Controlled Trial

**DOI:** 10.3390/nu8050277

**Published:** 2016-05-11

**Authors:** Lynn Cialdella-Kam, David C. Nieman, Amy M. Knab, R. Andrew Shanely, Mary Pat Meaney, Fuxia Jin, Wei Sha, Sujoy Ghosh

**Affiliations:** 1 Department of Nutrition, School of Medicine—WG 48, Case Western Reserve University, 10900 Euclid Avenue, Cleveland, OH 44106, USA; lynn.kam@case.edu; 2 Human Performance Laboratory, North Carolina Research Campus, Appalachian State University, 600 Laureate Way, Kannapolis, NC 28081, USA; meaneyMP@appstate.edu; 3 Levine Center for Health and Wellness, Queens University of Charlotte, 1900 Selwyn Avenue, Charlotte, NC 28274, USA; knaba@queens.edu; 4 Department of Health & Exercise Science, Appalachian State University, ASU Box 32071, 111 Rivers Street, 050 Convocation Center, Boone, NC 28608, USA; shanelyra@appstate.edu; 5 North Carolina Research Campus, Dole Nutrition Research Laboratory, Kannapolis, NC 28081, USA; fuxia.jin@dole.com; 6 Bioinformatics Services Division, North Carolina Research Campus, UNC Charlotte, 600 Laureate Way, Kannapolis, NC 28081, USA; wsha@uncc.edu; 7 Program in Cardiovascular & Metabolic Diseases and Center for Computational Biology, Duke-NUS Medical School, 8 College Road, Singapore 169857, Singapore; sujoy.ghosh@duke-nus.edu.sg

**Keywords:** dietary supplement, flavonol, genomic profiling, omega-3, quercetin

## Abstract

Flavonoids and fish oils have anti-inflammatory and immune-modulating influences. The purpose of this study was to determine if a mixed flavonoid-fish oil supplement (Q-Mix; 1000 mg quercetin, 400 mg isoquercetin, 120 mg epigallocatechin (EGCG) from green tea extract, 400 mg *n*3-PUFAs (omega-3 polyunsaturated fatty acid) (220 mg eicosapentaenoic acid (EPA) and 180 mg docosahexaenoic acid (DHA)) from fish oil, 1000 mg vitamin C, 40 mg niacinamide, and 800 µg folic acid) would reduce complications associated with obesity; that is, reduce inflammatory and oxidative stress markers and alter genomic profiles in overweight women. Overweight and obese women (*n* = 48; age = 40–70 years) were assigned to Q-Mix or placebo groups using randomized double-blinded placebo-controlled procedures. Overnight fasted blood samples were collected at 0 and 10 weeks and analyzed for cytokines, C-reactive protein (CRP), F_2_-isoprostanes, and whole-blood-derived mRNA, which was assessed using Affymetrix HuGene-1_1 ST arrays. Statistical analysis included two-way ANOVA models for blood analytes and gene expression and pathway and network enrichment methods for gene expression. Plasma levels increased with Q-Mix supplementation by 388% for quercetin, 95% for EPA, 18% for DHA, and 20% for docosapentaenoic acid (DPA). Q-Mix did not alter plasma levels for CRP (*p* = 0.268), F2-isoprostanes (*p* = 0.273), and cytokines (*p* > 0.05). Gene set enrichment analysis revealed upregulation of pathways in Q-Mix *vs.* placebo related to interferon-induced antiviral mechanism (false discovery rate, FDR < 0.001). Overrepresentation analysis further disclosed an inhibition of phagocytosis-related inflammatory pathways in Q-Mix *vs.* placebo. Thus, a 10-week Q-Mix supplementation elicited a significant rise in plasma quercetin, EPA, DHA, and DPA, as well as stimulated an antiviral and inflammation whole-blood transcriptomic response in overweight women.

## 1. Introduction

Chronic inflammation and oxidative stress levels are elevated in middle-aged, overweight/obese adults and are characteristic of metabolic syndrome. Lifestyle interventions focused on weight loss via physical activity and/or dietary changes are often used to treat overweight and obese individuals. Many individuals, however, find it difficult to successfully implement these lifestyle changes. Weight regain is common after participation in a lifestyle intervention, with about one-half of individuals unable to sustain weight loss in the following year [1]. A nutritional supplement that reduces inflammatory and pro-oxidant side effects of obesity may be an attractive treatment option for those struggling with weight loss.

Flavonoids such as quercetin and epigallocatechin (EGCG) have been demonstrated *in vitro* and in animal models to have antioxidant and anti-inflammatory influences [2,3]. Quercetin belongs to the flavonoid subgroup flavonols, and can be found in fruits, vegetables, and other foods including onions, tea, apples, peppers, and berries [4]. EGCG is a catechin that is the most abundant flavonoid found in green tea [4]. While the results from *in vitro* and animal studies have been promising, human studies validating the important health benefits of these compounds are limited. In large community studies including both normal weight and overweight participants, large-dose quercetin supplementation at 500 mg/day or 1000 mg/day for 12 weeks was reported to have no influence on innate immune function, inflammation [5], body composition [6], or disease risk factors in adults [7]. Quercetin supplementation was, however, associated with a reduction in the severity and number of sick days associated with upper respiratory tract infections (URTI) in older, physically active adults [8]. Investigators report inconsistent results for the influence of EGCG supplementation on body composition and disease risk factors in human participants. The negative relationship between obesity and EGCG is supported by epidemiological research [9]. In randomized controlled studies in humans, small but significant decreases in body weight, waist circumference, and body fat with green tea supplementation was reported in three studies [10,11,12] while no effect on body composition was found in two [13,14]. Green tea supplementation has a varied effect on factors related to the metabolic syndrome, with decreases reported for serum amyloid alpha [13] and lipid peroxidation byproducts [12], but no changes in lipid profile [12,13,14], blood glucose [13,14], and insulin sensitivity [14].

A mixed flavonoid approach may be more effective in altering biomarkers related to the metabolic syndrome. In overweight men with mildly elevated C-reactive protein (CRP), Bakker *et al.* (2010) utilized nutrigenomics methods to measure reduced inflammation and oxidative stress after a 5-week flavonoid-rich supplement (resveratrol, green tea extract, α-tocopherol, vitamin C, omega-3 fatty acids, and tomato extract) intervention [15]. Omega-3 polyunsaturated fatty acids (*n*3-PUFA) such as EPA and DHA have also been shown to enhance both the bioavailibity and bioactive effects of quercetin [16,17,18,19]. A study with athletic participants utilized a mixed flavonoid-nutrient-fish oil supplement (Q-Mix; 1000 mg quercetin, 400 mg isoquercetin, 120 mg EGCG from green tea extract, 400 mg *n*3-PUFAs (220 mg EPA and 180 mg DHA) from fish oil, 1000 mg vitamin C, 40 mg niacinamide, and 800 µg folic acid) or placebo for 14 days prior to exercise, during 3 days of intensified cycling, and 7 days post-exercise [18,19]. With Q-Mix, inflammation and oxidative stress in these athletes were lower after 3 days of exercise compared to placebo [18,19]. This flavonoid-fish oil mixture has not yet been examined in overweight/obese middle-aged individuals. Therefore, the purpose of this study was to determine if a mixed flavonoid-nutrient-fish oil supplement containing vitamin C, niacin, and folic acid (Q-Mix) would reduce markers of inflammation and oxidative stress and alter genomic profiles in overweight middle-aged women.

## 2. Subjects and Methods

### 2.1. Subjects

Healthy, non-institutionalized, overweight/obese women (*n* = 48; 40–70 years, BMI ≥ 25 kg/m^2^) were recruited via mass advertising in the Charlotte, NC metropolitan area. Individuals were invited to participate if they were non-smokers and free of heart disease, cancer, stroke, arthritis, and diabetes. In addition, the participants agreed to avoid non-steroidal anti-inflammatory drugs (NSAIDs), dietary supplements that had the potential to influence inflammation or oxidative stress, and maintain normal diet and physical activity habits during the 10-week study. In total, 48 individuals were enrolled with all participants completing the study. This study was conducted according to the guidelines laid down in the Declaration of Helsinki and all procedures involving human subjects/patients were approved by the Institutional Review Board of Appalachian State University (IRB Study #10-0218). Written informed consent was obtained from all participants.

### 2.2. Research Design

During the first visit to the laboratory, height was measured with a stadiometer, and BMI and body composition were determined using a Tanita bioelectrical impedance scale (Tanita, Arlington Heights, IL, USA). Prior to the first blood draw and after 10-week supplementation, participants were asked to complete a three-day food record to evaluate energy and nutrient intake. Subjects were randomized to either Q-Mix (*n* = 24; 1000 mg quercetin, 400 mg isoquercetin, 120 mg EGCG from green tea extract, 400 mg *n*3-PUFAs (220 mg EPA and 180 mg DHA) from fish oil, 1000 mg vitamin C, 40 mg niacinamide, and 800 µg folic acid) or placebo (*n* = 24). Subjects were instructed to ingest two soft chew supplements twice daily (upon awakening, and between 6:00 and 7:00 p.m.) for 70 days. Participants maintained usual diet and physical activity habits, and adherence to the supplementation regimen and study requirements was monitored using daily logs.

### 2.3. Supplements

Supplements were soft, individually wrapped chews (5.3 g/chew), and each contained 250 mg quercetin, 100 mg isoquercetin, 30 mg EGCG from green tea extract, 100 mg *n*3 PUFAs (55 mg EPA and 45 mg DHA) from fish oil, 250 mg vitamin C (ascorbic acid and sodium ascorbate), 10 mg niacin, 200 μg folic acid, and 84 kJ (20 kcal) of citric acid and sugars in a carnauba wax, gelatin, soy lecithin, maize starch, glycerine and palm oil colored with Food, Drug and Cosmetic Act (FD & C) yellow No. 5 and blue No. 1). Vitamin C and niacin were included in the chews because data from Quercegen Pharmaceutical [20] indicated that these molecules enhanced the bioavailability of quercetin. Thus, the study examined whether the combination of the flavonoid mixture with vitamin C and niacin impacted outcome measures. Placebo supplements were prepared in the same way but did not include quercetin, isoquercetin, EGCG, EPA, DHA, vitamin C, niacin, or folic acid. Supplements were prepared by Nutravail Technologies (Chantilly, VA, USA) with Quercegen Pharmaceutical (Newton, MA, USA) and were administered utilizing double-blinded procedures.

### 2.4. Blood Collection

Overnight fasted blood was collected from the antecubital vein of the subject prior to supplementation and after 10-week supplementation. For RNA analysis, whole blood (2.5 mL) was directly transferred to PAXgene blood RNA tubes (Qiagen, Santa Clara, CA, USA). Other blood samples were collected in serum separator, lithium heparin or ethylenediaminetetraacetic acid (EDTA) tubes as appropriate. Samples were sent to the Carolina Medical Center (Charlotte, NC, USA) for comprehensive metabolic panel and lipid panel. For the remaining assays, the plasma portion was collected, aliquoted into 1.5 mL microcentrifuge tubes, snap frozen in liquid nitrogen, and stored at −80 °C until further analysis.

### 2.5. Plasma Quercetin Analysis

Total plasma quercetin (quercetin and its primary conjugates) was measured by reversed-phase high-performance liquid chromatography with UV detection following solid-phase extraction as described previously [21]. Briefly, heparinized plasma aliquots (500 μL) were incubated for 2 h at 37 °C with 10 μL 10% dl-dithiothreitol solution, 50 μL of 0.58 mol/L acetic acid, 50 μL of a mixture of β-glucuronidase/arylsulfatase, and crude extract from *Helix pomatia* (Roche Diagnostics Corporation, Indianapolis, IN, USA). Chromatographic analysis was performed using the Ultimate 3000 HPLC-PDA system (Dionex Corporation, Sunnyvale, CA, USA) with a Gemini C18 column (Phenomenex, Torrance, CA, USA).

### 2.6. Plasma F_2_-Isoprostanes

Plasma F_2_-isoprostanes were assayed using a negative ion chemical ionization gas-chromatography mass spectrometry (GC-MS) and an Agilent 6890N gas chromatography interfaced to an Agilent 5975B inert MSD mass spectrometer (Agilent Technologies, Inc., Santa Clara, CA, USA) as described previously [22]. Briefly, after free F_2_-isoprostanes were extracted from heparinized plasma, an internal standard deuterated (2H4) prostoglandin F_2α_ was added. Next, the mixture was filtered through C18 Sep Pak column, and silica solid-phase extraction was performed. The F_2_-isoprostanes, after conversion to pentafluorobenzyl esters, were then subjected to thin-layer chromatography, converted to trimethylsilyl ether derivatives, and analyzed using GC-MS.

### 2.7. RNA Isolation and Microarray Hybridization

Fasting whole blood (2.5 mL) was drawn from study subjects by standard venipuncture and directly transferred to PAXgene blood RNA tubes (Qiagen, Santa Clara, CA, USA). Isolation of total RNA was accomplished according to the manufacturer’s instructions. PAXgene tubes were processed following the PAXgene protocol. Extracted RNA from all samples was stored at −70 °C until being processed for microarray hybridizations. RNA quality was assessed by spectrophotometry (absorbances at 230, 260 and 280 nm) and gel electrophoresis. Out of a total of 32 possible samples, 29 samples had sufficient total RNA concentration (>85 ng/uL) for further processing (260/280 ratio for all samples ranged from 1.97 to 2.12). Hybridization of 100 nanograms of labeled cRNA samples were carried out on Affymetrix Human HuGene-1_1-ST array plates according to the manufacturer’s instructions. Microarray data was deposited in the Gene Expression Omnibus data repository (accession number GSE74560).

### 2.8. Microarray Data Analysis

Background adjusted, quantile normalized gene expression signals were generated by the robust multi-array average method using RMAExpress [23]. Out of a total of 28,869 original probes tested, 22,148 probes could be annotated to protein-coding genes. Probes with a normalized average expression level of less than 50 units (5.64 in the log2 scale) in all of the four tested groups were eliminated from further analysis, resulting in 14,325 remaining probes. The magnitude and statistical significance of differentially regulated genes was ascertained by two-way repeated measures ANOVA [24] using SAS 9.3 (SAS Institute, Cary, NC, USA). The magnitude of differential expression was estimated by first calculating the difference in gene expression levels between baseline and end of supplementation in each of the two groups and then calculating the net change in expression between the two groups over the time course of supplementation. Net difference values were normalized to the magnitude of gene expression by conversion to percent change with respect to the gene’s expression level in the pre-treatment placebo group. Additionally, differential gene expression in each of the major white blood cell subtypes (lymphocytes, monocytes, neutrophils, eosinophils, basophils) was analyzed via a linear regression-based signal deconvolution algorithm in the cell-type-specific Significance Analysis of Microarrays (csSAM) software [25].

### 2.9. Pathway and Network Enrichment Analysis

Pathway enrichment analysis was conducted via the Gene Set Enrichment Analysis software package [26,27] using the “pre-ranked” option. For this analysis, genes with >10% net difference in expression in the pre-supplementation Q-Mix and placebo samples (representing potentially noisy genes) were first removed. The remaining genes were then ranked based on their net change in expression between Q-Mix and placebo groups. The ranked list was subsequently used to query the Reactome pathway database [28] for statistically significant enrichment of higher or lower ranked genes in one or more of the pathways. Significance estimates were adjusted for multiple testing via the false discovery rate (FDR).

We also carried out an overrepresentation analysis of canonical pathways and biofunctions via the Ingenuity Pathway Analysis (IPA) tool (Qiagen, Valencia, CA, USA). Genes showing an absolute percent change in expression of ≥25% between Q-mix and placebo were used as input. Statistical significance of overrepresentation was assessed by the right-tailed Fisher’s exact test. The likelihood for activation/inhibition of a pathway or biofunction, based on the expression patterns of its constituent genes and their known activating/inhibitory effects on that pathway or function (derived from literature), was statistically quantified by the *z*-score. Biofunctions with a *z*-score ≥2.0 or ≤−2.0 were considered to be “activated” or “inhibited”, respectively.

### 2.10. Statistics

Data was summarized using means and standard deviations. Two-way repeated-measures ANOVA (time × treatment) was used to detect significant differences for a participant’s characteristics, blood analysis, and gene expression. Multiple testing was corrected using the Benjamini-Hochberg method for *false discovery rate* (FDR). Significance was set at FDR-adjusted *p*-value < 0.05. Analysis was conducted using SAS 9.3 (SAS Institute, Cary, NC, USA).

## 3. Results

### 3.1. Participants’ Characteristics, Dietary Intake, and Blood Analytes

**Figure 1:**
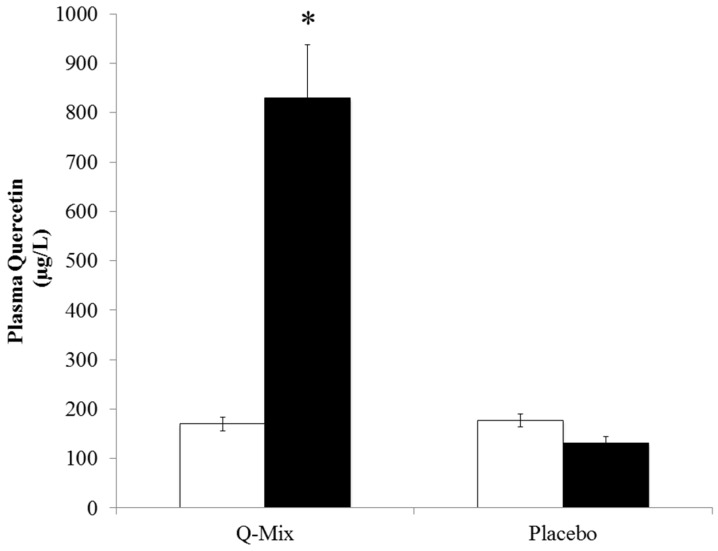
Plasma quercetin levels by supplement groups at pre- (□) and post- (■) supplementation. Participants were randomly assigned to either placebo (0-mg/day of isoquercetin, EGCG, and *n*3-PUFA) or Q-Mix (400 mg isoquercetin, 120 mg ECGC, and 400 mg *n*3-PUFAs containing vitamin C and niacin). Error bars represent SE. Significance test was based on two-way ANOVA (group and time). * Plasma quercetin levels increased with Q-mix (*p* < 0.001) from pre- to post-supplementation compared to placebo.

**Figure 2:**
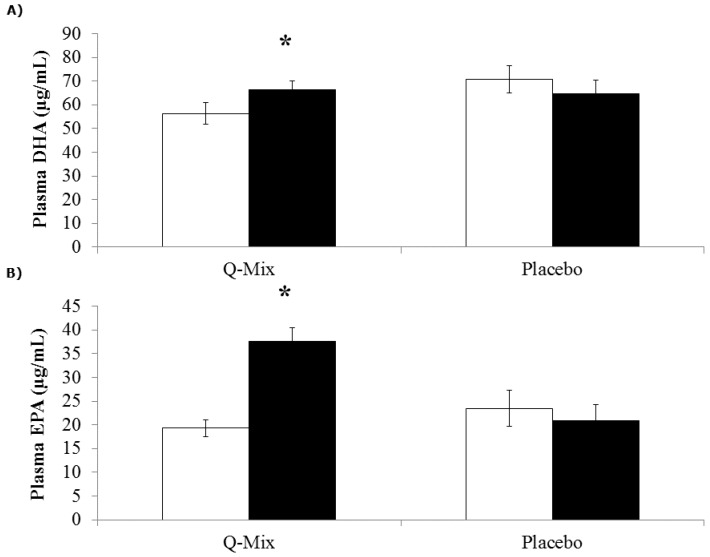
Plasma levels of *n*3-PUFAs in participants who were randomly assigned to either placebo (0 mg/day of isoquercetin, EGCG, and *n*3-PUFA) or Q-Mix (400 mg isoquercetin, 120 mg ECGC, and 400 mg *n*3-PUFAs containing vitamin C and niacin). (A) * Plasma DHA levels increased with Q-Mix (*p* < 0.001) from pre- (□) to post- (■) supplementation compared to placebo. Error bars represent SE. Significance test was based on two-way ANOVA (group and time); (B) * Plasma levels of EPA also increased with Q-Mix (*p* < 0.001) from pre- (□) to post- (■) supplementation compared to placebo. Error bars represent SE. Significance test was based on two-way ANOVA (group and time).

Participants in both Q-Mix and placebo groups were similar in age and body composition (Table 1). Plasma quercetin levels were six-fold higher at 10 weeks in the Q-Mix compared to placebo group (*p* < 0.001; Figure 1). Similarly, DHA and EPA were elevated by 4% and 80% at 10 weeks in the supplement compared to the placebo group (*p* < 0.001; Figure 2). Two-thirds of participants had CRP levels greater than 2 mg/L with four individuals (two in each group) having CRP levels greater than 10 mg/L (Table 2). The pattern of change between Q-Mix and placebo groups over the 10-week study period did not differ for inflammatory biomarkers, oxidative stress (Table 2), components of the lipid profile, and selected blood chemistries (Table 3). We evaluated CRP levels excluding the four participants with CRP >10 mg/L since this is associated with acute inflammation [29]. Changes in CRP levels were similar in both groups with these participants excluded (Q-mix = 1.55 ± 2.97 mg/L and placebo = 1.58 ± 2.06 mg/L, *p* = 0.9626). Energy and macronutrient intakes were similar between groups and did not change with the intervention (Table 3).

### 3.2. Single Gene-Level Analysis

In Q-Mix compared to placebo, 48 genes were found to be significantly differentially expressed (FDR *p*-value < 0.05) with 18 upregulated and 30 downregulated genes. These genes spanned a wide variety of functions with no obvious association with any particular biological process or molecular functions. A subset of genes with greater treatment-specific variation (≥10% net difference between Q-Mix and placebo) compared to random variation (<10% difference between pre-treatment samples in Q-Mix or placebo) are provided in Supplementary Materials Table S1.

Since gene expression values in whole blood can be influenced by the relative proportion of individual blood cell types, the proportions of the major white-blood cell types (lymphocytes, monocytes, neutrophils, eosinophils and basophils) in the post-supplementation Q-Mix and placebo samples were compared. The blood cellular distribution in the placebo samples was assumed to stay constant during the 10-week study. No statistically significant differences were identified for the cell-type frequencies between Q-Mix and placebo (Supplementary Materials Table S2). Additionally, cell-type specific gene expression analysis using csSAM software did not detect any cell-type-specific differentially expressed genes between Q-Mix and placebo.

### 3.3. Pathway Enrichment Analysis

**Figure 3:**
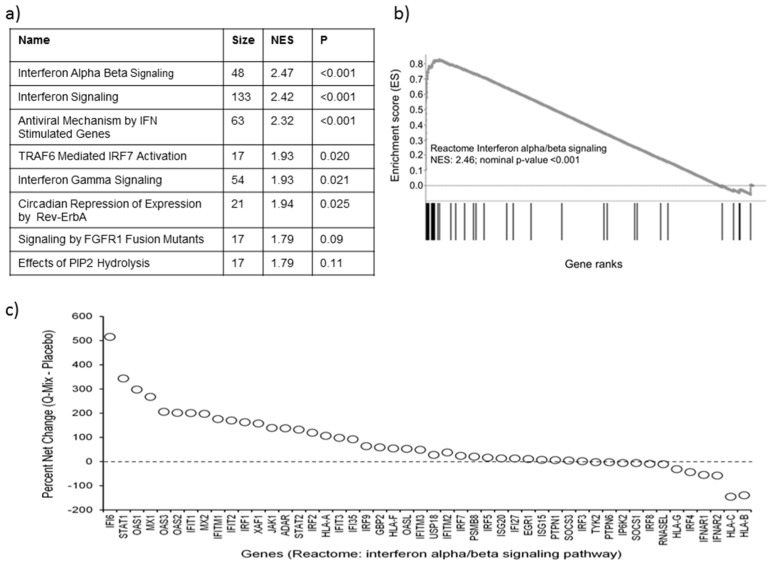
Differential regulation of interferon-signaling gene expression in Q-mix *vs.* placebo (Pbo). (**a**) Top-scoring Reactome pathways identified as upregulated in Q-mix relative to placebo by gene-set (pathway) enrichment analysis: Column 1, pathway names; column 2, number of genes in pathway; column 3, NES, a measure of pathway enrichment normalized for the number of genes in the pathway; column 4, false discovery rate (FDR) *p*-value. Pathways with FDR 2 ≤10% are shown; (**b**) Enrichment plot for the top-ranked Reactome pathway (interferon alpha/beta signalling), upregulated in Q-Mix compared to placebo. The graph represents the incremental change in the enrichment scores for this pathway during gene-set enrichment analysis (maximized at 0.82). Relative positions of genes belonging to this pathway are indicated by the bars under the graph. Lines clustered to the left represent higher ranked genes in the pre-ranked gene list; (**c**) Differential expression profiles for genes belonging to the interferon alpha beta signaling pathway, rank-ordered by their percent net expression change between Q-Mix and placebo.

**Figure 4:**
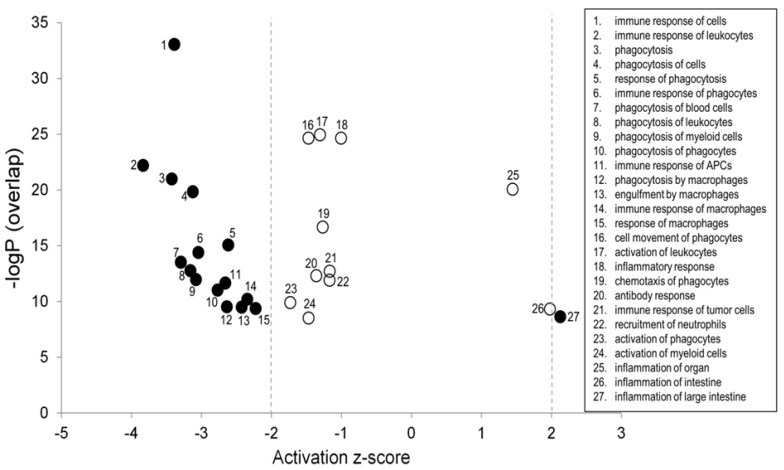
Overrepresentation analysis of inflammatory response-related bio-functions, using Ingenuity Pathway Analysis. A total of 27 sub-functions, under the broader category of inflammatory response, were identified from the data. Results are based on gene expression changes in Q-mix *vs.* placebo. The extent of pathway overrepresentation is shown via the negative logarithm of the overlap *p*-value (*y*-axis), and consistency of gene expression changes are shown via the *z*-score (*x*-axis). The dashed lines mark *z*-scores ≤−2.0 or ≥2.0, indicative of inhibited and activated sub-functions, respectively (filled circles). Sub-functions are numbered and described in the accompanying legend.

An exploratory pathway enrichment analysis was conducted on genes that were ranked by their percent net differences in expression between Q-Mix and placebo samples. The gene-set enrichment analysis algorithm (GSEA) was utilized in the “pre-ranked” mode and was used to query the Reactome pathway database for identification of enriched pathways. The top Q-Mix upregulated pathways (sorted by nominal *p*-values) are shown in Figure 3a, whereas a more detailed list of pathways (up- and downregulated in Q-Mix, nominal *p* ≤ 0.05) are presented in Supplementary Materials Table S3. The majority of the Q-Mix upregulated pathways were related to interferon signaling. We then investigated the individual expression patterns for the genes contained in the top-ranked interferon alpha beta signaling pathway (Figure 3b). The enrichment plot demonstrates that the enrichment scores rise sharply on the left and then decline, suggesting that several genes in the specified pathway occupy closely positioned ranks due to their high percent change in expression between Q-Mix and placebo. We then considered the individual expression differences for all 48 genes belonging to this pathway (Figure 3c). A consistently positive percent net change in the Q-Mix group was observed for 36/48 genes, suggesting that the observed significance of the pathway was derived from consistent changes in a majority of its constituent genes, some of the changes being large (e.g., IFI6, STAT1, OAS1, MX1, *etc.*). Overrepresentation analysis of genes undergoing at least a 25% change in expression between the Q-Mix and placebo groups via the IPA tool also identified significant differences in interferon signaling, among others (Supplementary Materials Table S4). Additionally, IPA also identified a subset of inflammatory response-related pathways, pertaining to phagocytosis, that were significantly downregulated in Q-Mix samples. The subset of inflammatory response-related biofunctions (sub-functions) with the extent of their overrepresentation in the experimental dataset (quantified by the overlap *p*-value, *y*-axis), and the likelihood of their activation or inhibition (quantified by the *z*-score, *x*-axis) are presented in Figure 4 and Supplementary Materials Figure S1. Fifteen sub-functions, most of them closely related to phagocytosis, demonstrated highly significant overlap with strong evidence for inhibition in the Q-Mix samples, compared to placebo (in contrast, 1 sub-function predicted as significantly activated). The majority of the other inflammatory response-related sub-functions were also highly overrepresented in the dataset, but not predicted to be significantly activated or inhibited (absolute *z*-score < 2.0).

## 4. Discussion

Ten weeks of supplementation with Q-Mix was associated with gene expression alterations consistent with augmented viral defense and reduced immune cell trafficking and phagocytosis, a component of the inflammatory response [30]. Change in traditional biomarkers for inflammation and oxidative stress did not differ between Q-Mix and placebo groups during the 10-week study. Notably, key genes in type 1 IFN-induced pathway were upregulated with Q-Mix *vs.* placebo including signal transducer and activator of transcription 2 (STAT2), IFN-regulatory factor 9 (IRF9), and MX1, thereby indicating enhanced anti-viral response [30]. A reduction in transcript levels of the host chaperone for viral replication, HSP90B1 [31], in Q-Mix relative to placebo was also observed, providing further support of the potential anti-viral properties of Q-Mix [30]. In addition, reductions in gene expression related to phagocytosis, a key process linking innate immunity to inflammation, may be indicative of an attenuation in general inflammatory load in Q-Mix-treated subjects [32]. Thus, Q-Mix compared to placebo supplementation in overweight women for 10 weeks resulted in gene expression indicative of enhanced antiviral defense and decreased leukocyte trafficking, but without change in typical biomarkers of reduced inflammation, oxidative stress, and blood lipids and lipoproteins.

The Q-mix supplement contained multiple flavonoids including quercetin, isoquercetin, EGCG, and other green tea catechins. Quercetin and EGCG have independent effects on improved viral response [33]. In cell culture, quercetin has been found to inhibit viral replication of hepatitis B virus and several respiratory viruses [33], and to stimulate apoptosis of the Epstein-Barr virus [34]. Quercetin supplementation has also been associated with reduction in the duration and incidence of URTI [8,21]. Specifically, Heinz *et al.* (2010) reported that a 12-week quercetin supplementation (1000 mg/day) was associated with reduction in severity of URTI and the number of total sick days from URTI in physically active middle-aged and older adults compared to the placebo [8]. In cyclists, a three-week supplementation of quercetin (1000 mg/day) *vs.* placebo was linked to a reduction in URTI incidence following three days of intensified training [21]. Although quercetin exerts strong anti-pathogenic effects against several causal agents of URTI in culture studies, quercetin’s *in vivo* role after metabolic transformation in augmenting immune function appears limited, as we have reported previously [5,18]. Flavonoid supplementation has recently been shown by our research group to increase factors in serum that interfere with the ability of viruses to effectively infect and replicate within cells [32]. The mechanism by which quercetin, EGCG, and other flavonoids provide protection from virus infections remains to be determined, but flavonoids may interfere with viral penetration into cells by damaging the virus particle [35,36]. Our study provides a molecular level understanding of the mechanisms underlying Q-Mix function, namely a transcriptomic “priming” of the antiviral response repertoire compared to levels in the placebo.

A potential suppression of immune cell trafficking and inflammatory pathways in Q-Mix *vs.* placebo is suggested by gene expression patterns. Whether this observed change is a direct effect of quercetin or a manifestation of the known anti-inflammatory properties of type 1 interferons, e.g., via inhibition of the inflammasome, is not clear [37]. Leukocyte trafficking in particular has been implicated as a potential therapeutic target for treating inflammatory disease [38]. In the present study, however, no alterations in cytokine levels were detected between groups. Our findings are consistent with previous studies in which a 12-week quercetin supplementation (1000 mg/day or 500 mg/day *vs.* placebo) in community-dwelling individuals had little impact on disease-related risk factors, including inflammation [7]. Bakker *et al.* (2010) reported that five-week supplementation with a polyphenol blend did not alter traditional biomarkers in overweight men compared to a placebo but favorably altered nutrigenomic profile factors consistent with anti-inflammatory processes [15]. In a prior study, we reported that 12-week supplementation with quercetin, vitamin C, and niacin (1000, 240, and 10 mg/day of quercetin, vitamin C, and niacin, respectively) exerted disparate and wide-ranging metabolic shifts, but current knowledge was insufficient to elucidate potential health effects [39]. The top metabolic shifts detected were serum guaiacol, 2-oxo-4-methyolthiobutanoic acid, allocystathionine, and two bile acids with no changes in inflammatory and oxidative stress metabolites found [39]. In a randomized controlled trial in a trained cyclist, our research group compared the effectiveness of Q-mix compared to quercetin alone and placebo on reducing post-exercise inflammation and oxidative stress [18]. Q-mix was associated with a 50% reduction in CRP levels after exercise (14 h post and 3 days post) and a 39% reduction in IL-6 immediately after exercise when compared to placebo [18]. No changes in CRP and IL-6 levels were observed with quercetin alone compared to placebo [18]. Thus Q-Mix may be more effective in countering acute compared to systemic inflammation. It should be noted that the Q-mix supplement also contained *n*3 PUFAs. Fish oil supplementation at doses of ~2–3 g/day have been associated with favorable gene changes involved in inflammatory [40,41], oxidative stress [42], and immune pathways [43]. In the present study, a lower dose (400 mg/day) of *n*3 PUFA was included in the supplement. Bowens *et al.* (2009) compared 26-week EPA + DHA supplementation at high dose (1.8 g/day) *vs.* low dose (400 mg/day) in older adults (≥65 years of age). The low dose resulted in similar but attenuated downregulation of five out of six genes examined compared to the high dose [41]. In randomized clinical trials, the effect of fish oil supplementation on inflammation had been examined and has not been consistently associated with an improvement in CRP and other inflammatory biomarkers [43,44].

A potential limitation in the current study includes the relatively short supplementation period of 10 weeks. Long duration of flavonoid supplementation may be needed before biological benefits are detected by traditional biomarkers due to metabolic transformation [37,38,39,40]. Studies support that high, habitual dietary polyphenol intake can be measured through elevated urinary phenolic concentration and is associated with reduced mortality rates [41,45]. Polyphenols are not absorbed in their pure forms but undergo metabolic transformation which diminishes their biological impact [45]. The majority of ingested polyphenols are not absorbed in the small intestine, and undergo bacterial degradation in the colon. The gut-derived phenolics can be reabsorbed and exert subtle bioactive effects that may become measurable over time [46,47,48]. Another potential limitation is that whereas the study was sufficiently powered to detect differences in improvements between groups of 1.5 mg/L for CRP and 0.18 pg/mL in IL-6, it was underpowered to detect smaller but potentially clinically significant changes in these biomarkers.

## 5. Conclusions

A 10-week supplementation with Q-Mix for overweight/obese women was associated with gene changes suggestive of improved anti-viral responses and lessening of immune cell trafficking. These gene expression changes with Q-mix supplementation occurred without alteration in traditional measures of inflammation, oxidative stress, blood lipids, or body weight. Future research should examine the impact of longer term flavonoid supplementation, reduced doses, and the potential synergistic effect of exercise on health outcomes.

## Figures and Tables

**Table 1:** Participants’ characteristics by supplement groups *.

	Q-Mix (*n* = 24)		Placebo (*n* = 24)		*p* ^†^
	Mean	SD	Mean	SD	
Age (year)	55.3	7.3	56.9	9.3	0.4919
Height (m)	1.64	0.06	1.63	0.06	0.5007
Body mass (kg)					
Pre-study	89.5	14.1	82.5	14.5	0.5349
Post-study	90.7	14.2	83.3	14.6	
BMI (kg/m^2^)	33.3	5.9	31.0	5.4	0.1692
Body fat (%)	37.0	4.7	35.3	5.7	0.2974

* Participants were randomly assigned to either: placebo (0 mg/day of isoquercetin, EGCG, and *n*3-PUFA) or Q-Mix (400 mg isoquercetin, 120 mg ECGC, and 400 mg *n*3-PUFAs containing Vitamin C and Niacin); ^†^ *p*-values are two-sided. Unpaired *t*-tests were used to assess significant differences between groups.

**Table 2:** Pre- and post-study inflammatory and oxidative stress blood markers by supplement group *.

Inflammatory Markers	Time	Q-Mix (*n* = 24)		Placebo (*n* = 24)		*p* ^†^
		Mean	SD	Mean	SD	
GM-CSF (pg/mL)	Pre	0.73	0.58	0.94	1.32	0.3367
	Post	0.78	0.56	0.90	1.14	
IFN-γ (pg/mL)	Pre	1.16	0.82	1.13	0.64	0.2288
	Post	1.51	1.09	1.22	0.54	
IL-1β (pg/mL)	Pre	0.16	0.17	0.20	0.24	0.5825
	Post	0.18	0.19	0.25	0.25	
IL-2 (pg/mL)	Pre	0.72	0.68	0.61	0.48	0.8645
	Post	0.69	0.69	0.60	0.44	
IL-6 (pg/mL)	Pre	1.47	0.97	1.63	1.69	0.1527
	Post	1.35	0.74	1.78	1.72	
IL-8 (pg/mL)	Pre	3.58	2.54	3.70	2.35	0.4471
	Post	3.63	2.00	4.09	2.44	
IL-10 (pg/mL)	Pre	4.06	9.77	2.20	2.86	0.6386
	Post	4.48	12.02	2.36	2.53	
IL-12p70 (pg/mL)	Pre	1.78	1.95	1.72	2.17	0.6003
	Post	1.81	2.20	1.95	2.02	
TNF-α (pg/mL)	Pre	5.52	1.83	5.63	2.40	0.5146
	Post	5.32	1.58	5.64	2.23	
CRP (mg/L)	Pre	4.64	5.14	4.69	7.29	0.268
	Post	5.60	4.27	6.81	8.64	
Oxidative Stress Marker						
F_2_-isoprostanes (pg/mL)	Pre	98.6	28.2	117.4	34.3	0.2727
	Post	96.4	26.0	106.1	23.0	

* Participants were randomly assigned to either: placebo (0 mg/day of isoquercetin, EGCG, and *n*3-PUFA) or Q-Mix (400 mg isoquercetin, 120 mg ECGC, and 400 mg *n*3-PUFAs containing vitamin C and niacin); ^†^ Significance difference was determined using two-way ANOVA. Blood marker concentration was the response variable, and supplement groups (placebo and Q-mix) and time (pre and post) were the predictor variables.

**Table 3:** Pre- and post-study dietary intake, lipid profile, and selected values from comprehensive metabolic panel by supplement group *.

Dietary Intake	Time	Q-Mix (*n* = 24)		Placebo (*n* = 24)		*p* ^†^
		Mean	SD	Mean	SD	
Energy (kJ/day)	Pre	8363	2161	8016	1727	0.3626
	Post	8081	2112	7223	2348	
Carbohydrates (% total energy)	Pre	50	9.0	54	7.7	0.2988
	Post	51	8.6	52	8.0	
Protein (% total energy)	Pre	15	3.6	16	2.8	0.0990
	Post	14	3.4	17	3.3	
Fat (% total energy)	Pre	35	7.8	32	7.8	0.5048
	Post	34	7.4	32	6.8	
Fiber (g/day)	Pre	18	7.7	20	11.1	0.2078
	Post	19	9.2	18	8.5	
Lipid Panel						
Cholesterol (mg/dL)	Pre	201	32.3	210	47.2	0.4144
	Post	199	35.5	213	51.6	
LDL-Cholesterol (mg/dL)	Pre	127	29.2	131	44.4	0.4218
	Post	125	31.1	133	51.3	
HDL-Cholesterol (mg/dL)	Pre	51.1	11.9	54.7	11.4	0.3122
	Post	52.9	13.1	58.1	11.8	
Triglycerides (mg/dL)	Pre	116	34.7	124	51.2	0.6909
	Post	109	40.5	112	37.5	
Comprehensive Metabolic Panel						
Alkaline phosphatase (IU/L)	Pre	72.6	14.2	74.5	13.9	0.1086
	Post	73.2	14.0	78.5	14.6	
Aspartate aminotransferase (AST; IU/L)	Pre	21.5	5.9	25.3	9.5	0.3035
	Post	22.2	7.2	24.1	9.6	
Blood Urea Nitrogen (BUN; mg/dL)	Pre	10.3	2.7	12.2	4.1	0.7283
	Post	10.7	2.7	12.3	3.8	
Creatinine (mg/dL)	Pre	0.816	0.14	0.836	0.24	0.6786
	Post	0.812	0.13	0.846	0.24	
Glucose (mg/dL)	Pre	93.8	9.7	97.0	10.2	0.6234
	Post	95.2	11.3	99.7	14.3	
Sodium (mEq/L)	Pre	139	1.8	139	2.6	0.7400
	Post	140	1.9	140	2.5	
Total Bilirubin (mg/dL)	Pre	0.688	0.18	0.667	0.18	0.6036
	Post	0.679	0.20	0.692	0.21	

* Participants were randomly assigned to either: placebo (0 mg/day of isoquercetin, EGCG, and *n*3-PUFA) or Q-Mix (400 mg isoquercetin, 120 mg ECGC, and 400 mg *n*3-PUFAs containing vitamin C and niacin); ^†^ Significance difference was determined using two-way ANOVA. Blood marker concentration was the response variable, and supplement groups (placebo and Q-mix) and time (pre and post) were the predictor variables.

## Supplementary Materials

The following are available online at http://www.mdpi.com/2072-6643/8/5/277/s1.
