# Supplementary material for: A Mixed Flavonoid-Fish Oil Supplement Induces Immune-Enhancing and Anti-Inflammatory Transcriptomic Changes in Adult Obese and Overweight Women—A Randomized Controlled Trial
Source: Nutrients. 2016 May 11;8(5):277. doi: 10.3390/nu8050277 (PMC4882690; doi:10.3390/nu8050277)
Supplement: Supplementary file 1 [file nutrients-08-00277-s001.docx]

Supplementary Materials: A Mixed Flavonoid-Fish Oil Supplement Induces Immune-Enhancing and Anti-Inflammatory Transcriptomic Changes in Adult Obese and Overweight Women—A Randomized
Controlled Trial

Lynn Cialdella-Kam, David C. Nieman, Amy M. Knab, R. Andrew Shanely,
Mary Pat Meaney, Fuxia Jin, Wei Sha and Sujoy Ghosh


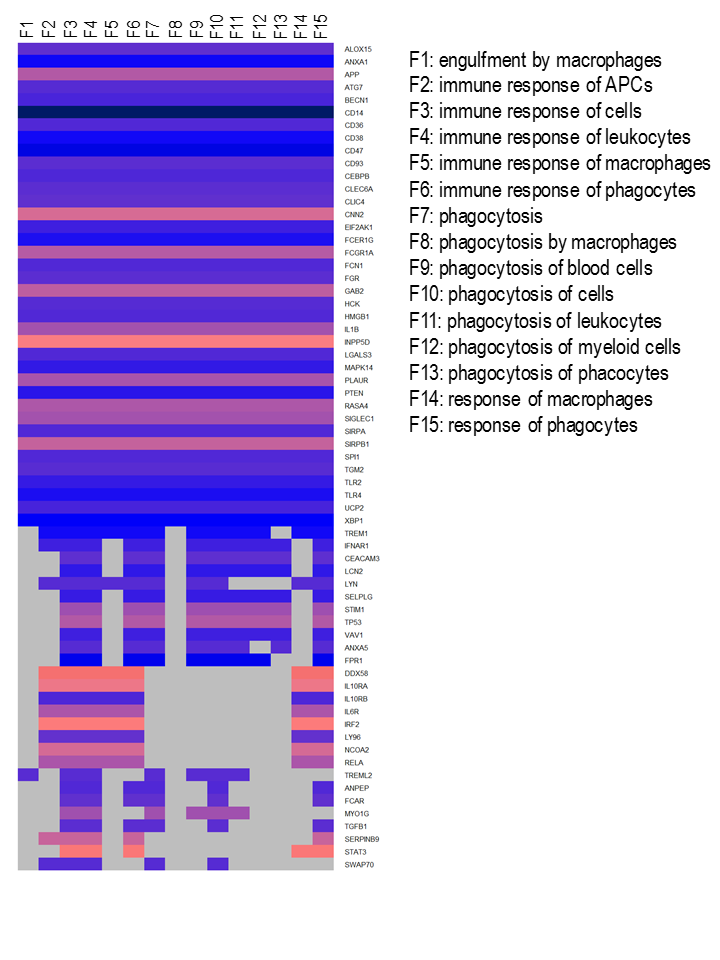


**Figure S1.** Over-representation analysis of inflammatory response-related bio-functions, using Ingenuity Pathway Analysis. Heatmap of gene expression in the significantly downregulated sub-functions (z < −2.0). Genes present across 5 or more sub-functions are shown and color-coded by their percent change (blue and red shades correspond to negative and positive percent changes in Q-Mix *vs.* placebo, respectively).

**Table S1.** 2-way repeated measures ANOVA analysis of whole blood gene expression in the Q-Mix and placebo groups at baseline and after 10-weeks
of supplementation.

| **Probe  Sets** | **Gene  Symbol** | **Gene Name** | **Placebo:  Pre to Post Change** | **Q-Mix: Pre  to Post  Change** | **Net Change:  Q-Mix *vs.*  Placebo** | **% Net Change:  Average  Placebo Pre** | **Q-mix as % of Placebo  Pre** | ***p*-Value** | **BH_FDR** | ***p* < FDR** |
| --- | --- | --- | --- | --- | --- | --- | --- | --- | --- | --- |
| 8018522 | FBF1 | Fas (TNFRSF6) binding factor 1 | −14.530 | 3.095 | 17.625 | 17.820 | 8.467 | 0.002 | 0.025 | * |
| 7997230 | PSMD7 | proteasome 26S subunit, non-ATPase 7 | 29.582 | −15.988 | −45.570 | −11.238 | −3.652 | 0.002 | 0.024 | * |
| 8042207 | COMMD1 | copper metabolism domain containing 1 | 18.999 | −16.344 | −35.344 | −15.968 | −4.273 | 0.003 | 0.025 | * |
| 8062695 | SRSF6 | serine/arginine-rich splicing factor 6 | 30.236 | −34.238 | −64.474 | −14.068 | −4.584 | 0.003 | 0.024 | * |
| 7942553 | SPCS2 | signal peptidase complex subunit 2 | 75.445 | −8.694 | −84.139 | −14.257 | 2.980 | 0.004 | 0.024 | * |
| 8178470 | POU5F1 | POU class 5 homeobox 1 | −9.583 | 3.829 | 13.413 | 15.998 | −1.384 | 0.004 | 0.025 | * |
| 8056753 | METTL8 | methyltransferase like 8 | 8.262 | −1.220 | −9.482 | −13.555 | −2.993 | 0.006 | 0.025 | * |
| 8010897 | METRNL | meteorin, glia lcell differentiation regulator-like | 26.260 | −21.690 | −47.950 | −34.664 | 6.662 | 0.006 | 0.025 | * |
| 7914180 | SPCS2 | signal peptidase complex subunit 2 | 93.422 | −4.750 | −98.172 | −23.683 | 1.362 | 0.006 | 0.024 | * |
| 8103188 | PET112L | Glutamyl-TRNA(Gln) amidotransferase, subunit B | 10.776 | −1.737 | −12.513 | −15.742 | −5.155 | 0.006 | 0.025 | * |
| 7955170 | WNT1 | wingless-type MMTV integration site family, member 1 | 4.786 | −5.369 | −10.155 | −14.789 | −2.448 | 0.007 | 0.025 | * |
| 7971208 | KBTBD6 | kelch repeat and BTB (POZ) domain containing 6 | 10.263 | −12.871 | −23.134 | −20.066 | −0.589 | 0.007 | 0.025 | * |
| 7966293 | C12orf76 | chromosome 12 open reading frame 76 | 6.877 | −4.379 | −11.255 | −22.647 | −5.121 | 0.007 | 0.025 | * |
| 8037723 | IRF2BP1 | interferon regulatory factor 2 binding protein 1 | 6.116 | −6.608 | −12.724 | −13.410 | −4.889 | 0.008 | 0.025 | * |
| 7977018 | TRAF3 | TNF receptor-associated factor 3 | 26.284 | −24.170 | −50.454 | −16.487 | −4.301 | 0.009 | 0.024 | * |
| 8011599 | ANKFY1 | ankyrin repeat and FYVE domain containing 1 | −51.618 | 16.158 | 67.776 | 17.717 | 0.372 | 0.009 | 0.024 | * |
| 7945539 | SLC25A22 | solute carrier family 25 (mitochondrial carrier: glutamate), member 22 | −12.679 | −0.443 | 12.236 | 12.430 | 4.242 | 0.010 | 0.025 | * |
| 8009761 | NUP85 | nucleoporin 85 kDa | −21.889 | 16.224 | 38.113 | 15.532 | 5.659 | 0.010 | 0.025 | * |
| 8149646 | NUDT18 | nudix (nucleoside diphosphatelinkedmoietyX)-type motif 18 | −12.158 | 3.280 | 15.438 | 18.741 | 7.270 | 0.011 | 0.025 | * |
| 8041967 | ERLEC1 | endoplasmic reticulum lectin 1 | 36.611 | −0.927 | −37.538 | −23.475 | −6.945 | 0.012 | 0.025 | * |
| 7953100 | FKBP4 | FK506 binding protein 4, 59 kDa | −3.656 | 14.527 | 18.183 | 11.740 | 2.848 | 0.012 | 0.025 | * |
| 8102024 | UBE2D3 | ubiquitin-conjugating enzyme E2D3 | 37.672 | −20.760 | −58.432 | −12.526 | −7.656 | 0.012 | 0.024 | * |
| 7960283 | CACNA2D4 | calcium channel, voltage-dependent, alpha 2/delta subunit 4 | −4.117 | 2.242 | 6.359 | 11.397 | 4.357 | 0.012 | 0.025 | * |
| 7973084 | ANG | angiogenin, ribonuclease, RNase A family, 5 | 1.177 | −7.173 | −8.349 | −13.689 | −4.175 | 0.013 | 0.025 | * |
| 8156838 | SEC61B | Sec61 translocon beta subunit | 57.494 | −21.264 | −78.758 | −19.412 | 0.154 | 0.013 | 0.024 | * |
| 8177782 | RPP21 | ribonuclease P/MRP 21 kDA subunit | 26.886 | −17.521 | −44.407 | −20.072 | −9.473 | 0.013 | 0.025 | * |
| 8179097 | RPP21 | ribonuclease P/MRP 21 kDA subunit | 26.886 | −17.521 | −44.407 | −20.072 | −9.473 | 0.013 | 0.025 | * |
| 8010454 | RNF213 | ring finger protein 213 | –219.912 | 40.539 | 260.451 | 16.923 | 1.278 | 0.014 | 0.022 | * |
| 7939569 | PRDM11 | PR domain containing 11 | 9.965 | −2.231 | −12.196 | −18.380 | −4.009 | 0.014 | 0.025 | * |
| 8103240 | ANXA2P1 | annexin A2 pseudogene 1 | −9.183 | 6.161 | 15.344 | 19.158 | 6.359 | 0.014 | 0.025 | * |
| 8173503 | RGAG4 | retrotransposon gag domain containing 4 | −10.364 | 1.697 | 12.061 | 23.428 | −1.841 | 0.014 | 0.025 | * |
| 8081335 | LOC100287852 | protein FAM136A pseudogene | −1.414 | 12.069 | 13.484 | 15.468 | 9.793 | 0.014 | 0.025 | * |
| 7901577 | LRRC42 | leucine rich repeat containing 42 | 9.232 | −3.146 | −12.379 | −15.250 | −6.284 | 0.015 | 0.025 | * |
| 8167573 | GAGE12B | G antigen 12B | −19.650 | 8.062 | 27.712 | 13.873 | 9.887 | 0.015 | 0.025 | * |
| 8009164 | DCAF7 | DDB1 and CUL4 associated factor 7 | –100.479 | −13.285 | 87.194 | 11.481 | 3.004 | 0.015 | 0.024 | * |
| 8140500 | TMEM60 | transmembrane protein 60 | 22.778 | −7.318 | −30.097 | −20.958 | −3.859 | 0.016 | 0.025 | * |
| 8023948 | C19orf20 | chromosome 19 open reading frame 20 | −9.870 | 4.464 | 14.334 | 19.466 | 9.650 | 0.018 | 0.025 | * |
| 8053449 | TMEM150A | transmembrane protein 150A | −13.909 | 8.840 | 22.749 | 14.502 | 4.889 | 0.019 | 0.025 | * |
| 7982531 | ANP32A | acidic (leucine-rich) nuclear phosphoprotein 32 family, member A | 89.064 | −90.788 | −179.852 | −14.750 | −5.476 | 0.020 | 0.023 | * |
| 7917338 | BCL10 | B-cell CLL/Lymphoma 10 | 23.728 | −5.530 | −29.258 | −16.560 | −4.576 | 0.021 | 0.025 | * |
| 7956978 | SLC35E3 | solute carrier family 35, member E3 | 18.739 | −18.207 | −36.946 | −17.220 | −6.586 | 0.021 | 0.025 | * |
| 8016320 | RPRML | reprimo-like | −7.365 | 6.073 | 13.438 | 18.927 | 1.074 | 0.022 | 0.025 | * |
| 7998577 | NME3 | NME/NM23 nucleoside disphosphate kinase 3 | 10.741 | −7.659 | −18.399 | −13.373 | −8.430 | 0.023 | 0.025 | * |
| 8053009 | FBXO41 | F-box protein 41 | −11.965 | −0.923 | 11.042 | 15.322 | 7.382 | 0.024 | 0.025 | * |
| 8164243 | TOR2A | torsin family 2, member A | 9.956 | −8.428 | −18.384 | −11.926 | −1.433 | 0.024 | 0.025 | * |
| 7910901 | LOC100130331 | POTE ankyrin domain family, member F pseudogene | 60.968 | −10.299 | −71.268 | −15.938 | −9.035 | 0.024 | 0.024 | * |
| 8117881 | RPP21 | ribonuclease P/MRP 21kDa submit | 17.027 | −13.978 | −31.005 | −14.448 | −5.787 | 0.024 | 0.025 |  |
| 7964466 | DCTN2 | dynactin 2 (P50) | 59.541 | 1.682 | −57.859 | −11.488 | −5.360 | 0.025 | 0.024 |  |
| 8157727 | GPR21 | G protein-coupled receptor 21 | −32.930 | 1.419 | 34.349 | 19.192 | 3.232 | 0.025 | 0.025 |  |
| 8048835 | MFF | Mitochondrial fission factor | 79.196 | 19.191 | −60.005 | −12.019 | 0.998 | 0.025 | 0.024 |  |
| 8131815 | KLHL7 | kelch-like family member 7 | 18.432 | −5.264 | −23.696 | −19.970 | −9.746 | 0.026 | 0.025 |  |
| 8123802 | MUTED | muted homolog (mouse) | 33.684 | −14.591 | −48.274 | −15.066 | −0.591 | 0.026 | 0.024 |  |
| 7982504 | PGBD4 | PiggyBac transposable element derived 4 | 14.674 | −10.118 | −24.793 | −24.644 | −2.294 | 0.026 | 0.025 |  |
| 7948399 | PATL1 | protein associated with topoisomerase II homolog 1 (yeast) | –149.596 | 27.491 | 177.087 | 13.283 | 6.591 | 0.026 | 0.023 |  |
| 8024228 | MIDN | midnolin | −29.088 | 32.110 | 61.199 | 10.639 | 9.332 | 0.027 | 0.024 |  |
| 8042211 | B3GNT2 | UDP-GlcNAc:BetaGal Beta-1, 3-*N*-acetylglucosaminyltransferase 2 | 14.809 | −8.001 | −22.810 | −24.381 | −3.348 | 0.028 | 0.025 |  |
| 7913415 | USP48 | ubiquitin specific peptidase 48 | 33.033 | −9.197 | −42.231 | −14.672 | −7.518 | 0.028 | 0.024 |  |
| 8074541 | TRMT2A | TRNA methyltransferase 2 homolog A | −22.860 | −0.691 | 22.169 | 13.558 | 9.011 | 0.028 | 0.025 |  |
| 8048980 | CAB39 | calcium binding protein 39 | 139.449 | −21.632 | −161.080 | −14.168 | −4.235 | 0.028 | 0.023 |  |
| 7911902 | NPHP4 | nephronophthisis 4 | 0.817 | −5.980 | −6.797 | −11.227 | −4.450 | 0.028 | 0.025 |  |
| 8007212 | STAT5A | signal transducer and activator oft ranscription 5A | −9.212 | 39.390 | 48.603 | 10.172 | 3.042 | 0.028 | 0.024 |  |
| 8121502 | GTF3C6 | general transcription factor IIIC, polypeptide 6, alpha 35kDa | 50.494 | 4.745 | −45.749 | −26.799 | −5.193 | 0.029 | 0.025 |  |
| 7970428 | ZMYM5 | zinc finger, MYM-type 5 | 25.934 | −10.655 | −36.589 | −17.408 | −9.204 | 0.029 | 0.025 |  |
| 7955589 | NR4A1 | nuclear receptor subfamily 4, group A, member 1 | −9.969 | 4.022 | 13.991 | 19.203 | −6.183 | 0.029 | 0.025 |  |
| 8009737 | KCTD2 | potassium channel tetramerisation domain containing 2 | 23.646 | −3.869 | −27.514 | −10.563 | −0.432 | 0.030 | 0.025 |  |
| 8175039 | ELF4 | E74-like factor 4 (ets domain transcription factor) | −73.395 | 4.439 | 77.835 | 11.450 | 5.223 | 0.031 | 0.024 |  |
| 7996715 | NRN1L | neuritin 1-like | 3.542 | −5.174 | −8.716 | −18.025 | −9.079 | 0.031 | 0.025 |  |
| 8136065 | LOC407835 | mitogen-activated protein kinase kinase 2 pseudogene | 1.282 | −8.182 | −9.464 | −11.773 | −6.861 | 0.031 | 0.025 |  |
| 7948775 | C11orf48 | chromosome 11 open reading frame 48 | 17.958 | −16.297 | −34.256 | −25.840 | −3.391 | 0.032 | 0.025 |  |
| 7957737 | TMPO | thymopoietin | 22.491 | −29.927 | −52.419 | −12.317 | −9.212 | 0.032 | 0.024 |  |
| 7983321 | CASC4 | cancer susceptibility candidate 4 | 74.827 | 3.174 | −71.652 | −16.183 | −3.704 | 0.033 | 0.024 |  |
| 8063531 | C20orf107 | chromosome 20 open reading frame 107 | −11.676 | 0.820 | 12.496 | 18.017 | 4.876 | 0.033 | 0.025 |  |
| 8138116 | ZNF12 | zinc finger protein 12 | 20.619 | −9.010 | −29.629 | −18.736 | −7.375 | 0.034 | 0.025 |  |
| 8016444 | HOXB3 | homeobox B3 | −9.891 | 3.817 | 13.708 | 20.959 | 6.350 | 0.034 | 0.025 |  |
| 8045182 | PTPN18 | protein tyrosine phosphatase, non-receptor type 18 (brain-derived) | −76.238 | −8.762 | 67.476 | 12.936 | −0.347 | 0.035 | 0.024 |  |
| 7949619 | SLC29A2 | solute carrier family 29 (equilibrative nucleoside transporters), member 2 | −12.846 | 2.871 | 15.717 | 15.850 | 7.900 | 0.036 | 0.025 |  |
| 8010426 | RNF213 | ring finger protein 213 | –304.771 | 56.768 | 361.539 | 34.436 | 6.919 | 0.036 | 0.023 |  |
| 8141305 | ZNF394 | zinc finger protein 394 | 83.618 | −2.411 | −86.029 | −15.674 | 4.269 | 0.036 | 0.024 |  |
| 8117655 | ZNF193 | zinc finger protein 193 | 3.324 | −8.126 | −11.450 | −12.901 | −5.757 | 0.037 | 0.025 |  |
| 8152222 | AZIN1 | antizyme inhibitor 1 | 59.708 | 4.054 | −55.654 | −16.497 | −9.929 | 0.037 | 0.024 |  |
| 8160805 | C9orf25 | chromosome 9 open reading frame 25 | −4.384 | 8.146 | 12.530 | 11.271 | 2.982 | 0.037 | 0.025 |  |
| 8015376 | KRT16 | keratin 16, Type 1 | −3.533 | 8.548 | 12.081 | 18.994 | 4.549 | 0.037 | 0.025 |  |
| 8137208 | ATP6V0E2 | ATPase, H+ transporting V0 subunit E2 | −23.153 | 11.620 | 34.773 | 13.365 | 7.428 | 0.038 | 0.025 |  |
| 7972737 | LIG4 | ligase IV, DNA, ATP-dependent | 17.320 | −3.787 | −21.106 | −19.506 | −5.949 | 0.038 | 0.025 |  |
| 7992205 | UBE2I | ubiquitin-conjugating enzyme E2I | 25.971 | 3.249 | −22.722 | −13.014 | −4.209 | 0.039 | 0.025 |  |
| 8025978 | ZNF763 | zinc finger protein 763 | 24.086 | 1.151 | −22.936 | −24.492 | −3.123 | 0.041 | 0.025 |  |
| 8036602 | ECH1 | enoyl CoA hydratase1, peroxisomal | 145.921 | −49.434 | −195.355 | −11.948 | −2.582 | 0.042 | 0.022 |  |
| 8024444 | EPWW6493 | Uncharacterized LOC100129831 | 18.756 | −29.101 | −47.858 | −23.902 | −8.111 | 0.043 | 0.025 |  |
| 7980381 | TMED8 | transmembrane P24 trafficking protein family member 8 | 43.162 | −16.734 | −59.896 | −20.057 | −7.419 | 0.043 | 0.024 |  |
| 8128429 | CCNC | cyclin C | 38.042 | 6.007 | −32.036 | −15.711 | 2.674 | 0.043 | 0.025 |  |
| 7992388 | NUBP2 | nucleotide binding protein 2 | 13.068 | −3.966 | −17.034 | −12.222 | 2.436 | 0.044 | 0.025 |  |
| 8056343 | COBLL1 | cordon-blue WH2 repeat protein-like 1 | 30.353 | −11.539 | −41.891 | −30.696 | 4.680 | 0.045 | 0.025 |  |
| 8026991 | PGPEP1 | pyroglutamyl-peptidase I | −12.020 | 5.347 | 17.366 | 13.192 | 0.905 | 0.045 | 0.025 |  |
| 8039695 | ZNF417 | zinc finger protein 417 | 19.820 | −18.132 | −37.952 | −18.264 | −2.670 | 0.046 | 0.025 |  |
| 7906146 | TMEM79 | transmembrane protein 79 | 2.821 | −5.369 | −8.189 | −10.153 | −5.054 | 0.047 | 0.025 |  |
| 7993622 | ITPRIPL2 | inositol 1, 4, 5-triphosphate receptor interacting protein-like 2 | −6.890 | 6.846 | 13.736 | 15.555 | 4.605 | 0.047 | 0.025 |  |
| 8078360 | STT3B | STT3, subunit of the oligosaccharyltransferase complex (catalytic) | 129.284 | 18.567 | −110.717 | −10.122 | 0.641 | 0.047 | 0.023 |  |
| 8157038 | SLC44A1 | solute carrier family 44 (choline transporter), member 1 | 27.797 | −6.793 | −34.589 | −17.345 | −5.081 | 0.047 | 0.025 |  |
| 8058914 | AAMP | angio-associated, migratory cell protein | 15.651 | −15.098 | −30.749 | −15.632 | −0.050 | 0.047 | 0.025 |  |
| 8132292 | 39326 | septin 7 | 63.979 | −1.826 | −65.805 | −20.176 | −8.469 | 0.048 | 0.024 |  |
| 8087485 | UBA7 | ubiquitin-like modifier activating enzyme 7 | −58.921 | −4.693 | 54.228 | 10.125 | 1.849 | 0.048 | 0.024 |  |
| 8112767 | TBCA | tubulin folding cofactor A | 33.589 | −19.536 | −53.125 | −19.821 | −5.936 | 0.048 | 0.024 |  |
| 8143772 | RARRES2 | retinoic acid receptor responder (tazarotene induced) 2 | −9.536 | 0.724 | 10.261 | 19.121 | 7.295 | 0.048 | 0.025 |  |
| 8076007 | BAIAP2L2 | BAI1-associated protein 2-like 2 | −5.742 | 0.956 | 6.697 | 13.077 | 2.637 | 0.048 | 0.025 |  |
| 7938293 | SNORA45 | small nucleolar RNA, H | 7.527 | −2.082 | −9.609 | −11.250 | −4.547 | 0.049 | 0.025 |  |
| 8155250 | GRHPR | glyoxylate reductase/hydroxypyruvate reductase | 18.941 | −11.889 | −30.830 | −15.177 | −2.734 | 0.049 | 0.025 |  |
| 7923528 | MYOG | myogenin (myogenic factor 4) | −4.246 | 3.781 | 8.028 | 14.403 | 8.843 | 0.049 | 0.025 |  |
| 7940924 | PLCB3 | phospholipase C, beta 3 (phosphatidylinositol-specific) | −15.965 | −0.983 | 14.982 | 16.209 | 6.831 | 0.050 | 0.025 |  |
| 8058512 | PLEKHM3 | pleckstrin homology domain containing, family M, member 3 | 33.499 | −14.269 | −47.769 | −10.696 | −4.377 | 0.050 | 0.024 |  |
| 7977657 | HNRNPC | heterogeneous nuclear ribonucleoprotein C(C1/C2) | 165.139 | −0.402 | −165.541 | −12.775 | −3.918 | 0.050 | 0.023 |  |
| 8150219 | BRF2 | BRF2, RNA polymerase III transcription initiation factor 50 KDa subunite | 11.081 | −2.845 | −13.926 | −12.110 | −2.058 | 0.050 | 0.025 |  |
| 7933298 | SYT15 | synaptotagmin XV | −10.642 | −2.979 | 7.663 | 14.811 | 6.684 | 0.050 | 0.025 |  |
| 8040949 | KRTCAP3 | keratinocyte associated protein 3 | −10.583 | −0.272 | 10.311 | 12.894 | 8.476 | 0.050 | 0.025 |  |
| 8047059 | HNRNPC | heterogeneous nuclear ribonucleoprotein C (C1/C2) | 100.926 | −10.347 | −111.273 | −10.777 | −6.950 | 0.051 | 0.023 |  |
| 8065510 | NANP | N-acetylneuraminic acid phosphatase | 7.350 | −1.274 | −8.624 | −18.662 | −7.437 | 0.052 | 0.025 |  |
| 7950751 | SNORA70E | Small nucleolar RNA, H/ACA Box 70E | 7.482 | −5.192 | −12.674 | −18.537 | −5.284 | 0.052 | 0.025 |  |
| 8137133 | KRBA1 | KRAB-A domain containing 1 | −6.038 | 3.280 | 9.318 | 12.231 | 7.138 | 0.053 | 0.025 |  |
| 8145660 | DCTN6 | dynactin 6 | 0.223 | 12.826 | 12.603 | 15.006 | 0.685 | 0.053 | 0.025 |  |
| 8174731 | RNF113A | ring finger protein 113A | 5.683 | −2.785 | −8.468 | −13.707 | −1.908 | 0.053 | 0.025 |  |
| 8059222 | DNPEP | aspartyl aminopeptidase | −22.408 | 1.988 | 24.396 | 10.858 | 5.182 | 0.054 | 0.025 |  |
| 8043476 | LOC652493 | ig kappa chain V-I region HK102-like | 205.316 | −63.655 | −268.971 | −62.667 | −1.436 | 0.054 | 0.024 |  |
| 8053366 | SUCLG1 | succinate-CoA ligase, alpha subunit | 24.456 | −11.365 | −35.821 | −15.993 | −3.761 | 0.055 | 0.025 |  |
| 7998692 | NTHL1 | nth –like DNA glycosylase 1 | 1.997 | −6.920 | −8.917 | −12.416 | −2.810 | 0.055 | 0.025 |  |
| 8006768 | SOCS7 | suppressor of cytokine signaling 7 | −13.606 | 2.332 | 15.938 | 13.658 | 8.977 | 0.056 | 0.025 |  |
| 8017810 | PSMD12 | proteasome 26S subunit, non-ATPase 12 | 16.441 | −6.701 | −23.142 | −15.719 | −7.142 | 0.057 | 0.025 |  |
| 8059731 | PDE6D | phosphodiesterase 6D, cGMP-specific, rod, delta | 51.548 | −3.460 | −55.008 | −14.830 | −3.665 | 0.058 | 0.024 |  |
| 8178080 | C6orf26 | chromosome 6 open reading frame 26 | −14.422 | 1.992 | 16.414 | 13.360 | 5.091 | 0.058 | 0.025 |  |
| 7918847 | SIKE1 | suppressor of IKBKE 1 | 31.927 | 2.418 | −29.508 | −16.852 | −7.097 | 0.058 | 0.025 |  |
| 8091009 | PIK3CB | phosphoinositide-4,5-bisphosphate 3-kinase, catalytic subunite beta | 28.734 | −25.521 | −54.255 | −11.607 | −7.271 | 0.059 | 0.024 |  |
| 8043564 | FAHD2A | fumarylacetoacetate hydrolase domain containing 2A | −34.089 | 7.502 | 41.591 | 24.468 | 9.632 | 0.059 | 0.025 |  |
| 8079753 | DAG1 | dystroglycan 1 (dystrophin-associated glycoprotein 1) | −11.728 | 0.047 | 11.775 | 11.927 | 7.886 | 0.059 | 0.025 |  |
| 7962151 | DENND5B | DENN/MADD domain containing 5B | 18.562 | −5.515 | −24.077 | −43.635 | −0.226 | 0.059 | 0.025 |  |
| 8043470 | IGKV3D-11 | immunoglobulin kappa variable 3D-11 | 210.624 | −59.453 | −270.077 | −83.210 | −1.498 | 0.059 | 0.024 |  |
| 8115076 | CSF1R | colony stimulating factor 1 receptor | −99.644 | −25.503 | 74.141 | 14.544 | −2.983 | 0.059 | 0.024 |  |
| 8053059 | BOLA3 | BolA family member 3 | 41.262 | −8.867 | −50.129 | −19.082 | −9.826 | 0.060 | 0.024 |  |
| 7963741 | SMUG1 | single-strand-selective monofunctional uracil-DNA glycosylase 1 | 10.613 | −6.472 | −17.085 | −15.131 | −4.829 | 0.061 | 0.025 |  |
| 8053949 | KIAA1310 | KAT8 regulatory NSL complex subunit 3 | −39.152 | −6.155 | 32.997 | 12.748 | −0.797 | 0.061 | 0.025 |  |
| 8178059 | LY6G5B | lymphocyte antigen 6 complex, locus G5B | −94.293 | −24.191 | 70.102 | 12.868 | 8.844 | 0.061 | 0.024 |  |
| 8015575 | GHDC | GH3 domain containing | −9.405 | 1.292 | 10.697 | 11.813 | 8.190 | 0.061 | 0.025 |  |
| 7932243 | FAM171A1 | family with sequence similarity 171, member A1 | −5.126 | 4.928 | 10.055 | 11.620 | 6.056 | 0.061 | 0.025 |  |
| 8083119 | RNF7 | ring finger protein 7 | 22.273 | −5.254 | −27.527 | −14.173 | −6.902 | 0.062 | 0.025 |  |
| 8173232 | FAM123B | APC membrane recruitment protein 1 | −0.232 | −12.444 | −12.212 | −14.600 | −7.772 | 0.062 | 0.025 |  |
| 7952927 | B4GALNT3 | beta-1, 4-*N*-acetyl-galactosaminyl transferase 3 | −4.018 | 3.954 | 7.972 | 13.338 | 5.514 | 0.063 | 0.025 |  |
| 8037688 | RSPH6A | Radial spoke head 6 homolog A (Chlamydomonas) | −7.650 | 0.049 | 7.698 | 13.169 | 8.380 | 0.063 | 0.025 |  |
| 7999102 | TFAP4 | transcription factor AP-4 (activating enhancer binding protein 4) | −14.580 | 3.215 | 17.796 | 15.566 | 6.611 | 0.063 | 0.025 |  |
| 8087568 | MON1A | MON1 secretory trafficking family member A | −8.913 | −0.125 | 8.788 | 14.945 | 1.287 | 0.064 | 0.025 |  |
| 7981387 | CDC42BPB | CDC42 binding protein kinase beta (DMPK-like) | −13.552 | 1.845 | 15.397 | 11.191 | 6.859 | 0.064 | 0.025 |  |
| 8045455 | UBXN4 | UBX domain protein 4 | 49.294 | −6.197 | −55.490 | −11.385 | −3.167 | 0.065 | 0.024 |  |
| 7924508 | SUSD4 | sushi domain containing 4 | −3.543 | 5.512 | 9.056 | 11.810 | 4.894 | 0.065 | 0.025 |  |
| 7968242 | GTF3A | general transcription factor IIIA | 38.511 | −8.878 | −47.389 | −19.012 | −4.638 | 0.065 | 0.025 |  |
| 7950235 | STARD10 | StAR-related lipid transfer (START) domain containing 10 | 29.094 | −3.897 | −32.990 | −13.234 | 4.659 | 0.065 | 0.025 |  |
| 8069985 | SNORA80 | small nucleolar RNA, H/ACA box80A | −13.365 | 4.509 | 17.873 | 15.361 | 7.746 | 0.065 | 0.025 |  |
| 8138489 | CDCA7L | cell division cycle associated 7-like | 22.853 | −11.192 | −34.045 | −21.502 | −3.339 | 0.065 | 0.025 |  |
| 8125134 | HSPA1L | heat shock 70k Da protein 1-like | 18.871 | −19.465 | −38.335 | −15.603 | −5.030 | 0.066 | 0.025 |  |
| 8178650 | HSPA1L | heat shock 70k Da protein 1-like | 18.871 | −19.465 | −38.335 | −15.603 | −5.030 | 0.066 | 0.025 |  |
| 8179848 | HSPA1L | heat shock 70k Da protein 1-like | 18.871 | −19.465 | −38.335 | −15.603 | −5.030 | 0.066 | 0.025 |  |
| 7958346 | C12orf23 | chromosome 12 open reading frame 23 | 7.846 | −11.380 | −19.226 | −15.712 | −3.161 | 0.066 | 0.025 |  |
| 8117330 | HIST1H3A | histone cluster 1, H3a | 7.405 | −3.845 | −11.250 | −17.361 | −1.851 | 0.066 | 0.025 |  |
| 8111941 | HMGCS1 | 3-hydroxy-3-methylglutaryl-CoA synthase1(soluble) | 8.303 | −1.371 | −9.674 | −10.118 | −0.344 | 0.067 | 0.025 |  |
| 7945730 | ASCL2 | achaete-scute family BHLH transcription factor 2 | −7.830 | 7.525 | 15.355 | 22.949 | 8.552 | 0.067 | 0.025 |  |
| 7962489 | PLEKHA9 | Pleckstrin homology domain containing, family A pseudogene 1 | 4.926 | −9.353 | −14.279 | −18.628 | −4.438 | 0.067 | 0.025 |  |
| 8033159 | PSPN | persephin | −7.177 | 2.219 | 9.397 | 16.951 | 4.301 | 0.067 | 0.025 |  |
| 8043431 | IGKC | immunoglobulin kappa constant | 54.224 | −22.153 | −76.377 | −18.197 | −9.186 | 0.068 | 0.024 |  |
| 8062480 | SNHG11 | small nucleolar RNA host gene 11 | −7.812 | 1.360 | 9.171 | 13.498 | 7.036 | 0.069 | 0.025 |  |
| 7980403 | VIPAR | VPS33B interacting protein, apical-basolateral polarity regulator, spe-39 homolog | 39.236 | −4.421 | −43.657 | −10.839 | −9.025 | 0.069 | 0.024 |  |
| 8143759 | ATP6V0E2 | ATPase, H+ transporting V0 subunit E2 | −14.227 | 1.288 | 15.515 | 19.261 | −1.856 | 0.069 | 0.025 |  |
| 8091648 | SSR3 | signal sequence receptor, gamma (translocon-associated protein gamma) | 44.199 | −12.855 | −57.054 | −16.901 | 2.675 | 0.069 | 0.024 |  |
| 8151788 | RBM12B | RNA binding motif protein 12B | 25.840 | −12.676 | −38.516 | −21.142 | −8.576 | 0.069 | 0.025 |  |
| 8121704 | NUS1 | NUS1 dehydrodolichyl disphosphate synthase subunit | 27.221 | −20.985 | −48.206 | −11.214 | −2.860 | 0.069 | 0.024 |  |
| 8137336 | ACCN3 | acid sensing (proton gated) ion channel 3 | −10.166 | 3.553 | 13.719 | 20.633 | 8.975 | 0.069 | 0.025 |  |
| 8168345 | ACRC | acidic repeat containing | −9.187 | 2.794 | 11.981 | 19.489 | −2.045 | 0.070 | 0.025 |  |
| 8164008 | NCRNA00287 | MIR600 host gene | 19.645 | −6.812 | −26.457 | −18.217 | 7.602 | 0.070 | 0.025 |  |
| 7945896 | CHRNA10 | cholinergic receptor, nicotinic, alpha 10 (neuronal) | −11.943 | 3.683 | 15.626 | 18.364 | 6.219 | 0.070 | 0.025 |  |
| 8119198 | FTSJD2 | cap methyltransferase 1 | −45.605 | 26.973 | 72.577 | 19.534 | 2.823 | 0.071 | 0.024 |  |
| 8131406 | RAC1 | ras-related C3 botulinum toxin substrate 1(Rho family, small GTP binding protein Rac1) | 24.584 | −34.811 | −59.395 | −13.865 | −7.176 | 0.071 | 0.024 |  |
| 8078380 | ZNF860 | zinc finger protein 860 | 20.993 | −0.799 | −21.792 | −35.047 | 2.670 | 0.072 | 0.025 |  |
| 7950555 | LRRC32 | leucine rich repeat containing 32 | −7.573 | 2.572 | 10.144 | 19.599 | 2.390 | 0.072 | 0.025 |  |
| 8000941 | ZNF629 | zinc finger protein 629 | −2.545 | 5.587 | 8.132 | 13.922 | 0.344 | 0.072 | 0.025 |  |
| 8013120 | PEMT | phosphatidylethanolamine N-methyltransferase | 22.393 | −6.371 | −28.764 | −13.286 | 7.089 | 0.072 | 0.025 |  |
| 7950654 | ALG8 | ALG8, alpha-1, 3-glucosyltransferase | 36.721 | −1.737 | −38.457 | −13.765 | −3.007 | 0.073 | 0.024 |  |
| 7965112 | PAWR | PRKC, apoptosis, WT1, regulator | 9.164 | −4.180 | −13.344 | −22.141 | −5.599 | 0.073 | 0.025 |  |
| 8028227 | ZNF383 | zinc finger protein 383 | 43.059 | −10.801 | −53.860 | −18.928 | −0.740 | 0.074 | 0.024 |  |
| 7939093 | DNAJC24 | DnaJ (Hsp40) homolog, subfamily C, member 24 | 12.561 | −4.469 | −17.030 | −23.668 | −5.354 | 0.075 | 0.025 |  |
| 7972828 | ANKRD10 | ankyrin repeat domain 10 | 41.140 | −4.563 | −45.703 | −13.174 | −3.153 | 0.076 | 0.024 |  |
| 8004832 | RANGRF | RAN guanine nucleotide release factor | 13.948 | −1.071 | −15.019 | −16.406 | −5.796 | 0.076 | 0.025 |  |
| 8008887 | RPS6KB1 | ribosomal protein S6 kinase, 70kDa, polypeptide 1 | 27.310 | −7.157 | −34.467 | −11.548 | −3.584 | 0.077 | 0.024 |  |
| 8093916 | MAN2B2 | mannosidase, alpha, class 2B, member 2 | −54.131 | −1.691 | 52.440 | 10.339 | 6.260 | 0.077 | 0.024 |  |
| 8119801 | ABCC10 | ATP-bindingcassette, sub-family C (CFTR/MRP), member 10 | −31.786 | −3.105 | 28.681 | 13.071 | 6.561 | 0.077 | 0.025 |  |
| 8164013 | STRBP | spermatid perinuclear RNA binding protein | 21.422 | 0.397 | −21.024 | −19.482 | 3.364 | 0.078 | 0.025 |  |
| 7962811 | C12orf41 | chromosome 12 open reading frame 41 | 20.133 | −10.407 | −30.540 | −18.387 | −9.301 | 0.078 | 0.025 |  |
| 8074498 | TXNRD2 | thioredoxin reductase 2 | 0.614 | −21.477 | −22.091 | −12.470 | −8.580 | 0.079 | 0.025 |  |
| 8084634 | DNAJB11 | DnaJ (Hsp40) homolog, subfamily B, member 11 | 16.288 | −1.718 | −18.006 | −13.394 | −3.730 | 0.079 | 0.025 |  |
| 8076424 | CYP2D6 | cytochrome P450, family 2, subfamily D, polypeptide 6 | −15.842 | 3.038 | 18.881 | 21.235 | 5.069 | 0.080 | 0.025 |  |
| 8005458 | LGALS9C | lectin, galactoside-binding, soluble, 9C | −60.863 | 30.312 | 91.176 | 16.572 | 4.237 | 0.080 | 0.024 |  |
| 7941457 | CCDC85B | coiled-coil domain containing 85B | −3.745 | 6.780 | 10.524 | 12.184 | 3.306 | 0.081 | 0.025 |  |
| 8119627 | PPP2R5D | protein phosphatase 2, regulatory subunitB', delta | −17.266 | 12.628 | 29.893 | 10.757 | 3.961 | 0.081 | 0.025 |  |
| 8000932 | C16orf93 | chromosome 16 open reading frame 93 | 11.123 | −1.667 | −12.790 | −17.208 | −2.806 | 0.081 | 0.025 |  |
| 7928516 | SAMD8 | sterile alpha motif domain containing 8 | 21.657 | −14.767 | −36.424 | −10.838 | −6.155 | 0.082 | 0.024 |  |
| 8039692 | ZNF814 | zinc finger protein 814 | 63.259 | 5.534 | −57.725 | −28.806 | −6.558 | 0.082 | 0.025 |  |
| 8114158 | PPP2CA | protein phosphatase 2, catalytic subunit, alpha isozyme | 58.417 | −1.103 | −59.520 | −10.046 | −8.137 | 0.082 | 0.024 |  |
| 8051113 | ZNF513 | zinc finger protein 513 | −27.333 | −1.807 | 25.525 | 12.322 | 8.630 | 0.082 | 0.025 |  |
| 8039530 | ZNF784 | zinc finger protein 784 | 7.479 | −6.713 | −14.193 | −10.662 | −1.259 | 0.082 | 0.025 |  |
| 7962367 | ZCRB1 | zinc finger CCHC-type and RNA binding motif 1 | 27.522 | −0.390 | −27.913 | −27.781 | −7.152 | 0.082 | 0.025 |  |
| 7940884 | NUDT22 | Nudix (nucleoside diphosphate linked moietyX)-type motif 22 | −15.110 | 0.344 | 15.454 | 11.041 | 7.839 | 0.083 | 0.025 |  |
| 8061129 | C20orf72 | chromosome 20 open reading frame 72 | 17.427 | −8.855 | −26.282 | −15.570 | −7.039 | 0.083 | 0.025 |  |
| 8020183 | IMPA2 | inositol(myo)-1(or 4)-monophosphatase 2 | 17.191 | −21.203 | −38.395 | −15.565 | −3.012 | 0.083 | 0.025 |  |
| 7970395 | TUBA3C | tubulin, alpha 3c | −3.839 | 8.163 | 12.002 | 15.975 | 7.284 | 0.083 | 0.025 |  |
| 8080923 | PSMC1 | proteasome 26S submit, ATPase, 1 | 31.576 | 5.722 | −25.854 | −10.265 | −2.605 | 0.084 | 0.025 |  |
| 8118228 | LY6G5B | lymphocyte antigen 6 complex, locus G5B | −66.891 | −11.441 | 55.450 | 13.811 | 8.943 | 0.084 | 0.024 |  |
| 7911413 | TNFRSF4 | tumor necrosis factor receptor superfamily, member 4 | −5.585 | 4.660 | 10.244 | 13.891 | 9.529 | 0.084 | 0.025 |  |
| 7963965 | WIBG | PYM homolog 1, exon junction complex associated factor | 3.628 | −9.855 | −13.483 | −12.697 | −4.046 | 0.084 | 0.025 |  |
| 8073032 | TOMM22 | translocase of outer mitochondrial membrane 22 homolog (yeast) | 83.315 | −44.022 | −127.337 | −75.497 | 2.379 | 0.084 | 0.025 |  |
| 7977418 | C14orf80 | chromosome 14 open reading frame 80 | −9.810 | −0.396 | 9.414 | 12.057 | 5.660 | 0.084 | 0.025 |  |
| 7995492 | ADCY7 | adenylate cyclase 7 | −87.357 | −18.162 | 69.195 | 13.209 | 6.413 | 0.085 | 0.024 |  |
| 7991017 | LOC283693 | actin, gamma pseudogene | 5.899 | −4.167 | −10.066 | −16.712 | −2.988 | 0.085 | 0.025 |  |
| 7929550 | CCNJ | cyclin J | 10.962 | −9.968 | −20.930 | −14.092 | −6.946 | 0.085 | 0.025 |  |
| 7930264 | NEURL | neutralized E3 ubiquitin protein ligase 1 | −6.532 | 9.059 | 15.591 | 14.547 | 4.034 | 0.085 | 0.025 |  |
| 7912292 | LZIC | leucine zipper and CTNNBIP1 domain containing | 33.792 | 5.828 | −27.963 | −14.341 | −6.923 | 0.086 | 0.025 |  |
| 7938812 | TMEM86A | transmembrane protein 86A | −8.116 | 2.044 | 10.160 | 14.794 | 4.340 | 0.086 | 0.025 |  |
| 8070665 | SIK1 | salt-inducible kinase 1 | −6.069 | 2.593 | 8.661 | 11.924 | 6.889 | 0.087 | 0.025 |  |
| 8026915 | ARRDC2 | arrestin domain containing 2 | −32.905 | 10.049 | 42.955 | 10.856 | −1.888 | 0.087 | 0.024 |  |
| 8105612 | CWC27 | CWC27 spliceosome-associated protein homolog | 15.234 | −8.558 | −23.792 | −17.902 | −6.746 | 0.087 | 0.025 |  |
| 8002547 | ZNF19 | zinc finger protein 19 | −8.951 | −1.690 | 7.261 | 11.217 | 8.372 | 0.087 | 0.025 |  |
| 7967987 | MRP63 | mitochondrial ribosomal protei 63 | 12.425 | −1.806 | −14.231 | −14.867 | −0.240 | 0.087 | 0.025 |  |
| 8086141 | EPM2AIP1 | EPM2A (laforin) interacting protein 1 | 40.594 | −8.212 | −48.806 | −19.475 | −5.199 | 0.088 | 0.024 |  |
| 8003263 | ZCCHC14 | zinc finger, CCHC domain containing 14 | −15.327 | 7.029 | 22.356 | 19.710 | −1.392 | 0.088 | 0.025 |  |
| 7922104 | GCSH | glycine cleavage system protein H(aminomethyl carrier) | 9.933 | −0.799 | −10.732 | −18.275 | −7.136 | 0.088 | 0.025 |  |
| 8038993 | ZNF28 | zinc finger protein 28 | 21.561 | −3.361 | −24.922 | −26.472 | −9.947 | 0.088 | 0.025 |  |
| 8045291 | TUBA3D | tubulin, alpha 3d | −3.714 | 3.187 | 6.901 | 11.381 | 9.674 | 0.088 | 0.025 |  |
| 8053690 | IGK | immunoglobulin kappa locus | 53.615 | −17.297 | −70.913 | −13.704 | 3.336 | 0.089 | 0.024 |  |
| 8027932 | COX6B1 | cytochrome C oxidase subunit VIb polypeptide 1 (ubiquitous) | 54.762 | −23.670 | −78.432 | −11.577 | −8.478 | 0.089 | 0.024 |  |
| 8043449 | IGK | immunoglobulin kappa locus | 215.631 | −83.008 | −298.639 | −96.258 | 6.756 | 0.089 | 0.024 |  |
| 8043360 | IGK | immunoglobulin kappa locus | 140.861 | −36.280 | −177.141 | −139.235 | 3.625 | 0.089 | 0.025 |  |
| 8130374 | FBXO5 | F-box protein 5 | 11.657 | 2.316 | −9.342 | −15.005 | −4.975 | 0.090 | 0.025 |  |
| 8169882 | BCORL1 | BCL6 corepressor-like 1 | −19.185 | 4.695 | 23.880 | 12.357 | −1.730 | 0.090 | 0.025 |  |
| 7981601 | IGHA2 | immunoglobulin heavy constant alpha 2 (A2m marker) | 69.991 | −33.312 | −103.303 | −40.499 | 6.533 | 0.091 | 0.024 |  |
| 8123006 | SYNJ2 | synaptojanin 2 | −7.223 | 6.060 | 13.282 | 10.532 | 0.697 | 0.092 | 0.025 |  |
| 8164217 | FAM129B | family with sequence similarity 129, member B | −13.098 | −0.910 | 12.188 | 10.123 | −0.902 | 0.092 | 0.025 |  |
| 8014047 | C17orf42 | chromosome 17 open reading frame 42 | 16.707 | −8.116 | −24.823 | −16.319 | −4.958 | 0.092 | 0.025 |  |
| 8043459 | IGKC | immunoglobulin kappa constant | 121.620 | −45.652 | −167.272 | −54.470 | −3.624 | 0.092 | 0.024 |  |
| 8039748 | A1BG | alpha-1-B glycoprotein | −7.612 | 0.142 | 7.754 | 11.167 | 0.362 | 0.092 | 0.025 |  |
| 8038981 | ZNF611 | zinc finger protein 611 | 12.448 | −10.881 | −23.329 | −17.970 | −5.677 | 0.092 | 0.025 |  |
| 7985016 | SNX33 | sorting nexin 33 | −0.886 | −11.192 | −10.306 | −14.694 | −5.029 | 0.093 | 0.025 |  |
| 8078544 | MLH1 | mutL homolog 1 | 23.117 | −2.250 | −25.367 | −10.251 | −7.319 | 0.093 | 0.025 |  |
| 8134098 | CDK14 | cyclin-dependent kinase 14 | 38.176 | −10.744 | −48.920 | −18.256 | 0.243 | 0.093 | 0.025 |  |
| 7924055 | C1orf74 | chromosome 1 open reading frame 74 | −8.081 | 2.831 | 10.913 | 13.916 | 8.359 | 0.093 | 0.025 |  |
| 8006634 | PIGW | phosphatidylinositol glycan anchor biosynthesis, class W | 3.555 | −3.647 | −7.203 | −13.432 | 2.089 | 0.093 | 0.025 |  |
| 8018236 | FDXR | ferredoxin reductase | −8.129 | −0.907 | 7.222 | 11.391 | 8.069 | 0.094 | 0.025 |  |
| 7940698 | TAF6L | TAF6-like RNA polymerase II, P300/CBP-associated factor (PCAF)-associated factor, 65kDa | 3.738 | −9.515 | −13.253 | −11.382 | 2.270 | 0.094 | 0.025 |  |
| 8091550 | KIAA1328 | KIAA1328 | 39.278 | 1.659 | −37.619 | −25.516 | 9.809 | 0.094 | 0.025 |  |
| 7928411 | KIAA0913 | KIAA0913 | −43.297 | −3.763 | 39.534 | 15.068 | 1.881 | 0.094 | 0.025 |  |
| 8036483 | YIF1B | Yip 1 interacting factor homolog B (S.cerevisiae) | 8.776 | −16.526 | −25.302 | −10.720 | −5.305 | 0.095 | 0.025 |  |
| 8101992 | SLC39A8 | solute carrier family 39 (zinc transporter), member 8 | 24.763 | −7.492 | −32.255 | −14.698 | −0.520 | 0.095 | 0.025 |  |
| 8032863 | C19orf10 | chromosome 19 open reading frame 10 | 20.147 | −6.770 | −26.916 | −15.606 | −1.306 | 0.095 | 0.025 |  |
| 7950490 | MIR326 | microRNA 326 | −28.122 | 1.229 | 29.352 | 15.360 | −1.293 | 0.095 | 0.025 |  |
| 8088535 | PSMD6 | proteasome 26S subunit, non-ATPase 6 | 17.770 | 2.020 | −15.750 | −12.628 | −8.027 | 0.096 | 0.025 |  |
| 8115806 | UBTD2 | ubiquitin domainccontainingc2 | 15.742 | −0.418 | −16.160 | −15.547 | −5.878 | 0.097 | 0.025 |  |
| 7963139 | BCDIN3D | BCDIN3 domain containing | 5.265 | −10.897 | −16.162 | −20.771 | −5.443 | 0.097 | 0.025 |  |
| 7970676 | SHISA2 | shisa family member 2 | −13.262 | 0.265 | 13.528 | 22.128 | 2.831 | 0.097 | 0.025 |  |
| 7991668 | GOLGA8F | golgin A8 family, member F | −3.493 | 2.512 | 6.004 | 11.319 | 7.136 | 0.098 | 0.025 |  |
| 8153819 | CYHR1 | cysteine/histidine-rich 1 | 26.389 | −11.020 | −37.410 | −13.805 | −9.204 | 0.098 | 0.024 |  |
| 8143279 | ZC3HAV1 | zinc finger CCCH-type, antiviral 1 | −76.544 | 79.886 | 156.430 | 11.614 | 3.089 | 0.098 | 0.023 |  |
| 8048647 | STK11IP | serine/threonine kinase 11 interacting protein | −11.731 | −0.642 | 11.090 | 11.839 | 0.645 | 0.098 | 0.025 |  |
| 8074606 | USP18 | ubiquitin specific peptidase 18 | −21.638 | 5.548 | 27.186 | 25.526 | 6.268 | 0.098 | 0.025 |  |
| 7922646 | TOR1AIP2 | torsin A interacting protein 2 | 58.951 | 0.372 | −58.579 | −14.831 | −6.540 | 0.099 | 0.024 |  |
| 7988414 | GATM | glycine amidinotransferase (l-arginine:glycine amidinotransferase) | 7.844 | −4.376 | −12.220 | −18.929 | 6.708 | 0.099 | 0.025 |  |
| 8101648 | HSD17B11 | hydroxysteroid (17-beta) dehydrogenase 11 | 194.980 | −15.726 | −210.706 | −13.985 | −7.627 | 0.099 | 0.022 |  |
| 8102247 | LOC285456 | hypothetical LOC285456 | 2.810 | −12.202 | −15.011 | −17.609 | −6.760 | 0.100 | 0.025 |  |
| 7941364 | PCNXL3 | pecanex-like 3 (Drosophila) | −47.293 | −7.448 | 39.845 | 10.193 | 7.022 | 0.100 | 0.024 |  |
| 8055445 | DARS | aspartyl-tRNA synthetase | 44.220 | 3.254 | −40.966 | −10.791 | −1.494 | 0.100 | 0.024 |  |
| 8055672 | MMADHC | methylmalonic aciduria (cobalamin deficiency) CblD type, with homocystinuria | 78.192 | 12.748 | −65.444 | −14.322 | −5.696 | 0.101 | 0.024 |  |
| 7937106 | NKX6-2 | NK6 homeobox 2 | −3.794 | 3.362 | 7.156 | 13.539 | 6.903 | 0.101 | 0.025 |  |
| 8102643 | CCNA2 | cyclin A2 | 5.454 | −2.527 | −7.981 | −13.652 | −6.537 | 0.102 | 0.025 |  |
| 8120992 | ZNF292 | zinc finger protein 292 | 95.337 | −1.181 | −96.518 | −14.261 | −3.816 | 0.102 | 0.024 |  |
| 8096224 | AFF1 | AF4/MR2 family, member 1 | −55.776 | 63.611 | 119.387 | 11.913 | −0.036 | 0.102 | 0.023 |  |
| 8072004 | IGLL3P | immunoglobulin lambda-like polypeptide 3, pseudogene | 5.799 | −17.118 | −22.917 | −12.506 | 0.797 | 0.103 | 0.025 |  |
| 7980327 | ANGEL1 | angel homolog 1 (Drosophila) | −21.498 | 9.288 | 30.787 | 10.791 | 6.370 | 0.103 | 0.025 |  |
| 8002029 | ZDHHC1 | zinc finger, DHHC-type containing1 | −7.281 | 2.627 | 9.909 | 14.888 | 6.625 | 0.103 | 0.025 |  |
| 8159945 | AK3 | adenylate kinase 3 | 19.317 | −5.016 | −24.332 | −13.895 | −1.443 | 0.103 | 0.025 |  |
| 7956470 | MBD6 | methyl-CpG binding domain protein 6 | −55.877 | 7.262 | 63.139 | 14.392 | 8.454 | 0.103 | 0.024 |  |
| 8008237 | ITGA3 | integrin, alpha 3 (antigen CD49C, alpha 3 subunit of VLA-3 receptor) | −9.460 | −1.473 | 7.987 | 11.648 | 8.024 | 0.104 | 0.025 |  |
| 8123951 | C6orf105 | chromosome 6 open reading frame 105 | −2.676 | 5.146 | 7.823 | 16.185 | 2.761 | 0.104 | 0.025 |  |
| 7950743 | RAB30 | RAB30, member RAS oncogene family | 25.116 | −6.881 | −31.997 | −18.028 | 4.743 | 0.105 | 0.025 |  |
| 8003939 | TM4SF5 | transmembrane 4 L six family member 5 | −1.562 | 4.882 | 6.445 | 11.902 | 9.262 | 0.105 | 0.025 |  |
| 8126474 | MEA1 | male-enhanced antigen 1 | 43.923 | 12.281 | −31.642 | −10.732 | 0.556 | 0.105 | 0.024 |  |
| 7981732 | IGHV4-59 | immunoglobulin heavy variable 4-59 | 290.606 | –102.497 | −393.103 | −52.898 | 9.333 | 0.105 | 0.023 |  |
| 7900597 | C1orf50 | chromosome 1 open reading frame 50 | 10.768 | −5.695 | −16.463 | −15.916 | −6.528 | 0.106 | 0.025 |  |
| 8157203 | DNAJC25 | DnaJ (Hsp40) homolog, subfamily C, member 25 | 12.946 | −5.699 | −18.645 | −11.643 | −0.346 | 0.106 | 0.025 |  |
| 7927091 | LOC399744 | hypothetical LOC399744 | −13.234 | 2.037 | 15.271 | 10.624 | 5.324 | 0.106 | 0.025 |  |
| 7948424 | MRPL16 | mitochondrial ribosomal protein L16 | 16.506 | −6.727 | −23.233 | −16.772 | −5.271 | 0.106 | 0.025 |  |
| 8041000 | GPN1 | GPN-loop GTPase 1 | 21.580 | 0.198 | −21.382 | −11.148 | 2.609 | 0.106 | 0.025 |  |
| 8029939 | GLTSCR1 | glioma tumor suppressor candidate region gene 1 | −19.263 | 0.564 | 19.827 | 12.402 | 6.685 | 0.107 | 0.025 |  |
| 8112584 | ZNF366 | zinc finger protein 366 | −7.438 | −0.477 | 6.961 | 13.560 | 3.125 | 0.107 | 0.025 |  |
| 7918188 | SLC25A24 | solute carrier family 25 (mitochondrial carrier; phosphate carrier), member 24 | 23.951 | −3.917 | −27.868 | −18.046 | −2.723 | 0.107 | 0.025 |  |
| 7935730 | CWF19L1 | CWF19-like 1, cell cycle control (S.pombe) | 25.982 | −10.469 | −36.451 | −13.149 | 2.269 | 0.107 | 0.024 |  |
| 7960894 | LOC389634 | hypothetical LOC389634 | −16.739 | −0.831 | 15.907 | 11.237 | 4.471 | 0.107 | 0.025 |  |
| 8080084 | MANF | mesencephalic astrocyte-derived neurotrophic factor | 31.951 | −1.211 | −33.162 | −11.422 | 1.474 | 0.107 | 0.024 |  |
| 7902382 | RABGGTB | Rab geranylgeranyltransferase, beta subunit | 32.417 | −1.676 | −34.092 | −17.565 | −6.899 | 0.108 | 0.025 |  |
| 8107655 | CSNK1G3 | casein kinase 1, gamma 3 | 24.719 | 2.163 | −22.556 | −12.159 | −2.761 | 0.108 | 0.025 |  |
| 7991367 | C15orf38 | chromosome 15 open reading frame 38 | −24.632 | −3.318 | 21.314 | 16.283 | 6.901 | 0.108 | 0.025 |  |
| 7899096 | ZNF593 | zinc finger protein 593 | 8.835 | −3.641 | −12.476 | −10.263 | −1.131 | 0.108 | 0.025 |  |
| 7935002 | SRP9 | signal recognition particle 9kDa | 71.814 | −1.800 | −73.614 | −14.956 | −7.077 | 0.109 | 0.024 |  |
| 8044965 | TSN | Translin | 9.128 | −6.910 | −16.038 | −10.948 | −7.615 | 0.109 | 0.025 |  |
| 8126095 | C6orf129 | chromosome 6 open reading frame 129 | 5.236 | −4.462 | −9.698 | −15.003 | −7.271 | 0.109 | 0.025 |  |
| 7913593 | TCEA3 | transcription elongation factor A (SII), 3 | −11.675 | 3.169 | 14.844 | 13.277 | 4.553 | 0.109 | 0.025 |  |
| 8045381 | CCNT2 | cyclin T2 | 65.763 | 8.903 | −56.860 | −10.050 | −2.391 | 0.109 | 0.024 |  |
| 8005501 | FAM18B2 | trans-golgi network vesicle protein 23 homolog C (S. Cervisiae) | 28.658 | 1.675 | −26.983 | −14.755 | −6.375 | 0.109 | 0.025 |  |
| 8059864 | GBX2 | gastrulation brain homeobox 2 | −3.315 | 10.021 | 13.336 | 17.314 | 9.986 | 0.109 | 0.025 |  |
| 8157362 | ZNF618 | zinc finger protein 618 | −12.528 | −1.606 | 10.922 | 14.279 | 3.399 | 0.110 | 0.025 |  |
| 7993019 | NUDT16L1 | nudix (nucleoside diphosphate linked moiety X)-type motif 16-like 1 | −10.780 | 7.052 | 17.831 | 10.528 | 9.675 | 0.111 | 0.025 |  |
| 8008132 | ATP5G1 | ATP synthase, H+ transporting, mitochondrial Fo complex, subunit C1 (subunit 9) | 57.258 | −26.987 | −84.245 | −12.237 | 8.549 | 0.111 | 0.024 |  |
| 8073192 | UQCRFS1 | ubiquinol-cytochrome C reductase, Rieske iron-sulfur polypeptide 1 | 35.882 | −3.516 | −39.397 | −10.870 | 2.289 | 0.112 | 0.024 |  |
| 7992147 | CACNA1H | calcium channel, voltage-dependent, T type, alpha 1H subunit | −8.590 | −1.385 | 7.204 | 11.567 | 6.398 | 0.113 | 0.025 |  |
| 8099541 | QDPR | quinoid dihydropteridine reductase | 6.298 | −6.686 | −12.983 | −10.716 | 0.282 | 0.113 | 0.025 |  |
| 8063566 | RAE1 | ribonucleic acid export 1 | 20.424 | −1.936 | −22.360 | −12.137 | −4.522 | 0.113 | 0.025 |  |
| 7939897 | FOLH1 | folate hydrolase (prostate-specific membrane antigen) 1 | −16.624 | 5.972 | 22.597 | 37.154 | 1.494 | 0.113 | 0.025 |  |
| 7955217 | SPATS2 | spermatogenesis associated, serine-rich 2 | 20.974 | −0.219 | −21.193 | −26.911 | −2.456 | 0.114 | 0.025 |  |
| 8008530 | UTP18 | UTP18 small subunit (SSU) processome component, homolog (yeast) | 26.922 | −6.239 | −33.161 | −18.087 | −7.215 | 0.114 | 0.025 |  |
| 8049950 | LOC100131763 | uncharacterized LOC100131763 | −9.439 | −0.869 | 8.569 | 15.558 | 7.967 | 0.114 | 0.025 |  |
| 7932285 | FAM188A | family with sequence similarity 188, member A | 28.194 | 1.317 | −26.876 | −12.789 | −5.510 | 0.115 | 0.025 |  |
| 8135197 | SPDYE2 | Speedy/RINGO cell cycle regulator family member E2 | −9.867 | 2.411 | 12.278 | 15.660 | 8.579 | 0.115 | 0.025 |  |
| 8150988 | ASPH | aspartate beta-hydroxylase | 18.491 | −16.978 | −35.468 | −21.897 | −1.595 | 0.116 | 0.025 |  |
| 8169969 | PHF6 | PHD finger protein 6 | 17.683 | −1.131 | −18.814 | −13.580 | −2.979 | 0.116 | 0.025 |  |
| 8103206 | FBXW7 | F-box and WD repeat domain containing 7, E3 ubiquitin protein ligase | 21.449 | −3.522 | −24.971 | −11.382 | −5.077 | 0.117 | 0.025 |  |
| 8175947 | HCFC1 | host cell factor C1 | –108.963 | −20.211 | 88.752 | 18.657 | 0.679 | 0.117 | 0.024 |  |
| 7929768 | CUTC | CutC copper transporter | 40.113 | 0.773 | −39.341 | −14.059 | −8.433 | 0.117 | 0.024 |  |
| 8049876 | BOK | BCL2-related ovarian killer | −10.191 | 5.023 | 15.215 | 14.786 | 9.405 | 0.117 | 0.025 |  |
| 8028607 | IL28A | interferon, lambda 2 | 3.986 | −6.870 | −10.857 | −21.446 | 0.385 | 0.118 | 0.025 |  |
| 8080911 | KBTBD8 | kelch repeat and BTB (POZ) domain containing 8 | 7.697 | −2.492 | −10.190 | −14.742 | 7.022 | 0.118 | 0.025 |  |
| 7912343 | CASZ1 | castor zinc finger 1 | −13.362 | 4.227 | 17.589 | 19.721 | 3.173 | 0.118 | 0.025 |  |
| 7925918 | AKR1C1 | aldo-keto reductase family 1, member C1 | 11.470 | −8.288 | −19.758 | −27.008 | −5.717 | 0.118 | 0.025 |  |
| 8038949 | ZNF841 | zinc finger protein 841 | 22.555 | −9.808 | −32.363 | −22.620 | −6.263 | 0.119 | 0.025 |  |
| 8104072 | FLJ38576 | uncharacterized LOC651430 | 10.834 | 0.812 | −10.022 | −16.852 | −6.401 | 0.119 | 0.025 |  |
| 8156309 | GADD45G | growth arrest and DNA-damage-inducible, gamma | 4.148 | −3.375 | −7.523 | −11.230 | −0.531 | 0.119 | 0.025 |  |
| 8080762 | ABHD6 | abhydrolase domain containing 6 | −14.480 | 3.338 | 17.818 | 13.678 | 4.485 | 0.120 | 0.025 |  |
| 8083272 | GYG1 | glycogenin 1 | 39.343 | −4.492 | −43.835 | −14.130 | −8.851 | 0.120 | 0.024 |  |
| 8039791 | ZBTB45 | zinc finger and BTB domain containing 45 | −18.257 | −3.602 | 14.656 | 10.947 | 3.831 | 0.120 | 0.025 |  |
| 7938608 | SPON1 | spondin 1, extracellular matrix protein | −0.262 | 6.180 | 6.441 | 12.895 | 2.663 | 0.120 | 0.025 |  |
| 8013450 | LGALS9B | lectin, galactoside-binding, soluble, 9B | −82.641 | 31.958 | 114.600 | 15.751 | 3.546 | 0.120 | 0.024 |  |
| 7943176 | MED17 | mediator complex subunit 17 | 23.512 | −0.589 | −24.101 | −14.739 | −8.554 | 0.120 | 0.025 |  |
| 8098740 | DUX4 | double homeobox 4 | −13.873 | 2.256 | 16.130 | 16.512 | 7.048 | 0.120 | 0.025 |  |
| 8051030 | SLC5A6 | solute carrier family 5 (sodium/multivitamin and iodide cotransporter), member 6 | −12.863 | 0.444 | 13.307 | 11.476 | 6.877 | 0.121 | 0.025 |  |
| 7921552 | DCAF8 | DDB1 and CUL4 associated factor 8 | −23.580 | 4.520 | 28.100 | 13.910 | −0.911 | 0.121 | 0.025 |  |
| 8031489 | ZNF628 | zinc finger protein 628 | −11.995 | 2.278 | 14.273 | 14.654 | 3.370 | 0.121 | 0.025 |  |
| 8063526 | C20orf106 | chromosome 20 open reading frame 106 | 9.015 | −3.449 | −12.465 | −18.070 | −7.706 | 0.121 | 0.025 |  |
| 7950731 | PRCP | prolylcarboxypeptidase (angiotensinase C) | 53.968 | −13.960 | −67.928 | −11.377 | 1.962 | 0.121 | 0.024 |  |
| 8145829 | ZNF703 | zinc finger protein 703 | −14.399 | 0.397 | 14.796 | 13.703 | 1.538 | 0.121 | 0.025 |  |
| 7943314 | JRKL | JRK-like | 10.075 | 0.457 | −9.617 | −16.500 | −7.335 | 0.122 | 0.025 |  |
| 7950140 | LOC220077 | dedicator of cytokinesis 1 pseudogene | −5.201 | 3.030 | 8.230 | 13.005 | 8.863 | 0.122 | 0.025 |  |
| 8129418 | PTPRK | protein tyrosine phosphatase, receptor type, K | 4.935 | −1.196 | −6.131 | −13.218 | −2.271 | 0.122 | 0.025 |  |
| 7953735 | LOC349196 | hypothetical LOC349196 | 13.528 | −21.647 | −35.175 | −22.353 | 5.162 | 0.122 | 0.025 |  |
| 8104122 | DUX4 | double homeobox 4 | −11.582 | 1.255 | 12.837 | 15.369 | 4.689 | 0.122 | 0.025 |  |
| 8022022 | LPIN2 | lipin 2 | −66.044 | 35.157 | 101.201 | 14.372 | 4.587 | 0.122 | 0.024 |  |
| 7993296 | SHISA9 | shisa family member 9 | −5.629 | 5.862 | 11.491 | 18.505 | 8.384 | 0.122 | 0.025 |  |
| 7976333 | C14orf109 | chromosome 14 open reading frame 109 | 20.868 | −16.841 | −37.709 | −18.228 | −7.765 | 0.123 | 0.025 |  |
| 7954077 | KIAA1467 | family with sequence similarity 234, member B | −10.988 | 5.374 | 16.363 | 16.906 | −1.127 | 0.123 | 0.025 |  |
| 8009476 | MAP2K6 | mitogen-activated protein kinase kinase 6 | 6.656 | −12.705 | −19.361 | −13.968 | −2.186 | 0.123 | 0.025 |  |
| 7973902 | SRP54 | signal recognition particle 54kDa | 42.636 | 2.422 | −40.214 | −11.225 | −6.084 | 0.123 | 0.024 |  |
| 8041273 | YIPF4 | Yip 1 domain family, member 4 | 35.496 | −1.350 | −36.845 | −15.742 | −8.583 | 0.123 | 0.025 |  |
| 8072941 | GALR3 | galanin receptor 3 | −13.948 | 8.014 | 21.962 | 23.343 | 9.976 | 0.123 | 0.025 |  |
| 8155525 | LOC100132167 | uncharacterized LOC100132167 | 4.171 | −4.656 | −8.827 | −14.036 | −1.440 | 0.124 | 0.025 |  |
| 8035156 | CHERP | calcium homeostasis endoplasmic reticulum protein | −40.527 | −2.813 | 37.713 | 10.273 | 1.655 | 0.124 | 0.024 |  |
| 8111814 | RPL37 | ribosomal protein L37 | 31.692 | −5.337 | −37.029 | −13.504 | −3.179 | 0.124 | 0.024 |  |
| 8147424 | C8orf38 | chromosome 8 open reading frame 38 | 8.602 | −1.343 | −9.945 | −14.149 | −5.822 | 0.124 | 0.025 |  |
| 8098732 | DUX4L4 | double homeobox 4 like 4 | −13.415 | 2.322 | 15.738 | 16.352 | 7.875 | 0.124 | 0.025 |  |
| 8047086 | NAB1 | NGFI-A binding protein 1 (EGR1 binding protein 1) | 18.658 | −0.928 | −19.586 | −16.949 | −8.868 | 0.125 | 0.025 |  |
| 8029728 | GIPR | gastric inhibitory polypeptide receptor | −6.037 | 3.932 | 9.969 | 12.977 | 9.142 | 0.125 | 0.025 |  |
| 8011270 | LOC284009 | uncharacterized LOC284009 | −8.778 | 0.994 | 9.772 | 11.850 | −3.344 | 0.125 | 0.025 |  |
| 8071597 | IGLL5 | immunoglobulin lambda-like polypeptide 5 | 27.645 | −10.850 | −38.494 | −32.965 | 2.552 | 0.125 | 0.025 |  |
| 7988970 | KIAA1370 | family with sequence similarity 214, member A | 45.575 | 0.054 | −45.521 | −10.930 | −6.055 | 0.125 | 0.024 |  |
| 7990540 | IMP3 | IMP3, U3 small nucleolar ribonucleoprotein | 7.266 | −9.167 | −16.432 | −13.549 | −3.163 | 0.125 | 0.025 |  |
| 8109732 | MAT2B | methionine adenosyltransferase II, beta | 44.018 | 6.030 | −37.988 | −12.230 | −9.922 | 0.126 | 0.024 |  |
| 8164580 | PTGES | prostaglandin E synthase | −3.335 | 7.221 | 10.556 | 14.586 | 3.267 | 0.126 | 0.025 |  |
| 8067242 | ANKRD60 | ankyrin repeat domain 60 | −7.461 | 1.544 | 9.005 | 16.589 | 8.406 | 0.126 | 0.025 |  |
| 8127370 | KHDRBS2 | KH domain containing, RNA binding, signal transduction associated 2 | 7.332 | −3.163 | −10.496 | −19.099 | −2.889 | 0.126 | 0.025 |  |
| 8172493 | KCND1 | potassium channel, voltage gated shal-related subfamily D, member 1 | −10.066 | −2.348 | 7.718 | 12.365 | 6.525 | 0.126 | 0.025 |  |
| 8155327 | ALDH1B1 | aldehyde dehydrogenase 1 family, member B1 | −2.663 | 8.603 | 11.266 | 12.885 | 5.144 | 0.127 | 0.025 |  |
| 7968898 | KIAA1704 | GPALPP motifs containing 1 | 31.757 | 2.168 | −29.589 | −18.640 | −6.358 | 0.127 | 0.025 |  |
| 8004144 | MIS12 | MIS12 kinetochore complex component | 19.879 | −2.038 | −21.917 | −16.181 | −5.634 | 0.127 | 0.025 |  |
| 7965200 | CCDC59 | coiled-coil domain containing 59 | 34.291 | 6.547 | −27.744 | −22.280 | −4.757 | 0.127 | 0.025 |  |
| 8135064 | TRIM56 | tripartite motif-containing 56 | −17.502 | 11.884 | 29.386 | 13.671 | 2.200 | 0.128 | 0.025 |  |
| 8043438 | IGKV1-5 | immunoglobulin kappa variable 1-5 | 352.405 | −95.319 | −447.724 | −67.337 | 9.344 | 0.128 | 0.023 |  |
| 8007414 | AOC2 | amine oxidase, copper containing 2 (retina-specific) | −23.953 | −6.722 | 17.231 | 12.548 | 2.702 | 0.129 | 0.025 |  |
| 8114138 | C5orf15 | chromosome 5 open reading frame 15 | 14.396 | −8.014 | −22.411 | −12.127 | 0.889 | 0.129 | 0.025 |  |
| 7908907 | ADORA1 | adenosine A1 receptor | −4.277 | 3.663 | 7.941 | 12.488 | 6.155 | 0.129 | 0.025 |  |
| 7925611 | TFB2M | transcription factor B2, mitochondrial | 18.949 | −3.177 | −22.126 | −16.508 | 2.470 | 0.129 | 0.025 |  |
| 8148572 | LY6E | lymphocyte antigen 6 complex, locus E | −84.380 | 54.651 | 139.031 | 26.501 | 6.743 | 0.129 | 0.024 |  |
| 8039139 | LENG1 | leukocyte receptor cluster (LRC) member 1 | 3.404 | −10.746 | −14.150 | −14.924 | −3.679 | 0.130 | 0.025 |  |
| 8114653 | TAF7 | TAF7 RNA polymerase II, TATA box binding protein (TBP)-associated factor, 55kDa | 26.729 | −1.371 | −28.100 | −17.939 | −6.385 | 0.130 | 0.025 |  |
| 8021453 | SEC11C | SEC11 homolog C, singal peptidase complex subunit | 17.258 | −0.772 | −18.030 | −23.712 | −8.699 | 0.130 | 0.025 |  |
| 7931571 | PRAP1 | proline-rich acidic protei 1 | −9.703 | 4.028 | 13.732 | 16.572 | 9.935 | 0.130 | 0.025 |  |
| 8158224 | SLC27A4 | solute carrier family 27(fatty acid transporter), member 4 | −11.197 | 2.787 | 13.983 | 10.258 | 4.549 | 0.130 | 0.025 |  |
| 7992744 | FLYWCH1 | FLYWCH-type zinc finger 1 | −9.871 | 7.728 | 17.599 | 13.018 | 6.174 | 0.130 | 0.025 |  |
| 8050591 | NDUFAF2 | NADH dehydrogenase (ubiquinone) complex 1, assembly factor 2 | 4.189 | −3.825 | −8.013 | −13.563 | −0.519 | 0.131 | 0.025 |  |
| 8027473 | PDCD5 | Programmed cell death 5 | 5.762 | −6.323 | −12.085 | −11.127 | 0.122 | 0.131 | 0.025 |  |
| 8061019 | C20orf7 | chromosome 20 open reading frame 7 | 7.581 | 1.839 | −5.742 | −11.088 | 0.396 | 0.131 | 0.025 |  |
| 8130720 | SFT2D1 | SFT2 domain containing 1 | 19.143 | −4.461 | −23.604 | −10.215 | 0.642 | 0.131 | 0.025 |  |
| 7939477 | ALKBH3 | AlkB homolog 3, alpha-ketoglutarate-dependent dioxygenase | 23.065 | −0.140 | −23.204 | −13.098 | −0.885 | 0.132 | 0.025 |  |
| 8165131 | SNAPC4 | small nuclear RNA activating complex, polypeptide 4, 190kDa | −6.327 | 3.838 | 10.165 | 14.832 | 9.250 | 0.132 | 0.025 |  |
| 7945101 | DCPS | decapping enzyme, scavenger | 6.722 | −11.340 | −18.061 | −15.447 | −0.560 | 0.132 | 0.025 |  |
| 8066260 | SNORA71C | small nucleolar RNA, H/ACA box 71C | 18.252 | −49.228 | −67.480 | −13.999 | −7.890 | 0.133 | 0.024 |  |
| 8125859 | TAF11 | TAF11 RNApolymerase II, TATA box binding protein (TBP)-associated factor, 28kDa | 6.376 | −3.804 | −10.180 | −12.392 | −7.961 | 0.133 | 0.025 |  |
| 8067680 | PRIC285 | helicase with zinc finger 2, transcriptional coactivator | −22.918 | 3.772 | 26.690 | 20.812 | 9.189 | 0.133 | 0.025 |  |
| 8136954 | FAM115C | TRPM8 channel-associated factor 2 | −20.273 | 6.512 | 26.785 | 13.903 | 9.390 | 0.133 | 0.025 |  |
| 8153342 | LYPD2 | LY6/PLAUR domain containing 2 | −12.783 | 2.587 | 15.370 | 12.852 | −1.565 | 0.134 | 0.025 |  |
| 8013500 | TMEM11 | transmembrane protein 11 | 7.508 | −6.831 | −14.338 | −14.220 | −7.839 | 0.134 | 0.025 |  |
| 7916282 | LRP8 | low density lipoprotein receptor-related protein 8, apolipoprotein E receptor | 10.361 | −9.587 | −19.947 | −11.732 | 1.653 | 0.134 | 0.025 |  |
| 8117408 | HIST1H2AE | histone cluster 1, H2ae | 7.743 | 0.349 | −7.394 | −11.478 | −7.317 | 0.134 | 0.025 |  |
| 7970194 | MCF2L | MCF.2 cell line derived transforming sequence-like | −4.010 | 2.564 | 6.574 | 11.046 | 8.878 | 0.134 | 0.025 |  |
| 8119080 | SRSF3 | serine/arginine-rich splicing factor 3 | 83.010 | −15.105 | −98.116 | −12.213 | −1.158 | 0.134 | 0.024 |  |
| 8164067 | PSMB7 | proteasome subunit beta 7 | 35.737 | −13.990 | −49.727 | −10.543 | −2.318 | 0.136 | 0.024 |  |
| 8070655 | U2AF1 | U2 small nuclear RNA auxiliary factor 1 | 30.940 | −75.724 | −106.664 | −10.630 | 0.045 | 0.136 | 0.023 |  |
| 8084092 | NDUFB5 | NADH dehydrogenase (ubiquinone)1 beta subcomplex, 5, 16kDa | 36.345 | −1.398 | −37.743 | −10.597 | −2.969 | 0.136 | 0.024 |  |
| 8035311 | TMEM221 | transmembrane protein 221 | −9.030 | 2.495 | 11.525 | 14.156 | 7.185 | 0.136 | 0.025 |  |
| 7949615 | B3GNT1 | UDP-GlcNAc:betaGal beta-1, 3-*N*-acetylglucosaminyltransferase 1 | 5.656 | 0.934 | −4.722 | −10.087 | −1.520 | 0.137 | 0.025 |  |
| 8033385 | PEX11G | peroxisomal biogenesis factor 11 gamma | −4.417 | 3.980 | 8.396 | 10.464 | 8.826 | 0.137 | 0.025 |  |
| 7981278 | DEGS2 | delta(4)-desaturase, sphingolipid 2 | −5.307 | 7.156 | 12.463 | 11.458 | 9.044 | 0.137 | 0.025 |  |
| 8092230 | ZMAT3 | zinc finger, matrin-type 3 | −8.153 | 25.511 | 33.663 | 14.031 | 4.424 | 0.137 | 0.025 |  |
| 8099897 | UGDH | UDP-glucose6-dehydrogenase | 16.946 | −1.812 | −18.758 | −11.265 | 0.542 | 0.138 | 0.025 |  |
| 8003193 | FAM92B | family with sequence similarity 92, member B | −6.748 | 2.683 | 9.431 | 11.881 | 9.235 | 0.138 | 0.025 |  |
| 8016088 | CCDC43 | coiled-coil domain containing 43 | 13.497 | −4.158 | −17.654 | −15.530 | −9.196 | 0.139 | 0.025 |  |
| 8035566 | HOMER3 | homer scaffolding protein 3 | −11.024 | 2.688 | 13.713 | 10.510 | 4.707 | 0.140 | 0.025 |  |
| 7986789 | ATP10A | ATPase, class V, type 10A | −15.032 | 2.510 | 17.543 | 12.147 | 7.203 | 0.140 | 0.025 |  |
| 7987554 | DNAJC17 | DnaJ (Hsp40) homolog, subfamily C, member 17 | −0.575 | −11.073 | −10.497 | −10.399 | 7.767 | 0.140 | 0.025 |  |
| 7897441 | H6PD | hexose-6-phosphate dehydrogenase (glucose1-dehydrogenase) | −34.681 | 1.993 | 36.674 | 16.630 | 3.195 | 0.140 | 0.025 |  |
| 8050507 | WDR35 | WD repeat domain 35 | 7.827 | −4.259 | −12.086 | −22.784 | −6.787 | 0.140 | 0.025 |  |
| 8005267 | RAI1 | retinoic acid induced 1 | −17.852 | −7.108 | 10.744 | 10.663 | 3.220 | 0.141 | 0.025 |  |
| 7950195 | MIR139 | microRNA 139 | −6.899 | 10.171 | 17.070 | 18.991 | 3.383 | 0.141 | 0.025 |  |
| 8151296 | LACTB2 | lactamase, beta 2 | 9.411 | −0.569 | −9.980 | −18.258 | −9.478 | 0.141 | 0.025 |  |
| 8064939 | TMX4 | thioredoxin-related transmembrane protein 4 | 46.056 | −6.732 | −52.789 | −11.156 | −7.184 | 0.141 | 0.024 |  |
| 7996725 | DUS2L | dihydrouridine synthase 2 | 23.261 | −11.380 | −34.641 | −11.228 | −1.262 | 0.141 | 0.024 |  |
| 8079167 | C3orf77 | chromosome 3 open reading frame 77 | −3.904 | 6.118 | 10.022 | 15.066 | 7.105 | 0.142 | 0.025 |  |
| 8100827 | IGJ | joining chain of multimeric IgA and IgM | 322.871 | −73.308 | −396.179 | −144.088 | −1.594 | 0.142 | 0.024 |  |
| 8122440 | LTV1 | LTV1 ribsome biogenesis factor | 15.562 | −3.338 | −18.900 | −14.339 | −1.863 | 0.142 | 0.025 |  |
| 7905968 | MTX1 | metaxin 1 | 13.406 | −3.028 | −16.434 | −13.070 | −6.274 | 0.142 | 0.025 |  |
| 8049670 | GPC1 | glypican 1 | −7.989 | 2.323 | 10.313 | 13.814 | 9.279 | 0.142 | 0.025 |  |
| 8033420 | FCER2 | Fc fragment of IgE, low affinity II, receptor for (CD23) | 35.390 | −14.638 | −50.027 | −16.141 | 4.846 | 0.142 | 0.024 |  |
| 7995926 | NLRC5 | NLR family, CARD domain containing 5 | −72.682 | 3.572 | 76.254 | 11.952 | 6.045 | 0.142 | 0.024 |  |
| 8114612 | CD14 | CD14 molecule | 64.720 | –271.183 | −335.903 | −12.013 | 5.575 | 0.143 | 0.020 |  |
| 7945663 | LOC402778 | CD225 family protein FLJ76511 | −1.956 | −12.461 | −10.505 | −17.402 | −0.867 | 0.144 | 0.025 |  |
| 8073949 | CRELD2 | cysteine-rich with EGF-like domains 2 | 14.060 | −0.256 | −14.316 | −17.694 | −6.864 | 0.144 | 0.025 |  |
| 7990273 | STOML1 | Stomatin (EPB72)-like 1 | 2.933 | −5.090 | −8.022 | −13.558 | −1.540 | 0.146 | 0.025 |  |
| 8006119 | MIR423 | microRNA 423 | 9.107 | −2.163 | −11.270 | −12.854 | −6.450 | 0.146 | 0.025 |  |
| 7914630 | TRIM62 | tripartite motif-containing 62 | −18.096 | 0.659 | 18.755 | 10.158 | 7.062 | 0.146 | 0.025 |  |
| 8131253 | FOXK1 | forkhead box K1 | −36.882 | −8.594 | 28.288 | 10.238 | 4.950 | 0.146 | 0.025 |  |
| 8157463 | C9orf91 | chromosome 9 open reading frame 91 | −28.853 | −0.868 | 27.985 | 12.039 | 3.536 | 0.147 | 0.025 |  |
| 7978838 | C14orf104 | chromosome 14 open reading frame 104 | 10.064 | −2.546 | −12.610 | −17.598 | −2.943 | 0.147 | 0.025 |  |
| 8152845 | FAM49B | family with sequence similarity 49, member B | 100.461 | 0.070 | −100.390 | −11.226 | −8.382 | 0.148 | 0.023 |  |
| 7919314 | FMO5 | flavin containing monooxygenase 5 | 6.417 | −5.706 | −12.123 | −15.464 | −9.562 | 0.148 | 0.025 |  |
| 8010673 | SLC25A10 | solute carrier family 25(mitochondrial carrier; dicarboxylate transporter), member 10 | −6.591 | 3.526 | 10.117 | 16.597 | 9.419 | 0.148 | 0.025 |  |
| 8155062 | TESK1 | testis-specific kinase 1 | −20.869 | 11.871 | 32.740 | 10.914 | 3.411 | 0.148 | 0.024 |  |
| 7900999 | C1orf228 | chromosome 1 open reading frame 228 | 10.472 | −0.134 | −10.605 | −12.777 | −1.535 | 0.149 | 0.025 |  |
| 8089743 | B4GALT4 | UDP-Gal:betaGlcNAc beta 1,4-galactosyltransferase, polypeptide 4 | −2.669 | 10.698 | 13.367 | 14.893 | 6.464 | 0.149 | 0.025 |  |
| 8038556 | NAPSA | napsin A aspartic peptidase | 1.304 | −5.801 | −7.105 | −13.114 | 0.266 | 0.149 | 0.025 |  |
| 8048460 | CDK5R2 | cyclin-dependent kinase 5, regulatory subunit 2 (p39) | −6.760 | 3.363 | 10.124 | 14.195 | 6.839 | 0.149 | 0.025 |  |
| 8096771 | OSTC | oligosaccharyltransferase complex subunit (non-catalytic) | 61.003 | −10.129 | −71.132 | −16.820 | −0.150 | 0.149 | 0.024 |  |
| 8073022 | CBY1 | chibby homolog 1(Drosophila) | 10.940 | −8.742 | −19.682 | −23.310 | −7.256 | 0.149 | 0.025 |  |
| 7938592 | FAR1 | fatty acyl CoA reductase 1 | 29.850 | −8.718 | −38.567 | −11.704 | −9.274 | 0.149 | 0.024 |  |
| 7992414 | TBL3 | transducin (beta)-like 3 | −15.679 | −0.711 | 14.968 | 12.335 | 3.549 | 0.150 | 0.025 |  |
| 7974447 | SOCS4 | suppressor of cytokine signaling 4 | 19.503 | 0.737 | −18.766 | −13.475 | −2.150 | 0.150 | 0.025 |  |
| 7964745 | TMBIM4 | transmembrane BAX inhibitor motif containing 4 | 55.551 | −14.792 | −70.343 | −10.429 | −6.326 | 0.150 | 0.024 |  |
| 8004741 | CNTROB | centrobin, centrosomal BRCA2 interacting protein | −15.684 | −4.406 | 11.278 | 13.123 | 3.395 | 0.150 | 0.025 |  |
| 7958275 | POLR3B | polymerase (RNA) III (DNA directed) polypeptide B | 8.369 | −5.088 | −13.456 | −10.895 | −9.518 | 0.151 | 0.025 |  |
| 8173366 | P2RY4 | pyrimidinergic receptor P2Y, G-protein coupled, 4 | −4.317 | 1.609 | 5.926 | 11.069 | 5.181 | 0.151 | 0.025 |  |
| 7945740 | RPL26L1 | ribosomal protein L26-like 1 | 6.578 | −3.271 | −9.849 | −12.252 | −7.167 | 0.151 | 0.025 |  |
| 8143028 | CHCHD3 | coiled-coil-helix-coiled-coil-helix domain containing 3 | 48.116 | 3.082 | −45.034 | −16.173 | −1.351 | 0.151 | 0.024 |  |
| 8039013 | ZNF321 | zinc finger protein 321 | 14.632 | −3.544 | −18.176 | −20.708 | −9.580 | 0.151 | 0.025 |  |
| 8050278 | PDIA6 | protein disulfide isomerase family A, member 6 | 72.894 | −2.393 | −75.288 | −14.150 | −7.252 | 0.151 | 0.024 |  |
| 8150175 | C8orf41 | chromosome 8 open reading frame 41 | 10.714 | −4.278 | −14.992 | −13.548 | −6.742 | 0.152 | 0.025 |  |
| 7899604 | ZCCHC17 | zinc finger, CCHC domain containing 17 | 22.950 | 1.952 | −20.998 | −14.906 | −1.975 | 0.152 | 0.025 |  |
| 8027213 | YJEFN3 | YjeF *N*-terminal domain containing 3 | −7.224 | 4.443 | 11.667 | 10.116 | 5.026 | 0.152 | 0.025 |  |
| 8054192 | MITD1 | MIT, microtubule interacting and transport, domain containing 1 | 9.848 | −1.050 | −10.897 | −20.468 | −9.900 | 0.152 | 0.025 |  |
| 7916393 | CYB5RL | cytochrome B5 reductase-like | −1.392 | 7.002 | 8.394 | 12.933 | 5.096 | 0.153 | 0.025 |  |
| 7928107 | H2AFY2 | H2A histone family, member Y2 | 5.675 | −4.304 | −9.979 | −17.156 | −6.164 | 0.153 | 0.025 |  |
| 8053984 | ANKRD23 | ankyrin repeat domain 23 | 1.652 | −9.392 | −11.044 | −10.926 | −2.907 | 0.153 | 0.025 |  |
| 7906140 | BGLAP | bone gamma-carboxyglutamate (gla) protein | −6.531 | 4.697 | 11.228 | 10.173 | 2.358 | 0.153 | 0.025 |  |
| 8117422 | HIST1H4F | histone cluster 1, H4f | 26.092 | 5.284 | −20.807 | −14.660 | −9.031 | 0.153 | 0.025 |  |
| 8122982 | ZDHHC14 | zinc finger, DHHC-type containing 14 | −10.850 | 8.432 | 19.282 | 26.382 | 4.584 | 0.153 | 0.025 |  |
| 8061373 | GZF1 | GDNF-inducible zinc finger protein 1 | 0.477 | −6.510 | −6.987 | −10.406 | −2.642 | 0.154 | 0.025 |  |
| 7901895 | ATG4C | autophagy related 4C, cysteine peptidase | 19.710 | 0.457 | −19.253 | −10.072 | 0.345 | 0.155 | 0.025 |  |
| 7993467 | NDE1 | NudE neurodevelopment protein 1 | 64.974 | −30.996 | −95.970 | −10.382 | −7.033 | 0.155 | 0.023 |  |
| 8138547 | TOMM7 | translocase of outer mitochondrial membrane 7 homolog (yeast) | −2.523 | 7.567 | 10.091 | 15.289 | 1.101 | 0.155 | 0.025 |  |
| 7986160 | UNC45A | Unc-45 myosin chaperone A | −10.111 | 8.895 | 19.006 | 13.480 | −1.711 | 0.155 | 0.025 |  |
| 8109901 | FOXI1 | fork headbox I1 | −6.485 | 4.212 | 10.697 | 18.907 | 6.742 | 0.156 | 0.025 |  |
| 7951966 | FXYD2 | FXYD domain containing ion transport regulator2 | 5.263 | −8.139 | −13.401 | −15.966 | −9.293 | 0.157 | 0.025 |  |
| 7910099 | SRP9 | signal recognition particle 9kDa | 42.516 | 0.986 | −41.530 | −12.237 | −6.532 | 0.157 | 0.024 |  |
| 7977693 | CHD8 | chromodomain helicase DNA binding protein 8 | −30.373 | 7.410 | 37.782 | 12.383 | −1.411 | 0.157 | 0.024 |  |
| 7939587 | DKFZp779M065 | hypothetical DKFZp779M0652 | −11.623 | 3.640 | 15.264 | 16.464 | 0.782 | 0.157 | 0.025 |  |
| 8033097 | RFX2 | regulatory factor X, 2 (influences HLA class II expression) | 18.405 | −29.330 | −47.735 | −17.448 | −9.731 | 0.158 | 0.024 |  |
| 8092241 | KCNMB3 | potassium channel subfamily M regulatory beta subunit 3 | −2.189 | 5.409 | 7.597 | 13.128 | 9.009 | 0.158 | 0.025 |  |
| 8139456 | SNORA9 | small nucleolar RNA, H/ACA box 9 | −25.320 | −5.500 | 19.820 | 17.936 | 3.566 | 0.158 | 0.025 |  |
| 7898227 | PLEKHM2 | pleckstrin homology domain containing, family M (with RUN domain) member 2 | −40.991 | 6.802 | 47.793 | 10.254 | 3.001 | 0.158 | 0.024 |  |
| 7977454 | POTEM | POTE ankyrin domain family, member M | –114.650 | −11.767 | 102.882 | 10.610 | 5.775 | 0.160 | 0.023 |  |
| 8063345 | SNORD12C | small nucleolar RNA, C/D box 12C | 36.648 | −16.930 | −53.578 | −15.688 | 5.227 | 0.160 | 0.024 |  |
| 7981722 | IGHA1 | immunoglobulin heavy constant alpha 1 | 414.655 | –131.637 | −546.292 | −21.936 | 9.700 | 0.160 | 0.019 |  |
| 7931623 | LOC619207 | scavenger receptor protein family member | −5.547 | 5.970 | 11.517 | 15.564 | 8.345 | 0.160 | 0.025 |  |
| 8129193 | C6orf204 | chromosome 6 open reading frame 204 | 17.399 | −7.775 | −25.174 | −10.869 | −9.430 | 0.161 | 0.025 |  |
| 7981724 | IGHD | immunoglobulin heavy constant delta | 635.087 | –203.976 | −839.063 | −54.281 | 5.856 | 0.161 | 0.021 |  |
| 8140113 | STX1A | syntaxin 1A (brain) | −6.464 | 0.856 | 7.321 | 10.552 | 1.690 | 0.161 | 0.025 |  |
| 8063427 | MOCS3 | molybdenum cofactor synthesis 3 | 3.937 | −8.235 | −12.172 | −13.957 | −2.803 | 0.161 | 0.025 |  |
| 8116494 | ZFP62 | ZFP62 zinc finger protein | 21.982 | −1.886 | −23.868 | −17.157 | −5.644 | 0.161 | 0.025 |  |
| 8153346 | LYNX1 | Ly6/neurotoxin 1 | −3.996 | 3.044 | 7.040 | 10.455 | −0.517 | 0.161 | 0.025 |  |
| 7909815 | IARS2 | isoleucyl-tRNA synthetase 2, mitochondrial | −8.521 | 9.152 | 17.673 | 10.552 | 7.786 | 0.162 | 0.025 |  |
| 7941714 | C11orf80 | chromosome 11 open reading frame 80 | 15.451 | −0.393 | −15.844 | −14.027 | −8.964 | 0.162 | 0.025 |  |
| 8022914 | RPRD1A | regulation of nuclear pre-mRNA domain containing 1A | 19.145 | −10.166 | −29.311 | −11.422 | −8.495 | 0.162 | 0.025 |  |
| 8043465 | IGKC | immunoglobulin kappa constant | 298.090 | −86.000 | −384.090 | −227.964 | 2.395 | 0.162 | 0.025 |  |
| 7915156 | POU3F1 | POU class 3 homeobox 1 | −9.324 | 3.336 | 12.660 | 13.674 | 9.040 | 0.163 | 0.025 |  |
| 7993248 | C16orf75 | chromosome 16 open reading frame 75 | −4.843 | 3.713 | 8.556 | 15.652 | 1.091 | 0.163 | 0.025 |  |
| 7951873 | SIK3 | SIK family kinase 3 | –117.965 | −5.172 | 112.794 | 12.347 | −4.886 | 0.163 | 0.023 |  |
| 8070933 | FTCD | formiminotransferase cyclodeaminase | −7.361 | −0.765 | 6.596 | 12.497 | 5.365 | 0.163 | 0.025 |  |
| 8001046 | ZNF843 | zinc finger protein 843 | −4.914 | 0.533 | 5.448 | 10.031 | 7.229 | 0.163 | 0.025 |  |
| 8141421 | MIR93 | microRNA 93 | −13.675 | −3.245 | 10.430 | 19.898 | 8.700 | 0.163 | 0.025 |  |
| 7975113 | CHURC1 | churchill domain containing 1 | 67.717 | 14.361 | −53.356 | −15.579 | 1.885 | 0.164 | 0.024 |  |
| 8098576 | SLC25A4 | solute carrier family 25 (mitochondrial carrier;adenine nucleotide translocator), member 4 | 14.310 | −0.024 | −14.334 | −19.695 | −1.470 | 0.164 | 0.025 |  |
| 7905233 | ADAMTSL4 | ADAMTS-like 4 | −76.401 | −19.897 | 56.504 | 50.748 | −3.739 | 0.164 | 0.025 |  |
| 8031815 | ZNF776 | zinc finger protein 776 | 32.318 | −5.697 | −38.016 | −13.900 | −6.625 | 0.164 | 0.024 |  |
| 7919208 | GNRHR2 | gonadotropin-releasing hormone (type 2) receptor 2 | −4.690 | 3.983 | 8.673 | 12.967 | 4.674 | 0.165 | 0.025 |  |
| 7918902 | CD58 | CD58 molecule | 43.318 | −10.900 | −54.219 | −11.057 | −6.160 | 0.165 | 0.024 |  |
| 8056766 | SLC25A12 | solute carrier family 25 (aspartate/glutamate carrier), member 12 | 29.757 | −5.963 | −35.720 | −16.340 | −8.109 | 0.165 | 0.025 |  |
| 8030980 | ZNF525 | zinc finger protei 525 | 224.290 | −17.285 | −241.575 | −15.617 | −7.473 | 0.165 | 0.022 |  |
| 7945648 | KRTAP5-2 | keratin associated protein 5-2 | −3.443 | 12.704 | 16.147 | 20.118 | 8.787 | 0.166 | 0.025 |  |
| 8039144 | TMC4 | transmembrane channel-like 4 | −4.713 | 4.677 | 9.390 | 12.630 | 6.357 | 0.167 | 0.025 |  |
| 8046276 | SP5 | Sp5 transcription factor | −9.850 | 3.094 | 12.945 | 17.598 | 5.835 | 0.167 | 0.025 |  |
| 7949340 | BATF2 | basicl eucine zipper transcription factor, ATF-like 2 | −22.033 | 14.206 | 36.240 | 33.802 | 2.918 | 0.167 | 0.025 |  |
| 7994961 | CTF1 | cardiotrophin 1 | 3.360 | −7.755 | −11.114 | −13.796 | −7.851 | 0.168 | 0.025 |  |
| 7986561 | VSIG7 | immuglobulin heavy variable 1/OR15-9 (non-functional) | 8.888 | −7.496 | −16.384 | −21.516 | 1.121 | 0.168 | 0.025 |  |
| 8165217 | NOTCH1 | motch1 | –129.020 | −41.862 | 87.158 | 10.701 | 4.234 | 0.168 | 0.024 |  |
| 7987675 | RPAP1 | RNA polymerase II associated protein 1 | −19.882 | −3.229 | 16.653 | 11.394 | 1.885 | 0.169 | 0.025 |  |
| 8153709 | FBXL6 | F-box and leucine-rich repeat protein 6 | −7.675 | −0.435 | 7.240 | 10.652 | 8.005 | 0.169 | 0.025 |  |
| 8138922 | KBTBD2 | kelch repeat and BTB (POZ) domain containing 2 | 45.227 | −0.877 | −46.104 | −10.549 | −9.593 | 0.169 | 0.024 |  |
| 8125483 | TAP2 | transporter 2, ATP-binding cassette, sub-family B (MDR/TAP) | −57.632 | −11.519 | 46.113 | 10.427 | 1.033 | 0.169 | 0.024 |  |
| 8113071 | LOC729040 | uncharaterized LOC729040 | −25.214 | −0.394 | 24.820 | 11.972 | −6.059 | 0.169 | 0.025 |  |
| 8078918 | SNORA62 | small nucleolar RNA, H/ACA box 62 | −19.695 | 15.815 | 35.510 | 26.828 | 5.180 | 0.169 | 0.025 |  |
| 8039440 | HSPBP1 | HSPA(heat shock 70kDa) binding protein, cytoplasmic cochaperone 1 | 5.507 | −3.541 | −9.048 | −10.643 | −2.658 | 0.169 | 0.025 |  |
| 8037657 | DMPK | dystrophia myotonica- protein kinase | −6.468 | 5.056 | 11.524 | 11.217 | 6.579 | 0.170 | 0.025 |  |
| 7981730 | IGLJ3 | immunoglobulin lambda joining 3 | 928.423 | –236.752 | −1165.175 | −50.961 | 7.401 | 0.170 | 0.020 |  |
| 8088020 | ITIH4 | inter-alpha trypsin inhibitor heavy chain family, member 4 | −17.273 | −4.285 | 12.987 | 11.493 | 8.658 | 0.170 | 0.025 |  |
| 7955290 | AQP2 | aquaporin 2 (collecting duct) | −8.240 | −2.252 | 5.989 | 10.849 | 7.991 | 0.170 | 0.025 |  |
| 7916568 | DAB1 | Dab, reelin singal transducer, homolog 1(Drosophila) | −12.848 | −2.530 | 10.318 | 12.840 | 2.446 | 0.170 | 0.025 |  |
| 7977440 | KIAA0125 | KIAA0125 | 24.827 | −6.717 | −31.543 | −24.930 | 7.676 | 0.170 | 0.025 |  |
| 8029368 | ZNF284 | zinc finger protein 284 | 19.584 | 0.343 | −19.241 | −19.732 | −5.919 | 0.171 | 0.025 |  |
| 8121144 | MANEA | mannosidase, endo-alpha | 38.716 | −2.032 | −40.748 | −21.724 | 0.958 | 0.171 | 0.025 |  |
| 8129773 | BCLAF1 | BCL2-associated transcription factor 1 | 82.473 | 1.283 | −81.190 | −12.092 | −3.383 | 0.171 | 0.024 |  |
| 8178988 | ZBTB22 | zinc finger and BTB domain containing 22 | −19.627 | −5.678 | 13.950 | 10.175 | 4.009 | 0.172 | 0.025 |  |
| 7977294 | ZBTB42 | zinc finger and BTB domain containing 42 | −23.990 | 5.239 | 29.229 | 25.657 | 2.156 | 0.172 | 0.025 |  |
| 8117537 | HIST1H4I | histone cluster 1, H4i | 5.936 | −1.311 | −7.247 | −12.807 | −6.055 | 0.172 | 0.025 |  |
| 8090866 | ANAPC13 | anaphase promoting complex subunit 13 | 11.740 | −10.509 | −22.248 | −10.189 | −2.841 | 0.172 | 0.025 |  |
| 8151890 | TP53INP1 | tumor protein p53 inducible nuclear protein 1 | 93.583 | 5.771 | −87.813 | −10.632 | −1.583 | 0.172 | 0.023 |  |
| 8135480 | DNAJB9 | DnaJ (Hsp40) homolog, subfamily B, member 9 | 20.889 | 3.592 | −17.297 | −17.207 | −7.296 | 0.172 | 0.025 |  |
| 8133049 | ZNF107 | zinc finger protein 107 | 57.870 | 3.838 | −54.032 | −16.252 | 4.042 | 0.173 | 0.024 |  |
| 8021768 | TSHZ1 | teashirt zinc finger homeobox 1 | 16.284 | −5.484 | −21.768 | −16.901 | −6.206 | 0.173 | 0.025 |  |
| 7902789 | HS2ST1 | heparan sulfate 2-O-sulfotransferase 1 | 17.676 | 2.799 | −14.876 | −10.399 | −2.765 | 0.173 | 0.025 |  |
| 8141625 | EPHB4 | EPH receptor B4 | −12.080 | 9.241 | 21.321 | 14.599 | 3.525 | 0.173 | 0.025 |  |
| 7937483 | SNORA52 | small nucleolar RNA, H/ACA box 52 | −38.211 | −5.219 | 32.992 | 19.386 | 1.575 | 0.174 | 0.025 |  |
| 8072796 | TMPRSS6 | transmembrane protease, serine 6 | −6.590 | 0.939 | 7.529 | 13.237 | 5.201 | 0.174 | 0.025 |  |
| 8022640 | DHFR | dihydrofolate reductase | 24.205 | −5.621 | −29.826 | −15.285 | −4.399 | 0.174 | 0.025 |  |
| 7925492 | OPN3 | opsin 3 | 18.795 | −1.783 | −20.579 | −10.757 | 0.065 | 0.174 | 0.025 |  |
| 8081867 | C3orf1 | chromosome 3 open reading frame 1 | 87.434 | 4.462 | −82.972 | −16.018 | −0.559 | 0.175 | 0.024 |  |
| 8162179 | GAS1 | growth arrest-specific 1 | −7.221 | 0.527 | 7.748 | 13.342 | 1.957 | 0.175 | 0.025 |  |
| 8157383 | COL27A1 | collagen, type XXVII, alpha 1 | −3.807 | 2.957 | 6.764 | 12.774 | 5.756 | 0.175 | 0.025 |  |
| 8120967 | NT5E | 5'-nucleotidase, ecto(CD73) | 7.047 | −3.244 | −10.291 | −13.794 | 5.418 | 0.175 | 0.025 |  |
| 8144995 | FGF17 | fibroblast growth factor 17 | 0.624 | 7.965 | 7.340 | 14.566 | 3.706 | 0.175 | 0.025 |  |
| 7954196 | MGST1 | microsomal glutathione S-transferase 1 | 11.302 | −11.684 | −22.986 | −14.533 | −4.676 | 0.176 | 0.025 |  |
| 8073309 | LOC100288034 | hypothetical protein LOC100288034 | −19.968 | −0.472 | 19.496 | 10.595 | 4.647 | 0.176 | 0.025 |  |
| 7899829 | S100PBP | S100P binding protein | 31.633 | −0.457 | −32.090 | −11.004 | −0.048 | 0.176 | 0.024 |  |
| 8026424 | CYP4F22 | cytochrome P450, family 4, subfamily F, polypeptide 22 | −6.438 | 5.124 | 11.562 | 15.673 | 4.679 | 0.176 | 0.025 |  |
| 8103563 | DDX60 | DEAD (Asp-Glu-Ala-Asp) box polypeptide 60 | −43.505 | 43.402 | 86.908 | 35.674 | 4.643 | 0.176 | 0.025 |  |
| 8136045 | IRF5 | interferon regulatory factor 5 | −21.347 | −5.546 | 15.801 | 10.746 | 7.332 | 0.177 | 0.025 |  |
| 8165064 | UBAC1 | UBA domain containing 1 | 22.492 | −18.024 | −40.516 | −16.811 | −7.134 | 0.177 | 0.025 |  |
| 7899528 | OPRD1 | opioid receptor, delta 1 | −12.330 | 4.968 | 17.299 | 20.773 | 5.890 | 0.177 | 0.025 |  |
| 7960117 | PXMP2 | peroxisomal membrane protein 2, 22kDa | 11.119 | −3.132 | −14.251 | −10.322 | −7.266 | 0.177 | 0.025 |  |
| 7961306 | PRB1 | proline-rich protein BstNI subfamily 1 | −5.951 | 2.066 | 8.017 | 14.031 | 8.460 | 0.177 | 0.025 |  |
| 8105878 | RAD17 | RAD17 checkpoint clam loader component | 24.376 | 5.069 | −19.307 | −16.675 | −6.558 | 0.178 | 0.025 |  |
| 8071642 | IGLV6-57 | immunoglobulin lambda variable 6-57 | 23.025 | −5.094 | −28.119 | −46.274 | 0.558 | 0.178 | 0.025 |  |
| 7995787 | MT1M | metallothionein 1M | 1.679 | −6.085 | −7.763 | −14.030 | −5.373 | 0.178 | 0.025 |  |
| 7913787 | C1orf201 | chromosome 1 open reading frame 201 | 5.263 | −0.879 | −6.141 | −12.412 | −4.215 | 0.178 | 0.025 |  |
| 7913682 | HMGCL | 3-hydroxymethyl-3-methylglutaryl-CoA lyase | 10.839 | −2.976 | −13.815 | −11.605 | −7.441 | 0.178 | 0.025 |  |
| 8131944 | NFE2L3 | nuclear factor (erythroid-derived 2)-like 3 | 8.322 | −8.692 | −17.014 | −14.393 | −3.260 | 0.178 | 0.025 |  |
| 7953622 | LOC283314 | hypothetical LOC283314 | −14.391 | −2.665 | 11.726 | 10.181 | 5.831 | 0.179 | 0.025 |  |
| 8063839 | SS18L1 | synovial sarcoma translocation gene on chromosome 18-like 1 | −2.092 | −11.584 | −9.492 | −10.696 | −6.594 | 0.179 | 0.025 |  |
| 8084478 | FAM131A | family with sequence similarity 131, member A | −7.420 | −0.443 | 6.977 | 10.829 | −2.377 | 0.179 | 0.025 |  |
| 7993298 | ERCC4 | excision repair cross-complementation group 4 | −8.634 | 3.627 | 12.261 | 10.365 | −3.937 | 0.179 | 0.025 |  |
| 7965789 | ARL1 | ADP-ribosylation factor-like 1 | 28.312 | 4.061 | −24.250 | −13.444 | −0.512 | 0.179 | 0.025 |  |
| 8098714 | FRG1 | FSHD region gene 1 | 40.453 | −76.733 | −117.186 | −86.515 | −7.551 | 0.180 | 0.025 |  |
| 8119722 | CUL9 | cullin 9 | −19.763 | 3.341 | 23.104 | 14.280 | 6.161 | 0.180 | 0.025 |  |
| 7919589 | HIST2H3D | histone cluster 2, H3d | 29.709 | −7.160 | −36.869 | −13.420 | −3.893 | 0.180 | 0.024 |  |
| 8035813 | ZNF43 | zinc finger protein 43 | 13.140 | −3.980 | −17.121 | −22.407 | −9.709 | 0.180 | 0.025 |  |
| 7954006 | PTMA | prothymosin, alpha | 174.513 | −3.912 | −178.425 | −12.032 | −8.759 | 0.181 | 0.022 |  |
| 8121312 | C6orf203 | chromosome 6 open reading frame 203 | 7.650 | −1.492 | −9.141 | −13.239 | −7.828 | 0.182 | 0.025 |  |
| 8109333 | GPX3 | glutathione peroxidase 3 | −3.299 | 6.496 | 9.795 | 14.053 | 3.545 | 0.182 | 0.025 |  |
| 8103518 | ANP32C | acidic (leucine-rich) nuclear phosphoprotein 32 family, member C | 6.989 | −2.125 | −9.115 | −12.034 | −3.323 | 0.182 | 0.025 |  |
| 7971419 | SIAH3 | Siah E3 ubiquitin protein ligase family member 3 | −3.986 | 1.898 | 5.884 | 11.313 | −0.007 | 0.182 | 0.025 |  |
| 7939818 | PTPMT1 | protein tyrosine phosphatase, mitochondrial 1 | 16.514 | −3.331 | −19.845 | −12.871 | −4.653 | 0.182 | 0.025 |  |
| 8172504 | GRIPAP1 | GRIP1 associated protein 1 | −18.085 | 1.633 | 19.718 | 14.564 | 2.051 | 0.182 | 0.025 |  |
| 7944365 | CCDC84 | coiled-coil domain containing 84 | 30.912 | −15.993 | −46.905 | −12.308 | −6.128 | 0.182 | 0.024 |  |
| 8084986 | FYTTD1 | forty-two-three domain containing 1 | 20.684 | −8.132 | −28.816 | −15.592 | −7.591 | 0.182 | 0.025 |  |
| 8145989 | HTRA4 | HtrA serine peptidase 4 | −1.922 | 6.470 | 8.392 | 12.087 | 4.120 | 0.183 | 0.025 |  |
| 7990020 | ANP32A | acidic (leucine-rich) nuclear phosphoprotein 32 family, member A | 69.177 | −13.289 | −82.466 | −13.038 | −4.842 | 0.183 | 0.024 |  |
| 8071907 | UPB1 | ureidopropionase, beta | 2.062 | −5.483 | −7.544 | −14.777 | −5.972 | 0.183 | 0.025 |  |
| 8018731 | RHBDF2 | rhomboid 5 homolog 2 (Drosophila) | −22.381 | 11.728 | 34.109 | 14.067 | 2.242 | 0.183 | 0.025 |  |
| 7940673 | GNG3 | guanine nucleotide binding protein (G protein ), gamma 3 | 5.211 | −2.133 | −7.344 | −15.209 | 7.692 | 0.183 | 0.025 |  |
| 7946781 | PLEKHA7 | pleckstrin homology domain containing, family A member 7 | 4.104 | −4.606 | −8.710 | −16.600 | −7.032 | 0.184 | 0.025 |  |
| 7945460 | MIR210 | microRNA 210 | −7.293 | 9.007 | 16.301 | 13.050 | 8.166 | 0.184 | 0.025 |  |
| 8008185 | ABI3 | ABI family, member 3 | −41.104 | 21.448 | 62.551 | 14.161 | 7.921 | 0.184 | 0.024 |  |
| 8038382 | PIH1D1 | PIH1 domain containing 1 | 5.440 | −8.034 | −13.474 | −10.260 | −1.429 | 0.185 | 0.025 |  |
| 7937257 | SCGB1C1 | secretoglobin, family 1C, member 1 | −7.098 | 2.102 | 9.200 | 12.743 | 6.223 | 0.185 | 0.025 |  |
| 8015223 | KRTAP4-12 | keratin associated protein 4-12 | −9.777 | 1.626 | 11.403 | 14.742 | 5.601 | 0.185 | 0.025 |  |
| 8003125 | TAF1C | TATA box binding protein (TBP)-associatedfactor, RNA polymerase I, C, 110kDa | −22.150 | −2.125 | 20.025 | 12.617 | 6.825 | 0.186 | 0.025 |  |
| 8071541 | TMEM191A | transmembrane protein 191A | 8.321 | −17.991 | −26.312 | −11.227 | −6.082 | 0.186 | 0.025 |  |
| 8053036 | TPRKB | TP53RK binding protein | 7.667 | 0.921 | −6.747 | −14.903 | −3.997 | 0.186 | 0.025 |  |
| 7950321 | UCP3 | uncoupling protein 3 (mitochondrial, proton carrier) | −2.453 | −11.675 | −9.222 | −10.503 | −0.797 | 0.186 | 0.025 |  |
| 8160587 | NDUFB6 | NADH dehydrogenase (ubiquinone) 1 beta subcomplex, 6, 17kDa | 12.419 | −1.613 | −14.032 | −13.343 | 1.071 | 0.186 | 0.025 |  |
| 7919612 | HIST2H3D | histone cluster 2, H3d | 33.435 | −4.466 | −37.901 | −14.936 | −4.556 | 0.187 | 0.025 |  |
| 8031999 | PPAP2C | phospholipid phosphatase 2 | −7.319 | 1.185 | 8.504 | 14.531 | 4.383 | 0.187 | 0.025 |  |
| 8090577 | MBD4 | methyl-CpG binding domain protein 4 | 36.341 | 0.425 | −35.916 | −10.169 | −0.020 | 0.187 | 0.024 |  |
| 8137414 | NUB1 | negative regulator of ubiquitin-like proteins 1 | −10.996 | 67.760 | 78.756 | 11.148 | −3.242 | 0.188 | 0.024 |  |
| 8032782 | TMIGD2 | transmembrane and immunoglobulin domain containing 2 | −11.446 | 12.408 | 23.854 | 15.358 | 3.092 | 0.188 | 0.025 |  |
| 7991224 | HAPLN3 | hyaluronan and proteoglycan link protein 3 | −8.626 | 8.564 | 17.190 | 10.466 | 6.914 | 0.188 | 0.025 |  |
| 8068713 | MX1 | MX dynamin-like GTPase 1 | –205.388 | 61.813 | 267.201 | 55.088 | 7.022 | 0.189 | 0.024 |  |
| 8095751 | PARM1 | prostate androgen-regulated mucin-like protein 1 | 13.184 | −2.716 | −15.900 | −25.473 | 6.843 | 0.189 | 0.025 |  |
| 7985493 | TM6SF1 | transmembrane 6 superfamily member 1 | 67.889 | −3.556 | −71.444 | −16.021 | −1.301 | 0.189 | 0.024 |  |
| 8016858 | COIL | coilin | 28.928 | −5.278 | −34.206 | −13.259 | 3.039 | 0.189 | 0.025 |  |
| 8019939 | TGIF1 | TGFB-induced factor homeobox 1 | 2.818 | −6.407 | −9.225 | −12.188 | −3.681 | 0.189 | 0.025 |  |
| 8121515 | SLC16A10 | solute carrier family 16, member 10 (aromatic amino acid transporter) | −2.540 | 25.590 | 28.130 | 18.298 | 7.368 | 0.189 | 0.025 |  |
| 8105136 | ZNF131 | zinc finger protein 131 | 37.529 | −2.354 | −39.883 | −13.743 | 0.206 | 0.190 | 0.024 |  |
| 8113094 | POU5F2 | POU domain class 5, transcription factor 2 | −13.431 | 1.883 | 15.314 | 17.167 | 3.379 | 0.190 | 0.025 |  |
| 8015104 | KRT10 | keratin 10 | −5.911 | 0.897 | 6.808 | 11.625 | 4.051 | 0.190 | 0.025 |  |
| 8062041 | ACSS2 | acyl-CoA synthetase short-chain family member 2 | −5.958 | 16.013 | 21.970 | 10.103 | 1.801 | 0.190 | 0.025 |  |
| 8118028 | VARS2 | valyl-tRNA synthetase 2, mitochondrial | −10.106 | −1.484 | 8.622 | 12.311 | 3.104 | 0.190 | 0.025 |  |
| 7979916 | SYNJ2BP | synaptojanin 2 binding protein | 24.512 | −1.073 | −25.585 | −13.168 | −3.577 | 0.190 | 0.025 |  |
| 8131831 | NUPL2 | nucleoporin like 2 | 13.053 | −2.579 | −15.632 | −12.581 | −1.820 | 0.191 | 0.025 |  |
| 8118682 | PHF1 | PHD finger protein 1 | −48.155 | 4.674 | 52.829 | 11.153 | 4.445 | 0.191 | 0.024 |  |
| 8006433 | CCL2 | chemokine (C–C motif) ligand 2 | −6.644 | 8.226 | 14.870 | 29.470 | 8.951 | 0.192 | 0.025 |  |
| 7902396 | SNORD45C | small nucleolar RNA, C/D box 45C | 12.606 | 3.781 | −8.826 | −17.675 | −0.592 | 0.192 | 0.025 |  |
| 7961320 | PRB2 | proline-rich protein BstNI subfamily 2 | −3.948 | 3.339 | 7.287 | 13.171 | 8.269 | 0.192 | 0.025 |  |
| 8031884 | ZNF544 | zinc finger protein 544 | 11.578 | −9.723 | −21.302 | −17.560 | −4.984 | 0.192 | 0.025 |  |
| 8157233 | HSDL2 | hydroxysteroid dehydrogenase like 2 | 61.972 | −13.172 | −75.144 | −19.057 | −8.477 | 0.192 | 0.024 |  |
| 8069574 | C21orf91 | chromosome 21 open reading frame 91 | 24.698 | 2.312 | −22.386 | −12.225 | −3.894 | 0.192 | 0.025 |  |
| 8143327 | PARP12 | poly (ADP-ribose) polymerase family, member 12 | −54.023 | 27.483 | 81.507 | 14.739 | 7.080 | 0.193 | 0.024 |  |
| 8008627 | NOG | noggin | −18.778 | 9.720 | 28.498 | 23.752 | 9.654 | 0.193 | 0.025 |  |
| 7992867 | FLJ39639 | hypothetical protein FLJ39639 | 36.476 | −53.802 | −90.279 | −11.978 | −3.066 | 0.193 | 0.024 |  |
| 7962441 | TWF1 | twinfilin, actin-binding protein 1 | 19.619 | 2.195 | −17.424 | −10.110 | 0.042 | 0.193 | 0.025 |  |
| 8049620 | FAM132B | family with sequence similarity 132, member B | 0.917 | 17.635 | 16.718 | 20.342 | 7.319 | 0.193 | 0.025 |  |
| 8032284 | UQCR11 | ubiquinol-cytochrome C reductase, complex III subunit XI | 17.051 | −6.392 | −23.443 | −15.851 | −9.934 | 0.193 | 0.025 |  |
| 8117389 | HIST1H2BE | histone cluster 1, H2be | 6.172 | −2.131 | −8.304 | −15.770 | −7.153 | 0.194 | 0.025 |  |
| 8082133 | PDIA5 | protein disulfideisomerase family A, member 5 | 11.437 | −9.158 | −20.595 | −20.955 | −2.077 | 0.194 | 0.025 |  |
| 8086683 | PRSS42 | protease, serine, 42 | −2.159 | 4.054 | 6.212 | 11.327 | 4.314 | 0.195 | 0.025 |  |
| 8087935 | NT5DC2 | 5′-nucleotidase domain containing 2 | 23.080 | −2.785 | −25.865 | −35.883 | 0.452 | 0.195 | 0.025 |  |
| 8004360 | KCTD11 | potassium channel tetramerisation domain containing 11 | −10.260 | −0.744 | 9.516 | 12.232 | 6.201 | 0.195 | 0.025 |  |
| 8086627 | ALS2CL | ALS2C-terminallike | −9.858 | −2.974 | 6.884 | 11.557 | −2.440 | 0.195 | 0.025 |  |
| 7917482 | CCBL2 | cysteine conjugate-beta lyase 2 | 49.994 | −0.903 | −50.897 | −11.750 | 2.893 | 0.195 | 0.024 |  |
| 7995776 | MT3 | metallothionein 3 | −8.032 | 3.927 | 11.959 | 14.667 | 8.129 | 0.196 | 0.025 |  |
| 8008566 | STXBP4 | syntaxin binding protein 4 | 15.411 | −0.423 | −15.834 | −21.251 | −3.911 | 0.196 | 0.025 |  |
| 8073743 | UPK3A | uroplakin 3A | −8.457 | 0.272 | 8.730 | 12.155 | −2.608 | 0.196 | 0.025 |  |
| 8038792 | ETFB | electron-transfer-flavoprotein, beta polypeptide | 5.842 | −4.331 | −10.174 | −10.122 | −3.766 | 0.196 | 0.025 |  |
| 8177901 | VARS2 | valyl-tRNA synthetase 2, mitochondrial | −10.010 | −0.734 | 9.277 | 13.992 | 2.841 | 0.197 | 0.025 |  |
| 8172876 | SMC1A | structural maintenance of chromosome s1A | −39.216 | 6.215 | 45.430 | 11.433 | −1.352 | 0.197 | 0.024 |  |
| 8048505 | STK16 | serine/threonine kinase 16 | 4.047 | −10.593 | −14.639 | −15.990 | −4.474 | 0.198 | 0.025 |  |
| 7938348 | WEE1 | WEE1 G2 checkpoint kinase | 14.526 | −1.551 | −16.077 | −21.652 | 6.405 | 0.199 | 0.025 |  |
| 7943162 | C11orf54 | chromosome 11 open reading frame 54 | 39.905 | 5.259 | −34.645 | −12.668 | −3.667 | 0.199 | 0.024 |  |
| 7977567 | KLHL33 | kelch-like family member 33 | −2.743 | 3.730 | 6.473 | 12.616 | 7.845 | 0.200 | 0.025 |  |
| 8064844 | PCNA | proliferating cell nuclear antigen | 10.174 | −1.776 | −11.951 | −14.538 | −6.222 | 0.200 | 0.025 |  |
| 8018937 | USP36 | ubiquitin specific peptidase 36 | −28.556 | 2.054 | 30.610 | 15.466 | 7.563 | 0.200 | 0.025 |  |
| 8008372 | EPN3 | epsin 3 | −6.878 | −0.673 | 6.205 | 11.245 | 9.709 | 0.200 | 0.025 |  |
| 8078300 | OXSM | 3-oxoacyl-ACP synthase, mitochondrial | 10.239 | 3.284 | −6.954 | −11.581 | −9.249 | 0.200 | 0.025 |  |
| 7907657 | RALGPS2 | Ral GEF with PH domain and SH3 binding motif 2 | 49.688 | 10.716 | −38.972 | −11.839 | 4.447 | 0.200 | 0.024 |  |
| 8139250 | MRPS24 | mitochondrialribosomal protein S24 | 50.900 | 3.374 | −47.526 | −13.104 | −2.893 | 0.200 | 0.024 |  |
| 7934278 | P4HA1 | prolyl4-hydroxylase, alpha polypeptide I | 9.764 | −7.828 | −17.592 | −10.951 | −9.970 | 0.201 | 0.025 |  |
| 8012344 | HES7 | HES family BHLH transcription factor 7 | −3.687 | 2.245 | 5.932 | 10.959 | 6.704 | 0.201 | 0.025 |  |
| 8087653 | ZMYND10 | zinc finger, MYND-type containing 10 | −1.510 | 4.176 | 5.686 | 11.409 | 6.507 | 0.201 | 0.025 |  |
| 8051413 | FAM98A | family with sequence similarity 98, member A | 32.331 | −3.920 | −36.251 | −16.389 | −7.155 | 0.201 | 0.025 |  |
| 7965123 | PPP1R12A | protein phosphatase 1, regulatory subunit 12A | 99.917 | 21.348 | −78.569 | −10.247 | −7.465 | 0.201 | 0.024 |  |
| 7981740 | IGHA1 | immunoglobulin heavy constant alpha 1 | 1104.799 | –250.052 | −1360.851 | −153.900 | 9.783 | 0.201 | 0.023 |  |
| 8136495 | TTC26 | tetratricopeptide repeat domain 26 | −3.141 | 10.324 | 13.465 | 30.607 | −2.534 | 0.201 | 0.025 |  |
| 8053165 | MRPL53 | mitochondrial ribosomal protein L53 | 54.107 | −24.689 | −78.797 | −14.879 | −5.113 | 0.201 | 0.024 |  |
| 8016600 | TAC4 | tachykinin 4 (hemokinin) | −3.464 | 4.718 | 8.181 | 12.730 | 5.334 | 0.201 | 0.025 |  |
| 7934719 | SFTPD | surfactant protein D | −3.749 | 4.137 | 7.885 | 13.636 | 3.846 | 0.201 | 0.025 |  |
| 7967456 | RILPL2 | Rab interacting lysosomal protein -like 2 | 57.457 | −6.366 | −63.822 | −10.969 | −2.309 | 0.202 | 0.024 |  |
| 8013179 | ATPAF2 | ATP synthase mitochondrial F1 complex assembly factor 2 | 18.928 | −7.124 | −26.052 | −12.445 | −5.681 | 0.202 | 0.025 |  |
| 8117194 | MRS2 | MRS2 magnesium transporter | 18.909 | −6.302 | −25.212 | −17.151 | −2.173 | 0.202 | 0.025 |  |
| 7948741 | B3GAT3 | beta-1,3-glucuronyltransferase 3 | 25.751 | −6.907 | −32.658 | −18.239 | 2.259 | 0.203 | 0.025 |  |
| 8021635 | SERPINB2 | serpin peptidase inhibitor, clade B (ovalbumin), member 2 | 14.277 | −10.215 | −24.491 | −22.479 | 2.207 | 0.203 | 0.025 |  |
| 8074558 | RTN4R | reticulon 4 receptor | −6.973 | 2.415 | 9.388 | 10.098 | 8.534 | 0.203 | 0.025 |  |
| 7904050 | MOV10 | Mov 10 RISC complex RNA helicase | −42.394 | 9.480 | 51.874 | 15.836 | 1.549 | 0.204 | 0.024 |  |
| 8103622 | CBR4 | carbonyl reductase 4 | 14.948 | −2.131 | −17.079 | −15.615 | −7.873 | 0.204 | 0.025 |  |
| 8033002 | LONP1 | lon peptidase 1, mitochondrial | −7.150 | 3.489 | 10.640 | 13.739 | 7.286 | 0.204 | 0.025 |  |
| 8006477 | ZNF830 | zinc finger protein 830 | 10.587 | −9.511 | −20.098 | −12.486 | −2.149 | 0.205 | 0.025 |  |
| 7958846 | PTPN11 | protein tyrosinephosphatase, non-receptor type 11 | −21.580 | −2.723 | 18.857 | 10.019 | 1.781 | 0.205 | 0.025 |  |
| 8167815 | MAGED2 | melanoma antigen family D2 | 19.564 | 2.039 | −17.525 | −11.552 | −7.236 | 0.206 | 0.025 |  |
| 8123463 | PHF10 | PHD finger protein 10 | 36.794 | −2.268 | −39.062 | −13.868 | −0.262 | 0.206 | 0.024 |  |
| 8013906 | TWF1 | twinfilin actin binding protein 1 | 17.782 | −0.091 | −17.873 | −12.499 | −2.169 | 0.206 | 0.025 |  |
| 8082305 | CHCHD6 | coiled-coil-helix-coiled-coil-helix domain containing 6 | −2.316 | 6.321 | 8.637 | 12.314 | 4.191 | 0.207 | 0.025 |  |
| 8016336 | OSBPL7 | oxysterol binding protein-like 7 | −20.333 | −1.222 | 19.111 | 16.475 | 3.968 | 0.207 | 0.025 |  |
| 7899284 | TRNP1 | TMF1-regulated nuclear protein 1 | −4.408 | 5.686 | 10.094 | 10.608 | 7.329 | 0.207 | 0.025 |  |
| 7998055 | MC1R | melanocortin 1 receptor (alpha melanocyte stimulating hormone receptor) | −4.783 | 2.791 | 7.575 | 12.214 | 9.690 | 0.207 | 0.025 |  |
| 7961829 | BCAT1 | branched chain amino-acid transaminase 1, cytosolic | −5.008 | 8.368 | 13.376 | 27.680 | 9.794 | 0.207 | 0.025 |  |
| 8065433 | C20orf3 | chromosome 20 open reading frame 3 | 38.592 | −29.723 | −68.316 | −12.136 | −7.354 | 0.208 | 0.024 |  |
| 8049934 | GAL3ST2 | galactose-3-*O*-sulfotransferase 2 | −3.281 | 3.897 | 7.177 | 14.203 | 6.218 | 0.208 | 0.025 |  |
| 8146564 | RAB2A | RAB2A, member RAS oncogene family | 59.722 | −1.515 | −61.237 | −12.716 | −8.277 | 0.208 | 0.024 |  |
| 7992854 | ZNF213 | zinc finger protein 213 | 1.808 | −8.642 | −10.451 | −12.544 | −7.598 | 0.208 | 0.025 |  |
| 8066444 | LOC79015 | hypothetical LOC79015 | −1.271 | 5.751 | 7.022 | 14.536 | 7.850 | 0.209 | 0.025 |  |
| 8069037 | AIRE | autoimmune regulator | −2.831 | 4.995 | 7.826 | 13.546 | 7.048 | 0.210 | 0.025 |  |
| 8031720 | ZNF543 | zinc finger protein 543 | 10.831 | −5.987 | −16.818 | −14.930 | 0.526 | 0.210 | 0.025 |  |
| 8005191 | NCRNA00188 | LRRC75A antisense RNA 1 | 32.581 | −6.233 | −38.814 | −18.165 | −2.719 | 0.210 | 0.025 |  |
| 8083569 | TIPARP | TCDD-inducible poly(ADP-ribose) polymerase | 37.820 | −4.410 | −42.231 | −13.128 | −6.480 | 0.210 | 0.024 |  |
| 7924840 | LOC100506571 | hypothetical protein LOC100506571 | −19.906 | −5.298 | 14.607 | 18.123 | 6.052 | 0.211 | 0.025 |  |
| 8116128 | LOC202181 | SUMO-interacting motif containing 1 psuedogene | 3.476 | −15.318 | −18.794 | −14.150 | −8.923 | 0.211 | 0.025 |  |
| 8141459 | GAL3ST4 | galactose-3-*O*-sulfotransferase 4 | −18.047 | 0.504 | 18.551 | 15.827 | 3.108 | 0.211 | 0.025 |  |
| 8135204 | SPDYE2 | Speedy/RINGO cell cycle regulator family member E2 | −6.603 | 2.809 | 9.412 | 13.024 | 8.517 | 0.212 | 0.025 |  |
| 7904364 | WDR3 | WD repeat domain 3 | 5.165 | −6.547 | −11.712 | −16.610 | −9.066 | 0.212 | 0.025 |  |
| 8038725 | KLK10 | kallikrein-related peptidase 10 | 3.405 | −2.683 | −6.087 | −12.904 | −1.313 | 0.212 | 0.025 |  |
| 8033780 | ZNF426 | zinc finger protein 426 | 11.063 | −8.212 | −19.276 | −13.233 | −0.826 | 0.212 | 0.025 |  |
| 8090420 | TPRA1 | transmembrane protein, adipocyte asscociated 1 | −20.928 | 3.812 | 24.740 | 10.251 | 5.081 | 0.213 | 0.025 |  |
| 8098725 | DUX4L4 | double homeobox 4 like 4 | −13.781 | −0.412 | 13.369 | 14.700 | 7.170 | 0.213 | 0.025 |  |
| 7967969 | IL17D | interleukin 17D | −4.767 | 5.312 | 10.079 | 14.875 | 4.133 | 0.213 | 0.025 |  |
| 8081676 | GTPBP8 | GTP-binding protein 8 (putative) | 28.581 | 8.456 | −20.125 | −14.957 | −6.397 | 0.213 | 0.025 |  |
| 7981718 | IGHM | immunoglobulin heavy constant mu | 339.779 | −30.146 | −369.925 | −38.144 | 9.887 | 0.214 | 0.023 |  |
| 7999718 | C16orf63 | chromosome 16 open reading frame 63 | 18.725 | −1.475 | −20.201 | −14.803 | −7.453 | 0.214 | 0.025 |  |
| 8146914 | TERF1 | telomeric repeat binding factor (NIMA-interacting) 1 | 62.926 | 1.209 | −61.717 | −14.680 | −4.645 | 0.214 | 0.024 |  |
| 7949971 | CPT1A | carnitine palmitoyltransferase 1A (liver) | 18.262 | −50.803 | −69.065 | −16.024 | −2.346 | 0.214 | 0.024 |  |
| 8113083 | FAM172A | family with sequence similarity 172, member A | 44.240 | 4.316 | −39.923 | −11.320 | −1.262 | 0.214 | 0.024 |  |
| 8062206 | ROMO1 | reactive oxygen species modulator 1 | 10.184 | −4.972 | −15.156 | −14.329 | −3.006 | 0.214 | 0.025 |  |
| 8024966 | RPL36 | ribosomal protein L36 | 37.475 | −2.955 | −40.430 | −10.603 | 0.889 | 0.215 | 0.024 |  |
| 7936091 | USMG5 | up-regulated during skeletal muscle growth 5 homolog (mouse) | 45.527 | −3.886 | −49.413 | −20.097 | −1.759 | 0.216 | 0.025 |  |
| 7958884 | OAS1 | 2′, 5′-oligoadenylate synthetase 1, 40/46kDa | –223.350 | 73.804 | 297.154 | 34.356 | 3.887 | 0.216 | 0.023 |  |
| 8007069 | RNY4P8 | RNA, Ro-associated Y4 pseudogene 8 | −24.419 | −3.224 | 21.194 | 10.175 | −6.761 | 0.216 | 0.025 |  |
| 7931455 | LRRC27 | leucine rich repeat containing 27 | −7.187 | 1.422 | 8.609 | 11.274 | −0.464 | 0.217 | 0.025 |  |
| 8102214 | PAPSS1 | 3′-phosphoadenosine 5′-phosphosulfate synthase 1 | 34.560 | −26.849 | −61.409 | −25.945 | −9.565 | 0.217 | 0.025 |  |
| 8141024 | BET1 | Bet1 golgi vesicular membrane trafficking protein | 16.674 | −1.131 | −17.805 | −13.892 | 2.056 | 0.217 | 0.025 |  |
| 8026294 | LOC113230 | uncharaterized protein LOC113230 | 4.137 | −3.185 | −7.323 | −11.260 | −0.012 | 0.217 | 0.025 |  |
| 7915733 | PRDX1 | peroxiredoxin 1 | 42.495 | −16.601 | −59.097 | −11.828 | −5.118 | 0.218 | 0.024 |  |
| 8063410 | PARD6B | par-6p family cell polarity regulator beta | 15.586 | −1.297 | −16.883 | −10.018 | −2.410 | 0.218 | 0.025 |  |
| 8176570 | DUX4L4 | double homeobox 4 like 4 | −11.117 | −0.391 | 10.725 | 14.559 | 6.870 | 0.218 | 0.025 |  |
| 8003611 | FAM57A | family with sequence similarity 57, member A | −2.300 | 4.392 | 6.693 | 10.697 | 3.215 | 0.218 | 0.025 |  |
| 8161147 | HINT2 | histidine triad nucleotide binding protein 2 | 28.190 | −0.821 | −29.012 | −10.330 | 6.187 | 0.218 | 0.024 |  |
| 8039511 | ISOC2 | isochorismatase domain containing 2 | 14.395 | −1.637 | −16.032 | −13.890 | 6.900 | 0.219 | 0.025 |  |
| 7897006 | MMP23B | matrix metallopeptidase 23B | −9.259 | 3.601 | 12.860 | 12.475 | 9.593 | 0.219 | 0.025 |  |
| 8104998 | WDR70 | WD repeat domain 70 | 8.918 | −12.580 | −21.498 | −14.931 | −8.339 | 0.219 | 0.025 |  |
| 8032518 | DIRAS1 | DIRAS family, GTP-binding RAS-like 1 | −11.714 | 0.562 | 12.276 | 13.175 | 5.628 | 0.220 | 0.025 |  |
| 7992439 | GFER | growth factor, augmenter of liver regeneration | −3.790 | 3.159 | 6.950 | 12.184 | 7.858 | 0.220 | 0.025 |  |
| 8062766 | MYBL2 | V-Myb avian myeloblastosis viral oncogene homolog-like 2 | 27.713 | 2.370 | −25.343 | −20.927 | −0.289 | 0.220 | 0.025 |  |
| 7993185 | NUBP1 | nucleotide binding protein 1 | 21.245 | −12.690 | −33.935 | −13.755 | −4.854 | 0.220 | 0.025 |  |
| 8081362 | CEP97 | centrosomal protein 97kDa | 13.072 | 0.998 | −12.073 | −11.557 | −8.604 | 0.221 | 0.025 |  |
| 7964119 | STAT2 | signal transducer and activator of transcription 2, 113kDa | –108.632 | 22.936 | 131.568 | 23.482 | 2.867 | 0.221 | 0.024 |  |
| 8025984 | ZNF844 | zinc finger protein 844 | 11.912 | −1.341 | −13.252 | −16.811 | −7.853 | 0.222 | 0.025 |  |
| 8146930 | TMEM70 | transmembrane protein 70 | 7.111 | −8.085 | −15.196 | −13.755 | −1.561 | 0.222 | 0.025 |  |
| 8056363 | SLC38A11 | solute carrier family 38, member 11 | 9.598 | −2.722 | −12.320 | −19.669 | −5.438 | 0.222 | 0.025 |  |
| 8160024 | GLDC | glycine dehydrogenase (decarboxylating) | 9.186 | −9.828 | −19.014 | −28.084 | −5.978 | 0.222 | 0.025 |  |
| 8050102 | CMPK2 | cytidine monophosphate (UMP-CMP) kinase 2, mitochondrial | −84.545 | 32.110 | 116.655 | 43.219 | 9.734 | 0.222 | 0.025 |  |
| 7936777 | NSMCE4A | NSE4 homolog A, SMC5-SMC6 complex component | 20.662 | 1.214 | −19.448 | −12.145 | −6.284 | 0.222 | 0.025 |  |
| 8073890 | GRAMD4 | GRAM domain containing 4 | 6.525 | −12.529 | −19.053 | −14.135 | −0.194 | 0.223 | 0.025 |  |
| 8098769 | MFSD7 | major facilitator superfamily domain containing 7 | −12.138 | 2.343 | 14.480 | 10.529 | 6.559 | 0.223 | 0.025 |  |
| 7957271 | LOC100289143 | NOP10 ribonucleoprotein homolog (yeast) pseudogene | 5.351 | −1.071 | −6.422 | −11.978 | −1.214 | 0.224 | 0.025 |  |
| 8164907 | REXO4 | REX4 homolog, 3′-5′ exonuclease | −6.683 | 2.414 | 9.097 | 15.134 | 1.790 | 0.224 | 0.025 |  |
| 8103922 | CASP3 | caspase 3, apoptosis-related cysteine peptidase | 22.744 | −1.813 | −24.557 | −12.125 | −2.740 | 0.225 | 0.025 |  |
| 8149574 | CSGALNACT1 | chondroitin sulfate *N*-acetylgalactosaminyltransferase 1 | 31.472 | −3.359 | −34.831 | −14.196 | −4.381 | 0.225 | 0.025 |  |
| 8099850 | TMEM156 | transmembrane protein 156 | 23.151 | 8.211 | −14.940 | −12.274 | 4.069 | 0.225 | 0.025 |  |
| 7971296 | EPSTI1 | epithelial stromal interaction 1 (breast) | −42.070 | 36.159 | 78.228 | 48.666 | 8.338 | 0.225 | 0.025 |  |
| 8076002 | SLC16A8 | solute carrier family 16 (monocarboxylate transporter), member 8 | −3.562 | 6.268 | 9.830 | 14.784 | −0.996 | 0.225 | 0.025 |  |
| 8167476 | PPP1R3F | protein phosphatase1, regulatory subunit 3F | 0.632 | −11.693 | −12.325 | −11.175 | −4.806 | 0.225 | 0.025 |  |
| 7979416 | TIMM9 | translocase of inner mitochondrial membrane 9 homolog (yeast) | 7.959 | −4.367 | −12.326 | −18.767 | −8.579 | 0.226 | 0.025 |  |
| 7958379 | PWP1 | PWP1 homolog, endonuclein | 21.826 | 1.906 | −19.920 | −10.530 | 0.109 | 0.226 | 0.025 |  |
| 8008517 | NME1 | NME/NM23 nucleoside disphosphate kinase 1 | 11.439 | −0.174 | −11.614 | −12.098 | −4.306 | 0.226 | 0.025 |  |
| 7990027 | C15orf28 | chromosome 15 open reading frame 28 | 13.316 | −32.888 | −46.203 | −11.299 | −4.513 | 0.227 | 0.024 |  |
| 8023549 | RAX | retina and anterior neural fold homeobox | −8.137 | 0.482 | 8.619 | 16.796 | 3.239 | 0.227 | 0.025 |  |
| 7953520 | LRRC23 | leucine rich repeat containing 23 | −3.033 | 3.296 | 6.329 | 11.400 | 8.295 | 0.227 | 0.025 |  |
| 7915870 | ATPAF1 | ATP synthase mitochondrial F1 complex assembly factor 1 | 8.441 | −1.723 | −10.164 | −10.464 | −9.641 | 0.228 | 0.025 |  |
| 7958200 | EID3 | EP300 interacting inhibitor of differentiation 3 | 10.203 | −6.064 | −16.267 | −11.047 | −4.128 | 0.228 | 0.025 |  |
| 8159981 | RLN1 | relaxin 1 | 6.623 | −0.094 | −6.717 | −14.439 | −6.175 | 0.228 | 0.025 |  |
| 7946680 | BTBD10 | BTB(POZ) domain containing 10 | 37.766 | 3.190 | −34.576 | −11.855 | −6.523 | 0.228 | 0.024 |  |
| 7910001 | DEGS1 | delta(4)-desaturase, sphingolipid 1 | 38.520 | −0.065 | −38.585 | −13.526 | −4.722 | 0.229 | 0.024 |  |
| 7983606 | EID1 | EP300 interacting inhibitor of differentiation 1 | 29.655 | −3.366 | −33.021 | −16.303 | −7.600 | 0.229 | 0.025 |  |
| 8086555 | LZTFL1 | leucine zipper transcription factor -like 1 | 7.730 | −0.746 | −8.476 | −13.010 | −7.394 | 0.229 | 0.025 |  |
| 8042874 | TTC31 | tetratricopeptide repeat domain 31 | 0.745 | −14.699 | −15.443 | −10.766 | −6.484 | 0.229 | 0.025 |  |
| 8133741 | UPK3B | uroplakin 3B | −14.585 | −2.498 | 12.087 | 15.056 | 8.678 | 0.229 | 0.025 |  |
| 8141522 | C7orf61 | chromosome 7 open reading frame 61 | −1.668 | 4.638 | 6.306 | 10.752 | 3.922 | 0.230 | 0.025 |  |
| 8048195 | RPL37A | ribosomal protein L37a | 30.964 | −12.146 | −43.110 | −13.463 | −4.523 | 0.230 | 0.024 |  |
| 8099235 | MRFAP1L1 | Morf4 family associated protein 1-like 1 | 16.940 | −5.719 | −22.659 | −12.592 | 4.681 | 0.230 | 0.025 |  |
| 8030871 | ZNF613 | zinc finger protein 613 | 5.889 | −4.882 | −10.771 | −10.472 | −0.254 | 0.230 | 0.025 |  |
| 8122684 | SUMO4 | Small ubiquitin-like modifier 4 | 33.020 | −4.971 | −37.991 | −13.167 | −6.069 | 0.231 | 0.024 |  |
| 8029360 | ZNF223 | zinc finger protein 223 | 7.020 | 0.137 | −6.883 | −10.633 | −3.328 | 0.231 | 0.025 |  |
| 7995258 | ZNF267 | zinc finger protein 267 | 64.126 | 9.519 | −54.607 | −20.089 | −9.947 | 0.231 | 0.024 |  |
| 7945864 | ZNF195 | zinc finger protein 195 | 31.225 | −0.138 | −31.363 | −13.697 | −5.034 | 0.231 | 0.025 |  |
| 8107859 | LYRM7 | LYR motif containing 7 | 14.864 | 2.115 | −12.748 | −17.017 | −3.918 | 0.231 | 0.025 |  |
| 8096160 | ARHGAP24 | Rho GTPase activating protein 24 | 8.503 | −18.852 | −27.356 | −15.686 | −3.348 | 0.231 | 0.025 |  |
| 8163999 | ZBTB6 | zinc finger and BTB domain containing 6 | 13.866 | −2.279 | −16.145 | −14.521 | 0.363 | 0.231 | 0.025 |  |
| 8150565 | RNF170 | ring finger protein 170 | 12.129 | −3.277 | −15.406 | −10.206 | −4.343 | 0.232 | 0.025 |  |
| 8136940 | FAM115C | family with sequence similarity 115, member C | −12.761 | 6.335 | 19.096 | 10.174 | 8.271 | 0.232 | 0.025 |  |
| 7967030 | RNU4-1 | RNA, U4 small nuclear 1 | –120.206 | 67.636 | 187.842 | 15.705 | 2.083 | 0.232 | 0.023 |  |
| 8162729 | TRIM14 | tripartite motif-containing 14 | −50.360 | 11.942 | 62.302 | 10.337 | −1.007 | 0.232 | 0.024 |  |
| 7947649 | CHRM4 | cholinergic receptor, muscarinic 4 | −5.337 | 4.722 | 10.059 | 16.088 | 8.880 | 0.233 | 0.025 |  |
| 7949377 | FAU | Finkel-Biskis-Reilly murine sarcoma virus (FBR-MuSV) ubiquitously expressed | 18.713 | −5.303 | −24.016 | −10.997 | 0.001 | 0.233 | 0.025 |  |
| 8165808 | XG | Xg blood group | 3.137 | −6.614 | −9.750 | −16.940 | 1.335 | 0.233 | 0.025 |  |
| 7959772 | NCOR2 | nuclear receptor corepressor 2 | −33.371 | −14.679 | 18.692 | 20.152 | 0.424 | 0.233 | 0.025 |  |
| 8113250 | ERAP1 | endoplasmic reticulum aminopeptidase 1 | −47.040 | 11.028 | 58.068 | 10.019 | 0.162 | 0.233 | 0.024 |  |
| 8110666 | TRIM52 | tripartite motif-containing 52 | 34.957 | −9.101 | −44.059 | −25.420 | −4.369 | 0.234 | 0.025 |  |
| 8109585 | LSM11 | LSM11, U7 small nuclear RNA associated | 0.579 | −6.953 | −7.532 | −14.169 | −9.608 | 0.234 | 0.025 |  |
| 7926356 | PTER | phosphotriesterase related | 39.528 | 8.157 | −31.371 | −14.687 | 5.129 | 0.234 | 0.025 |  |
| 8004431 | POLR2A | polymerase (RNA) II (DNA directed) polypeptide A, 220kDa | –110.847 | −18.850 | 91.997 | 15.480 | −0.497 | 0.235 | 0.024 |  |
| 8153896 | ZNF34 | zinc finger protein 34 | 3.446 | −4.570 | −8.016 | −10.318 | 0.393 | 0.235 | 0.025 |  |
| 8040265 | KCNF1 | Potassium channel, voltage gated modifier subfamily F, member 1 | −3.108 | 5.251 | 8.359 | 14.269 | 9.036 | 0.235 | 0.025 |  |
| 8066074 | DSN1 | DSN1 homolog, MIS12 kinetochore complex component | 16.405 | −0.267 | −16.672 | −10.293 | −3.259 | 0.235 | 0.025 |  |
| 7928679 | PLAC9 | placenta-specific 9 | −3.231 | 5.134 | 8.364 | 16.497 | 8.499 | 0.235 | 0.025 |  |
| 8035465 | ISYNA1 | inositol-3-phosphate synthase 1 | −6.076 | 6.988 | 13.064 | 10.133 | 7.570 | 0.235 | 0.025 |  |
| 8025868 | CCDC159 | coiled-coil domain containing 159 | 18.684 | −2.870 | −21.554 | −12.286 | −2.374 | 0.236 | 0.025 |  |
| 8010287 | C1QTNF1 | C1q and tumor necrosis factor related protein 1 | −4.963 | 3.434 | 8.398 | 14.846 | 9.688 | 0.236 | 0.025 |  |
| 8084128 | TTC14 | tetratricopeptide repeat domain 14 | 36.965 | −4.674 | −41.639 | −12.740 | −8.905 | 0.237 | 0.024 |  |
| 8003953 | PSMB6 | proteasome subunit beta 6 | 93.830 | 14.429 | −79.401 | −13.076 | −7.313 | 0.237 | 0.024 |  |
| 8147548 | POP1 | POP1 homolog, ribonuclease P/MRP subunit | 7.205 | 0.393 | −6.811 | −12.831 | −0.019 | 0.237 | 0.025 |  |
| 8124134 | TPMT | thiopurine S-methyltransferase | 12.163 | −5.053 | −17.216 | −20.250 | −7.204 | 0.237 | 0.025 |  |
| 8013696 | KIAA0100 | KIAA0100 | −55.513 | −7.806 | 47.707 | 10.805 | 4.780 | 0.237 | 0.024 |  |
| 8071559 | SDF2L1 | stromal cell-derived factor 2-like 1 | 5.882 | −3.570 | −9.453 | −11.023 | −3.692 | 0.237 | 0.025 |  |
| 7991070 | HDGFRP3 | hepatoma-derived growth factor, related protein 3 | 15.773 | 4.457 | −11.316 | −15.085 | −4.574 | 0.238 | 0.025 |  |
| 7925589 | SMYD3 | SET and MYND domain containing 3 | 10.668 | −1.859 | −12.527 | −12.848 | −2.939 | 0.238 | 0.025 |  |
| 8036252 | CLIP3 | CAP-GLY domain containing linker protein 3 | −7.328 | 0.764 | 8.092 | 10.421 | 9.658 | 0.238 | 0.025 |  |
| 8071823 | CABIN1 | calcineurin binding protein 1 | −29.745 | 1.249 | 30.994 | 10.190 | 3.268 | 0.238 | 0.024 |  |
| 8083749 | PPM1L | protein phosphatase, Mg2+/Mn2+ dependent, 1L | −11.668 | 3.269 | 14.937 | 11.854 | 0.522 | 0.238 | 0.025 |  |
| 7946957 | SAAL1 | serum amyloid A-like 1 | 12.139 | −5.244 | −17.383 | −15.479 | −5.156 | 0.238 | 0.025 |  |
| 7962349 | GXYLT1 | glucoside xylosyltransferase 1 | 16.090 | −0.421 | −16.510 | −11.903 | −2.034 | 0.238 | 0.025 |  |
| 7952132 | SLC37A4 | solute carrier family 37 (glucose-6-phosphate transporter), member 4 | −16.516 | 0.492 | 17.008 | 11.504 | 8.698 | 0.239 | 0.025 |  |
| 8040090 | RNF144A | ring finger protein 144A | −8.193 | 13.355 | 21.548 | 10.273 | −3.832 | 0.239 | 0.025 |  |
| 8038919 | ZNF350 | zinc finger protein 350 | 13.468 | −6.534 | −20.002 | −15.178 | −6.924 | 0.239 | 0.025 |  |
| 8174288 | MCART6 | solute carrier family 25, member 53 | 15.198 | 0.078 | −15.120 | −10.223 | 5.118 | 0.239 | 0.025 |  |
| 7962427 | PUS7L | pseudouridylate synthase 7-like | 15.235 | −0.502 | −15.737 | −16.608 | −1.882 | 0.239 | 0.025 |  |
| 7997933 | RPL13 | ribosomal protein L13 | −3.110 | 11.286 | 14.396 | 14.807 | 2.869 | 0.239 | 0.025 |  |
| 7910589 | KIAA1383 | Microtubule-associated protein 10 | 5.124 | −0.785 | −5.909 | −10.654 | −2.529 | 0.239 | 0.025 |  |
| 7931656 | DUX4L4 | double homeobox 4 like 4 | −10.732 | −0.464 | 10.268 | 14.019 | 5.912 | 0.240 | 0.025 |  |
| 7931659 | DUX4L4 | double homeobox 4 like 4 | −10.732 | −0.464 | 10.268 | 14.019 | 5.912 | 0.240 | 0.025 |  |
| 7931662 | DUX4L4 | double homeobox 4 like 4 | −10.732 | −0.464 | 10.268 | 14.019 | 5.912 | 0.240 | 0.025 |  |
| 7931668 | DUX4L4 | double homeobox 4 like 4 | −10.732 | −0.464 | 10.268 | 14.019 | 5.912 | 0.240 | 0.025 |  |
| 7931671 | DUX4L4 | double homeobox 4 like 4 | −10.732 | −0.464 | 10.268 | 14.019 | 5.912 | 0.240 | 0.025 |  |
| 8021357 | ONECUT2 | one cut homeobox 2 | −3.335 | 5.075 | 8.410 | 16.106 | 4.705 | 0.240 | 0.025 |  |
| 8156873 | INVS | Inversin | 14.175 | −9.197 | −23.372 | −13.181 | −6.080 | 0.240 | 0.025 |  |
| 7981290 | WARS | tryptophanyl-tRNA synthetase | −68.007 | 73.815 | 141.822 | 15.661 | −6.794 | 0.240 | 0.023 |  |
| 8017106 | TRIM37 | tripartite motif-containing 37 | 13.382 | −6.073 | −19.455 | −11.855 | −7.477 | 0.240 | 0.025 |  |
| 8142993 | LOC100128596 | NADH dehydrogenase (ubiquinone) 1 beta subcomplex, 9, 22kDa pseudogene | −0.565 | −7.157 | −6.592 | −10.832 | −7.558 | 0.240 | 0.025 |  |
| 8057933 | GTF3C3 | general transcription factor IIIC, polypeptide 3, 102kDa | 26.117 | −1.201 | −27.318 | −17.679 | −4.742 | 0.240 | 0.025 |  |
| 7914127 | IFI6 | interferon, alpha-inducible protein 6 | –315.204 | 199.578 | 514.781 | 43.371 | 5.599 | 0.240 | 0.023 |  |
| 8036686 | FBXO17 | F-box protein 17 | −11.707 | 0.034 | 11.742 | 14.120 | 9.151 | 0.240 | 0.025 |  |
| 8040365 | TRIB2 | tribbles pseudokinase 2 | −15.257 | 14.776 | 30.033 | 10.571 | 4.888 | 0.240 | 0.025 |  |
| 8121429 | FIG4 | FIG4 phosphoinositide 5-phosphate | 24.512 | −12.782 | −37.294 | −10.291 | −6.674 | 0.241 | 0.024 |  |
| 8006409 | CDK5R1 | cyclin-dependent kinase 5, regulatory subunit 1 (p35) | 3.347 | −16.374 | −19.721 | −16.435 | −2.108 | 0.241 | 0.025 |  |
| 7995674 | IRX6 | iroquois homeobox 6 | −2.425 | 8.759 | 11.184 | 19.236 | 2.784 | 0.241 | 0.025 |  |
| 8112961 | RPS23 | ribosomal protein S23 | 74.213 | −38.417 | −112.629 | −16.191 | −8.620 | 0.241 | 0.024 |  |
| 8015189 | KRTAP1-3 | keratin associated protein 1-3 | −8.007 | 0.404 | 8.412 | 13.481 | 7.391 | 0.241 | 0.025 |  |
| 8019585 | KRTAP1-3 | keratin associated protein 1-3 | −8.007 | 0.404 | 8.412 | 13.481 | 7.391 | 0.241 | 0.025 |  |
| 8079334 | LIMD1 | LIM domains containing 1 | 5.675 | −17.202 | −22.876 | −10.084 | −6.434 | 0.241 | 0.025 |  |
| 8026051 | RNASEH2A | ribonuclease H2, subunit A | −8.841 | 3.541 | 12.382 | 10.311 | 9.550 | 0.241 | 0.025 |  |
| 8046628 | RBM45 | RNA binding motif protein 45 | 17.373 | −3.325 | −20.698 | −17.494 | −8.751 | 0.242 | 0.025 |  |
| 8117888 | RAN | RAN, member RAS oncogene family | 29.636 | 7.788 | −21.848 | −10.939 | −1.274 | 0.243 | 0.025 |  |
| 8116070 | PDLIM7 | PDZ and LIM domain 7 (enigma) | 18.111 | −23.885 | −41.996 | −11.614 | 2.845 | 0.243 | 0.024 |  |
| 7958130 | HSP90B1 | heat shock protein 90kDa beta (Grp94), member 1 | 259.992 | 0.247 | −259.746 | −24.376 | −6.157 | 0.243 | 0.023 |  |
| 8165416 | ENTPD2 | ectonucleoside triphosphate diphosphohydrolase 2 | −3.564 | 3.322 | 6.885 | 12.300 | 3.323 | 0.243 | 0.025 |  |
| 8029829 | LOC400707 | uncharacterized LOC400707 | 7.802 | −0.931 | −8.733 | −13.991 | −1.007 | 0.243 | 0.025 |  |
| 8124524 | HIST1H2AK | histone cluster 1, H2ak | 7.732 | −6.329 | −14.061 | −12.264 | 0.591 | 0.244 | 0.025 |  |
| 8176255 | TMLHE | trimethyllysine hydroxylase, epsilon | 66.017 | −8.253 | −74.269 | −14.203 | −7.781 | 0.245 | 0.024 |  |
| 8172244 | FUNDC1 | FUN14 domain containing 1 | 8.235 | −6.047 | −14.282 | −16.349 | −3.420 | 0.245 | 0.025 |  |
| 7898653 | FAM43B | family with sequence similarity 43, member B | −7.921 | 5.798 | 13.720 | 10.216 | 8.763 | 0.246 | 0.025 |  |
| 8178188 | RNF5P1 | ring finger protein 5 pseudogene 1 | 22.890 | −26.646 | −49.535 | −10.150 | 1.440 | 0.246 | 0.024 |  |
| 7941255 | TIGD3 | tigger transposable element derived 3 | −19.233 | −1.659 | 17.574 | 12.840 | 2.643 | 0.246 | 0.025 |  |
| 7991406 | PRC1 | protein regulator of cytokinesis 1 | 5.480 | −0.009 | −5.489 | −10.399 | −9.184 | 0.246 | 0.025 |  |
| 7999478 | TXNDC11 | thioredoxin domain containing 11 | 43.823 | −0.547 | −44.370 | −18.152 | 0.903 | 0.247 | 0.025 |  |
| 8099918 | UBE2K | ubiquitin-conjugating enzyme E2K | −2.340 | 4.643 | 6.983 | 12.307 | 9.116 | 0.247 | 0.025 |  |
| 7963265 | POU6F1 | POU class 6 homeobox 1 | −23.812 | −5.209 | 18.603 | 10.664 | 1.756 | 0.247 | 0.025 |  |
| 8143575 | EPHA1 | EPH receptor A1 | −17.169 | 0.973 | 18.142 | 14.551 | 5.016 | 0.247 | 0.025 |  |
| 8087611 | HYAL1 | hyaluronoglucosaminidase 1 | −2.324 | 4.224 | 6.548 | 12.629 | 5.878 | 0.248 | 0.025 |  |
| 7939767 | MADD | MAP-kinase activating death domain | −87.755 | −12.462 | 75.293 | 12.025 | 1.616 | 0.248 | 0.024 |  |
| 7961386 | GPRC5D | G protein-coupled receptor, family C, group 5, member D | 25.905 | −6.152 | −32.057 | −73.489 | 0.416 | 0.248 | 0.025 |  |
| 8039771 | ZNF132 | zinc finger protein 132 | 8.113 | −1.491 | −9.604 | −10.253 | 5.993 | 0.249 | 0.025 |  |
| 7978586 | CFL2 | cofilin 2 (muscle) | 17.195 | 2.832 | −14.363 | −19.856 | −9.963 | 0.249 | 0.025 |  |
| 8006540 | SNORD7 | small nucleolar RNA, C/D box 7 | −6.512 | 2.420 | 8.932 | 16.491 | −1.071 | 0.250 | 0.025 |  |
| 8060334 | SOX12 | SRY (sex determining region Y)-box 12 | −1.838 | 8.706 | 10.545 | 14.091 | 9.147 | 0.250 | 0.025 |  |
| 8098730 | DUX4 | double homeobox 4 | −11.168 | −0.819 | 10.349 | 13.792 | 3.888 | 0.250 | 0.025 |  |
| 8098743 | DUX4 | double homeobox 4 | −11.168 | −0.819 | 10.349 | 13.792 | 3.888 | 0.250 | 0.025 |  |
| 8142821 | KCP | kielin/chordin-like protein | −9.286 | −0.393 | 8.893 | 11.542 | −6.464 | 0.250 | 0.025 |  |
| 8112902 | DHFR | dihydrofolate reductase | 22.191 | −1.678 | −23.869 | −14.298 | −4.539 | 0.251 | 0.025 |  |
| 8113220 | ELL2 | elongation factor, RNA polymerase II, 2 | 42.875 | 6.444 | −36.432 | −18.110 | −4.442 | 0.251 | 0.025 |  |
| 8063074 | ZSWIM1 | zinc finger, SWIM-type containing 1 | 21.318 | −8.822 | −30.140 | −10.196 | −4.524 | 0.251 | 0.024 |  |
| 7973584 | RNF31 | ring finger protein 31 | −29.047 | −0.677 | 28.370 | 10.239 | 4.828 | 0.251 | 0.025 |  |
| 7945631 | MOB2 | MOB kinase activator 2 | 7.366 | −1.951 | −9.317 | −12.552 | −7.256 | 0.252 | 0.025 |  |
| 8135955 | CALU | Calumenin | 19.240 | 2.415 | −16.826 | −12.544 | −4.482 | 0.252 | 0.025 |  |
| 8117219 | ACOT13 | acyl-CoA thioesterase 13 | 21.112 | 0.796 | −20.316 | −12.774 | −7.757 | 0.252 | 0.025 |  |
| 8099912 | C4orf34 | chromosome 4 open reading frame 34 | 20.806 | −3.003 | −23.809 | −11.125 | −2.153 | 0.252 | 0.025 |  |
| 7983616 | GALK2 | galactokinase 2 | 11.896 | −6.963 | −18.859 | −10.624 | −4.579 | 0.252 | 0.025 |  |
| 7961440 | PLBD1 | Phospholipase B domain containing 1 | 135.423 | −58.075 | −193.499 | −13.381 | −2.163 | 0.252 | 0.022 |  |
| 8088680 | C3orf64 | chromosome 3 open reading frame 64 | −9.298 | 5.571 | 14.869 | 13.338 | 4.349 | 0.253 | 0.025 |  |
| 8099834 | TLR1 | toll-like receptor 1 | 120.432 | 26.739 | −93.693 | −12.051 | −6.273 | 0.253 | 0.024 |  |
| 8141750 | SPDYE2 | speedy/RINGO cell cycle regulator family member E2 | −7.487 | 2.351 | 9.838 | 14.473 | 8.935 | 0.253 | 0.025 |  |
| 8036302 | LOC100127980 | hypothetical protein LOC100127980 | 6.118 | −3.191 | −9.309 | −15.219 | 5.328 | 0.253 | 0.025 |  |
| 8028600 | NCCRP1 | non-specific cytotoxic cell receptor protein 1 homolog (zebrafish) | −4.631 | 6.048 | 10.678 | 15.027 | 9.518 | 0.253 | 0.025 |  |
| 7965627 | LTA4H | leukotriene A4 hydrolase | 99.773 | −42.296 | −142.069 | −11.824 | 1.484 | 0.253 | 0.023 |  |
| 7900201 | UTP11L | UTP11-like, U3 small nucleolar ribonucleoprotein (yeast) | 15.977 | −0.660 | −16.638 | −10.765 | 3.592 | 0.253 | 0.025 |  |
| 8115886 | THOC3 | THO complex 3 | −13.215 | 7.469 | 20.684 | 10.564 | 5.901 | 0.253 | 0.025 |  |
| 8117535 | HIST1H2AG | histone cluster 1, H2ag | 30.781 | 1.108 | −29.673 | −16.887 | −6.044 | 0.254 | 0.025 |  |
| 7992145 | SSTR5 | somatostatin receptor 5 | −0.316 | 5.275 | 5.591 | 10.152 | 3.809 | 0.254 | 0.025 |  |
| 8137693 | COX19 | COX19 cytochrome C oxidase assembly factor | −1.149 | −10.705 | −9.556 | −10.324 | −8.662 | 0.254 | 0.025 |  |
| 7978831 | PPIL5 | leucine rich repeat protein 1 | 6.245 | −3.377 | −9.623 | −18.512 | −4.999 | 0.254 | 0.025 |  |
| 8011141 | PRPF8 | pre-mRNA processing factor 8 | –124.110 | 19.771 | 143.881 | 10.515 | −2.849 | 0.254 | 0.023 |  |
| 8080714 | FLNB | filamin B, beta | −35.534 | −8.764 | 26.770 | 12.600 | 1.965 | 0.254 | 0.025 |  |
| 8089314 | IFT57 | intraflagellar transport 57 | 18.716 | 4.483 | −14.233 | −13.892 | 5.744 | 0.254 | 0.025 |  |
| 8111952 | C5orf28 | chromosome 5 open reading frame 28 | 10.621 | 0.175 | −10.446 | −16.128 | −9.255 | 0.254 | 0.025 |  |
| 8027205 | NDUFA13 | NADH dehydrogenase (ubiquinone) 1 alpha subcomplex, 13 | −7.520 | 0.890 | 8.410 | 10.561 | 7.646 | 0.254 | 0.025 |  |
| 8105353 | SKIV2L2 | superkiller viralicidic activity2-like 2 (S.cerevisiae) | 57.573 | 9.303 | −48.270 | −12.819 | −3.958 | 0.254 | 0.024 |  |
| 8035773 | ZNF506 | zinc finger protein 506 | 69.167 | 5.426 | −63.741 | −14.996 | −9.091 | 0.255 | 0.024 |  |
| 8141311 | FAM200A | family with sequence similarity 200, member A | 5.643 | −4.205 | −9.849 | −19.858 | −7.230 | 0.255 | 0.025 |  |
| 8086125 | TRANK1 | tetratricopeptide repeat and ankyrin repeat containing 1 | −76.332 | 15.695 | 92.027 | 11.859 | −6.967 | 0.255 | 0.023 |  |
| 8116559 | IRF4 | interferon regulatory factor 4 | 32.867 | −11.024 | −43.891 | −19.959 | 5.247 | 0.255 | 0.025 |  |
| 8031981 | WASH3P | WAS protein family homolog 3 pseudogene | −70.001 | −14.631 | 55.369 | 10.607 | 0.706 | 0.255 | 0.024 |  |
| 8172914 | HUWE1 | HECT, UBA andWWE domain containing 1, E3 ubiquitin protein ligase | −97.177 | −5.965 | 91.212 | 14.571 | 0.640 | 0.255 | 0.024 |  |
| 7919095 | NOTCH2 | notch 2 | –200.416 | −56.072 | 144.343 | 11.342 | −6.154 | 0.256 | 0.023 |  |
| 8110382 | PRR7 | proline rich 7 (synaptic) | −8.991 | 2.579 | 11.570 | 11.397 | 8.920 | 0.256 | 0.025 |  |
| 7971386 | SNORA31 | small nucleolar RNA, H H/ACA box 31 | 3.794 | −3.702 | −7.496 | −10.714 | −9.970 | 0.256 | 0.025 |  |
| 8012887 | CDRT15 | CMT1A duplicated region transcript 15 | −0.669 | 6.889 | 7.558 | 14.905 | 7.538 | 0.257 | 0.025 |  |
| 7988093 | TP53BP1 | tumor protein p53 binding protein 1 | −15.231 | −0.019 | 15.213 | 13.081 | −1.820 | 0.257 | 0.025 |  |
| 8175256 | MGC16121 | MIR503 host gene | −5.029 | 2.768 | 7.797 | 11.641 | 4.148 | 0.257 | 0.025 |  |
| 8114767 | PCDH1 | protocadherin 1 | −4.032 | 4.171 | 8.203 | 10.509 | 8.718 | 0.258 | 0.025 |  |
| 8025058 | TRIP10 | thyroid hormone receptor interactor 10 | 5.047 | −6.307 | −11.354 | −16.757 | −7.632 | 0.258 | 0.025 |  |
| 7961279 | TAS2R14 | taste receptor, type 2, member 14 | 6.297 | −10.201 | −16.498 | −12.430 | −3.144 | 0.258 | 0.025 |  |
| 7919715 | ANP32E | acidic (leucine-rich) nuclear phosphoprotein 32 family, member E | 15.615 | −0.445 | −16.060 | −14.493 | −7.265 | 0.258 | 0.025 |  |
| 8007799 | MGC57346 | ADP-ribosylation factor pseudogene | 66.466 | −11.422 | −77.888 | −14.856 | −1.295 | 0.259 | 0.024 |  |
| 7949896 | OR7E87P | olfactory receptor, family 7, subfamily E, member 87 pseudogene | −1.029 | 20.059 | 21.088 | 18.745 | 3.408 | 0.259 | 0.025 |  |
| 8063814 | LSM14B | LSM family member 14B | 16.912 | −8.038 | −24.950 | −15.907 | −2.000 | 0.259 | 0.025 |  |
| 7989611 | FAM96A | family with sequence similarity 96, member A | 18.963 | −1.222 | −20.185 | −17.717 | −9.559 | 0.260 | 0.025 |  |
| 8151436 | PEX2 | peroxisomal biogenesis factor2 | 15.339 | 0.021 | −15.318 | −13.783 | −4.191 | 0.260 | 0.025 |  |
| 8042896 | TLX2 | T-cell leukemia homeobox 2 | −6.387 | 5.205 | 11.593 | 16.563 | 9.526 | 0.260 | 0.025 |  |
| 8146711 | C8orf44 | chromosome 8 open reading frame 44 | 21.946 | −3.290 | −25.236 | −14.344 | −8.340 | 0.260 | 0.025 |  |
| 8011375 | TRPV1 | transient receptor potential cation channel, subfamily V, member 1 | −10.979 | −2.182 | 8.797 | 10.253 | 4.983 | 0.261 | 0.025 |  |
| 8031732 | ZNF547 | zinc finger protein 547 | 9.143 | −1.050 | −10.193 | −13.417 | −4.523 | 0.261 | 0.025 |  |
| 8065230 | RBBP9 | retinoblastoma binding protein 9 | 4.482 | −4.844 | −9.326 | −11.116 | −6.932 | 0.261 | 0.025 |  |
| 8098084 | ETFDH | electron-transferring-flavoprotein dehydrogenase | 17.422 | −2.726 | −20.148 | −15.337 | −4.898 | 0.262 | 0.025 |  |
| 8131042 | FLJ44124 | uncharacterized LOC641737 | −4.000 | 1.871 | 5.870 | 11.345 | −3.582 | 0.262 | 0.025 |  |
| 8028194 | ZNF382 | zinc finger protein 382 | 20.677 | 2.828 | −17.850 | −17.878 | −6.989 | 0.263 | 0.025 |  |
| 8029353 | ZNF222 | zinc finger protein 222 | 6.017 | −2.964 | −8.982 | −15.826 | −2.806 | 0.263 | 0.025 |  |
| 7955637 | KRT18 | keratin 18 | 32.296 | −8.995 | −41.290 | −56.179 | 3.059 | 0.263 | 0.025 |  |
| 8072108 | ASPHD2 | aspartate beta-hydroxylase domain containing 2 | −12.013 | 5.552 | 17.565 | 13.363 | −1.362 | 0.263 | 0.025 |  |
| 8141283 | PTCD1 | pentatricopeptide repeat domain 1 | −7.381 | 3.034 | 10.415 | 11.234 | 5.884 | 0.263 | 0.025 |  |
| 8161499 | FAM27E3 | family with sequence similarity 27, member E3 | 10.320 | −6.409 | −16.729 | −17.389 | −0.953 | 0.264 | 0.025 |  |
| 8089659 | KIAA1407 | Coiled-coil domain containing 191 | 8.513 | −0.435 | −8.948 | −15.879 | −4.735 | 0.264 | 0.025 |  |
| 8040440 | GEN1 | GEN1 holiday junction 5′ flap endonuclease | 21.112 | 9.658 | −11.454 | −13.938 | 0.408 | 0.264 | 0.025 |  |
| 7978833 | RPL36AL | ribosomal protein L36a-like | 71.015 | −7.949 | −78.964 | −13.225 | 6.298 | 0.264 | 0.024 |  |
| 7913001 | UBR4 | ubiquitin protein ligase E3 component *N*-recognin 4 | –128.381 | −35.588 | 92.793 | 11.872 | −0.819 | 0.264 | 0.024 |  |
| 8027247 | ZNF93 | zinc finger protein 93 | 8.201 | −4.763 | −12.964 | −17.537 | −9.575 | 0.264 | 0.025 |  |
| 8149228 | LOC349196 | hypothetical LOC349196 | −9.097 | 5.453 | 14.550 | 10.427 | 9.311 | 0.264 | 0.025 |  |
| 8144488 | LOC349196 | hypothetical LOC349196 | −9.097 | 5.453 | 14.550 | 10.671 | −7.372 | 0.264 | 0.025 |  |
| 7999909 | GPRC5B | G protein-coupled receptor, class C, group 5, member B | 2.030 | 9.093 | 7.063 | 13.830 | −7.832 | 0.264 | 0.025 |  |
| 8051427 | FEZ2 | fasciculation and elongation protein zeta 2 (zygin II) | 14.134 | −4.235 | −18.369 | −13.898 | −7.846 | 0.265 | 0.025 |  |
| 8064686 | ADAM33 | ADAM metallopeptidase domain 33 | −4.629 | 2.623 | 7.251 | 10.234 | 3.858 | 0.265 | 0.025 |  |
| 7941501 | BANF1 | barrier to autointegration factor 1 | −6.596 | 11.581 | 18.177 | 11.090 | 5.639 | 0.265 | 0.025 |  |
| 8105111 | FBXO4 | F-box protein 4 | 6.484 | 1.174 | −5.310 | −10.669 | −7.506 | 0.265 | 0.025 |  |
| 8165610 | WDR85 | WD repeat domain 85 | −11.089 | −0.475 | 10.614 | 11.437 | 4.482 | 0.266 | 0.025 |  |
| 8132305 | EEPD1 | endonuclease/exonuclease/phosphatase family domain containing 1 | −3.854 | −35.675 | −31.821 | −11.895 | −7.832 | 0.267 | 0.024 |  |
| 8174717 | UPF3B | UPF3 regulator of nonsense transcripts homolog B (yeast) | 12.022 | −2.591 | −14.613 | −14.635 | −7.609 | 0.267 | 0.025 |  |
| 7974621 | ARID4A | AT rich interactive domain 4A (RBP1-like ) | 31.275 | −7.216 | −38.491 | −10.991 | −7.067 | 0.268 | 0.024 |  |
| 8082100 | PARP14 | poly (ADP-ribose) polymerase family, member 14 | –126.495 | 117.944 | 244.439 | 23.936 | 3.565 | 0.268 | 0.023 |  |
| 7910630 | C1orf31 | chromosome 1 open reading frame 31 | 6.196 | −2.539 | −8.735 | −13.021 | −9.799 | 0.268 | 0.025 |  |
| 8091432 | COMMD2 | COMM domain containing 2 | 35.249 | 0.629 | −34.620 | −13.354 | −3.969 | 0.268 | 0.025 |  |
| 8015821 | MEOX1 | mesenchyme homeobox 1 | −5.490 | 1.247 | 6.738 | 10.202 | 8.066 | 0.268 | 0.025 |  |
| 8175811 | FAM58A | family with sequence similarity 58, member A | −4.617 | 6.772 | 11.389 | 14.034 | −0.852 | 0.268 | 0.025 |  |
| 7979196 | GNPNAT1 | glucosamine-phosphate *N*-acetyltransferase 1 | 9.941 | −3.615 | −13.555 | −14.044 | −8.245 | 0.268 | 0.025 |  |
| 8082750 | UBA5 | ubiquitin-like modifier activating enzyme 5 | 19.812 | −0.937 | −20.750 | −14.673 | −5.019 | 0.269 | 0.025 |  |
| 8010405 | SLC26A11 | solute carrier family 26 (anion exchanger), member 11 | −21.751 | −4.573 | 17.179 | 11.822 | 6.819 | 0.269 | 0.025 |  |
| 7946142 | PRKCDBP | protein kinase C, delta binding protein | 5.797 | −7.514 | −13.311 | −22.393 | 5.183 | 0.269 | 0.025 |  |
| 7898582 | RPS14P3 | ribosomal protein S14 pseudogene 3 | 24.615 | 0.461 | −24.153 | −14.661 | 1.296 | 0.269 | 0.025 |  |
| 8107458 | COMMD10 | COMM domain containing 10 | 37.477 | 3.385 | −34.093 | −12.513 | −6.028 | 0.269 | 0.024 |  |
| 7902398 | SNORD45A | small nucleolar RNA, C/D box 45A | 231.831 | 34.425 | −197.406 | −12.451 | −5.520 | 0.269 | 0.022 |  |
| 8076223 | SNORD43 | small nucleolar RNA, C/D box 43 | 6.440 | −9.438 | −15.878 | −15.871 | 1.244 | 0.269 | 0.025 |  |
| 7957404 | C12orf26 | chromosome 12 open reading frame 26 | 21.456 | 1.401 | −20.055 | −11.252 | −9.856 | 0.269 | 0.025 |  |
| 7964262 | NACA | nascent polypeptide-associated complex alpha subunit | 39.109 | −32.512 | −71.621 | −10.573 | −3.031 | 0.270 | 0.024 |  |
| 8137448 | GALNT11 | polypeptide *N*-acetylgalactosaminyltransferase11 | −17.815 | 8.422 | 26.237 | 11.576 | 4.795 | 0.270 | 0.025 |  |
| 7974542 | C14orf101 | chromosome 14 open reading frame 101 | 49.044 | 3.280 | −45.764 | −10.697 | −2.168 | 0.270 | 0.024 |  |
| 8085340 | VGLL4 | vestigial-like family member 4 | −3.994 | 7.564 | 11.557 | 11.197 | 8.055 | 0.270 | 0.025 |  |
| 8046340 | DYNC1I2 | dynein, cytoplasmic 1, intermediate chain 2 | 2.924 | −7.607 | −10.531 | −12.090 | −4.399 | 0.271 | 0.025 |  |
| 8021542 | ZCCHC2 | zinc finger, CCHC domain containing 2 | −91.213 | −0.389 | 90.823 | 41.974 | 7.950 | 0.271 | 0.025 |  |
| 8010212 | TMC8 | transmembrane channel-like 8 | −62.575 | 3.647 | 66.221 | 12.424 | 6.946 | 0.271 | 0.024 |  |
| 7937020 | MKI67 | marker of proliferation Ki-67 | 5.205 | −4.476 | −9.681 | −13.215 | 1.231 | 0.271 | 0.025 |  |
| 8094688 | LIAS | lipoic acid synthetase | 13.707 | 0.615 | −13.092 | −14.328 | −9.775 | 0.271 | 0.025 |  |
| 8139832 | ZNF117 | zinc finger protein 117 | 64.680 | −17.387 | −82.067 | −19.472 | −6.854 | 0.273 | 0.024 |  |
| 8047272 | SPATS2L | spermatogenesis associated, serine-rich 2-like | −20.365 | 2.565 | 22.930 | 27.347 | 5.345 | 0.273 | 0.025 |  |
| 8156688 | TDRD7 | tudor domain containing 7 | −18.639 | 21.563 | 40.202 | 17.490 | 2.357 | 0.273 | 0.025 |  |
| 8116579 | FOXQ1 | fork headbox Q1 | −4.808 | 0.584 | 5.393 | 10.346 | 5.569 | 0.273 | 0.025 |  |
| 8031499 | SSC5D | scavenger receptor cysteine rich family, 5 domains | −4.150 | 3.531 | 7.681 | 12.638 | 3.520 | 0.274 | 0.025 |  |
| 8179224 | MUC21 | mucin 21, cell surface associated | −8.488 | 0.663 | 9.152 | 15.756 | 2.382 | 0.274 | 0.025 |  |
| 8038584 | LRRC4B | leucine rich repeat containing 4B | −4.414 | 2.631 | 7.045 | 12.916 | 0.618 | 0.274 | 0.025 |  |
| 8114511 | MGC29506 | Marginal zone B and B1 cell-specific protein | 28.830 | −10.655 | −39.486 | −45.576 | 2.915 | 0.274 | 0.025 |  |
| 8115410 | GEMIN5 | gem (nuclear organelle) associated protein 5 | −16.771 | 0.406 | 17.177 | 10.362 | 3.820 | 0.275 | 0.025 |  |
| 7940669 | C11orf83 | chromosome 11 open reading frame 83 | 8.173 | 0.671 | −7.502 | −11.481 | −6.629 | 0.275 | 0.025 |  |
| 7997542 | NECAB2 | *N*-terminal EF-hand calcium binding protein 2 | 9.032 | −0.409 | −9.441 | −13.533 | 0.813 | 0.275 | 0.025 |  |
| 7949227 | CDC42BPG | CDC42 binding protein kinase gamma (DMPK-like ) | −7.184 | 0.428 | 7.612 | 12.276 | 1.424 | 0.275 | 0.025 |  |
| 7913558 | LUZP1 | leucine zipper protein 1 | −8.749 | 7.495 | 16.244 | 12.912 | −1.870 | 0.275 | 0.025 |  |
| 8008646 | SCPEP1 | serine carboxypeptidase 1 | 23.044 | −37.579 | −60.623 | −10.913 | −4.556 | 0.276 | 0.024 |  |
| 8058509 | PLEKHM3 | pleckstrin homology domain containing, family M, member 3 | 12.110 | −4.759 | −16.869 | −10.043 | −2.957 | 0.276 | 0.025 |  |
| 8005726 | KCNJ12 | potassium channel, inwardly rectifying subfamily J, member 12 | −9.283 | −2.135 | 7.148 | 11.432 | 8.733 | 0.276 | 0.025 |  |
| 8130191 | RMND1 | required for meiotic nuclear division 1 homolog | 8.227 | 0.867 | −7.361 | −15.672 | −2.626 | 0.276 | 0.025 |  |
| 7898713 | CELA3B | chymotrypsin-like elastase family, member 3B | −6.530 | 0.873 | 7.403 | 10.493 | 9.670 | 0.276 | 0.025 |  |
| 8159732 | FLJ40292 | hypothetical LOC643210 | −29.400 | 1.265 | 30.666 | 11.516 | 4.802 | 0.276 | 0.025 |  |
| 7969145 | ARL11 | ADP-ribosylation factor-like 11 | 22.172 | −10.638 | −32.810 | −13.278 | −5.666 | 0.276 | 0.025 |  |
| 8019924 | MYL12A | myosin, light chain 12A, regulatory, non-sarcomeric | 40.160 | −8.133 | −48.294 | −10.660 | −4.251 | 0.276 | 0.024 |  |
| 7989037 | CCPG1 | cell cycle progression 1 | 38.462 | −3.497 | −41.959 | −12.102 | −9.011 | 0.277 | 0.024 |  |
| 7991893 | C16orf11 | chromosome 16 open reading frame 11 | −6.288 | 4.795 | 11.083 | 13.047 | 8.634 | 0.277 | 0.025 |  |
| 8012282 | LOC284023 | uncharaterized LOC284023 | 7.998 | 1.018 | −6.980 | −10.323 | −2.763 | 0.277 | 0.025 |  |
| 8043436 | IGKC | immunoglobulin kappa constant | 5.779 | −23.475 | −29.254 | −25.946 | 5.692 | 0.277 | 0.025 |  |
| 8161326 | ZNF658 | zinc finger protein 658 | 6.368 | −0.504 | −6.872 | −11.320 | 0.456 | 0.277 | 0.025 |  |
| 7989670 | RBPMS2 | RNA binding protein with multiple splicing 2 | −12.973 | −0.407 | 12.566 | 11.137 | 4.195 | 0.278 | 0.025 |  |
| 7910198 | ZNF678 | zinc finger protein 678 | 30.493 | 6.596 | −23.897 | −16.143 | −4.084 | 0.278 | 0.025 |  |
| 8058335 | SUMO1 | Small ubiquitin-like modifier 1 | 14.076 | 4.178 | −9.898 | −11.171 | 3.983 | 0.279 | 0.025 |  |
| 8165622 | ZMYND19 | zinc finger, MYND-type containing 19 | −15.478 | −3.537 | 11.942 | 10.870 | 7.417 | 0.279 | 0.025 |  |
| 8038904 | ZNF577 | zinc finger protein 577 | 21.089 | 1.211 | −19.878 | −14.698 | 2.526 | 0.279 | 0.025 |  |
| 7917561 | GBP4 | guanylate binding protein 4 | −34.948 | 42.880 | 77.828 | 20.302 | −3.123 | 0.279 | 0.024 |  |
| 8114709 | FCHSD1 | FCH and double SH3 domains 1 | −22.393 | 0.602 | 22.995 | 12.665 | 7.476 | 0.280 | 0.025 |  |
| 7959500 | HIP1R | huntingtin interacting protein 1 related | −10.007 | 1.237 | 11.245 | 11.455 | 9.639 | 0.281 | 0.025 |  |
| 8088958 | GBE1 | glucan (1,4-alpha-), branching enzyme 1 | 4.968 | −12.103 | −17.070 | −11.434 | −8.051 | 0.281 | 0.025 |  |
| 8177478 | RAD17 | RAD17 checkpoint clampu loader component | 25.121 | 5.753 | −19.368 | −17.450 | −8.084 | 0.281 | 0.025 |  |
| 8166140 | MOSPD2 | motile sperm domain containing 2 | 69.406 | 0.377 | −69.029 | −10.179 | −1.429 | 0.281 | 0.024 |  |
| 8047228 | MOBKL3 | MOB family member 4, phocein | 23.573 | −3.599 | −27.172 | −11.538 | −7.304 | 0.281 | 0.025 |  |
| 7901460 | GPX7 | glutathione peroxidase 7 | 28.332 | −4.260 | −32.592 | −12.354 | −3.252 | 0.282 | 0.024 |  |
| 8054308 | TBC1D8 | TBC1 domain family, member 8 (with GRAM domain) | −27.302 | −3.658 | 23.645 | 13.439 | −0.531 | 0.282 | 0.025 |  |
| 7982287 | ARHGAP11B | Rho GTPase activating protein 11B | 1.289 | −15.267 | −16.555 | −21.637 | −5.759 | 0.282 | 0.025 |  |
| 7944262 | MLL | Myloid/lymphoid or mixed-lineage leukemia (trithorax homolog, drosophila) | −83.979 | 7.550 | 91.528 | 60.572 | 8.545 | 0.282 | 0.025 |  |
| 8159354 | EGFL7 | EGF-like-domain, multiple 7 | −2.122 | 6.453 | 8.575 | 10.431 | 5.621 | 0.282 | 0.025 |  |
| 7937728 | TNNI2 | troponin I type 2 (skeletal, fast) | −0.077 | −7.581 | −7.504 | −10.860 | −2.251 | 0.282 | 0.025 |  |
| 7997520 | HSBP1 | heat shock factor binding protein 1 | 18.208 | −2.837 | −21.044 | −13.938 | −9.039 | 0.282 | 0.025 |  |
| 8016909 | MKS1 | Meckel syndrome, type 1 | 3.620 | −6.135 | −9.755 | −14.768 | −5.740 | 0.282 | 0.025 |  |
| 8116807 | SNRNP48 | small nuclear ribonucleoprotein 48kDa (U11/U12) | 25.103 | 3.807 | −21.295 | −10.939 | −2.234 | 0.282 | 0.025 |  |
| 8022326 | MPPE1 | metallophosphoesterase 1 | 9.946 | −26.257 | −36.203 | −13.377 | −9.146 | 0.283 | 0.024 |  |
| 8093141 | WDR53 | WD repeat domain 53 | 8.276 | −3.939 | −12.215 | −11.327 | −0.145 | 0.284 | 0.025 |  |
| 8053882 | DUSP2 | dual specificity phosphatase 2 | −6.613 | 4.238 | 10.851 | 13.540 | 2.632 | 0.284 | 0.025 |  |
| 7975598 | ACOT1 | acyl-CoA thioesterase 1 | −7.511 | 1.138 | 8.650 | 12.085 | 2.824 | 0.284 | 0.025 |  |
| 8150276 | PPAPDC1B | Phospholipid phosphatase 5 | 21.478 | −12.093 | −33.570 | −11.698 | 5.088 | 0.284 | 0.024 |  |
| 7946149 | APBB1 | amyloid beta (A4) precursor protein -binding, family B, member 1(Fe65) | −3.272 | 10.098 | 13.370 | 12.817 | 7.861 | 0.284 | 0.025 |  |
| 8059067 | IHH | Indian hedgehog | 5.818 | 0.706 | −5.111 | −10.477 | 7.053 | 0.284 | 0.025 |  |
| 7910369 | HIST3H2BB | histone cluster 3, H2bb | 1.406 | −5.986 | −7.392 | −11.425 | −7.271 | 0.284 | 0.025 |  |
| 8152053 | ANKRD46 | ankyrin repeat domain 46 | 12.540 | 0.113 | −12.427 | −12.188 | 1.084 | 0.285 | 0.025 |  |
| 8059801 | USP40 | ubiquitin specific peptidase 40 | −5.910 | 2.794 | 8.705 | 13.043 | 0.935 | 0.285 | 0.025 |  |
| 8156523 | MIRLET7D | microRNA let-7d | 8.249 | −4.648 | −12.897 | −15.513 | −6.296 | 0.286 | 0.025 |  |
| 8056716 | METTL5 | methyltransferase like 5 | 15.721 | −0.926 | −16.646 | −12.865 | −5.485 | 0.287 | 0.025 |  |
| 8035050 | RASAL3 | RAS protein activator like 3 | −46.495 | −1.353 | 45.142 | 10.642 | 5.723 | 0.287 | 0.024 |  |
| 7981266 | CCDC85C | coiled-coil domain containing 85C | −4.109 | 4.215 | 8.324 | 12.527 | 1.105 | 0.287 | 0.025 |  |
| 8077731 | FANCD2 | Fanconi anemia, complementation group D2 | 11.185 | −1.107 | −12.292 | −12.240 | −2.455 | 0.287 | 0.025 |  |
| 8161319 | ZNF658 | zinc finger protein 658 | 6.536 | −0.377 | −6.913 | −13.210 | −1.132 | 0.287 | 0.025 |  |
| 7906742 | PCP4L1 | Purkinje cell protein 4 like 1 | −6.418 | 0.352 | 6.770 | 10.046 | 7.277 | 0.288 | 0.025 |  |
| 8164002 | ZBTB26 | zinc finger and BTB domain containing 26 | 10.856 | −0.808 | −11.665 | −10.144 | −3.333 | 0.288 | 0.025 |  |
| 7949275 | MIR194-2 | microRNA 194-2 | −6.021 | 3.189 | 9.210 | 12.025 | 9.874 | 0.288 | 0.025 |  |
| 8057554 | CACYBP | calcyclin binding protein | 26.783 | 1.730 | −25.053 | −10.452 | 0.472 | 0.288 | 0.025 |  |
| 8147012 | PKIA | protein kinase (CAMP-dependent, catalytic) inhibitor alpha | 4.889 | 13.569 | 8.681 | 10.500 | 1.758 | 0.288 | 0.025 |  |
| 7991695 | GOLGA8DP | golgin A8 family, member D, pseudogene | −6.724 | 2.394 | 9.117 | 13.828 | 5.770 | 0.289 | 0.025 |  |
| 8155794 | C9orf85 | chromosome 9 open reading frame 85 | 11.829 | −3.319 | −15.148 | −14.925 | 1.922 | 0.290 | 0.025 |  |
| 7983132 | STARD9 | StAR-related lipid transfer (START) domain containing 9 | −5.081 | 1.773 | 6.854 | 10.510 | −1.682 | 0.290 | 0.025 |  |
| 8098177 | KLHL2 | kelch-like family member 2 | 59.268 | 4.674 | −54.594 | −15.672 | −0.714 | 0.290 | 0.024 |  |
| 7898371 | LOC644634 | UPF0627 protein ENSP00000358171-like | −4.764 | 7.418 | 12.182 | 12.868 | 4.598 | 0.290 | 0.025 |  |
| 7912861 | LOC644634 | UPF0627 protein ENSP00000358171-like | −4.764 | 7.418 | 12.182 | 12.868 | 4.598 | 0.290 | 0.025 |  |
| 8043512 | ZNF2 | zinc finger protein 2 | 4.495 | −4.025 | −8.519 | −11.900 | −6.388 | 0.291 | 0.025 |  |
| 7994609 | QPRT | quinolinate phosphoribosyltransferase | −7.049 | 1.701 | 8.750 | 11.255 | 7.501 | 0.292 | 0.025 |  |
| 8101637 | HSD17B13 | hydroxysteroid (17-beta) dehydrogenase 13 | 6.693 | −5.482 | −12.175 | −20.780 | −3.007 | 0.292 | 0.025 |  |
| 8001197 | NETO2 | neuropilin (NRP) and tolloid (TLL)-like 2 | 8.329 | −4.124 | −12.453 | −13.097 | −4.717 | 0.292 | 0.025 |  |
| 7915245 | HPCAL4 | hippocalcin like 4 | 6.335 | −0.093 | −6.428 | −13.534 | −6.285 | 0.292 | 0.025 |  |
| 8014066 | EVI2A | ecotropic viral integration site 2A | 82.441 | 14.485 | −67.955 | −18.635 | −8.162 | 0.293 | 0.024 |  |
| 8036395 | ZNF569 | zinc finger protein 569 | 4.272 | −0.921 | −5.193 | −10.167 | 3.221 | 0.293 | 0.025 |  |
| 8018652 | RNF157 | ring finger protein 157 | −31.453 | 8.096 | 39.549 | 11.066 | 9.809 | 0.293 | 0.024 |  |
| 7936408 | DCLRE1A | DNA cross-link repair 1A | 2.739 | −5.784 | −8.524 | −17.727 | −6.124 | 0.293 | 0.025 |  |
| 8091757 | TRIM59 | tripartite motif-containing 59 | 14.879 | 0.258 | −14.620 | −14.882 | −6.688 | 0.294 | 0.025 |  |
| 8059350 | AP1S3 | adaptor-related protein complex 1, sigma 3 subunit | 9.276 | −1.116 | −10.392 | −17.204 | −3.919 | 0.294 | 0.025 |  |
| 8038225 | PLEKHA4 | pleckstrin homology domain containing, family A (phosphoinositide binding specific) member 4 | −2.415 | 4.508 | 6.922 | 10.750 | 4.141 | 0.294 | 0.025 |  |
| 8027685 | ZNF181 | zinc finger protein 181 | 8.941 | 1.178 | −7.763 | −12.056 | −1.078 | 0.294 | 0.025 |  |
| 8130383 | MTRF1L | mitochondrial translational release factor 1-like | 19.709 | 6.399 | −13.310 | −10.101 | −3.293 | 0.294 | 0.025 |  |
| 8007115 | KRTAP4-9 | keratin associated protein 4-9 | −8.656 | 5.689 | 14.345 | 10.017 | 6.144 | 0.295 | 0.025 |  |
| 7965486 | CCDC41 | coiled-coil domain containing 41 | 6.748 | −1.501 | −8.249 | −14.874 | −5.332 | 0.295 | 0.025 |  |
| 8126860 | MUT | methylmalonyl CoA mutase | 18.421 | −1.448 | −19.869 | −11.784 | −5.524 | 0.296 | 0.025 |  |
| 8158560 | TOR1B | torsin family 1, member B (torsin B) | −62.268 | 4.203 | 66.471 | 20.453 | 3.907 | 0.296 | 0.024 |  |
| 8077663 | JAGN1 | jagunal homolog 1 | 23.016 | −2.332 | −25.348 | −10.429 | −1.942 | 0.296 | 0.025 |  |
| 8167601 | USP27X | ubiquitin specific peptidase 27, X-linked | −10.264 | 1.276 | 11.540 | 12.618 | 5.134 | 0.296 | 0.025 |  |
| 7905043 | LOC388692 | uncharaterized LOC388692 | −14.661 | 27.332 | 41.993 | 11.596 | −5.956 | 0.297 | 0.024 |  |
| 7935462 | EXOSC1 | exosome component 1 | 35.983 | 1.953 | −34.030 | −10.032 | −3.027 | 0.297 | 0.024 |  |
| 8069561 | BTF3L4 | basic transcription factor 3-like 4 | 49.801 | −6.987 | −56.788 | −21.462 | −1.790 | 0.297 | 0.024 |  |
| 8131949 | CBX3 | chromobox homolog 3 | 34.059 | 5.672 | −28.387 | −14.470 | −7.558 | 0.298 | 0.025 |  |
| 8092000 | TERC | telomeraseRNA component | 13.788 | −2.047 | −15.835 | −15.561 | −2.894 | 0.298 | 0.025 |  |
| 7922404 | SNORD80 | small nucleolar RNA, C/D box 80 | 21.788 | −3.422 | −25.210 | −14.253 | 0.691 | 0.299 | 0.025 |  |
| 8004184 | XAF1 | XIAP associated factor 1 | –109.059 | 48.035 | 157.095 | 28.189 | 2.461 | 0.299 | 0.024 |  |
| 7896817 | ISG15 | ISG15 ubiquitin-like modifier | −6.206 | 0.380 | 6.586 | 12.587 | 2.670 | 0.299 | 0.025 |  |
| 8035808 | ZNF100 | zinc finger protein 100 | 23.063 | −5.916 | −28.979 | −23.333 | 4.618 | 0.299 | 0.025 |  |
| 7973468 | LRRC16B | leucine rich repeat containing 16B | −3.891 | 2.794 | 6.685 | 13.521 | 5.260 | 0.299 | 0.025 |  |
| 7902495 | NEXN | nexilin (F actin binding protein ) | −5.975 | 4.938 | 10.912 | 21.094 | 7.261 | 0.299 | 0.025 |  |
| 7901102 | MMACHC | methylmalonic aciduria (cobalamin deficiency) cblC type, with homocystinuria | 6.024 | −1.927 | −7.951 | −10.167 | −4.437 | 0.300 | 0.025 |  |
| 8066266 | MAFB | V-Maf musculoaponeurotic fibrosarcoma oncogene homolog B | −28.910 | −2.892 | 26.018 | 14.991 | −2.564 | 0.300 | 0.025 |  |
| 8097717 | ARHGAP10 | Rho GTPase activating protein 10 | 8.770 | 0.210 | −8.561 | −13.297 | −1.794 | 0.301 | 0.025 |  |
| 8019711 | SCGB1C1 | secretoglobin, family 1C, member 1 | −5.775 | 1.529 | 7.304 | 10.870 | 5.788 | 0.301 | 0.025 |  |
| 7901557 | DMRTB1 | DMRT-like family B with proline-rich *C*-terminal, 1 | −4.502 | 2.124 | 6.626 | 10.458 | 9.082 | 0.301 | 0.025 |  |
| 8101723 | NAP1L5 | nucleosome assembly protein 1-like 5 | 2.636 | 10.901 | 8.265 | 14.111 | 8.772 | 0.301 | 0.025 |  |
| 8173189 | SPIN2B | spindlin family, member 2B | 1.823 | −5.219 | −7.042 | −10.666 | −7.875 | 0.302 | 0.025 |  |
| 8033462 | CTXN1 | cortexin 1 | −6.174 | 3.529 | 9.703 | 11.817 | 3.682 | 0.302 | 0.025 |  |
| 8029399 | ZNF226 | zinc finger protein 226 | 5.096 | −7.558 | −12.654 | −13.231 | −1.609 | 0.303 | 0.025 |  |
| 7949444 | KCNK7 | potassium channel, two pore domain subfamily K, member 7 | −0.565 | −6.219 | −5.654 | −10.208 | 2.129 | 0.303 | 0.025 |  |
| 8012598 | RCVRN | Recoverin | 4.973 | −6.421 | −11.395 | −20.208 | −3.834 | 0.304 | 0.025 |  |
| 8101013 | RCHY1 | ring finger and CHY zinc finger domain containing 1, E3 ubiquitin protein ligase | 11.433 | −0.839 | −12.272 | −10.032 | −5.821 | 0.304 | 0.025 |  |
| 7915078 | EPHA10 | EPH receptor A10 | −4.807 | 1.946 | 6.753 | 12.867 | 7.752 | 0.304 | 0.025 |  |
| 8065410 | CST4 | cystatin S | −10.533 | −2.231 | 8.302 | 15.588 | 1.551 | 0.304 | 0.025 |  |
| 8110392 | TMED9 | transmembrane P24 trafficking protein 9 | 9.380 | −13.211 | −22.591 | −11.355 | 6.053 | 0.304 | 0.025 |  |
| 7978739 | TRAPPC6B | trafficking protein particle complex 6B | 22.991 | 2.859 | −20.132 | −11.218 | −1.743 | 0.305 | 0.025 |  |
| 8024885 | TNFAIP8L1 | tumor necrosis factor, alpha-induced protein 8-like 1 | 8.172 | −3.522 | −11.694 | −12.381 | −6.256 | 0.305 | 0.025 |  |
| 8035304 | BST2 | bone marrow stromal cell antigen 2 | −12.032 | 10.935 | 22.966 | 12.475 | −0.711 | 0.305 | 0.025 |  |
| 7900454 | DEM1 | exonuclease 5 | 8.517 | −3.911 | −12.428 | −14.792 | −3.785 | 0.306 | 0.025 |  |
| 8134834 | AGFG2 | ArfGAP with FG repeats 2 | −13.257 | −0.757 | 12.500 | 10.026 | 5.844 | 0.307 | 0.025 |  |
| 8004842 | ARHGEF15 | Rho guanine nucleotide exchange factor (GEF) 15 | −1.254 | 4.427 | 5.680 | 10.686 | 6.057 | 0.307 | 0.025 |  |
| 7968703 | NHLRC3 | NHL repeat containing 3 | 8.735 | −8.344 | −17.079 | −11.878 | −6.755 | 0.307 | 0.025 |  |
| 8042259 | MDH1 | malate dehydrogenase 1, NAD (soluble) | 15.712 | 1.264 | −14.448 | −11.405 | −0.810 | 0.307 | 0.025 |  |
| 8165658 | ND2 | mitochondrially encoded NADH dehydrogenase 2 | −60.023 | 11.762 | 71.786 | 11.135 | 4.888 | 0.307 | 0.024 |  |
| 8009602 | KIF19 | kinesin family member 19 | −0.264 | 5.333 | 5.597 | 12.005 | 1.863 | 0.307 | 0.025 |  |
| 8154962 | DNAJB5 | DnaJ (Hsp40) homolog, subfamily B, member 5 | 10.142 | −1.909 | −12.051 | −11.697 | 0.390 | 0.307 | 0.025 |  |
| 8031744 | ZNF17 | zinc finger protein 17 | 6.698 | −4.453 | −11.151 | −11.153 | −5.599 | 0.307 | 0.025 |  |
| 7914232 | TMEM200B | transmembrane protein 200B | −8.178 | 1.982 | 10.160 | 11.274 | 9.093 | 0.308 | 0.025 |  |
| 8077103 | TYMP | thymidine phosphorylase | −58.659 | 13.130 | 71.790 | 11.874 | 2.805 | 0.308 | 0.024 |  |
| 8058552 | IDH1 | isocitrate dehydrogenase 1 (NADP+), soluble | 21.994 | −11.214 | −33.208 | −11.710 | −0.978 | 0.308 | 0.024 |  |
| 7954481 | SSPN | sarcospan | 16.607 | 2.328 | −14.279 | −17.093 | 6.793 | 0.308 | 0.025 |  |
| 8068200 | C21orf119 | chromosome 21 open reading frame 119 | 8.949 | 2.005 | −6.945 | −12.253 | 1.696 | 0.309 | 0.025 |  |
| 7905544 | SPRR1A | small proline-rich protein 1A | 1.737 | 8.661 | 6.924 | 10.201 | 3.185 | 0.309 | 0.025 |  |
| 8141180 | TECPR1 | tectonin beta-propeller repeat containing 1 | −12.529 | −0.503 | 12.027 | 11.295 | 2.625 | 0.309 | 0.025 |  |
| 7911343 | UIMC1 | ubiquitin interaction motif containing 1 | −29.883 | −2.618 | 27.265 | 14.174 | 7.920 | 0.309 | 0.025 |  |
| 8165703 | UIMC1 | ubiquitin interaction motif containing 1 | −29.883 | −2.618 | 27.265 | 14.174 | 7.920 | 0.309 | 0.025 |  |
| 8071676 | RAB36 | RAB36, member RAS oncogene family | 9.381 | −3.120 | −12.500 | −12.300 | 1.726 | 0.310 | 0.025 |  |
| 8007446 | IFI35 | interferon-induced protein 35 | −41.365 | 50.253 | 91.618 | 22.564 | 5.170 | 0.310 | 0.024 |  |
| 7948176 | TNKS1BP1 | tankyrase1 binding protein 1, 182kDa | −6.856 | 0.173 | 7.029 | 10.140 | 7.703 | 0.310 | 0.025 |  |
| 8032478 | LINGO3 | leucine rich repeat and Ig domain containing 3 | −12.582 | 1.861 | 14.443 | 16.113 | 3.297 | 0.310 | 0.025 |  |
| 7900354 | PPIE | peptidylprolyl isomerase E (cyclophilin E) | 16.421 | −2.959 | −19.380 | −12.432 | 5.132 | 0.310 | 0.025 |  |
| 7931951 | SFMBT2 | Scm-like with four Mbt domains 2 | −21.235 | 13.094 | 34.329 | 11.165 | 0.527 | 0.311 | 0.024 |  |
| 7915995 | FAF1 | Fas (TNFRSF6) associated factor 1 | 27.688 | −4.248 | −31.937 | −12.905 | −6.013 | 0.311 | 0.025 |  |
| 8075616 | SYN3 | synapsin III | −6.139 | 0.435 | 6.574 | 11.672 | 9.389 | 0.311 | 0.025 |  |
| 8161242 | EXOSC3 | exosome component 3 | 25.883 | −4.485 | −30.368 | −12.513 | −2.610 | 0.311 | 0.025 |  |
| 7975926 | KIAA1737 | CLOCK-interacting pacemaker | 13.068 | −3.768 | −16.836 | −10.276 | −1.203 | 0.312 | 0.025 |  |
| 8161346 | ZNF658 | zinc finger protein 658 | 7.229 | −0.122 | −7.351 | −14.715 | −1.415 | 0.312 | 0.025 |  |
| 8082075 | DTX3L | deltex 3 like, E3 ubiquitin ligase | −47.846 | 65.702 | 113.548 | 15.493 | 3.945 | 0.312 | 0.024 |  |
| 7907213 | GORAB | golgin, RAB6-interacting | 12.197 | −1.884 | −14.080 | −12.967 | 9.240 | 0.312 | 0.025 |  |
| 7980390 | C14orf148 | chromosome 14 open reading frame 148 | 4.421 | −3.491 | −7.912 | −12.777 | −3.897 | 0.313 | 0.025 |  |
| 7940824 | NAA40 | N(alpha)-acetyltransferase 40, NatD catalytic subunit | −21.393 | 7.141 | 28.535 | 13.562 | 9.552 | 0.314 | 0.025 |  |
| 8108006 | LEAP2 | liver expressed antimicrobial peptide 2 | −2.750 | 3.782 | 6.532 | 11.223 | 6.151 | 0.314 | 0.025 |  |
| 8126524 | C6orf108 | chromosome 6 open reading frame 108 | 15.323 | −3.461 | −18.785 | −11.350 | 1.005 | 0.314 | 0.025 |  |
| 8160295 | SCARNA8 | small Cajal body-specific RNA 8 | 10.192 | −0.546 | −10.738 | −15.474 | −8.467 | 0.314 | 0.025 |  |
| 8005733 | C20orf191 | nuclear receptor co-repressor 1 pseudogene | −20.279 | 3.802 | 24.082 | 11.689 | −5.544 | 0.314 | 0.025 |  |
| 8124406 | HIST1H2BC | histone cluster 1, H2bc | 54.579 | 12.319 | −42.260 | −11.422 | −5.464 | 0.314 | 0.024 |  |
| 8028200 | ZNF567 | zinc finger protein 567 | 4.422 | −2.444 | −6.866 | −11.608 | −4.340 | 0.314 | 0.025 |  |
| 7971565 | LPAR6 | lysophosphatidic acid receptor 6 | 33.260 | −0.896 | −34.156 | −18.120 | 6.520 | 0.315 | 0.025 |  |
| 8148615 | ZNF696 | zinc finger protein 696 | 1.658 | −6.424 | −8.083 | −11.574 | −7.591 | 0.315 | 0.025 |  |
| 7956301 | LRP1 | low density lipoprotein receptor-related protein 1 | –127.957 | −58.039 | 69.918 | 21.380 | −1.028 | 0.315 | 0.024 |  |
| 8106660 | RASGRF2 | Ras protein–specific guanine nucleotide-releasing factor 2 | −9.690 | 28.643 | 38.333 | 14.088 | 0.907 | 0.315 | 0.024 |  |
| 8160559 | DDX58 | DEAD (Asp-Glu-Ala-Asp) box polypeptide 58 | −58.488 | 81.032 | 139.520 | 22.923 | 5.987 | 0.316 | 0.024 |  |
| 7979437 | C14orf149 | chromosome 14 open reading frame 149 | 6.229 | −4.534 | −10.763 | −10.645 | 0.734 | 0.316 | 0.025 |  |
| 8173373 | PDZD11 | PDZ domain containing 11 | 1.783 | −4.593 | −6.376 | −10.370 | −0.137 | 0.316 | 0.025 |  |
| 8174026 | YWHAQP8 | YWHAQ pseudogene 8 | 13.270 | −2.860 | −16.130 | −10.882 | −5.314 | 0.316 | 0.025 |  |
| 8153359 | LY6D | lymphocyte antigen 6 complex, locus D | −9.915 | −0.426 | 9.488 | 13.969 | 5.362 | 0.317 | 0.025 |  |
| 8030899 | ZNF766 | zinc finger protein 766 | 16.240 | 0.830 | −15.411 | −12.813 | −8.415 | 0.317 | 0.025 |  |
| 7898736 | HSPC157 | long intergenic non-protein coding RNA 339 | 7.577 | −5.727 | −13.304 | −18.543 | 0.189 | 0.317 | 0.025 |  |
| 7909628 | FLVCR1 | feline leukemia virus subgroup C cellular receptor 1 | 31.338 | 4.753 | −26.585 | −10.519 | 9.543 | 0.317 | 0.025 |  |
| 7898549 | MRTO4 | MRT4 homolog, ribosome maturation factor | 10.331 | 0.586 | −9.745 | −14.828 | −4.786 | 0.317 | 0.025 |  |
| 7927202 | ZNF22 | zinc finger protein 22 | 10.959 | −1.949 | −12.908 | −11.600 | 7.042 | 0.318 | 0.025 |  |
| 7970810 | SLC7A1 | solute carrier family 7(cationic amino acid transporter, Y+ system), member 1 | −12.732 | 19.303 | 32.035 | 17.675 | −5.460 | 0.318 | 0.025 |  |
| 7983123 | HAUS2 | HAUS augmin-like complex, subunit2 | 13.177 | −4.323 | −17.500 | −12.291 | −2.696 | 0.318 | 0.025 |  |
| 8169742 | XIAP | X-linked inhibitor of apoptosis, E3 protein ligase | 18.681 | 0.822 | −17.859 | −11.596 | −6.509 | 0.319 | 0.025 |  |
| 7970844 | KATNAL1 | katanin P60 subunit A-like 1 | 7.269 | −1.825 | −9.094 | −15.732 | 1.901 | 0.319 | 0.025 |  |
| 8124459 | ZNF322A | zinc finger protein 322A | 6.524 | −0.201 | −6.726 | −10.348 | 0.697 | 0.319 | 0.025 |  |
| 7970999 | SPG20 | spastic paraplegia 20 (Troyer syndrome) | 10.741 | −4.374 | −15.115 | −11.102 | −9.324 | 0.319 | 0.025 |  |
| 7981242 | BCL11B | B-cell CLL/lymphoma 11B (zinc finger protein) | −50.148 | 2.650 | 52.798 | 12.984 | 4.623 | 0.320 | 0.024 |  |
| 8025506 | PIN1 | peptidylprolyl cis/trans isomerase, NIMA-interacting 1 | 21.325 | −9.801 | −31.126 | −10.817 | −1.359 | 0.320 | 0.024 |  |
| 8106976 | GPR150 | G protein-coupled receptor 150 | −5.585 | 3.086 | 8.671 | 10.724 | 9.573 | 0.320 | 0.025 |  |
| 7911634 | TMEM52 | transmembrane protein 52 | −3.218 | 5.720 | 8.938 | 10.935 | 6.268 | 0.320 | 0.025 |  |
| 7969228 | ALG11 | ALG11, alpha-1,2-mannosyltransferase | 24.481 | 4.624 | −19.857 | −10.609 | −6.758 | 0.320 | 0.025 |  |
| 7942409 | P2RY6 | pyrimidinergic receptor P2Y, G- protein coupled, 6 | −3.420 | 2.109 | 5.529 | 10.061 | 6.880 | 0.320 | 0.025 |  |
| 8093386 | MYL5 | myosin, light chain 5, regulatory | 4.048 | −1.462 | −5.510 | −11.052 | −1.149 | 0.320 | 0.025 |  |
| 8171708 | CXorf23 | chromosome X open reading frame 23 | 16.001 | 2.545 | −13.456 | −10.532 | −5.979 | 0.320 | 0.025 |  |
| 7929243 | NHP2 | NHP2 ribonucleoprotein | 13.775 | 1.025 | −12.750 | −10.553 | 2.034 | 0.322 | 0.025 |  |
| 8053801 | ANKRD36B | ankyrin repeat domain 36B | 161.826 | 26.283 | −135.543 | −13.561 | −5.189 | 0.322 | 0.023 |  |
| 8047038 | PMS1 | PMS1 homolog 1, mismatch repair system component | 26.892 | 2.683 | −24.209 | −14.158 | −3.557 | 0.322 | 0.025 |  |
| 7918569 | ST7L | suppression of tumorigenicity 7 like | 7.222 | −0.460 | −7.682 | −12.531 | −7.659 | 0.322 | 0.025 |  |
| 7935983 | PITX3 | paired-like homeodomain 3 | −10.075 | −1.025 | 9.051 | 13.875 | 7.540 | 0.323 | 0.025 |  |
| 8022251 | PPP4R1 | protein phosphatase 4, regulatory subunit 1 | 73.859 | −21.882 | −95.741 | −12.095 | −8.428 | 0.323 | 0.024 |  |
| 8030582 | MYH14 | myosin, heavy chain 14, non-muscle | −0.267 | 5.184 | 5.451 | 10.186 | 8.489 | 0.323 | 0.025 |  |
| 7912250 | MIR34A | microRNA 34a | −5.104 | 1.856 | 6.960 | 11.299 | 7.148 | 0.323 | 0.025 |  |
| 8103769 | HPGD | hydroxyprostaglandin dehydrogenase 15-(NAD) | 19.655 | 3.690 | −15.965 | −21.287 | 6.847 | 0.323 | 0.025 |  |
| 7947027 | UEVLD | UEV and lactate/malate dehydrogenase domains | 17.891 | −3.472 | −21.362 | −10.146 | −4.521 | 0.324 | 0.025 |  |
| 8138776 | HIBADH | 3-hydroxyisobutyrate dehydrogenase | 26.684 | 10.686 | −15.998 | −10.757 | −2.799 | 0.324 | 0.025 |  |
| 8095697 | CXCL1 | chemokine (C-X-Cmotif) ligand 1 (melanoma growth stimulating activity, alpha) | 26.134 | −2.859 | −28.993 | −15.407 | −3.425 | 0.324 | 0.025 |  |
| 8143684 | PDIA4 | protein disulfide isomerase family A, member 4 | 62.535 | −14.745 | −77.279 | −23.629 | 2.684 | 0.324 | 0.024 |  |
| 7908966 | NCRNA00260 | Long intergenic non-protein coding RNA 260 | −16.175 | 4.420 | 20.595 | 16.158 | 1.278 | 0.325 | 0.025 |  |
| 7945462 | IRF7 | interferon regulatory factor 7 | −22.062 | 1.501 | 23.563 | 11.830 | 4.528 | 0.325 | 0.025 |  |
| 8131927 | MPP6 | membrane protein, palmitoylated 6 (MAGUK P55 subfamily member 6) | 18.341 | 4.026 | −14.315 | −13.374 | −7.078 | 0.325 | 0.025 |  |
| 8037502 | NKPD1 | NTPase, KAP family P-loop domain containing 1 | −3.615 | 1.832 | 5.448 | 10.356 | 1.835 | 0.325 | 0.025 |  |
| 7958158 | HCFC2 | host cell factor C2 | 9.918 | −3.948 | −13.867 | −10.137 | −6.507 | 0.326 | 0.025 |  |
| 8053496 | POLR1A | polymerase (RNA) I (DNA directed) polypeptide A, 194kDa | −22.485 | −4.332 | 18.153 | 10.163 | −0.699 | 0.326 | 0.025 |  |
| 8119124 | PI16 | peptidase inhibitor 16 | −5.810 | 7.165 | 12.976 | 11.175 | −1.282 | 0.326 | 0.025 |  |
| 8040655 | EPT1 | ethanolaminephosphotransferase 1 | 10.372 | −0.956 | −11.328 | −10.864 | −3.159 | 0.326 | 0.025 |  |
| 7946589 | MRVI1 | murine retrovirus integration site1 homolog | 10.174 | 0.243 | −9.931 | −11.140 | −8.935 | 0.327 | 0.025 |  |
| 8079189 | ZNF167 | zinc finger protein 167 | 8.470 | 0.003 | −8.467 | −11.219 | 1.924 | 0.327 | 0.025 |  |
| 8172225 | EFHC2 | EF-hand domain (*C*-terminal) containing 2 | 16.069 | −1.733 | −17.802 | −21.021 | −8.608 | 0.327 | 0.025 |  |
| 7944006 | RBM7 | RNA binding motif protein 7 | 11.456 | −5.548 | −17.005 | −10.096 | 0.542 | 0.328 | 0.025 |  |
| 7914516 | MTMR9LP | myotubularin related protein 9-like, pseudogene | −4.782 | 5.052 | 9.834 | 12.898 | 3.973 | 0.328 | 0.025 |  |
| 8026007 | ZNF791 | zinc finger protein 791 | 68.656 | 7.203 | −61.452 | −13.355 | 0.922 | 0.329 | 0.024 |  |
| 8071547 | UBE2L3 | ubiquitin-conjugating enzyme E2L 3 | 105.930 | 44.487 | −61.444 | −18.256 | −1.640 | 0.329 | 0.024 |  |
| 8117773 | LOC554223 | histocompatibility antigen-related | −7.168 | 6.648 | 13.816 | 16.045 | 7.221 | 0.330 | 0.025 |  |
| 8117402 | HIST1H4E | histone cluster 1, H4e | 49.306 | 3.701 | −45.606 | −11.164 | −5.087 | 0.330 | 0.024 |  |
| 7954460 | LYRM5 | LYR motif containing 5 | 22.595 | −5.311 | −27.906 | −13.475 | −4.237 | 0.330 | 0.025 |  |
| 7934870 | ATAD1 | ATPase family, AAA domain containing 1 | 22.629 | 0.005 | −22.624 | −10.365 | −2.062 | 0.330 | 0.025 |  |
| 8149629 | GFRA2 | GDNF family receptor alpha 2 | −10.597 | −1.709 | 8.888 | 16.602 | 2.250 | 0.331 | 0.025 |  |
| 7911301 | PGBD2 | piggyBac transposable element derived 2 | 2.339 | −7.868 | −10.207 | −13.853 | −5.487 | 0.331 | 0.025 |  |
| 7963567 | KRT8 | keratin 8, type II | 5.425 | −3.713 | −9.138 | −10.564 | −3.021 | 0.332 | 0.025 |  |
| 8162586 | SLC35D2 | solute carrier family 35 (UDP-Glc-Nac/UDP-glucose transporter), member D2 | 17.486 | −6.774 | −24.260 | −13.522 | −8.730 | 0.333 | 0.025 |  |
| 8072484 | MGC15705 | hypothetical protein MGC15705 | 4.584 | −3.462 | −8.046 | −10.626 | 0.662 | 0.333 | 0.025 |  |
| 7932433 | NSUN6 | NOP2/sun domain family, member 6 | 22.475 | −1.012 | −23.487 | −13.840 | −5.205 | 0.333 | 0.025 |  |
| 8099721 | SEL1L3 | sel-1 suppressor of lin-12-like 3 (C.elegans) | 100.986 | 0.006 | −100.980 | −17.347 | 8.631 | 0.334 | 0.024 |  |
| 8146649 | MTFR1 | mitochondrial fission regulator 1 | 17.770 | 0.793 | −16.978 | −11.451 | −2.017 | 0.334 | 0.025 |  |
| 7900624 | ZNF691 | zinc finger protein 691 | 6.394 | −3.499 | −9.893 | −10.410 | −8.081 | 0.335 | 0.025 |  |
| 8159004 | SNORD24 | small nucleolar RNA, C/D box 24 | 10.844 | −1.669 | −12.512 | −18.371 | −3.347 | 0.335 | 0.025 |  |
| 7903321 | RTCD1 | RNA 3′-terminal phosphate cyclase | 49.162 | 6.857 | −42.305 | −10.480 | −7.954 | 0.336 | 0.024 |  |
| 8066612 | NEURL2 | neuralized E3 ubiquitin protein ligase 2 | −7.986 | −1.250 | 6.736 | 10.127 | 3.799 | 0.336 | 0.025 |  |
| 8077944 | CAND2 | cullin-associated and neddylation-dissociated 2 (putative) | −1.282 | 6.821 | 8.103 | 13.239 | 4.573 | 0.336 | 0.025 |  |
| 8092265 | MRPL47 | mitochondrial ribosomal protein L47 | 11.389 | −4.442 | −15.831 | −11.463 | −7.805 | 0.337 | 0.025 |  |
| 7942647 | LOC387790 | hypothetical LOC387790 | −9.510 | −1.324 | 8.186 | 11.777 | −1.428 | 0.337 | 0.025 |  |
| 8081503 | DZIP3 | DAZ interacting zinc finger protein 3 | 15.793 | 3.307 | −12.486 | −16.189 | −0.371 | 0.337 | 0.025 |  |
| 7995161 | ITGAD | integrin, alpha D | 3.357 | −4.033 | −7.390 | −10.752 | −0.742 | 0.337 | 0.025 |  |
| 8070786 | KRTAP10-6 | keratin associated protein 10-6 | −4.492 | 4.861 | 9.354 | 10.964 | 8.098 | 0.338 | 0.025 |  |
| 8000856 | TBC1D10B | TBC1 domain family, member 10B | −17.248 | −2.921 | 14.327 | 10.062 | 0.118 | 0.338 | 0.025 |  |
| 8178435 | IER3 | immediate early response 3­­ | 4.703 | −54.515 | −59.218 | −20.778 | 1.680 | 0.339 | 0.024 |  |
| 7949995 | MRPL21 | mitochondrial ribosomal protein L21 | 27.250 | 3.826 | −23.423 | −12.654 | −3.013 | 0.339 | 0.025 |  |
| 8124828 | FLOT1 | flotillin 1 | 88.689 | 1.911 | −86.777 | −10.768 | 8.384 | 0.340 | 0.024 |  |
| 8178419 | FLOT1 | flotillin 1 | 88.689 | 1.911 | −86.777 | −10.768 | 8.384 | 0.340 | 0.024 |  |
| 8179688 | FLOT1 | flotillin 1 | 88.689 | 1.911 | −86.777 | −10.768 | 8.384 | 0.340 | 0.024 |  |
| 8099826 | TLR10 | toll-like receptor10 | 27.183 | 0.986 | −26.197 | −12.797 | 4.975 | 0.340 | 0.025 |  |
| 8161446 | FAM27E3 | family with sequence similarity 27, member E3 | 8.073 | −5.911 | −13.984 | −15.746 | −2.676 | 0.340 | 0.025 |  |
| 8030908 | ZNF480 | zinc finger protein 480 | 38.443 | 4.202 | −34.241 | −13.403 | −1.496 | 0.340 | 0.024 |  |
| 8110618 | ARPP19 | cAMP-regulated phosphoprotein, 19kDa | 30.716 | 5.600 | −25.116 | −14.341 | 2.420 | 0.340 | 0.025 |  |
| 8142971 | KLF14 | Kruppel-like factor 14 | −5.618 | 1.373 | 6.992 | 11.853 | 7.352 | 0.341 | 0.025 |  |
| 7982284 | LOC653075 | golginA8 family, member A pseudogene | −11.065 | 12.828 | 23.893 | 19.475 | 9.063 | 0.341 | 0.025 |  |
| 8147439 | PLEKHF2 | pleckstrin homology domain containing, family F (with FYVE domain) member 2 | 35.260 | 7.828 | −27.432 | −11.364 | 1.105 | 0.342 | 0.025 |  |
| 8001185 | DNAJA2 | DnaJ (Hsp40) homolog, subfamily A, member 2 | 29.189 | 1.706 | −27.483 | −12.677 | −9.918 | 0.343 | 0.025 |  |
| 8141898 | DNAJC2 | DnaJ (Hsp40) homolog, subfamily C, member 2 | 12.776 | −0.587 | −13.363 | −13.576 | −6.600 | 0.343 | 0.025 |  |
| 7936320 | RPL13AP6 | ribosomal protein L13a pseudogene 6 | 15.761 | 6.773 | −8.987 | −12.289 | −3.728 | 0.343 | 0.025 |  |
| 8081537 | TRAT1 | T cell receptor associated transmembrane adaptor 1 | 192.149 | 109.172 | −82.977 | −10.493 | 2.778 | 0.343 | 0.023 |  |
| 8012423 | C17orf68 | chromosome 17 open reading frame 68 | −60.902 | −7.577 | 53.325 | 11.224 | 7.890 | 0.343 | 0.024 |  |
| 7926345 | RPP38 | ribonuclease P/MRP 38kDA subunit | 25.129 | 1.926 | −23.203 | −13.017 | −3.924 | 0.343 | 0.025 |  |
| 8169009 | BEX4 | brain expressed, X-linked 4 | 10.386 | −8.559 | −18.945 | −10.184 | 0.957 | 0.343 | 0.025 |  |
| 8076195 | PDGFB | platelet-derived growth factor beta polypeptide | −2.778 | 3.059 | 5.836 | 10.719 | 6.282 | 0.343 | 0.025 |  |
| 7946579 | LYVE1 | lymphatic vessel endothelial hyaluronan receptor 1 | 6.752 | −1.493 | −8.245 | −14.549 | −7.518 | 0.343 | 0.025 |  |
| 8171917 | FTHL17 | ferritin, heavy polypeptide-like 17 | −11.491 | −1.898 | 9.593 | 14.228 | 8.105 | 0.343 | 0.025 |  |
| 8113369 | SLCO4C1 | solute carrier organic anion transporter family, member 4C1 | 16.237 | −44.058 | −60.295 | −13.783 | −9.721 | 0.344 | 0.024 |  |
| 8030403 | PRR12 | proline rich 12 | −33.860 | −6.083 | 27.777 | 10.168 | −1.830 | 0.344 | 0.025 |  |
| 7949080 | NRXN2 | neurexin 2 | −10.272 | −1.702 | 8.570 | 12.449 | 8.322 | 0.345 | 0.025 |  |
| 8120943 | CYB5R4 | cytochrome B5 reductase 4 | 77.457 | 5.243 | −72.214 | −10.972 | −5.950 | 0.346 | 0.024 |  |
| 7959995 | EP400 | E1A binding protein P400 | −42.770 | −9.809 | 32.961 | 11.532 | −0.696 | 0.347 | 0.024 |  |
| 7973629 | REC8 | REC8 meiotic recombination protein | −20.181 | −5.166 | 15.015 | 10.557 | −5.559 | 0.347 | 0.025 |  |
| 8043504 | MAL | mal, T-cell differentiation protein | −15.372 | 53.138 | 68.510 | 14.685 | 0.403 | 0.347 | 0.024 |  |
| 8034075 | CDKN2D | cyclin-dependent kinase inhibitor 2D (P19, inhibits CDK4) | 11.089 | −10.216 | −21.306 | −12.514 | −8.002 | 0.347 | 0.025 |  |
| 8117685 | ZKSCAN3 | zinc finger with KRAB and SCAN domains 3 | 9.429 | −1.124 | −10.553 | −15.376 | −1.460 | 0.348 | 0.025 |  |
| 8116734 | LY86 | lymphocyte antigen 86 | 15.173 | −39.866 | −55.038 | −15.796 | −8.900 | 0.348 | 0.024 |  |
| 8030736 | ACPT | acid phosphatase, testicular | −5.555 | 2.226 | 7.782 | 10.314 | 3.752 | 0.348 | 0.025 |  |
| 7980990 | C14orf142 | chromosome 14 open reading frame 142 | 2.424 | −7.098 | −9.521 | −11.345 | −3.026 | 0.349 | 0.025 |  |
| 8113358 | ST8SIA4 | ST8 alpha -*N*-acetyl-neuraminide alpha -2,8-sialyltransferase 4 | 120.273 | 2.586 | −117.687 | −10.729 | −5.026 | 0.349 | 0.023 |  |
| 8064464 | SIRPD | signal-regulatory protein delta | 17.347 | −26.889 | −44.236 | −10.659 | −6.992 | 0.349 | 0.024 |  |
| 8106633 | MSH3 | MutS homolog 3 | 29.272 | 1.206 | −28.066 | −10.957 | −4.586 | 0.350 | 0.025 |  |
| 8075963 | SH3BP1 | SH3-domain binding protein 1 | −21.715 | −3.503 | 18.212 | 26.387 | 9.380 | 0.350 | 0.025 |  |
| 7940066 | TMX2 | thioredoxin-related transmembrane protein 2 | 8.123 | −18.900 | −27.023 | −10.896 | 3.852 | 0.351 | 0.025 |  |
| 8124307 | CMAH | cytidine monophosphate-*N*-acetylneuraminic acid hydroxylase, pseudogene | 16.079 | −6.247 | −22.326 | −12.990 | 2.481 | 0.351 | 0.025 |  |
| 8023561 | LMAN1 | lectin, mannose-binding, 1 | 72.855 | 6.235 | −66.620 | −19.817 | 1.461 | 0.351 | 0.024 |  |
| 8005809 | LGALS9 | lectin, galactoside-binding, soluble, 9 | −20.133 | 0.738 | 20.872 | 13.587 | 7.431 | 0.352 | 0.025 |  |
| 8165711 | PLCXD1 | phosphatidylinositol-specific phospholipase C, X domain containing 1 | 2.989 | −5.911 | −8.899 | −10.574 | −6.577 | 0.352 | 0.025 |  |
| 7994572 | RUNDC2C | sorting nexin 29 pseudogene 2 | −4.902 | −33.760 | −28.857 | −14.408 | 5.937 | 0.353 | 0.025 |  |
| 7987361 | ZNF770 | zinc finger protein 770 | 17.706 | −0.666 | −18.372 | −10.920 | −1.597 | 0.353 | 0.025 |  |
| 8030022 | GRIN2D | glutamate receptor, ionotropic, *N*-methyl d-aspartate 2D | −7.096 | 2.269 | 9.365 | 16.768 | 5.129 | 0.353 | 0.025 |  |
| 7961365 | MANSC1 | MANSC domain containing 1 | 37.962 | −27.910 | −65.872 | −13.027 | −2.607 | 0.353 | 0.024 |  |
| 7983298 | C15orf63 | chromosome 15 open reading frame 63 | 1.984 | −4.736 | −6.720 | −10.372 | −3.687 | 0.354 | 0.025 |  |
| 8051501 | EIF2AK2 | eukaryotic translation initiation factor 2-alpha kinase 2 | –105.810 | 83.249 | 189.059 | 28.956 | 0.612 | 0.354 | 0.024 |  |
| 8097553 | IL15 | interleukin 15 | −3.317 | 1.513 | 4.830 | 10.767 | −4.047 | 0.354 | 0.025 |  |
| 7969093 | MLNR | motilin receptor | −6.391 | −1.014 | 5.377 | 10.280 | 7.084 | 0.355 | 0.025 |  |
| 7914245 | MECR | mitochondrial trans-2-enoyl-CoA reductase | 5.930 | −6.237 | −12.167 | −11.295 | 0.111 | 0.355 | 0.025 |  |
| 8043278 | MRPL35 | mitochondrial ribosomal protein L35 | 11.543 | −4.347 | −15.890 | −13.598 | 1.059 | 0.355 | 0.025 |  |
| 7902367 | ACADM | acyl-CoAdehydrogenase, C-4 to C-12 straight chain | 40.855 | 5.335 | −35.519 | −11.049 | 8.817 | 0.356 | 0.024 |  |
| 8060813 | MCM8 | minichromosome maintenance 8 homologous recombination repair factor | 22.694 | 3.196 | −19.498 | −18.349 | −5.502 | 0.356 | 0.025 |  |
| 8148198 | WDYHV1 | WDYHV motif containing 1 | 3.961 | −1.736 | −5.696 | −11.225 | −9.162 | 0.356 | 0.025 |  |
| 8070853 | C21orf67 | chromosome 21 open reading frame 67 | −3.994 | 1.786 | 5.780 | 10.169 | 8.195 | 0.357 | 0.025 |  |
| 8007584 | ASB16 | ankyrin repeat and SOCS box-containing 16 | 3.797 | −3.601 | −7.398 | −12.375 | −7.603 | 0.357 | 0.025 |  |
| 8160823 | CNTFR | ciliary neurotrophic factor receptor | −3.444 | 2.835 | 6.279 | 11.916 | 9.419 | 0.357 | 0.025 |  |
| 8065327 | NKX2-4 | NK2 homeobox 4 | −9.872 | −0.948 | 8.924 | 10.495 | 6.810 | 0.357 | 0.025 |  |
| 7998427 | TPSG1 | tryptase gamma 1 | −2.626 | 2.452 | 5.078 | 10.004 | 1.098 | 0.357 | 0.025 |  |
| 8012466 | KRBA2 | KRAB-A domain containing 2 | 4.062 | −2.149 | −6.212 | −11.097 | −7.232 | 0.358 | 0.025 |  |
| 8139488 | IGFBP3 | insulin-like growth factor binding protein 3 | −2.332 | 6.642 | 8.975 | 12.990 | −0.781 | 0.358 | 0.025 |  |
| 7965245 | MKRN9P | makorinring finger protein 9, pseudogene | −1.113 | 5.760 | 6.873 | 13.792 | −2.261 | 0.358 | 0.025 |  |
| 8026513 | TPM4 | tropomyosin 4 | −9.414 | 20.898 | 30.312 | 10.457 | 6.219 | 0.359 | 0.024 |  |
| 8080804 | KCTD6 | potassium channel tetramerisation domain containing 6 | 8.374 | −2.330 | −10.705 | −12.302 | −5.872 | 0.359 | 0.025 |  |
| 8109639 | PTTG1 | pituitary tumor-transforming 1 | 5.267 | −1.932 | −7.199 | −13.354 | −4.430 | 0.359 | 0.025 |  |
| 8137240 | GIMAP7 | GTPase, IMAP family member 7 | 34.023 | −25.614 | −59.637 | −13.636 | −7.613 | 0.360 | 0.024 |  |
| 7931268 | BCCIP | BRCA2 and CDKN1A interacting protein | 9.297 | −0.694 | −9.991 | −11.484 | −4.362 | 0.360 | 0.025 |  |
| 8179235 | HCG27 | HLA complex group 27 (non-protein coding) | 18.831 | −46.304 | −65.134 | −14.447 | −4.002 | 0.360 | 0.024 |  |
| 7981720 | IGHV3-48 | immunoglobulin heavy variable 3-48 | 136.814 | 9.655 | −127.158 | −23.904 | 8.070 | 0.361 | 0.024 |  |
| 8166157 | BMX | BMX non-receptor tyrosine kinase | 6.798 | −2.549 | −9.347 | −17.964 | −6.777 | 0.361 | 0.025 |  |
| 8030383 | RCN3 | reticulocalbin 3, EF-hand calcium binding domain | −16.393 | −4.509 | 11.884 | 11.375 | 0.953 | 0.361 | 0.025 |  |
| 8043993 | LOC100131131 | AHPA9419 | −31.668 | −3.359 | 28.309 | 18.964 | −3.518 | 0.362 | 0.025 |  |
| 8043478 | IGKV1D-8 | immunoglobulin kappa variable 1D-8 | 4.090 | −5.419 | −9.509 | −18.728 | 5.754 | 0.363 | 0.025 |  |
| 8076431 | CYP2D7P1 | cytochrome P450, family 2, subfamily D, polypeptide 7 (gene/pseudogene) | −17.555 | −5.348 | 12.207 | 11.349 | 5.348 | 0.363 | 0.025 |  |
| 8154934 | IL11RA | interleukin 11 receptor, alpha | −29.810 | −8.786 | 21.025 | 13.645 | 5.670 | 0.363 | 0.025 |  |
| 8056285 | IFIH1 | interferon induced with helicase C domain 1 | −38.964 | 18.274 | 57.237 | 22.797 | 8.011 | 0.364 | 0.025 |  |
| 7924184 | NCRNA00292 | FLVCR1 antisense RNA 1 (heat to head) | 8.635 | −3.055 | −11.689 | −22.167 | −3.473 | 0.364 | 0.025 |  |
| 7954012 | LOH12CR1 | BLOC-1 related complect subunit 5 | −10.118 | 17.688 | 27.806 | 10.440 | 8.752 | 0.364 | 0.025 |  |
| 8129861 | IFNGR1 | interferon gamma receptor 1 | 121.302 | −15.412 | −136.714 | −11.686 | 2.126 | 0.364 | 0.023 |  |
| 8004940 | C17orf48 | chromosome 17 open reading frame 48 | 15.199 | −0.742 | −15.942 | −15.237 | −5.323 | 0.364 | 0.025 |  |
| 8095680 | IL8 | chemokine (C-X-C motif) ligand 8 | 17.151 | −3.461 | −20.611 | −18.471 | 3.945 | 0.364 | 0.025 |  |
| 8071051 | MAFIP | MAFF interacting protein (pseudogene) | −9.943 | 23.181 | 33.124 | 21.495 | −0.314 | 0.364 | 0.025 |  |
| 8043036 | LOC1720 | dihydrofolate reductase pseudogene | 21.751 | 3.280 | −18.471 | −17.224 | −6.849 | 0.364 | 0.025 |  |
| 8043602 | NCAPH | non-SMC condensing I complex, subunit H | 1.340 | 8.424 | 7.083 | 11.807 | 9.132 | 0.365 | 0.025 |  |
| 8041745 | C2orf34 | chromosome 2 open reading frame 34 | 13.321 | −3.385 | −16.706 | −11.351 | 0.759 | 0.365 | 0.025 |  |
| 8148985 | ERICH1 | glutamate-rich 1 | 5.352 | −38.083 | −43.435 | −10.972 | −9.572 | 0.366 | 0.024 |  |
| 7948908 | SNORD26 | small nucleolar RNA, C/D box 26 | 10.368 | −5.493 | −15.861 | −12.316 | −9.388 | 0.366 | 0.025 |  |
| 7928401 | CHCHD1 | coiled-coil-helix-coiled-coil-helix domain containing 1 | 11.970 | 4.135 | −7.835 | −11.438 | −4.121 | 0.366 | 0.025 |  |
| 8122756 | PLEKHG1 | pleckstrin homology domain containing, family G (with RhoGef domain) member 1 | 4.754 | −2.958 | −7.712 | −10.198 | 4.733 | 0.366 | 0.025 |  |
| 8057719 | HIBCH | 3-hydroxyisobutyryl-CoA hydrolase | 28.837 | 8.009 | −20.828 | −12.486 | −6.509 | 0.367 | 0.025 |  |
| 8179228 | TCF19 | transcription factor 19 | −6.163 | 4.510 | 10.673 | 12.112 | 1.629 | 0.368 | 0.025 |  |
| 8118086 | TCF19 | transcription factor 19 | −6.163 | 4.510 | 10.673 | 13.131 | 2.169 | 0.368 | 0.025 |  |
| 8134470 | TRRAP | transformation/transcription domain-associated protein | −73.055 | −18.705 | 54.350 | 10.769 | 1.745 | 0.368 | 0.024 |  |
| 8162639 | ZNF782 | zinc finger protein 782 | 8.977 | −2.616 | −11.593 | −11.755 | −8.923 | 0.369 | 0.025 |  |
| 7909441 | G0S2 | G0/G1 switch 2 | 3.604 | −4.791 | −8.396 | −11.289 | −7.003 | 0.369 | 0.025 |  |
| 7982564 | SPRED1 | sprouty-related, EVH1 domain containing 1 | −4.815 | 5.165 | 9.980 | 12.697 | 3.080 | 0.370 | 0.025 |  |
| 7967175 | KDM2B | lysine (K)-specific demethylase 2B | 4.417 | −13.055 | −17.471 | −11.761 | −4.785 | 0.370 | 0.025 |  |
| 8163599 | DFNB31 | deafness, autosomal recessive 31 | −8.709 | −0.091 | 8.618 | 10.870 | 2.865 | 0.372 | 0.025 |  |
| 8116649 | TUBB2A | tubulin, beta 2A | −1.723 | 9.758 | 11.481 | 13.707 | 2.289 | 0.372 | 0.025 |  |
| 7934215 | SPOCK2 | sparc/osteonectin, Cwcv and Kazal-like domains proteoglycan (testican) 2 | −60.260 | 46.186 | 106.446 | 11.576 | 4.700 | 0.372 | 0.023 |  |
| 8008900 | CA4 | carbonic anhydrase IV | 7.718 | −10.528 | −18.246 | −12.234 | 6.099 | 0.373 | 0.025 |  |
| 8001953 | EXOC3L | exocyst complex component 3-like 1 | −3.043 | 2.677 | 5.720 | 10.599 | 7.361 | 0.374 | 0.025 |  |
| 8122409 | PEX3 | peroxisomal biogenesis factor 3 | 19.666 | 4.862 | −14.804 | −14.443 | −5.349 | 0.375 | 0.025 |  |
| 8111443 | C1QTNF3 | C1q and tumor necrosis factor related protein 3 | 2.846 | −5.490 | −8.336 | −13.138 | −6.943 | 0.375 | 0.025 |  |
| 8031617 | ZNF444 | zinc finger protein 444 | −1.499 | 4.457 | 5.956 | 10.500 | 7.720 | 0.376 | 0.025 |  |
| 7958000 | CHPT1 | choline phosphotransferase 1 | 102.030 | −33.499 | −135.529 | −12.287 | 0.302 | 0.377 | 0.023 |  |
| 8012450 | SLC25A35 | solute carrier family 25, member 35 | −9.642 | −0.202 | 9.440 | 11.807 | 5.571 | 0.377 | 0.025 |  |
| 8040578 | CENPO | centromere protein O | 11.450 | 1.797 | −9.653 | −11.275 | −7.893 | 0.378 | 0.025 |  |
| 8079370 | CCR9 | chemokine (C–C motif) receptor 9 | −5.770 | 3.397 | 9.167 | 17.400 | 6.455 | 0.378 | 0.025 |  |
| 7955156 | CCDC65 | coiled-coil domain containing 65 | 7.242 | −1.324 | −8.567 | −14.342 | −8.434 | 0.378 | 0.025 |  |
| 8025237 | KIAA1543 | pleckstrin homology domain containing, family A member 7 | −5.763 | −0.807 | 4.956 | 10.319 | −8.964 | 0.379 | 0.025 |  |
| 7909175 | SRGAP2 | SLIT-ROBO Rho GTPase activating protein 2 | −83.766 | −32.506 | 51.260 | 17.020 | −8.599 | 0.379 | 0.024 |  |
| 7980702 | C14orf102 | chromosome 14 open reading frame 102 | 6.355 | −4.872 | −11.227 | −13.076 | −5.292 | 0.379 | 0.025 |  |
| 7960764 | RBP5 | retinol binding protein 5, cellular | −3.268 | 3.174 | 6.442 | 11.737 | 3.385 | 0.380 | 0.025 |  |
| 8007112 | KRTAP4-7 | keratin associated protein 4-7 | −17.646 | 2.812 | 20.457 | 15.167 | 2.953 | 0.381 | 0.025 |  |
| 8033667 | ZNF558 | zinc finger protein 558 | 11.648 | −1.023 | −12.671 | −14.235 | −7.638 | 0.381 | 0.025 |  |
| 8176026 | FLNA | Filamin A, alpha | –294.384 | –111.759 | 182.625 | 10.084 | −1.374 | 0.382 | 0.022 |  |
| 8151401 | UBE2W | ubiquitin-conjugating enzyme E2W (putative) | 41.738 | 0.911 | −40.827 | −11.633 | −7.776 | 0.382 | 0.024 |  |
| 8031640 | ZNF583 | zinc finger protein 583 | 10.794 | −0.746 | −11.540 | −12.885 | 3.168 | 0.382 | 0.025 |  |
| 7925500 | CHML | choroideremia-like (Rab escort protein 2) | 9.014 | −6.333 | −15.347 | −13.137 | −2.636 | 0.382 | 0.025 |  |
| 8094848 | SLC30A9 | solute carrier family 30 (zinc transporter), member 9 | 28.392 | 4.312 | −24.080 | −10.374 | −2.341 | 0.383 | 0.025 |  |
| 8029377 | ZNF224 | zinc finger protein 224 | 10.276 | −7.312 | −17.589 | −11.133 | −7.883 | 0.383 | 0.025 |  |
| 7965846 | CCDC53 | coiled-coil domain containing 53 | 11.633 | −4.982 | −16.615 | −10.730 | −6.593 | 0.383 | 0.025 |  |
| 8039687 | ZNF552 | zinc finger protein 552 | 4.714 | −35.204 | −39.918 | −36.812 | −3.274 | 0.383 | 0.025 |  |
| 7964575 | AVIL | Advillin | 9.909 | −0.675 | −10.584 | −14.873 | −9.692 | 0.384 | 0.025 |  |
| 8147221 | OSGIN2 | oxidative stress induced growth inhibitor family member 2 | 29.383 | 0.088 | −29.295 | −12.193 | −1.678 | 0.384 | 0.025 |  |
| 8155514 | FAM27E3 | family with sequence similarity 27, member E3 | 8.366 | −6.362 | −14.728 | −16.866 | −1.622 | 0.384 | 0.025 |  |
| 7994345 | SBK1 | SH3 binding domain kinase 1 | −9.223 | 6.525 | 15.748 | 10.170 | 3.576 | 0.384 | 0.025 |  |
| 8136930 | TAS2R60 | taste receptor, type 2, member 60 | −19.989 | 1.060 | 21.049 | 15.646 | 9.805 | 0.385 | 0.025 |  |
| 7951422 | KIAA1826 | Myb/SANT-like DNA-binding domain containing 4 with coiled-coils | 11.487 | −0.406 | −11.893 | −11.802 | −6.503 | 0.385 | 0.025 |  |
| 8108199 | SLC25A48 | solute carrier family 25, member 48 | 0.835 | −8.106 | −8.941 | −14.497 | −2.669 | 0.385 | 0.025 |  |
| 8033023 | PRR22 | proline rich 22 | −7.486 | 1.007 | 8.493 | 10.180 | 6.349 | 0.386 | 0.025 |  |
| 8007867 | LRRC37A2 | leucine rich repeat containing 37, member A2 | 25.596 | −67.115 | −92.711 | −10.033 | −6.887 | 0.386 | 0.023 |  |
| 7960143 | ZNF84 | zinc finger protein 84 | 13.935 | −3.648 | −17.582 | −13.845 | −4.154 | 0.386 | 0.025 |  |
| 8036324 | ZNF260 | zinc finger protein 260 | 19.840 | 1.023 | −18.817 | −13.116 | −2.393 | 0.386 | 0.025 |  |
| 8029321 | ZNF283 | zinc finger protein 283 | 6.946 | −0.143 | −7.088 | −13.831 | −9.039 | 0.386 | 0.025 |  |
| 7925480 | FH | fumarate hydratase | 12.603 | 4.336 | −8.267 | −11.641 | −5.254 | 0.387 | 0.025 |  |
| 8039109 | VSTM1 | V-set and transmembrane domain containing 1 | 0.690 | −21.436 | −22.126 | −10.297 | 1.283 | 0.387 | 0.025 |  |
| 8055872 | CACNB4 | calcium channel, voltage-dependent, beta 4 subunit | −7.667 | 3.063 | 10.730 | 19.357 | 2.488 | 0.387 | 0.025 |  |
| 8022531 | NPC1 | Niemann-Pick disease, type C1 | −60.155 | −2.895 | 57.260 | 11.968 | 3.188 | 0.387 | 0.024 |  |
| 8010696 | NPB | neuropeptide B | −1.798 | 7.331 | 9.129 | 10.834 | 4.181 | 0.387 | 0.025 |  |
| 8019693 | NPB | neuropeptide B | −1.798 | 7.331e | 9.129 | 10.834 | 4.181 | 0.387 | 0.025 |  |
| 7951325 | DCUN1D5 | DCN1, defective in cullin neddylation 1, domain containing 5 | 8.084 | −4.358 | −12.442 | −12.859 | −7.369 | 0.387 | 0.025 |  |
| 7912224 | SLC2A5 | solute carrier family 2 (facilitated glucose/fructose transporter), member 5 | 6.950 | −0.282 | −7.232 | −14.389 | −6.661 | 0.388 | 0.025 |  |
| 8068697 | MX2 | MX dynamin-like GTPase 2 | −99.501 | 97.449 | 196.950 | 13.012 | 0.120 | 0.388 | 0.022 |  |
| 7899939 | GJA4 | gap junction protein, alpha 4, 37kDa | −8.102 | 0.127 | 8.230 | 14.737 | −1.290 | 0.388 | 0.025 |  |
| 8007850 | LRRC37A2 | leucine rich repeat containing 37, member A2 | 26.021 | −67.452 | −93.473 | −10.287 | −6.978 | 0.389 | 0.023 |  |
| 8158539 | C9orf106 | chromosome 9 open reading frame 106 | 11.383 | −4.003 | −15.386 | −12.794 | 7.137 | 0.389 | 0.025 |  |
| 8009301 | PRKCA | protein kinase C, alpha | −33.350 | 11.688 | 45.038 | 10.623 | −0.153 | 0.389 | 0.024 |  |
| 8095760 | THAP6 | THAP domain containing 6 | 19.871 | 4.817 | −15.053 | −10.505 | −5.913 | 0.390 | 0.025 |  |
| 8145027 | FAM160B2 | family with sequence similarity 160, member B2 | −24.440 | −4.989 | 19.451 | 11.244 | 3.396 | 0.391 | 0.025 |  |
| 7931888 | ANKRD16 | ankyrin repeat domain 16 | 5.975 | 0.455 | −5.520 | −10.934 | −6.349 | 0.391 | 0.025 |  |
| 8021984 | YES1 | YES proto-oncogense 1, src family tyrosine kinase | 5.350 | −1.033 | −6.383 | −13.823 | −2.277 | 0.392 | 0.025 |  |
| 8144701 | LOC100131581 | uncharacterized LOC100131581 | −0.662 | −12.106 | −11.444 | −14.966 | −8.442 | 0.392 | 0.025 |  |
| 7980765 | GPR68 | G protein-coupled receptor 68 | −5.475 | 2.270 | 7.745 | 10.276 | 1.079 | 0.392 | 0.025 |  |
| 8160016 | RANBP6 | RAN binding protein 6 | 21.579 | 2.323 | −19.256 | −10.799 | −3.105 | 0.393 | 0.025 |  |
| 7976876 | DYNC1H1 | dynein, cytoplasmic 1, heavy chain 1 | −75.778 | −14.576 | 61.202 | 11.951 | −1.693 | 0.394 | 0.024 |  |
| 7980246 | MLH3 | MutL homolog 3 | 13.318 | −0.667 | −13.985 | −10.424 | −4.802 | 0.394 | 0.025 |  |
| 8137332 | ABCB8 | ATP-bindingcassette, sub-family B (MDR/TAP), member 8 | −6.189 | 4.144 | 10.333 | 15.994 | 4.044 | 0.394 | 0.025 |  |
| 8169984 | HPRT1 | hypoxanthine phosphoribosyltransferase 1 | 10.153 | −2.044 | −12.197 | −10.437 | −2.782 | 0.395 | 0.025 |  |
| 8101489 | FAM175A | family with sequence similarity 175, member A | 1.710 | 18.912 | 17.203 | 11.234 | 8.170 | 0.395 | 0.025 |  |
| 8103601 | DDX60L | DEAD (Asp-Glu-Ala-Asp) box polypeptide 60-like | −79.973 | 67.045 | 147.019 | 16.906 | −1.813 | 0.395 | 0.023 |  |
| 7904433 | PHGDH | phosphoglycerate dehydrogenase | 8.613 | 0.367 | −8.246 | −11.055 | 0.368 | 0.395 | 0.025 |  |
| 8145532 | EPHX2 | epoxide hydrolase 2, cytoplasmic | −5.186 | 11.454 | 16.640 | 16.304 | 8.385 | 0.397 | 0.025 |  |
| 8077116 | ODF3B | outer dense fiber of sperm tails 3B | −17.401 | −0.019 | 17.382 | 11.725 | 2.534 | 0.397 | 0.025 |  |
| 8149979 | C8orf80 | chromosome 8 open reading frame 80 | 15.234 | 0.597 | −14.637 | −22.613 | −2.361 | 0.397 | 0.025 |  |
| 8162669 | ZNF322A | zinc finger protein 322A | 5.365 | −0.778 | −6.143 | −10.978 | −1.813 | 0.397 | 0.025 |  |
| 8036341 | ZNF461 | zinc finger protein 461 | 8.219 | 1.968 | −6.252 | −10.712 | −6.400 | 0.397 | 0.025 |  |
| 7939559 | TSPAN18 | tetraspanin 18 | −26.555 | 9.842 | 36.397 | 10.215 | 4.174 | 0.397 | 0.024 |  |
| 7942964 | TMEM135 | transmembrane protein 135 | 15.398 | −0.742 | −16.140 | −12.001 | −7.849 | 0.398 | 0.025 |  |
| 8108174 | PCBD2 | pterin-4 alpha-carbinolamine dehydratase/dimerization cofactor of hepatocyte nuclear factor 1 alpha (TCF1) 2 | 6.842 | −4.178 | −11.020 | −13.270 | −8.172 | 0.399 | 0.025 |  |
| 7918622 | SLC16A1 | solute carrier family 16 (monocarboxylate transporter), member 1 | −4.531 | 2.409 | 6.940 | 11.014 | 1.850 | 0.399 | 0.025 |  |
| 7937713 | SYT8 | synaptotagminVIII | −2.275 | 4.766 | 7.041 | 12.257 | 8.498 | 0.400 | 0.025 |  |
| 8100298 | OCIAD2 | OCIA domain containing 2 | 15.758 | −3.981 | −19.740 | −16.138 | −9.561 | 0.400 | 0.025 |  |
| 8177947 | TCF19 | transcription factor 19 | −6.974 | 3.583 | 10.556 | 12.988 | 2.169 | 0.401 | 0.025 |  |
| 7912854 | ESPNP | espin pseudogene | −10.290 | 0.095 | 10.385 | 12.779 | 4.115 | 0.401 | 0.025 |  |
| 7905517 | LCE1F | late cornified envelope 1F | −14.010 | −0.694 | 13.315 | 11.881 | 5.882 | 0.401 | 0.025 |  |
| 8102912 | TBC1D9 | TBC1 domain family, member 9 (with GRAM domain) | 8.635 | −8.939 | −17.574 | −10.252 | −8.928 | 0.401 | 0.025 |  |
| 8028186 | ZNF146 | zinc finger protein 146 | 35.784 | 11.547 | −24.236 | −10.101 | −3.244 | 0.402 | 0.025 |  |
| 7922137 | NME7 | NME/NM23 family member 7 | 4.751 | −1.224 | −5.976 | −12.180 | −7.649 | 0.402 | 0.025 |  |
| 8034097 | LOC147727 | hypothetical LOC147727 | 5.442 | −5.748 | −11.189 | −11.932 | 2.043 | 0.402 | 0.025 |  |
| 7949015 | C11orf95 | chromosome 11 open reading frame 95 | −9.578 | 4.130 | 13.708 | 10.001 | 6.327 | 0.402 | 0.025 |  |
| 8139632 | FIGNL1 | fidgetin-like 1 | 5.221 | −2.392 | −7.613 | −14.796 | 6.400 | 0.403 | 0.025 |  |
| 8048171 | PK155 | hypothetical protein DKFZp434H1419 | −26.200 | 0.166 | 26.366 | 11.867 | 8.073 | 0.404 | 0.025 |  |
| 7982738 | BAHD1 | bromo adjacent homology domain containing 1 | −17.408 | −3.041 | 14.367 | 10.549 | 7.067 | 0.404 | 0.025 |  |
| 8174761 | NKAPP1 | NFKB activating protein pseudogene 1 | −5.190 | 7.182 | 12.372 | 11.480 | 6.745 | 0.406 | 0.025 |  |
| 8157691 | SKA2 | spindle and kinetochore associated complex subunit 2 | 45.091 | 13.884 | −31.206 | −11.853 | −2.637 | 0.406 | 0.024 |  |
| 8067221 | ZBP1 | Z-DNA binding protein 1 | −43.135 | 14.804 | 57.938 | 14.467 | 4.560 | 0.407 | 0.024 |  |
| 8014257 | SLFN12L | schlafen family member 12-like | 113.429 | −15.194 | −128.623 | −12.246 | 5.405 | 0.408 | 0.023 |  |
| 8030831 | ZNF175 | zinc finger protein 175 | 14.635 | 2.826 | −11.809 | −10.900 | −9.789 | 0.408 | 0.025 |  |
| 7944739 | CRTAM | cytotoxic and regulatory T cell molecule | −4.703 | 5.452 | 10.155 | 13.220 | 5.933 | 0.409 | 0.025 |  |
| 7991313 | PLIN1 | perilipin 1 | 2.685 | −2.490 | −5.175 | −10.447 | −1.381 | 0.409 | 0.025 |  |
| 7907531 | GPR52 | G protein-coupled receptor 52 | −42.325 | 6.953 | 49.278 | 15.088 | 5.914 | 0.409 | 0.024 |  |
| 7902425 | ST6GALNAC3 | ST6 (alpha-*N*-acetyl-neuraminyl-2,3-beta-galactosyl-1,3)-*N*-acetylgalactosaminide alpha-2,6-sialyltransferase 3 | −10.847 | −2.164 | 8.683 | 10.125 | −2.806 | 0.410 | 0.025 |  |
| 8051226 | TRMT61B | tRNA methyltransferase 61B | 10.795 | −1.020 | −11.815 | −11.859 | −9.738 | 0.410 | 0.025 |  |
| 7917516 | GBP1 | guanylate binding protein 1, interferon-inducible | −32.866 | 220.934 | 253.800 | 19.588 | 5.742 | 0.411 | 0.022 |  |
| 8017476 | CSH2 | chorionic somatomammotropin hormone 2 | −2.411 | 7.393 | 9.804 | 17.737 | 7.550 | 0.413 | 0.025 |  |
| 7947138 | FANCF | fanconi anemia, complementation group F | 14.129 | 1.335 | −12.794 | −11.484 | −2.431 | 0.413 | 0.025 |  |
| 8071981 | KIAA1671 | KIAA1671 | −14.099 | 4.945 | 19.043 | 19.348 | −8.780 | 0.413 | 0.025 |  |
| 8063437 | TSHZ2 | teashirt zinc finger homeobox 2 | −7.217 | 13.386 | 20.603 | 11.425 | −0.043 | 0.414 | 0.025 |  |
| 8089954 | IQCB1 | IQ motif containing B1 | 22.946 | 1.329 | −21.617 | −11.156 | −9.083 | 0.414 | 0.025 |  |
| 8046124 | DHRS9 | dehydrogenase/reductase (SDR family) member 9 | −11.293 | 36.107 | 47.400 | 23.433 | 2.476 | 0.414 | 0.025 |  |
| 8063903 | RPS21 | ribosomal protein S21 | 18.264 | 1.941 | −16.324 | −13.249 | −8.232 | 0.415 | 0.025 |  |
| 8103437 | PPID | peptidylprolyl isomerase D | 21.437 | 3.337 | −18.100 | −11.601 | −8.255 | 0.415 | 0.025 |  |
| 7920217 | SPRR2G | small proline-rich protein 2G | 3.792 | −9.560 | −13.352 | −13.852 | −8.619 | 0.416 | 0.025 |  |
| 8124502 | ZNF184 | zinc finger protein 184 | 21.169 | 2.935 | −18.234 | −12.407 | −1.258 | 0.416 | 0.025 |  |
| 7978666 | MBIP | MAP3K12 binding inhibitory protein 1 | 14.314 | −3.785 | −18.099 | −11.004 | −2.191 | 0.416 | 0.025 |  |
| 8084206 | B3GNT5 | UDP-GlcNAc:betaGal beta-1, 3-*N*-acetylglucosaminyltransferase 5 | 31.133 | 10.657 | −20.476 | −14.493 | −4.837 | 0.417 | 0.025 |  |
| 7965855 | NUP37 | nucleoporin 37kDa | 17.065 | 5.322 | −11.743 | −12.972 | −0.659 | 0.417 | 0.025 |  |
| 8041808 | RHOQ | ras homolog gene family member Q | 67.928 | 10.754 | −57.173 | −21.108 | −3.703 | 0.418 | 0.024 |  |
| 8133155 | TPST1 | tyrosylprotein sulfotransferase 1 | 11.071 | −36.590 | −47.661 | −25.100 | −8.333 | 0.418 | 0.025 |  |
| 7959623 | GTF2H3 | general transcription factor IIH, polypeptide 3, 34kDa | 14.119 | 1.621 | −12.499 | −10.333 | −4.017 | 0.418 | 0.025 |  |
| 8043687 | ANKRD36 | ankyrin repeat domain 36 | 69.313 | 16.281 | −53.032 | −16.480 | −2.131 | 0.418 | 0.024 |  |
| 8058182 | FAM126B | family with sequence similarity 126, member B | 121.895 | 2.319 | −119.576 | −12.862 | −7.483 | 0.419 | 0.023 |  |
| 8099395 | RAB28 | RAB28, member RAS oncogene family | 24.694 | 7.119 | −17.576 | −10.893 | −6.067 | 0.419 | 0.025 |  |
| 7990566 | ETFA | electron-transfer-flavoprotein, alpha polypeptide | 41.085 | 2.340 | −38.745 | −10.634 | −6.358 | 0.419 | 0.024 |  |
| 7989493 | RPS27L | ribosomal protein S27-like | 6.756 | −1.430 | −8.186 | −11.847 | −5.526 | 0.420 | 0.025 |  |
| 8047518 | NOP58 | NOP58 ribonucleoprotein | 58.414 | 15.780 | −42.634 | −12.371 | −8.032 | 0.420 | 0.024 |  |
| 8059708 | SNORA75 | small nucleolar RNA, H/ACA box 75 | −6.110 | 18.512 | 24.622 | 11.633 | 4.888 | 0.421 | 0.025 |  |
| 7899187 | HMGN2 | high-mobility group nucleosomal binding domain 2 | 116.758 | 16.653 | −100.105 | −31.908 | −9.838 | 0.421 | 0.024 |  |
| 8119435 | NCR2 | natural cytotoxicity triggering receptor 2 | −7.234 | 0.110 | 7.343 | 11.924 | 4.226 | 0.422 | 0.025 |  |
| 8112615 | ENC1 | ectodermal-neuralcortex 1 (with BTB domain) | −3.376 | 6.323 | 9.699 | 12.355 | −5.434 | 0.422 | 0.025 |  |
| 7953532 | ENO2 | enolase 2 (gamma, neuronal) | −12.900 | 8.078 | 20.978 | 11.791 | 4.153 | 0.423 | 0.025 |  |
| 8107578 | SRFBP1 | serum response factor binding protein 1 | 21.803 | 4.911 | −16.892 | −10.873 | −4.582 | 0.424 | 0.025 |  |
| 8129974 | FUCA2 | fucosidase, alpha-L-2, plasma | 8.085 | −6.556 | −14.641 | −10.382 | −5.107 | 0.424 | 0.025 |  |
| 8030364 | SNORD34 | small nucleolar RNA, C/D box 34 | 15.882 | −3.861 | −19.743 | −19.437 | −1.297 | 0.425 | 0.025 |  |
| 8081853 | POGLUT1 | protein *O*-glucosyltransferase 1 | 12.720 | −5.669 | −18.388 | −14.073 | −6.077 | 0.425 | 0.025 |  |
| 7962455 | NELL2 | neural EGFL like 2 | −7.224 | 44.123 | 51.347 | 10.057 | 6.421 | 0.425 | 0.024 |  |
| 8041360 | TTC27 | tetratricopeptide repeat domain 27 | 24.950 | 7.978 | −16.972 | −11.952 | −2.308 | 0.425 | 0.025 |  |
| 8151587 | C8orf59 | chromosome 8 open reading frame 59 | 11.403 | 1.467 | −9.936 | −16.651 | −7.817 | 0.426 | 0.025 |  |
| 7993664 | CP110 | centriolar coiled coil protein 110kDa | 21.466 | 6.215 | −15.251 | −11.442 | −9.342 | 0.426 | 0.025 |  |
| 7980496 | C14orf145 | chromosome 14 open reading frame 145 | 10.279 | −4.721 | −15.000 | −16.615 | −6.627 | 0.426 | 0.025 |  |
| 8120552 | FAM135A | family with sequence similarity 135, member A | 11.007 | 0.068 | −10.939 | −11.370 | −2.884 | 0.427 | 0.025 |  |
| 7945831 | OSBPL5 | oxysterol binding protein-like 5 | −32.893 | −13.955 | 18.937 | 10.706 | 6.160 | 0.427 | 0.025 |  |
| 7900446 | ZNF642 | zinc finger protein 642 | 6.290 | −0.459 | −6.749 | −11.102 | −3.935 | 0.428 | 0.025 |  |
| 7998666 | SNORA64 | small nucleolar RNA, H/ACA box 64 | −1.363 | −25.034 | −23.672 | −20.925 | −7.197 | 0.428 | 0.025 |  |
| 7977033 | AMN | amnion associated transmembrane protein | −6.804 | 0.152 | 6.955 | 10.160 | 9.725 | 0.428 | 0.025 |  |
| 7900228 | NDUFS5 | NADH dehydrogenase (ubiquinone) Fe-S protein 5, 15kDa (NADH-coenzyme Q reductase) | 69.928 | 9.883 | −60.044 | −15.207 | −5.010 | 0.429 | 0.024 |  |
| 8116610 | NQO2 | NAD(P)H dehydrogenase, quinone 2 | 75.579 | −31.556 | −107.135 | −12.055 | −0.833 | 0.429 | 0.023 |  |
| 8077099 | SCO2 | SCO2 cytochrome C oxidase assembly protein | −5.173 | 5.242 | 10.415 | 11.058 | −1.034 | 0.429 | 0.025 |  |
| 7971661 | MIR15A | microRNA 15a | 57.508 | −13.518 | −71.026 | −11.567 | −9.623 | 0.430 | 0.024 |  |
| 8056829 | CIR1 | corepressor interacting with RBPJ, 1 | 30.171 | 2.191 | −27.980 | −12.209 | −6.162 | 0.431 | 0.025 |  |
| 7906777 | FCGR2B | Fc fragment of IgG, low affinity IIb, receptor (CD32) | 3.158 | −12.678 | −15.836 | −10.619 | 2.117 | 0.431 | 0.025 |  |
| 8103025 | ZNF827 | zinc finger protein 827 | −11.487 | 12.735 | 24.222 | 13.053 | 8.183 | 0.431 | 0.025 |  |
| 8147654 | POLR2K | polymerase (RNA) II (DNA directed) polypeptide K, 7.0kDa | 28.485 | 7.199 | −21.286 | −17.227 | −7.136 | 0.432 | 0.025 |  |
| 8120239 | TMEM14A | transmembrane protein 14A | 9.482 | −0.961 | −10.443 | −13.838 | −4.447 | 0.432 | 0.025 |  |
| 7974380 | PSMC6 | proteasome 26S submit, ATPase 6 | 30.146 | 12.444 | −17.703 | −14.671 | −4.539 | 0.432 | 0.025 |  |
| 7970858 | HMGB1 | high-mobility group box 1 | 49.724 | 17.587 | −32.137 | −11.171 | 1.656 | 0.432 | 0.024 |  |
| 7918394 | EPS8L3 | EPS8-like 3 | 3.592 | −1.601 | −5.193 | −11.149 | −4.506 | 0.432 | 0.025 |  |
| 8055952 | NR4A2 | nuclear receptor subfamily 4, group A, member 2 | −4.147 | 3.581 | 7.728 | 14.229 | 3.374 | 0.433 | 0.025 |  |
| 8115476 | MED7 | mediator complex subunit 7 | 22.047 | 4.073 | −17.975 | −13.385 | −6.121 | 0.433 | 0.025 |  |
| 7915787 | PIK3R3 | phosphoinositide-3-kinase, regulatory subunit 3 (gamma) | −2.181 | 2.768 | 4.949 | 10.055 | 2.528 | 0.433 | 0.025 |  |
| 8014115 | MYO1D | myosin ID | 17.090 | 4.258 | −12.832 | −13.640 | −5.910 | 0.434 | 0.025 |  |
| 8157074 | FKTN | fukutin | 8.767 | 0.166 | −8.601 | −12.406 | −8.991 | 0.434 | 0.025 |  |
| 8070141 | CRYZL1 | crystallin, zeta (quinone reductase)-like 1 | 16.195 | 1.920 | −14.275 | −10.409 | −7.267 | 0.434 | 0.025 |  |
| 7971361 | NUFIP1 | nuclear fragile X mental retardation protein interacting protein 1 | −0.536 | 8.471 | 9.007 | 10.745 | −5.025 | 0.435 | 0.025 |  |
| 7961097 | FLJ46363 | uncharacterized FLJ46363 | −8.698 | −3.194 | 5.504 | 10.844 | 0.423 | 0.435 | 0.025 |  |
| 7929052 | IFIT3 | interferon-induced protein with tetratricopeptide repeats 3 | −65.395 | 32.192 | 97.587 | 30.809 | 6.023 | 0.435 | 0.024 |  |
| 8028233 | HKR1 | HKR1, GLI-Kruppel zinc finger family member | 9.193 | −4.120 | −13.313 | −10.671 | −4.212 | 0.435 | 0.025 |  |
| 7979260 | GMFB | glia maturation factor, beta | 8.770 | −1.680 | −10.450 | −10.444 | −2.869 | 0.436 | 0.025 |  |
| 7966441 | C12orf47 | chromosome 12 open reading frame 47 | 12.519 | 0.529 | −11.990 | −10.465 | −0.949 | 0.436 | 0.025 |  |
| 8135625 | CAPZA2 | capping protein (actin filament) muscle Z-line, alpha 2 | 75.611 | 7.554 | −68.058 | −12.795 | −7.270 | 0.437 | 0.024 |  |
| 8016259 | LRRC37A2 | leucine rich repeat containing 37, member A2 | 39.590 | −24.953 | −64.543 | −10.211 | −1.408 | 0.437 | 0.024 |  |
| 8009008 | METTL2A | methyltransferase like 2A | −3.418 | 9.254 | 12.672 | 15.719 | 6.742 | 0.438 | 0.025 |  |
| 7902992 | RPAP2 | RNA polymerase II associated protein 2 | 21.586 | −0.744 | −22.330 | −10.397 | −5.942 | 0.438 | 0.025 |  |
| 8041508 | QPCT | glutaminyl-peptide cyclotransferase | 35.762 | −12.170 | −47.932 | −13.203 | −5.121 | 0.439 | 0.024 |  |
| 7984330 | ZWILCH | Zwilch kinetochore protein | 10.461 | 0.817 | −9.644 | −10.591 | −6.591 | 0.439 | 0.025 |  |
| 8020455 | GATA6 | GATA binding protein 6 | −7.886 | 1.066 | 8.952 | 11.240 | 5.712 | 0.439 | 0.025 |  |
| 8092765 | C3orf59 | chromosome 3 open reading frame 59 | 6.350 | −5.002 | −11.352 | −10.605 | 2.147 | 0.439 | 0.025 |  |
| 8112668 | GCNT4 | glucosaminyl(*N*-acetyl) transferase 4, core 2 | 30.979 | 12.162 | −18.817 | −10.542 | 1.222 | 0.439 | 0.025 |  |
| 7999447 | LOC400499 | uncharaterized protein LOC400499 | −24.798 | −10.376 | 14.422 | 11.490 | −2.696 | 0.440 | 0.025 |  |
| 7992887 | ZNF75A | zinc finger protein 75a | 20.024 | −3.664 | −23.688 | −17.159 | 1.046 | 0.440 | 0.025 |  |
| 8159439 | C9orf172 | chromosome 9 open reading frame 172 | −14.664 | 0.531 | 15.195 | 13.184 | 8.187 | 0.440 | 0.025 |  |
| 7972487 | DOCK9 | dedicator of cytokinesis 9 | −17.831 | 24.071 | 41.902 | 10.387 | −1.428 | 0.442 | 0.024 |  |
| 8103975 | SLED1 | proteoglycan 3 pseudogene | 28.616 | −4.505 | −33.122 | −11.510 | 1.774 | 0.442 | 0.024 |  |
| 8071554 | CCDC116 | coiled-coil domain containing 116 | −0.820 | −8.005 | −7.185 | −12.128 | 2.866 | 0.443 | 0.025 |  |
| 8140971 | SAMD9L | sterile alpha motif domain containing 9-like | −84.054 | 127.725 | 211.779 | 15.408 | 3.353 | 0.444 | 0.022 |  |
| 8072710 | APOL6 | apolipoprotein L, 6 | −32.323 | 30.026 | 62.350 | 13.628 | 2.610 | 0.445 | 0.024 |  |
| 7972932 | FLJ41484 | hypothetical LOC650669 | 4.156 | −1.381 | −5.537 | −11.391 | −5.582 | 0.446 | 0.025 |  |
| 8118734 | ITPR3 | inositol 1,4,5-triphosphate receptor, type 3 | −27.682 | 6.282 | 33.965 | 13.825 | 6.285 | 0.446 | 0.025 |  |
| 7943892 | NCAM1 | neural cell adhesion molecule 1 | −19.058 | 2.049 | 21.107 | 20.695 | 8.780 | 0.446 | 0.025 |  |
| 8017210 | AP1S2 | adaptor-related protein complex 1, sigma 2 subunit | 45.544 | −16.605 | −62.149 | −10.212 | −6.469 | 0.447 | 0.024 |  |
| 7938133 | DNHD1 | dynein heavy chain domain 1 | −17.110 | −5.419 | 11.692 | 10.391 | 2.983 | 0.447 | 0.025 |  |
| 7952451 | LOC100130428 | IGYY565 | 30.183 | 8.400 | −21.783 | −11.587 | −6.395 | 0.447 | 0.025 |  |
| 7949440 | EHBP1L1 | EH domain binding protein 1-like 1 | −7.245 | −1.617 | 5.627 | 10.389 | 9.557 | 0.448 | 0.025 |  |
| 7921344 | ELL2 | elongation factor, RNA polymerase II, 2 | 12.210 | 3.333 | −8.877 | −10.356 | 0.127 | 0.448 | 0.025 |  |
| 8076673 | LDOC1L | leucine zipper, down-regulated in cancer1-like | −10.345 | 2.729 | 13.073 | 11.300 | 1.012 | 0.449 | 0.025 |  |
| 7990757 | CTSH | cathepsin H | 17.258 | −52.682 | −69.940 | −10.820 | −3.638 | 0.449 | 0.024 |  |
| 7969916 | BIVM | basic, immunoglobulin-like variable motif containing | 3.373 | −3.553 | −6.925 | −12.677 | −2.429 | 0.449 | 0.025 |  |
| 8113286 | RIOK2 | RIO kinase 2 | 16.141 | 2.673 | −13.468 | −12.731 | −8.040 | 0.450 | 0.025 |  |
| 7948249 | SLC43A1 | solute carrier family 43 (amino acid system L transporter), member 1 | −17.420 | 8.663 | 26.083 | 11.064 | 2.786 | 0.451 | 0.025 |  |
| 7929288 | EXOC6 | exocyst complex component 6 | 62.914 | 21.535 | −41.380 | −10.743 | 0.015 | 0.451 | 0.024 |  |
| 8025285 | C19orf59 | chromosome 19 open reading frame 59 | 4.336 | −6.029 | −10.364 | −11.253 | 0.393 | 0.453 | 0.025 |  |
| 7992219 | BAIAP3 | BAI1-associated protein 3 | −4.763 | −14.453 | −9.690 | −11.133 | −7.155 | 0.453 | 0.025 |  |
| 7915485 | EBNA1BP2 | EBNA1 binding protein 2 | 12.189 | 0.454 | −11.735 | −10.289 | −5.267 | 0.454 | 0.025 |  |
| 7967117 | OASL | 2′-5′-oligoadenylate synthetase-like | −39.423 | 12.801 | 52.224 | 24.142 | 3.243 | 0.455 | 0.025 |  |
| 8094228 | BST1 | bone marrow stromal cell antigen 1 | 57.773 | −22.303 | −80.076 | −10.789 | −8.988 | 0.456 | 0.024 |  |
| 8039674 | ZNF154 | zinc finger protein 154 | −6.580 | 7.458 | 14.038 | 10.269 | 2.781 | 0.456 | 0.025 |  |
| 7916910 | LRRC40 | leucine rich repeat containing 40 | 17.006 | 4.036 | −12.970 | −11.476 | −9.097 | 0.458 | 0.025 |  |
| 7973618 | IRF9 | interferon regulatory factor 9 | −28.857 | 34.619 | 63.476 | 10.531 | −0.055 | 0.458 | 0.024 |  |
| 7987165 | AVEN | apoptosis, caspase activation inhibitor | 13.171 | 2.152 | −11.020 | −12.618 | −3.146 | 0.458 | 0.025 |  |
| 7923547 | CHI3L1 | chitinase 3-like 1 (cartilage glycoprotein-39) | −16.311 | –178.860 | −162.549 | −12.148 | 0.582 | 0.458 | 0.022 |  |
| 8096109 | MRPS18C | mitochondrial ribosomal protein S18C | 6.447 | −2.673 | −9.120 | −18.952 | −4.983 | 0.458 | 0.025 |  |
| 8012953 | TRIM16 | tripartite motif-containing 16 | −5.542 | 6.167 | 11.710 | 12.278 | −5.402 | 0.460 | 0.025 |  |
| 8124537 | HIST1H3J | histone cluster 1, H3j | 8.240 | 1.505 | −6.735 | −11.586 | 2.609 | 0.461 | 0.025 |  |
| 7921237 | FCRL5 | Fc receptor-like 5 | 16.548 | 0.970 | −15.578 | −15.371 | −3.003 | 0.462 | 0.025 |  |
| 7950753 | CCDC90B | coiled-coil domain containing 90B | 8.631 | −4.366 | −12.998 | −11.310 | −5.776 | 0.462 | 0.025 |  |
| 7977445 | KIAA0125 | KIAA0125 | 6.656 | 0.081 | −6.575 | −11.904 | 8.399 | 0.463 | 0.025 |  |
| 7945169 | TMEM45B | transmembrane protein 45B | 32.490 | −33.953 | −66.442 | −16.190 | −6.964 | 0.463 | 0.024 |  |
| 8153935 | ZNF252 | zinc finger protein 252 | 5.924 | −2.699 | −8.623 | −14.622 | −7.267 | 0.464 | 0.025 |  |
| 8088458 | FHIT | fragile histidine triad | −10.970 | 15.313 | 26.284 | 10.974 | −1.191 | 0.464 | 0.025 |  |
| 8131140 | LOC100288594 | hypothetical LOC100288594 | −16.878 | 3.388 | 20.266 | 15.086 | 8.044 | 0.464 | 0.025 |  |
| 8124848 | IER3 | immediate early response 3 | −4.295 | −49.057 | −44.763 | −15.678 | 3.926 | 0.464 | 0.024 |  |
| 8179704 | IER3 | immediate early response 3 | −4.295 | −49.057 | −44.763 | −15.678 | 3.926 | 0.464 | 0.024 |  |
| 8125993 | ETV7 | Ets variant 7 | −4.310 | 9.810 | 14.120 | 22.297 | 8.102 | 0.465 | 0.025 |  |
| 8093074 | ZDHHC19 | zinc finger, DHHC-type containing 19 | −2.193 | 5.094 | 7.287 | 12.280 | 7.012 | 0.466 | 0.025 |  |
| 8054004 | SEMA4C | sema domain, immunoglobulin domain (Ig), transmembrane domain (TM) and short cytoplasmic domain, (semaphorin) 4C | −12.006 | 2.742 | 14.749 | 10.804 | 4.275 | 0.466 | 0.025 |  |
| 7978686 | NKX2-8 | NK2 homeobox 8 | −4.107 | 1.909 | 6.017 | 10.490 | 5.061 | 0.466 | 0.025 |  |
| 8124484 | HIST1H2BJ | histone cluster 1, H2bj | 4.064 | −6.473 | −10.537 | −11.499 | −6.612 | 0.467 | 0.025 |  |
| 8099670 | CCDC149 | coiled-coil domain containing 149 | −5.479 | 1.637 | 7.117 | 10.512 | 2.576 | 0.468 | 0.025 |  |
| 7936949 | DHX32 | DEAH (Asp-Glu-Ala-His) box polypeptide 32 | 6.431 | −1.308 | −7.739 | −11.481 | −9.470 | 0.468 | 0.025 |  |
| 7924499 | TLR5 | toll-like receptor 5 | 42.721 | −1.288 | −44.009 | −11.686 | 3.153 | 0.469 | 0.024 |  |
| 8121454 | GPR6 | G protein-coupled receptor 6 | 3.005 | −2.222 | −5.227 | −10.470 | −1.510 | 0.469 | 0.025 |  |
| 7920238 | S100A12 | S100 calcium binding protein A12 | 275.712 | 71.232 | −204.480 | −10.352 | 3.185 | 0.470 | 0.021 |  |
| 7911529 | MXRA8 | matrix-remodelling associated 8 | −8.181 | 4.425 | 12.606 | 10.280 | 6.061 | 0.471 | 0.025 |  |
| 8175768 | PNMA6A | paraneoplastic Ma antigen family member 6A | −4.703 | 2.232 | 6.935 | 10.045 | 8.631 | 0.471 | 0.025 |  |
| 7923958 | C1orf116 | chromosome 1 open reading frame 116 | −2.809 | 4.636 | 7.445 | 13.611 | 2.726 | 0.471 | 0.025 |  |
| 7951068 | CWC15 | CWC15 spliceosome-associated protein homolog | 10.755 | 2.674 | −8.081 | −10.517 | −2.724 | 0.471 | 0.025 |  |
| 8014749 | RPL23 | ribosomal protein L23 | 10.788 | −3.290 | −14.079 | −10.027 | −2.692 | 0.471 | 0.025 |  |
| 8125038 | LY6G6E | lymphocyte antigen 6 complex, locus G6E (psedogene) | −6.513 | 1.799 | 8.312 | 13.166 | 3.393 | 0.472 | 0.025 |  |
| 8178582 | LY6G6E | lymphocyte antigen 6 complex, locus G6E (psedogene) | −6.513 | 1.799 | 8.312 | 13.166 | 3.393 | 0.472 | 0.025 |  |
| 8179810 | LY6G6E | lymphocyte antigen 6 complex, locus G6E (psedogene) | −6.513 | 1.799 | 8.312 | 13.166 | 3.393 | 0.472 | 0.025 |  |
| 8021645 | SERPINB10 | serpin peptidase inhibitor, clade B (ovalbumin), member 10 | 2.369 | −11.222 | −13.591 | −23.993 | −3.547 | 0.473 | 0.025 |  |
| 7935528 | SFRP5 | secreted frizzled-related protein 5 | −1.773 | 4.653 | 6.426 | 10.537 | 5.744 | 0.473 | 0.025 |  |
| 8127841 | PGM3 | phosphoglucomutase 3 | 8.152 | −0.357 | −8.509 | −11.406 | −7.832 | 0.474 | 0.025 |  |
| 8128409 | USP45 | ubiquitin specific peptidase 45 | 9.777 | 2.832 | −6.945 | −10.385 | −9.532 | 0.474 | 0.025 |  |
| 8131292 | RBAK | RB-associated KRAB zinc finger | 13.807 | 1.520 | −12.287 | −13.196 | −6.638 | 0.474 | 0.025 |  |
| 8039054 | ZNF347 | zinc finger protein 347 | 7.801 | 0.127 | −7.674 | −10.605 | −0.440 | 0.476 | 0.025 |  |
| 7979864 | ERH | enhancer of rudimentary homolog (Drosophila) | 124.656 | 31.416 | −93.240 | −10.345 | −3.010 | 0.477 | 0.023 |  |
| 8026292 | IL27RA | interleukin 27 receptor, alpha | 10.705 | 2.970 | −7.735 | −10.429 | −2.953 | 0.477 | 0.025 |  |
| 8060734 | MIR103-2 | microRNA 103-2 | −3.031 | 3.642 | 6.673 | 10.081 | −8.953 | 0.478 | 0.025 |  |
| 8123760 | LY86-AS | LY86 antisense RNA 1 | −2.707 | 4.503 | 7.210 | 14.667 | −9.159 | 0.480 | 0.025 |  |
| 8100179 | NFXL1 | nuclear transcription factor, X-box binding-like 1 | 37.515 | −6.947 | −44.462 | −15.067 | −8.158 | 0.480 | 0.024 |  |
| 8161839 | C9orf95 | chromosome 9 open reading frame 95 | 18.259 | 3.460 | −14.800 | −14.463 | −0.760 | 0.481 | 0.025 |  |
| 8032682 | MATK | megakaryocyte-associated tyrosine kinase | 13.052 | −0.870 | −13.923 | −13.135 | 8.213 | 0.481 | 0.025 |  |
| 8075182 | XBP1 | X-box binding protein 1 | 161.741 | 42.593 | −119.147 | −12.677 | 7.658 | 0.481 | 0.023 |  |
| 8019699 | KRTAP4-11 | keratin associated protein 4-11 | −8.460 | 3.679 | 12.140 | 21.321 | 4.181 | 0.482 | 0.025 |  |
| 8008885 | MIR21 | microRNA 21 | −73.497 | −5.958 | 67.538 | 10.518 | −9.063 | 0.482 | 0.024 |  |
| 8049942 | NEU4 | sialidase 4 | −4.950 | 1.302 | 6.252 | 11.812 | 7.379 | 0.484 | 0.025 |  |
| 8159992 | ERMP1 | endoplasmic reticulum metallopeptidase 1 | −15.702 | 4.167 | 19.869 | 10.369 | 0.070 | 0.484 | 0.025 |  |
| 7961198 | KLRAP1 | killer cell lectin-like receptor subfamily A pseudogene 1 | 8.047 | −2.777 | −10.824 | −15.907 | 1.795 | 0.485 | 0.025 |  |
| 7939072 | METT5D1 | methyltransferase like 15 | 29.129 | 5.866 | −23.263 | −31.241 | −1.117 | 0.485 | 0.025 |  |
| 8171747 | EIF1AX | eukaryotic translation initiation factor 1A, X-linked | 40.287 | 10.400 | −29.887 | −11.023 | −3.547 | 0.486 | 0.024 |  |
| 7937508 | CD151 | CD151 molecule (Raph blood group) | 26.956 | −23.911 | −50.866 | −13.691 | −8.740 | 0.488 | 0.024 |  |
| 7938139 | DNHD1 | dynein heavy chain domain 1 | −13.450 | 3.282 | 16.732 | 20.643 | 8.163 | 0.489 | 0.025 |  |
| 7983469 | SPATA5L1 | spermatogenesis associated 5-like 1 | 8.101 | −1.793 | −9.894 | −11.747 | −8.239 | 0.490 | 0.025 |  |
| 8027323 | ZNF257 | zinc finger protein 257 | 7.358 | −0.642 | −8.000 | −16.579 | −4.439 | 0.490 | 0.025 |  |
| 8043981 | IL1R2 | interleukin 1 receptor, type II | 37.093 | −69.929 | −107.022 | −12.580 | −3.360 | 0.490 | 0.023 |  |
| 8096335 | HERC6 | HECT and RLD6 domain containing E3 ubiquitin protein ligase family member 6 | −11.341 | 13.987 | 25.327 | 15.600 | 1.166 | 0.490 | 0.025 |  |
| 8068883 | CRYAA | crystallin, alpha A | −14.123 | −1.782 | 12.341 | 11.223 | 5.125 | 0.491 | 0.025 |  |
| 8052331 | PNPT1 | polyribonucleotide nucleotidyltransferase 1 | −8.815 | 5.180 | 13.996 | 13.456 | −0.686 | 0.491 | 0.025 |  |
| 7976621 | VRK1 | vaccinia related kinase 1 | 28.191 | 8.912 | −19.280 | −12.905 | −5.947 | 0.491 | 0.025 |  |
| 7901549 | CPT2 | carnitine palmitoyltransferase 2 | 33.405 | 3.949 | −29.456 | −20.869 | −2.599 | 0.491 | 0.025 |  |
| 8134452 | BHLHA15 | basichelix-loop-helix family, member A15 | 34.916 | 10.086 | −24.830 | −18.146 | 7.293 | 0.491 | 0.025 |  |
| 8112803 | LHFPL2 | lipoma HMGIC fusion partner-like 2 | −15.925 | 11.018 | 26.943 | 15.288 | −8.564 | 0.491 | 0.025 |  |
| 7926105 | GATA3 | GATA binding protein 3 | −27.254 | 11.459 | 38.712 | 11.114 | 4.098 | 0.492 | 0.024 |  |
| 7927305 | AGAP5 | ArfGAP with GTPase domain, ankyrin repeat and PH domain 5 | 4.812 | −22.951 | −27.763 | −10.962 | −3.572 | 0.492 | 0.025 |  |
| 7961546 | EPS8 | epidermal growth factor receptor pathway substrate 8 | −4.397 | 4.321 | 8.718 | 19.067 | −4.937 | 0.493 | 0.025 |  |
| 8036351 | ZNF850 | zinc finger protein 850 | 6.685 | −3.748 | −10.433 | −10.088 | −6.573 | 0.493 | 0.025 |  |
| 8157939 | ZBTB34 | zinc finger and BTB domain containing 34 | 24.762 | −22.536 | −47.298 | −10.229 | −2.116 | 0.493 | 0.024 |  |
| 8044804 | DBI | diazepam binding inhibitor (GABA receptor modulator, acyl-CoA binding protein) | 7.124 | 0.770 | −6.353 | −10.274 | 0.750 | 0.496 | 0.025 |  |
| 8083887 | CLDN11 | claudin 11 | −1.999 | 4.126 | 6.126 | 10.236 | 7.884 | 0.497 | 0.025 |  |
| 8084917 | LOC440993 | hypothetical LOC440993 | −14.225 | 5.200 | 19.425 | 10.123 | −4.889 | 0.497 | 0.025 |  |
| 8166989 | ZNF673 | KRAB box domain containing 4 | 10.875 | −5.055 | −15.930 | −12.666 | −6.182 | 0.498 | 0.025 |  |
| 7897522 | RBP7 | retinol binding protein 7, cellular | 11.640 | 2.214 | −9.426 | −11.877 | −6.036 | 0.498 | 0.025 |  |
| 8162276 | NFIL3 | nuclear factor, interleukin 3 regulated | 39.032 | −8.547 | −47.579 | −11.454 | −2.692 | 0.499 | 0.024 |  |
| 7913965 | ZNF683 | zinc finger protein 683 | −9.316 | 0.724 | 10.040 | 12.217 | 2.065 | 0.499 | 0.025 |  |
| 8085431 | NUP210 | nucleoporin 210 kDa | –121.360 | −33.839 | 87.521 | 11.571 | 0.202 | 0.500 | 0.024 |  |
| 7989915 | TIPIN | TIMELESS interacting protein | 11.303 | 5.073 | −6.230 | −13.339 | −4.837 | 0.501 | 0.025 |  |
| 8135922 | METTL2B | methyltransferase like 2B | 2.048 | −6.901 | −8.949 | −11.344 | −2.551 | 0.502 | 0.025 |  |
| 8042052 | RPS27A | ribosomal protein S27a | 50.318 | −8.047 | −58.365 | −14.508 | 0.673 | 0.503 | 0.024 |  |
| 7972888 | PCID2 | PCI domain containing 2 | −35.486 | −4.415 | 31.071 | 10.092 | 3.814 | 0.504 | 0.024 |  |
| 8014100 | C17orf75 | chromosome 17 open reading frame 75 | 7.717 | −0.270 | −7.987 | −11.836 | −9.535 | 0.504 | 0.025 |  |
| 8014969 | GJD3 | Gap junction protein, delta 3, 31.9 kDa | −4.394 | 6.287 | 10.681 | 12.026 | 9.838 | 0.505 | 0.025 |  |
| 8083757 | NMD3 | NMD3 ribosome export adaptor | 27.249 | 10.355 | −16.894 | −12.080 | −4.485 | 0.505 | 0.025 |  |
| 7944867 | SIAE | sialic acid acetylesterase | 0.973 | 32.513 | 31.540 | 11.903 | 2.876 | 0.506 | 0.025 |  |
| 7976571 | C14orf129 | chromosome 14 open reading frame 129 | 8.993 | 1.493 | −7.500 | −12.559 | −0.569 | 0.508 | 0.025 |  |
| 8158317 | SPTAN1 | spectrin, alpha, non-erythrocytic 1 | −68.155 | −10.846 | 57.309 | 11.352 | −8.696 | 0.509 | 0.024 |  |
| 7970569 | SACS | sacsin molecular chaperone | 1.857 | 16.840 | 14.983 | 10.653 | 2.183 | 0.509 | 0.025 |  |
| 8095333 | EXOC5 | exocyst complex component 5 | 15.741 | 4.592 | −11.149 | −11.909 | −0.666 | 0.511 | 0.025 |  |
| 8171001 | FUNDC2 | FUN14 domain containing 2 | 26.585 | 0.434 | −26.151 | −14.034 | −4.940 | 0.512 | 0.025 |  |
| 8026339 | SNRPG | small nuclear ribonucleoprotein polypeptide G | 4.621 | −3.670 | −8.291 | −11.844 | −8.494 | 0.512 | 0.025 |  |
| 7952805 | LOC283174 | hypothetical LOC283174 | −7.734 | −0.281 | 7.453 | 11.653 | −3.072 | 0.514 | 0.025 |  |
| 8009243 | C17orf60 | chromosome 17 open reading frame 60 | 26.378 | −10.527 | −36.905 | −14.980 | −5.618 | 0.516 | 0.025 |  |
| 7969096 | CDADC1 | cytidine and DCMP deaminase domain containing 1 | 10.164 | 1.167 | −8.997 | −10.090 | −8.259 | 0.516 | 0.025 |  |
| 8090018 | PARP9 | poly (ADP-ribose) polymerase family, member 9 | −33.608 | 61.140 | 94.748 | 16.491 | 0.080 | 0.517 | 0.024 |  |
| 7916984 | MIR186 | microRNA1 86 | 10.378 | −1.898 | −12.276 | −13.516 | −2.403 | 0.518 | 0.025 |  |
| 8133770 | CCDC146 | coiled-coil domain containing 146 | 5.308 | 29.819 | 24.511 | 12.111 | −1.357 | 0.520 | 0.025 |  |
| 8153424 | C8orf73 | chromosome 8 open reading frame 73 | −7.512 | −0.015 | 7.497 | 10.583 | −3.868 | 0.521 | 0.025 |  |
| 7961102 | CLEC1B | C-type lectin domain family 1, member B | 2.591 | −4.869 | −7.460 | −10.687 | −6.107 | 0.521 | 0.025 |  |
| 8148512 | FLJ43860 | FLJ43860 protein | −6.644 | 0.517 | 7.161 | 11.157 | 2.990 | 0.522 | 0.025 |  |
| 8169249 | MID2 | midline 2 | −0.913 | 11.970 | 12.883 | 10.659 | 0.459 | 0.523 | 0.025 |  |
| 8019565 | KRTAP4-12 | keratin associated protein 4-12 | −11.997 | 0.020 | 12.018 | 15.537 | 5.601 | 0.523 | 0.025 |  |
| 8122818 | C6orf211 | chromosome 6 open reading frame 211 | 17.663 | 2.527 | −15.136 | −10.709 | −4.912 | 0.524 | 0.025 |  |
| 8022747 | B4GALT6 | UDP-Gal:betaGlcNAc beta 1, 4-galactosyltransferase, polypeptide 6 | 5.384 | −1.822 | −7.207 | −11.169 | 4.591 | 0.525 | 0.025 |  |
| 8178897 | COL11A2 | collagen, typeXI, alpha 2 | 5.509 | −3.107 | −8.616 | −16.404 | −2.142 | 0.526 | 0.025 |  |
| 8125568 | COL11A2 | collagen, typeXI, alpha 2 | 5.509 | −3.107 | −8.616 | −14.180 | 8.433 | 0.526 | 0.025 |  |
| 8013157 | TOM1L2 | target of Myb1 like 2 membrane trafficking protein | −33.658 | −19.351 | 14.306 | 10.813 | 9.905 | 0.527 | 0.025 |  |
| 7956261 | RBMS2 | RNA binding motif, single stranded interacting protein 2 | −3.836 | −18.990 | −15.154 | −11.314 | −1.804 | 0.529 | 0.025 |  |
| 8063444 | TSHZ2 | teashirt zinc finger homeobox 2 | −5.113 | 5.372 | 10.486 | 13.593 | −3.310 | 0.532 | 0.025 |  |
| 8090193 | HEG1 | heart development protein with EGF-like domains 1 | −14.043 | 5.093 | 19.135 | 10.191 | −1.730 | 0.535 | 0.025 |  |
| 8057517 | NCKAP1 | NCK-associated protein 1 | 3.134 | −2.592 | −5.726 | −10.313 | −4.186 | 0.535 | 0.025 |  |
| 7954382 | PYROXD1 | pyridine nucleotide-disulphide oxidoreductase domain 1 | 39.499 | 15.125 | −24.375 | −10.118 | 2.796 | 0.535 | 0.025 |  |
| 8026895 | CCDC124 | coiled-coil domain containing 124 | 25.065 | −19.662 | −44.727 | −11.485 | −6.275 | 0.536 | 0.024 |  |
| 7923173 | MIR181B1 | microRNA 181b-1 | 5.280 | −6.397 | −11.677 | −10.016 | −1.092 | 0.537 | 0.025 |  |
| 7991758 | HBM | hemoglobin, mu | 124.133 | −46.163 | −170.296 | −19.554 | 2.937 | 0.538 | 0.023 |  |
| 8007919 | LRRC37A4 | leucine rich repeat containing 37, member A4, pseudogene | 28.143 | −84.788 | −112.930 | −11.034 | −9.707 | 0.538 | 0.023 |  |
| 7912012 | ACOT7 | acyl-CoA thioesterase 7 | −0.299 | 10.457 | 10.756 | 11.646 | −0.598 | 0.539 | 0.025 |  |
| 7928999 | LIPN | lipase, family member N | 36.879 | 6.431 | −30.448 | −12.311 | 5.076 | 0.539 | 0.025 |  |
| 7995793 | MT1L | metallothionein 1L (gene/pseudogene) | 58.630 | 8.810 | −49.820 | −12.028 | 4.458 | 0.539 | 0.024 |  |
| 8062927 | PI3 | peptidase inhibitor 3, skin-derived | 164.971 | 34.562 | −130.409 | −17.273 | −3.299 | 0.540 | 0.024 |  |
| 7938416 | LOC100129827 | hypothetical LOC100129827 | 1.593 | −14.988 | −16.580 | −10.709 | −1.920 | 0.540 | 0.025 |  |
| 7942771 | FLJ38894 | hypothetical protein LOC646029 | −1.178 | 4.552 | 5.730 | 10.195 | −3.421 | 0.540 | 0.025 |  |
| 7948444 | TCN1 | transcobalamin I (vitamin B12 binding protein, R binder family ) | 39.568 | 10.141 | −29.427 | −17.189 | 1.484 | 0.541 | 0.025 |  |
| 7960874 | C3AR1 | complement component 3a receptor 1 | −2.876 | –102.548 | −99.671 | −14.181 | 0.302 | 0.543 | 0.024 |  |
| 8070579 | TFF1 | trefoil factor1 | 0.398 | −5.230 | −5.628 | −11.395 | −3.613 | 0.545 | 0.025 |  |
| 8039006 | ZNF320 | zinc finger protein 320 | 8.284 | 1.807 | −6.477 | −11.589 | −9.138 | 0.546 | 0.025 |  |
| 7996081 | GPR56 | adhesion G protein-coupled receptor G1 | –122.407 | −3.812 | 118.595 | 20.934 | 8.426 | 0.549 | 0.024 |  |
| 8144239 | FBXO25 | F-box protein 25 | 74.552 | 35.764 | −38.788 | −20.095 | 7.597 | 0.549 | 0.025 |  |
| 8155521 | FAM27A | family with sequence similarity 27, member A | 3.201 | −8.352 | −11.553 | −16.373 | 6.620 | 0.551 | 0.025 |  |
| 8168387 | NCRNA00246B | Family with sequence similarity 226, member B (non-protein coding) | −3.330 | 2.187 | 5.517 | 10.379 | 6.999 | 0.554 | 0.025 |  |
| 7963817 | GTSF1 | gametocyte specific factor 1 | 22.543 | 10.234 | −12.309 | −11.638 | 4.049 | 0.554 | 0.025 |  |
| 8121510 | RPF2 | ribosome production factor 2 homolog | 17.262 | 9.381 | −7.881 | −11.302 | −7.084 | 0.556 | 0.025 |  |
| 7948910 | SNORD25 | small nucleolar RNA, C/D box 25 | 3.248 | −7.745 | −10.993 | −22.338 | −4.724 | 0.556 | 0.025 |  |
| 7978911 | C14orf182 | chromosome 14 open reading frame 182 | 2.371 | −6.660 | −9.031 | −11.537 | −3.685 | 0.558 | 0.025 |  |
| 7912316 | CASZ1 | castor zinc finger 1 | −11.571 | 1.023 | 12.594 | 16.937 | 0.559 | 0.558 | 0.025 |  |
| 7920873 | SNORA42 | small nucleolar RNA, H H/ACA box 80E | −38.254 | −20.653 | 17.601 | 11.517 | 3.328 | 0.560 | 0.025 |  |
| 8133233 | AUTS2 | autism susceptibility candidate 2 | −12.024 | 2.718 | 14.742 | 15.481 | 7.038 | 0.561 | 0.025 |  |
| 8145736 | NRG1 | neuregulin 1 | 10.156 | −3.021 | −13.177 | −15.032 | 1.190 | 0.563 | 0.025 |  |
| 8059244 | CHPF | chondroitin polymerizing factor | 1.821 | 9.172 | 7.351 | 10.135 | 4.195 | 0.564 | 0.025 |  |
| 8052438 | LOC339803 | uncharacterized LOC339803 | 3.505 | −2.316 | −5.821 | −10.732 | 4.547 | 0.565 | 0.025 |  |
| 8100085 | GNPDA2 | glucosamine-6-phosphate deaminase 2 | 13.556 | 2.300 | −11.255 | −10.446 | 1.041 | 0.565 | 0.025 |  |
| 8087691 | CACNA2D2 | calcium channel, voltage-dependent, alpha 2/delta subunit 2 | −12.537 | −0.635 | 11.902 | 14.963 | 7.680 | 0.565 | 0.025 |  |
| 8002322 | PDXDC2P | pyridoxal-dependent decarboxylase domain containing 2, pseudogene | −40.873 | 7.116 | 47.989 | 10.650 | −1.970 | 0.569 | 0.024 |  |
| 8018761 | ST6GALNAC2 | ST6 (alpha-*N*-acetyl-neuraminyl-2,3-beta-galactosyl-1, 3)-*N*-acetylgalactosaminide alpha-2, 6-sialyltransferase2 | 28.675 | −81.784 | −110.459 | −10.419 | −2.189 | 0.570 | 0.023 |  |
| 7928551 | RPS24 | ribosomal protein S24 | −2.066 | 7.076 | 9.143 | 15.157 | 0.499 | 0.570 | 0.025 |  |
| 8173755 | ITM2A | integral membrane protein 2A | 29.555 | 6.895 | −22.660 | −11.928 | −9.569 | 0.570 | 0.025 |  |
| 8151334 | MSC | Musculin | 1.221 | −7.821 | −9.041 | −14.462 | −9.977 | 0.571 | 0.025 |  |
| 8094759 | NSUN7 | NOP2/sun domain family, member 7 | 14.320 | 0.768 | −13.551 | −10.306 | 5.093 | 0.572 | 0.025 |  |
| 8108475 | C5orf53 | chromosome 5 open reading frame 53 | 8.586 | 2.692 | −5.895 | −10.986 | −5.574 | 0.573 | 0.025 |  |
| 8128698 | SESN1 | sestrin 1 | 41.550 | 13.747 | −27.803 | −10.291 | 4.751 | 0.575 | 0.024 |  |
| 7920422 | RAB13 | RAB13, member RAS oncogene family | 4.153 | −3.594 | −7.747 | −11.712 | 7.424 | 0.576 | 0.025 |  |
| 8016245 | LRRC37A4 | leucine rich repeat containing 37, member A4 (pseudogene) | 21.622 | −78.428 | −100.050 | −10.636 | −9.590 | 0.580 | 0.023 |  |
| 7929072 | IFIT5 | interferon-induced protein with tetratricopeptide repeats 5 | −35.501 | 14.422 | 49.923 | 16.778 | 9.268 | 0.580 | 0.024 |  |
| 8122058 | ARG1 | arginase 1 | 5.771 | −9.044 | −14.816 | −15.231 | −2.167 | 0.581 | 0.025 |  |
| 7958784 | ALDH2 | aldehyde dehydrogenase 2 family (mitochondrial) | 9.442 | −52.237 | −61.679 | −10.507 | −8.732 | 0.583 | 0.024 |  |
| 7951413 | CARD17 | caspase recruitment domain family, member 17 | 11.072 | 2.883 | −8.188 | −11.075 | −7.223 | 0.583 | 0.025 |  |
| 8083704 | BRD7 | bromodomain containing 7 | 20.417 | 6.767 | −13.650 | −16.862 | 0.441 | 0.585 | 0.025 |  |
| 7999217 | ROGDI | rogdi homolog | 34.612 | −25.599 | −60.211 | −10.484 | 3.409 | 0.585 | 0.024 |  |
| 8049538 | LRRFIP1 | leucine rich repeat (in FLII) interacting protein 1 | 257.584 | 85.326 | −172.258 | −35.562 | −2.394 | 0.585 | 0.024 |  |
| 8035297 | PLVAP | plasmalemma vesicle associated protein | −8.245 | 24.251 | 32.496 | 15.016 | −0.739 | 0.586 | 0.025 |  |
| 8163839 | C5 | complement component 5 | −3.033 | 2.611 | 5.644 | 11.353 | −8.734 | 0.587 | 0.025 |  |
| 8071044 | MAFIP | MAFF interacting protein (pseudogene) | −5.807 | 26.181 | 31.988 | 21.177 | −0.585 | 0.589 | 0.025 |  |
| 7973298 | TRAV8-3 | T cell receptor alpha variable 8-3 | −2.133 | 7.677 | 9.811 | 12.425 | 7.086 | 0.589 | 0.025 |  |
| 8004241 | RNASEK | ribonuclease, RNase K | 22.170 | 122.959 | 100.789 | 23.410 | 6.533 | 0.594 | 0.024 |  |
| 7977371 | PACS2 | phosphofurin acidic cluster sorting protein 2 | −5.422 | 1.975 | 7.397 | 11.098 | 5.024 | 0.596 | 0.025 |  |
| 8128133 | LYRM2 | LYR motif containing 2 | 37.117 | 7.838 | −29.279 | −21.134 | −3.424 | 0.598 | 0.025 |  |
| 7985192 | AGPHD1 | hydroxylysine kinase | 9.762 | 4.663 | −5.099 | −10.021 | −4.425 | 0.601 | 0.025 |  |
| 8122071 | ENPP3 | ectonucleotide pyrophosphatase/phosphodiesterase 3 | 5.706 | 13.384 | 7.678 | 13.201 | −4.393 | 0.604 | 0.025 |  |
| 8139121 | TRGV3 | T cell receptor gamma variable 3 | −7.753 | 21.707 | 29.460 | 10.947 | 4.389 | 0.606 | 0.024 |  |
| 8158059 | STXBP1 | syntaxin binding protein 1 | −4.094 | 19.955 | 24.050 | 13.696 | 6.936 | 0.607 | 0.025 |  |
| 7992670 | PDPK1 | 3-phosphoinositide dependent protein kinase-1 | –102.560 | −45.574 | 56.986 | 11.310 | 4.249 | 0.610 | 0.024 |  |
| 7952813 | IGSF9B | immunoglobulin superfamily, member 9B | −18.704 | −8.692 | 10.013 | 13.025 | 2.644 | 0.618 | 0.025 |  |
| 8062339 | SAMHD1 | SAM domain and HD domain 1 | −58.908 | 14.544 | 73.451 | 137.891 | 6.361 | 0.619 | 0.025 |  |
| 7934898 | ANKRD22 | ankyrin repeat domain 22 | −10.874 | 9.085 | 19.959 | 24.824 | −1.007 | 0.620 | 0.025 |  |
| 7917649 | TGFBR3 | transforming growth factor, beta receptor III | −36.245 | 19.463 | 55.708 | 19.234 | 1.550 | 0.623 | 0.024 |  |
| 8135931 | FLJ45340 | hypothetical LOC402483 | –109.670 | −19.760 | 89.910 | 18.575 | −4.469 | 0.624 | 0.024 |  |
| 7903010 | RPL5 | ribosomal protein L5 | 401.787 | 192.354 | −209.433 | −51.891 | −5.389 | 0.627 | 0.024 |  |
| 7905746 | AQP10 | aquaporin 10 | −0.009 | −8.408 | −8.399 | −14.258 | −8.923 | 0.628 | 0.025 |  |
| 7984704 | NEO1 | neogenin 1 | −8.935 | 2.036 | 10.971 | 10.670 | −3.777 | 0.630 | 0.025 |  |
| 8149345 | CTSB | cathepsin B | 36.064 | −95.784 | −131.849 | −116.551 | 4.242 | 0.630 | 0.025 |  |
| 7977270 | LOC388022 | Uncharacterized LOC388022 | 14.017 | 5.869 | −8.148 | −14.368 | −5.543 | 0.632 | 0.025 |  |
| 8091327 | PLSCR1 | phospholipid scramblase 1 | 1.610 | 69.049 | 67.440 | 14.280 | −6.952 | 0.632 | 0.024 |  |
| 8117594 | HIST1H2BM | histone cluster 1, H2bm | 78.689 | 36.029 | −42.660 | −12.752 | −7.711 | 0.638 | 0.024 |  |
| 8005955 | SNORD42A | small nucleolar RNA, C/D box 42A | 8.084 | −1.182 | −9.266 | −11.644 | −8.994 | 0.640 | 0.025 |  |
| 8070389 | HMGN1 | high-mobility group nucleosome binding domain 1 | −18.704 | 2.914 | 21.618 | 10.517 | 0.778 | 0.642 | 0.025 |  |
| 8142452 | TFEC | transcription factor EC | 12.980 | −2.054 | −15.034 | −10.091 | −6.993 | 0.647 | 0.025 |  |
| 8032815 | UBXN6 | UBX domain protein 6 | 53.952 | −52.760 | −106.712 | −11.092 | −6.794 | 0.653 | 0.023 |  |
| 8131666 | ITGB8 | integrin, beta 8 | 4.732 | 12.148 | 7.416 | 16.739 | −2.294 | 0.655 | 0.025 |  |
| 7912511 | KIAA2013 | KIAA2013 | −19.349 | −35.341 | −15.993 | −10.145 | 7.091 | 0.658 | 0.025 |  |
| 7907156 | XCL1 | chemokine (C motif) ligand 1 | −3.942 | 8.151 | 12.094 | 19.467 | −2.088 | 0.666 | 0.025 |  |
| 8064432 | FKBP1A | FK506 binding protein 1A, 12kDa | 17.535 | −22.393 | −39.928 | −15.141 | −0.044 | 0.668 | 0.025 |  |
| 8167360 | GATA1 | GATA binding protein 1 (globin transcription factor 1) | 23.919 | −14.149 | −38.068 | −12.566 | −2.354 | 0.669 | 0.024 |  |
| 8016932 | MPO | myeloperoxidase | −5.047 | −13.907 | −8.860 | −10.197 | −4.693 | 0.669 | 0.025 |  |
| 7961151 | KLRK1 | killer cell lectin-like receptor subfamily K, member 1 | 42.462 | −42.564 | −85.025 | −10.513 | −7.243 | 0.676 | 0.023 |  |
| 7922200 | SELP | selectin P (granule membrane protein 140kDa, antigen CD62) | −17.498 | 6.029 | 23.527 | 11.906 | −7.620 | 0.676 | 0.025 |  |
| 8066493 | SLPI | Secretory leukocyte peptidase inhibitor | 55.545 | 25.238 | −30.307 | −12.920 | −8.085 | 0.679 | 0.025 |  |
| 8014298 | C17orf66 | chromosome 17 open reading frame 66 | −6.692 | 0.533 | 7.224 | 11.206 | 5.109 | 0.682 | 0.025 |  |
| 8139203 | C7orf11 | chromosome 7 open reading frame 11 | −93.283 | 24.269 | 117.551 | 100.250 | −7.462 | 0.684 | 0.025 |  |
| 7980773 | CCDC88C | coiled-coil domain containing 88C | −49.808 | 34.023 | 83.831 | 18.323 | 9.864 | 0.691 | 0.024 |  |
| 7915910 | PDZK1IP1 | PDZK1 interacting protein 1 | 31.248 | –124.025 | −155.273 | −10.830 | −9.939 | 0.691 | 0.022 |  |
| 7927108 | BMS1 | BMS1 ribosome biogenesis factor | 23.285 | −3.238 | −26.523 | −20.357 | −6.074 | 0.696 | 0.025 |  |
| 7922648 | TOR1AIP1 | torsin A interacting protein 1 | 33.001 | 10.756 | −22.246 | −17.019 | −6.149 | 0.698 | 0.025 |  |
| 7982206 | GOLGA8IP | golgin A8 family, member I (pseudogene) | −9.722 | 10.691 | 20.413 | 21.510 | 1.504 | 0.699 | 0.025 |  |
| 7987114 | GOLGA8IP | golgin A8 family, member I (pseudogene) | −9.722 | 10.691 | 20.413 | 26.651 | −4.827 | 0.699 | 0.025 |  |
| 8104139 | LOC389834 | ankyrin repeat domain 57 pseudogene | 3.234 | 27.375 | 24.141 | 15.901 | −4.143 | 0.702 | 0.025 |  |
| 8097118 | FLJ45340 | hypothetical LOC402483 | −39.516 | −20.801 | 18.715 | 18.339 | −0.362 | 0.707 | 0.025 |  |
| 8156604 | C9orf102 | chromosome 9 open reading frame 102 | 36.673 | 13.460 | −23.213 | −19.753 | −2.545 | 0.722 | 0.025 |  |
| 7920472 | TPM3 | tropomyosin 3 | 73.168 | −4.775 | −77.943 | −45.919 | −6.231 | 0.724 | 0.025 |  |
| 8074980 | GSTT1 | glutathione S-transferase theta 1 | 8.619 | −0.436 | −9.055 | −10.494 | 1.462 | 0.728 | 0.025 |  |
| 8077728 | TMEM111 | transmembrane protein 111 | 78.303 | 2.273 | −76.030 | −76.491 | −1.450 | 0.731 | 0.025 |  |
| 7933413 | BMS1P1 | BMS1 ribosome biogenesis factor pseudogene 1 | 7.149 | −11.701 | −18.850 | −13.805 | −8.503 | 0.732 | 0.025 |  |
| 7952986 | WNK1 | WNK lysine deficient protein kinase 1 | –109.134 | −6.969 | 102.165 | 22.491 | 3.165 | 0.739 | 0.024 |  |
| 8105331 | GZMK | granzyme K (granzyme 3; tryptase II) | 73.224 | 8.961 | −64.263 | −11.657 | −2.705 | 0.748 | 0.024 |  |
| 7998700 | PKD1 | polycystic kidney disease 1 (autosomal dominant) | −29.842 | −5.920 | 23.922 | 23.248 | 7.746 | 0.759 | 0.025 |  |
| 7913252 | PINK1 | PTEN induced putative kinase 1 | 20.814 | −12.613 | −33.426 | −14.797 | 6.051 | 0.767 | 0.025 |  |
| 8027241 | ZNF253 | zinc finger protein 253 | 71.025 | 98.371 | 27.346 | 18.775 | −4.795 | 0.771 | 0.025 |  |
| 8083260 | CPA3 | carboxypeptidase A3 (mast cell) | 41.282 | 61.800 | 20.518 | 13.276 | 8.015 | 0.775 | 0.025 |  |
| 7898353 | LOC388692 | Uncharacterized LOC388692 | −20.601 | −7.632 | 12.969 | 22.350 | −7.918 | 0.775 | 0.025 |  |
| 8071272 | GP1BB | glyco protein Ib (platelet), beta polypeptide | 14.864 | −0.325 | −15.188 | −20.948 | 1.242 | 0.787 | 0.025 |  |
| 8029854 | SLC1A5 | solute carrier family 1(neutral amino acid transporter), member 5 | 13.288 | −36.656 | −49.944 | −93.820 | −0.822 | 0.828 | 0.025 |  |
| 8141050 | RPS3A | ribosomal protein S3A | 110.027 | 79.605 | −30.422 | −14.116 | −1.601 | 0.848 | 0.025 |  |
| 8128695 | SNX3 | sorting nexin 3 | 29.607 | 4.670 | −24.937 | −42.482 | 4.658 | 0.882 | 0.025 |  |
| 7924491 | AIDA | axin interactor, dorsalization associated | 85.941 | 102.194 | 16.253 | 13.781 | 0.270 | 0.914 | 0.025 |  |
| 7919604 | HIST2H2BF | histone cluster 2, H2bf | −22.419 | −16.411 | 6.008 | 10.676 | 7.123 | 0.950 | 0.025 |  |
| 7939613 | MAPK8IP1 | mitogen-activated protein kinase 8 interacting protein 1 | −23.584 | −14.811 | 8.773 | 14.346 | 5.329 | 0.955 | 0.025 |  |

**Table S2.** Differential blood count in Q-Mix and placebo treated subjects after 10 weeks of supplementation. Average, post-supplementation values in the Q-mix (Q) and placebo (Pbo) groups are indicated in bold for each of the cellular subtypes. Statistical significance of the differences was assessed by a *t*-test.

| **Subject** | **NeutrB (%)** | **LymphB (%)** | **MonoB (%)** | **EosB (%)** | **BasoB (%)** |
| --- | --- | --- | --- | --- | --- |
| Q_Post_9B | 65 | 27 | 7 | 1 | 0 |
| Q_Post_46B | 66 | 22 | 6 | 5 | 1 |
| Q_Post_43B | 59 | 34 | 6 | 1 | 0 |
| Q_Post_36B | 62 | 27 | 8 | 2 | 1 |
| Q_Post_31B | 66 | 27 | 5 | 2 | 0 |
| Q_Post_24B | 58 | 32 | 7 | 2 | 1 |
| Q_Post_14B | 57 | 34 | 6 | 2 | 1 |
| Q_Post_13B | 58 | 33 | 0 | 2 | 1 |
| PBO_Post_8B | 72 | 22 | 4 | 2 | 0 |
| PBO_Post_42B | 50 | 39 | 8 | 2 | 1 |
| PBO_Post_41B | 63 | 29 | 5 | 2 | 1 |
| PBO_Post_33B | 60 | 29 | 8 | 3 | 0 |
| PBO_Post_32B | 53 | 35 | 8 | 4 | 0 |
| PBO_Post_3B | 55 | 29 | 11 | 4 | 1 |
| PBO_Post_25B | 66 | 26 | 5 | 3 | 0 |
| **Average of Placebo P** | **59.8571429** | **29.85714286** | **7** | **2.85714286** | **0.42857143** |
| **Average of Q-Mix Po** | **61.375** | **29.5** | **5.625** | **2.125** | **0.625** |
| ***p*-value (*t*-test)** | **0.6511536** | **0.894155904** | **0.29788444** | **0.21183374** | **0.48428176** |

**Table S3.** Top scoring Reactome pathways from gene-set (pathway) enrichment analysis. Gene expression data was ranked by the percent net change (Q-mix *vs.* placebo) and analyzed via the GSEA tool using the “preranked” option. Pathway names (column 1, Name) are followed by the number of genes in pathway (column 2, Size), a measure of pathway enrichment normalized for the number of genes in the pathway (column 3, NES), nominal *p*-value to assess the significance of pathway enrichment (column 4, p-val), and an assessment of false discoveries due to multiple testing (column 5, FDR).Pathways with a nominal *p* ≤ 0.05 are shown.

| **NAME** | **SIZE** | **NES** | **NOM p-va** | **FDR q-va** | **NetChange** |
| --- | --- | --- | --- | --- | --- |
| REACTOME_INTERFERON_ALPHA_BETA_SIGNALING | 48 | 2.467 | 0.000 | 0.000 | Higher in Q-Mix |
| REACTOME_INTERFERON_SIGNALING | 133 | 2.423 | 0.000 | 0.000 | Higher in Q-Mix |
| REACTOME_ANTIVIRAL_MECHANISM_BY_IFN_STIMULATED_GENES | 63 | 2.325 | 0.000 | 0.000 | Higher in Q-Mix |
| REACTOME_CIRCADIAN_REPRESSION_OF_EXPRESSION_BY_REV_ERBA | 21 | 1.937 | 0.000 | 0.025 | Higher in Q-Mix |
| REACTOME_INTERFERON_GAMMA_SIGNALING | 54 | 1.934 | 0.000 | 0.021 | Higher in Q-Mix |
| REACTOME_TRAF6_MEDIATED_IRF7_ACTIVATION | 17 | 1.926 | 0.000 | 0.020 | Higher in Q-Mix |
| REACTOME_BMAL1_CLOCK_NPAS2_ACTIVATES_CIRCADIAN_EXPRESSION | 33 | 1.750 | 0.003 | 0.125 | Higher in Q-Mix |
| REACTOME_REGULATION_OF_IFNA_SIGNALING | 11 | 1.697 | 0.005 | 0.171 | Higher in Q-Mix |
| REACTOME_SIGNALING_BY_FGFR1_FUSION_MUTANTS | 17 | 1.792 | 0.005 | 0.094 | Higher in Q-Mix |
| REACTOME_RORA_ACTIVATES_CIRCADIAN_EXPRESSION | 23 | 1.737 | 0.006 | 0.131 | Higher in Q-Mix |
| REACTOME_EFFECTS_OF_PIP2_HYDROLYSIS | 17 | 1.793 | 0.006 | 0.105 | Higher in Q-Mix |
| REACTOME_SIGNALING_BY_FGFR1_MUTANTS | 20 | 1.756 | 0.006 | 0.129 | Higher in Q-Mix |
| REACTOME_TRIGLYCERIDE_BIOSYNTHESIS | 33 | 1.581 | 0.011 | 0.336 | Higher in Q-Mix |
| REACTOME_RESPONSE_TO_ELEVATED_PLATELET_CYTOSOLIC_CA2_ | 60 | 1.514 | 0.016 | 0.363 | Higher in Q-Mix |
| REACTOME_SIGNALING_BY_FGFR_MUTANTS | 27 | 1.619 | 0.018 | 0.288 | Higher in Q-Mix |
| REACTOME_NUCLEAR_RECEPTOR_TRANSCRIPTION_PATHWAY | 24 | 1.580 | 0.019 | 0.319 | Higher in Q-Mix |
| REACTOME_TRANSCRIPTIONAL_REGULATION_OF_WHITE_ADIPOCYTE_DIFFERENTIATION | 61 | 1.527 | 0.024 | 0.366 | Higher in Q-Mix |
| REACTOME_GROWTH_HORMONE_RECEPTOR_SIGNALING | 18 | 1.614 | 0.031 | 0.281 | Higher in Q-Mix |
| REACTOME_PLATELET_ACTIVATION_SIGNALING_AND_AGGREGATION | 156 | 1.367 | 0.032 | 0.487 | Higher in Q-Mix |
| REACTOME_G_ALPHA_Q_SIGNALLING_EVENTS | 90 | 1.396 | 0.033 | 0.445 | Higher in Q-Mix |
| REACTOME_COMPLEMENT_CASCADE | 13 | 1.566 | 0.040 | 0.313 | Higher in Q-Mix |
| REACTOME_TRAF3_DEPENDENT_IRF_ACTIVATION_PATHWAY | 13 | 1.578 | 0.042 | 0.306 | Higher in Q-Mix |
| REACTOME_PREFOLDIN_MEDIATED_TRANSFER_OF_SUBSTRATE_TO_CCT_TRIC | 24 | 1.565 | 0.042 | 0.301 | Higher in Q-Mix |
| REACTOME_NEPHRIN_INTERACTIONS | 14 | 1.664 | 0.044 | 0.216 | Higher in Q-Mix |
| REACTOME_RIG_I_MDA5_MEDIATED_INDUCTION_OF_IFN_ALPHA_BETA_PATHWAYS | 58 | 1.426 | 0.057 | 0.401 | Higher in Q-Mix |
| REACTOME_TRANSLATION | 141 | −1.687 | 0.002 | 0.869 | Lower in Q-Mix |
| REACTOME_METABOLISM_OF_PORPHYRINS | 13 | −1.695 | 0.003 | 1.000 | Lower in Q-Mix |
| REACTOME_AMYLOIDS | 54 | −1.796 | 0.004 | 1.000 | Lower in Q-Mix |
| REACTOME_SRP_DEPENDENT_COTRANSLATIONAL_ PROTEIN _TARGETING_TO_MEMBRANE | 105 | −1.648 | 0.008 | 0.808 | Lower in Q-Mix |
| REACTOME_TOLL_RECEPTOR_CASCADES | 107 | −1.623 | 0.010 | 0.737 | Lower in Q-Mix |
| REACTOME_STRIATED_MUSCLE_CONTRACTION | 12 | −1.651 | 0.011 | 0.946 | Lower in Q-Mix |
| REACTOME_PEPTIDE_CHAIN_ELONGATION | 82 | −1.565 | 0.011 | 0.668 | Lower in Q-Mix |
| REACTOME_3_UTR_MEDIATED_TRANSLATIONAL_REGULATION | 101 | −1.545 | 0.014 | 0.604 | Lower in Q-Mix |
| REACTOME_INFLUENZA_VIRAL_RNA_TRANSCRIPTION_AND_REPLICATION | 98 | −1.562 | 0.016 | 0.636 | Lower in Q-Mix |
| REACTOME_PKB_MEDIATED_EVENTS | 24 | −1.703 | 0.017 | 1.000 | Lower in Q-Mix |
| REACTOME_NONSENSE_MEDIATED_DECAY_ENHANCED_BY_THE_EXON_JUNCTION_COMPLEX | 101 | −1.491 | 0.019 | 0.459 | Lower in Q-Mix |
| REACTOME_RNA_POL_I_PROMOTER_OPENING | 46 | −1.571 | 0.020 | 0.686 | Lower in Q-Mix |
| REACTOME_INSULIN_RECEPTOR_SIGNALLING_CASCADE | 57 | −1.525 | 0.020 | 0.507 | Lower in Q-Mix |
| REACTOME_RNA_POL_I_TRANSCRIPTION | 69 | −1.549 | 0.020 | 0.620 | Lower in Q-Mix |
| REACTOME_ACTIVATED_AMPK_STIMULATES_FATTY_ACID_OXIDATION_IN_MUSCLE | 14 | −1.635 | 0.021 | 0.771 | Lower in Q-Mix |
| REACTOME_REGULATION_OF_AMPK_ACTIVITY_VIA_LKB1 | 11 | −1.618 | 0.022 | 0.679 | Lower in Q-Mix |
| REACTOME_EXTRACELLULAR_MATRIX_ORGANIZATION | 33 | −1.537 | 0.025 | 0.576 | Lower in Q-Mix |
| REACTOME_DEPOSITION_OF_NEW_CENPA_CONTAINING_NUCLEOSOMES_AT_THE_CENTROMERE | 43 | −1.591 | 0.026 | 0.695 | Lower in Q-Mix |
| REACTOME_PURINE_METABOLISM | 28 | −1.591 | 0.027 | 0.638 | Lower in Q-Mix |
| REACTOME_FORMATION_OF_THE_TERNARY_COMPLEX_AND_SUBSEQUENTLY_THE_43S_COMPLEX | 47 | −1.537 | 0.027 | 0.551 | Lower in Q-Mix |
| REACTOME_ACTIVATED_TLR4_SIGNALLING | 85 | −1.496 | 0.028 | 0.458 | Lower in Q-Mix |
| REACTOME_SIGNALING_BY_WNT | 62 | −1.520 | 0.029 | 0.468 | Lower in Q-Mix |
| REACTOME_ACTIVATION_OF_THE_MRNA_UPON_BINDING_OF_THE_CAP_BINDING_COMPLEX_AND_EIFS_AND_SUBSEQUENT_BINDING_TO | 54 | −1.500 | 0.029 | 0.489 | Lower in Q-Mix |
| REACTOME_PACKAGING_OF_TELOMERE_ENDS | 38 | −1.550 | 0.032 | 0.655 | Lower in Q-Mix |
| REACTOME_PI3K_CASCADE | 44 | −1.531 | 0.032 | 0.522 | Lower in Q-Mix |
| REACTOME_INFLUENZA_LIFE_CYCLE | 131 | −1.404 | 0.035 | 0.517 | Lower in Q-Mix |
| REACTOME_MEIOTIC_RECOMBINATION | 60 | −1.482 | 0.036 | 0.440 | Lower in Q-Mix |
| REACTOME_REGULATION_OF_ORNITHINE_DECARBOXYLASE_ODC | 46 | −1.515 | 0.037 | 0.467 | Lower in Q-Mix |
| REACTOME_ENERGY_DEPENDENT_REGULATION_OF_MTOR_BY_LKB1_AMPK | 14 | −1.597 | 0.037 | 0.727 | Lower in Q-Mix |
| REACTOME_ADVANCED_GLYCOSYLATION_ENDPRODUCT_RECEPTOR_SIGNALING | 12 | −1.520 | 0.039 | 0.486 | Lower in Q-Mix |
| REACTOME_VIF_MEDIATED_DEGRADATION_OF_APOBEC3G | 48 | −1.481 | 0.040 | 0.430 | Lower in Q-Mix |
| REACTOME_SIGNALING_BY_INSULIN_RECEPTOR | 72 | −1.486 | 0.041 | 0.440 | Lower in Q-Mix |
| REACTOME_PURINE_SALVAGE | 12 | −1.528 | 0.041 | 0.515 | Lower in Q-Mix |
| REACTOME_TRAF6_MEDIATED_INDUCTION_OF_TAK1_COMPLEX | 12 | −1.544 | 0.043 | 0.579 | Lower in Q-Mix |
| REACTOME_PYRUVATE_METABOLISM | 18 | −1.534 | 0.043 | 0.537 | Lower in Q-Mix |
| REACTOME_SCFSKP2_MEDIATED_DEGRADATION_OF_P27_P21 | 50 | −1.480 | 0.045 | 0.423 | Lower in Q-Mix |
| REACTOME_ACTIVATION_OF_IRF3_IRF7_MEDIATED_BY_TBK1_IKK_EPSILON | 12 | −1.520 | 0.045 | 0.502 | Lower in Q-Mix |
| REACTOME_ACTIVATION_OF_CHAPERONES_BY_ATF6_ALPHA | 10 | −1.512 | 0.045 | 0.463 | Lower in Q-Mix |
| REACTOME_P53_INDEPENDENT_G1_S_DNA_DAMAGE_CHECKPOINT | 44 | −1.499 | 0.046 | 0.476 | Lower in Q-Mix |
| REACTOME_CYCLIN_E_ASSOCIATED_EVENTS_DURING_G1_S_TRANSITION_ | 57 | −1.426 | 0.047 | 0.504 | Lower in Q-Mix |
| REACTOME_SCF_BETA_TRCP_MEDIATED_DEGRADATION_OF_EMI1 | 48 | −1.443 | 0.047 | 0.492 | Lower in Q-Mix |
| REACTOME_METABOLISM_OF_NUCLEOTIDES | 60 | −1.433 | 0.048 | 0.513 | Lower in Q-Mix |
| REACTOME_RNA_POL_I_RNA_POL_III_AND_MITOCHONDRIAL_TRANSCRIPTION | 99 | −1.402 | 0.048 | 0.514 | Lower in Q-Mix |
| REACTOME_DEGRADATION_OF_THE_EXTRACELLULAR_MATRIX | 11 | −1.488 | 0.049 | 0.458 | Lower in Q-Mix |
| REACTOME_TRAFFICKING_AND_PROCESSING_OF_ENDOSOMAL_TLR | 12 | −1.488 | 0.050 | 0.446 | Lower in Q-Mix |
| REACTOME_ACTIVATION_OF_NF_KAPPAB_IN_B_CELLS | 60 | −1.465 | 0.050 | 0.457 | Lower in Q-Mix |
| REACTOME_TELOMERE_MAINTENANCE | 65 | −1.449 | 0.051 | 0.496 | Lower in Q-Mix |

**Table S4.** Over-representation analysis of canonical pathways (*p* < 0.001) identified by Ingenuity Pathway Analysis (IPA). Column 1, name of pathway; column 2, negative log of the p-value of overlap between pathway genes and genes from experiment; column 3, ratio of experimentally observed genes to total number of genes for a pathway’ column 4, z-score (when available) predicting activation/inhibition of the pathway based on the consistency of gene expression changes; column 5, component genes contributing to pathway over-representation.

| **Ingenuity Canonical Pathways** | **-log(*p*-Value)** | **Ratio** | | | ***z*-Score** | **Molecules** |
| --- | --- | --- | --- | --- | --- | --- |
| EIF2 Signaling | 2.32 × 10^1^ | 0.405 | | | −3.015 | EIF2AK2, SOS2, EIF3G, RPL13A, PIK3R5, RPL37A, RPS5, PIK3CB, RPS13, PIK3CD, RPS11, RPL29, AGO2, RPL14, RPL19, RPL39, RPS6, RPS18, RPLP2, RPL35, PABPC1, EIF3L, EIF4G2, EIF5, RPL7, NRAS, RPLP0, RPL36, PPP1CB, EIF3K, RPS27A, RPL15, RPS15, GSK3B, EIF2S1, PPP1CC, EIF3M, RPS10, RPS26, RPL35A, PIK3R1, RPL12, RPL5, EIF2AK1, RPL28, RPS15A, RPL37, RPL3, GRB2, RPS8, RPL41, RPS3A, RPL18A, MAPK3, RPS25, KRAS, EIF1AX, RPL21, ATM, RPS2, AKT2, RPL23A, RPS3, EIF2S3, PDPK1, RPL24, RPL18, RPS17, MAPK1, RPS23, RPL36AL, RPS28, RRAS, RPL10, RPL10A |
| Integrin Signaling | 1.56 × 10^1^ | 0.333 | | | −1.050 | SOS2, ITGAL, PTEN, NEDD9, PIK3R5, PIK3CB, ARPC1B, ZYX, ARPC5, RALB, PIK3CD, ACTB, ARF3, ACTN4, ARHGAP26, TLN1, ARPC3, CRKL, MYL9, ACTN1, PPP1R12B, ARPC2, ARF1, NRAS, BRAF, NCK2, ARF4, PPP1CB, ITGA4, LIMS1, ARF6, ITGA5, GSK3B, RHOH, TSPAN2, ACTR2, RHOQ, TSPAN7, WIPF1, PIK3R1, RAC2, ARPC4, TSPAN5, GRB2, ITGB3, GIT1, ROCK1, RAP2B, CRK, MAPK8, CAPNS1, MAPK3, KRAS, PPP1R12A, PAK1, ATM, CAPN1, MYL12B, ITGA6, MYL12A, AKT2, ACTR3, PLCG2, MAPK1, PLCG1, RRAS, RAC1 |
| Glucocorticoid Receptor Signaling | 1.41 × 10^1^ | 0.287 | | | NaN | POU2F1, KAT2B, HSPA1L, HSPA1A/HSPA1B, SOS2, TGFB1, NFATC2, FCGR1A, POLR2A, JAK3, PIK3R5, IL1R2, SMAD4, PRKACB, HSPA6, NFKBIA, PIK3CB, GTF2A2, STAT3, PIK3CD, HMGB1, MED14, ACTB, SMAD3, POLR2J2/POLR2J3, CD3E, NCOA1, CCL5, IL1B, HSP90B1, JAK1, SMARCC2, TAF7, GTF2A1, ANXA1, NRAS, GTF2F2, MAP3K1, BCL2L1, ARID1A, IKBKE, MAPK14, PIK3R1, YWHAH, FKBP5, TSC22D3, DUSP1, HSPA8, STAT1, STAT5A, GRB2, CREBBP, PHF10, MAPK8, HSP90AA1, MAPK3, KRAS, TSG101, CD3G, TAF2, ATM, SLPI, EP300, PPP3CA, AKT2, RELA, NFATC3, POLR2H, PBX1, HSPA5, BCL2, CEBPB, MAPK1, TAF10, HSP90AB1, CD247, RRAS, RAC1, NCOA2 |
| Role of NFAT in Regulation of the Immune Response | 1.40 × 10^1^ | 0.339 | | | 0.603 | FCGR1B, SOS2, GNA13, NFATC2, FCGR1A, GNG11, GNG7, GNB1, PIK3R5, FCER1G, NFKBIA, PIK3CB, FCGR2C, GNAI3, CSNK1D, LCP2, GNAQ, PIK3CD, HLA-DRB5, CD4, LAT, CD79A, ZAP70, CD3E, GNAZ, HLA-DQB1, HLA-DOB, NRAS, CABIN1, IKBKE, LYN, GSK3B, CSNK1A1, ATF2, CALM1(includesothers), PIK3R1, GNAI2, HLA-DMA, ITK, GRB2, BLNK, MAPK3, KRAS, GNB2L1, CD3G, ATM, PPP3CA, AKT2, PLCG2, RELA, NFATC3, ITPR3, MAPK1, PLCG1, GNG2, CD247, RRAS, FCGR3A/FCGR3B |
| FcÎ³ Receptor-mediated Phagocytosis in Macrophages and Monocytes | 1.37 × 10^1^ | 0.430 | | | −2.214 | HCK, RAB11A, PTEN, ACTR2, FCGR1A, NCF1, FGR, PIK3R1, RAC2, CBL, ARPC1B, ARPC5, LCP2, INPP5D, ACTB, ARPC4, CRK, PRKCH, RAB11B, RPS6KB1, MAPK3, TLN1, ARPC3, PAK1, EZR, FYB, ACTR3, AKT2, VAV1, ARPC2, PRKCD, GAB2, PRKCA, NCK2, MAPK1, PLCG1, ARF6, RAC1, FCGR3A/FCGR3B, LYN |
| Cdc42 Signaling | 1.27 × 10^1^ | 0.329 | | | −1.029 | IQGAP1, HLA-DRB3, LIMK1, FCER1G, ARPC1B, ARPC5, ARHGEF6, HLA-DRB5, HLA-DPA1, HLA-DPB1, CD3E, MYL4, ARPC3, HLA-DQB1, EXOC8, HLA-DRB4, HLA-G, HLA-DOB, MYL9, VAV1, PPP1R12B, ARPC2, PPP1CB, ITGA4, HLA-C, HLA-B, ITGA5, GSK3B, ATF2, MAPK14, ACTR2, WIPF1, HLA-F, APC, EXOC6, CFL1, CLIP1, HLA-DMA, ARPC4, ITK, MAPK8, DIAPH1, HLA-E, LIMK2, CD3G, PPP1R12A, PAK1, MYL12B, MYL12A, B2M, ACTR3, MYL6, HLA-A, MAPK1, CD247 |
| Regulation of eIF4 and p70S6K Signaling | 1.23 × 10^1^ | 0.342 | | | −0.655 | EIF4EBP2, SOS2, EIF3G, PIK3R5, RPS5, PIK3CB, RPS13, PIK3CD, PPP2R5A, RPS11, AGO2, RPS6KB1, RPS6, RPS18, PABPC1, EIF3L, EIF4G2, NRAS, PPP2R5D, ITGA4, EIF3K, RPS27A, ITGA5, RPS15, EIF2S1, EIF3M, MAPK14, RPS10, RPS26, PIK3R1, RPS15A, GRB2, RPS8, RPS3A, PPP2CA, MAPK3, RPS25, KRAS, EIF1AX, ATM, RPS2, AKT2, RPS3, EIF2S3, PDPK1, RPS17, MAPK1, RPS23, RPS28, RRAS |
| CD28 Signaling in T Helper Cells | 1.17 × 10^1^ | 0.364 | | | −0.174 | ACTR2, CALM1 (includes others), NFATC2, PIK3R5, FCER1G, PIK3R1, NFKBIA, PIK3CB, ARPC1B, ARPC5, LCP2, PIK3CD, BCL10, HLA-DMA, ARPC4, HLA-DRB5, ITK, GRB2, CD4, MAPK8, LAT, ZAP70, CD3E, ARPC3, HLA-DQB1, CD3G, PAK1, ATM, HLA-DOB, PPP3CA, ACTR3, AKT2, VAV1, RELA, NFATC3, ARPC2, PDPK1, ITPR3, MAP3K1, PLCG1, CD247, RAC1, IKBKE |
| Antigen Presentation Pathway | 1.14 × 10^1^ | 0.595 | | | NaN | PSMB6, HLA-DPB1, HLA-E, CD74, NLRC5, HLA-DRB3, HLA-G, HLA-DRB4, HLA-DOB, B2M, TAP1, TAPBP, HLA-F, HLAA, TAP2, HLA-DMA, HLA-DRB5, HLA-DPA1, CANX, HLA-C, HLA-B, PSMB9 |
| T Cell Receptor Signaling | 1.09 × 10^1^ | 0.381 | | | NaN | SOS2, CALM1 (includes others), NFATC2, PIK3R5, PIK3R1, NFKBIA, CBL, PIK3CB, RASGRP1, LCP2, CD8A, PIK3CD, BCL10, ITK, GRB2, CD4, MAPK8, LAT, ZAP70, MAPK3, CD3E, KRAS, PAG1, CD3G, ATM, PPP3CA, VAV1, RELA, NFATC3, NRAS, MAP3K1, MAPK1, PLCG1, CD247, RRAS, RAC1, IKBKE |
| Interferon Signaling | 1.07 × 10^1^ | 0. 583 | | | 2.183 | IRF1, OAS1, IRF9, IFITM1, IFNAR2, IFIT3, STAT2, JAK1, IFI6, IFITM3, TAP1, IFNAR1, RELA, IFNGR1, IFIT1, MED14, STAT1, IFITM2, BCL2, MX1, IFI35 |
| iCOS-iCOSL Signaling in T Helper Cells | 1.06 × 10^1^ | 0.361 | | | 1.134 | PTEN, CALM1 (includes others), NFATC2, PLEKHA2, PIK3R5, IL2RB, FCER1G, PIK3R1, NFKBIA, PIK3CB, IL2RG, LCP2, INPP5D, PIK3CD, HLA-DMA, HLA-DRB5, ITK, GRB2, CD4, LAT, ZAP70, CD3E, HLA-DQB1, CD3G, ATM, HLA-DOB, PPP3CA, AKT2, VAV1, RELA, NFATC3, PDPK1, GAB2, ITPR3, TRAT1, PLCG1, CD247, RAC1, IKBKE |
| FLT3 Signaling in Hematopoietic Progenitor Cells | 1.05 × 10^1^ | 0.419 | | | NaN | FLT3LG, SOS2, ATF2, MAPK14, PIK3R5, PIK3R1, CBL, PIK3CB, STAT3, INPP5D, PIK3CD, STAT1, STAT5A, GRB2, CREBBP, RPS6KB1, MAPK3, KRAS, RPS6KA3, ATM, STAT2, EP300, AKT2, NRAS, PDPK1, ATF4, GAB2, STAT6, MAPK1, RPS6KA1, RRAS |
| B Cell Receptor Signaling | 1.02 × 10^1^ | 0.299 | | | −0.980 | SOS2, PTEN, NFATC2, PIK3R5, NFKBIA, PIK3CB, FCGR2C, PIK3CD, INPP5B, PIK3AP1, BCL10, CD79A, RPS6KB1, PAG1, VAV1, BCL6, NRAS, MAP3K1, BCL2L1, ETS1, IKBKE, LYN, GSK3B, ATF2, MAPK14, CALM1 (includes others), PIK3R1, RAC2, CFL1, INPP5D, GRB2, CREBBP, APBB1IP, RAP2B, MAPK8, BLNK, MAPK3, KRAS, ATM, EP300, PPP3CA, AKT2, PLCG2, RELA, NFATC3, PDPK1, ATF4, GAB2, MAP3K2, MAPK1, RRAS, RAC1 |
| Actin Cytoskeleton Signaling | 1.01× 10^1^ | 0.276 | | | −0.944 | IQGAP1, SOS2, GNA13, LIMK1, PIK3R5, PIK3CB, ARPC1B, MYH9, ARPC5, ARHGAP24, ARHGEF6, PIK3CD, ACTB, ACTN4, MYL4, TLN1, ARPC3, CRKL, CD14, FLNA, EZR, MYL9, ACTN1, VAV1, PIP4K2A, PPP1R12B, ARPC2, NRAS, PPP1CB, ITGA4, ITGA5, ACTR2, PIK3R1, RAC2, APC, CFL1, ARPC4, GRB2, GIT1, ROCK1, CRK, CYFIP2, WASF2, MAPK3, KRAS, DIAPH1, LIMK2, PPP1R12A, PAK1, ATM, MYL12B, MYL12A, ACTR3, MYL6, NCKAP1L, ARHGEF1, MAPK1, RRAS, RAC1, TMSB10/TMSB4X |
| Breast Cancer Regulation by Stathmin1 | 1.01 × 10^1^ | 0.288 | | | NaN | SOS2, GNA13, GNG11, LIMK1, GNG7, TUBB1, GNB1, PIK3R5, PRKACB, PIK3CB, GNAI3, GNAQ, TUBA1A, ARHGEF6, PIK3CD, PPP2R5A, PRKCH, RB1CC1, CAMK1D, TUBB4B, PRKAR1A, PRKCD, NRAS, ARHGEF2, TUBA1B, PPP1CB, PPP2R5D, TUBA4A, PPP1CC, CALM1 (includes others), PPP1R7, PIK3R1, TP53, GNAI2, E2F2, GRB2, ROCK1, PPP2CA, MAPK3, KRAS, TSG101, LIMK2, GNB2L1, TUBB, PPP1R12A, ADCY7, PAK1, ATM, ITPR3, PRKCA, ARHGEF1, MAPK1, GNG2, RRAS, RAC1 |
| fMLP Signaling in Neutrophils | 9.93 | | 0.352 | | −1.521 | ACTR2, CALM1 (includes others), NFATC2, GNG11, GNG7, NCF1, PIK3R5, GNB1, PIK3R1, FPR1, NFKBIA, PIK3CB, ARPC1B, GNAI3, ARPC5, GNAI2, PIK3CD, ARPC4, PRKCH, MAPK3, KRAS, ARPC3, GNB2L1, ATM, FPR2, PPP3CA, ACTR3, RELA, NFATC3, ARPC2, NRAS, PRKCD, PRKCA, ITPR3, MAPK1, GNG2, RRAS, RAC1 |
| PKCÎ¸ Signaling in T Lymphocytes | 9.84 | | 0.339 | | −1.121 | POU2F1, SOS2, NFATC2, PIK3R5, FCER1G, PIK3R1, RAC2, NFKBIA, PIK3CB, LCP2, PIK3CD, BCL10, HLA-DMA, HLA-DRB5, GRB2, CD4, MAPK8, LAT, ZAP70, MAPK3, CD3E, KRAS, HLA-DQB1, CD3G, ATM, HLA-DOB, PPP3CA, VAV1, RELA, PLCG2, NFATC3, NRAS, MAP3K2, MAP3K1, MAPK1, PLCG1, CD247, RRAS, RAC1, IKBKE |
| Natural Killer Cell Signaling | 9.66 | | 0.345 | | NaN | SOS2, KIR2DS4 (includes others), PIK3R5, FCER1G, PIK3R1, RAC2, PIK3CB, LCP2, INPP5B, INPP5D, PIK3CD, CD300A, GRB2, LAT, PRKCH, KLRC4-KLRK1/KLRK1, ZAP70, MAPK3, KRAS, KIR3DL2, LAIR1, KIR2DL2, PAK1, ATM, KIR2DL1/KIR2DL3, AKT2, VAV1, PLCG2, NRAS, PRKCD, PRKCA, MAPK1, PLCG1, CD247, RRAS, RAC1, FCGR3A/FCGR3B, SH2D1B |
| Role of Tissue Factor in Cancer | 9.66 | | 0.345 | | NaN | HCK, MAPK14, PTEN, F2RL1, GNA13, LIMK1, PIK3R5, FGR, PIK3R1, PIK3CB, TP53, ARRB1, GNAQ, CFL1, PIK3CD, STAT5A, CXCL1, ITGB3, RPS6KB1, MAPK3, KRAS, LIMK2, IL1B, RPS6KA3, PAK1, ATM, ITGA6, PLAUR, AKT2, NRAS, PRKCA, MAPK1, RPS6KA1, BCL2L1, RRAS, RAC1, P4HB, LYN |
| Virus Entry via Endocytic Pathways | 9.39 | | 0.371 | | NaN | FLNB, ITGAL, PIK3R5, PIK3R1, RAC2, PIK3CB, PIK3CD, ACTB, ITGB3, PRKCH, KRAS, FLNA, ATM, CLTC, ITGA6, AP2B1, B2M, PLCG2, AP1G2, NRAS, PRKCD, HLA-A, PRKCA, CD55, AP2A1, ITGA4, PLCG1, HLA-C, HLA-B, AP2M1, RRAS, RAC1, ITGA5 |
| Phospholipase C Signaling | 9.38 | | 0.262 | | −0.160 | SOS2, GNA13, NFATC2, GNG11, GNG7, GNB1, FCER1G, FCGR2C, RALB, LCP2, GNAQ, ARHGEF6, LAT, PRKCH, CD79A, HDAC1, ZAP70, CD3E, MYL4, MYL9, PPP1R12B, PRKCD, NRAS, ARHGEF2, PPP1CB, ITGA4, ITGA5, TGM2, LYN, RHOH, ATF2, CALM1 (includes others), RHOQ, ITK, GRB2, CREBBP, BLNK, MAPK3, KRAS, GNB2L1, CD3G, PPP1R12A, ADCY7, RPS6KA3, MYL12B, EP300, MYL12A, PPP3CA, PLCG2, RELA, NFATC3, MYL6, ATF4, ITPR3, PRKCA, ARHGEF1, MAPK1, PLCG1, GNG2, CD247, RRAS, RAC1 |
| Prostate Cancer Signaling | 9.11 | | 0.378 | | NaN | GSK3B, SOS2, ATF2, PTEN, PIK3R5, PIK3R1, NFKBIA, PIK3CB, TP53, PIK3CD, GRB2, CREBBP, GSTP1, HSP90AA1, MAPK3, KRAS, HSP90B1, ATM, LEF1, EP300, AKT2, RELA, NRAS, PDPK1, ATF4, BCL2, MAPK1, MDM2, HSP90AB1, TFDP1, RRAS |
| mTOR Signaling | 8.89 | | 0.277 | | −1.134 | EIF3G, EIF4B, PIK3R5, RPS5, PIK3CB, MAPKAP1, RPS13, PIK3CD, PPP2R5A, RPS11, PRKCH, RPS6KB1, RPS6, RPS18, EIF3L, EIF4G2, PRKCD, NRAS, PPP2R5D, EIF3K, RPS27A, RPS15, RHOH, EIF3M, RPS10, RPS26, FKBP1A, RHOQ, PIK3R1, ATG13, RPS15A, RPS8, DGKZ, RPS3A, PPP2CA, MAPK3, RPS25, KRAS, ATM, RPS6KA3, RPS2, AKT2, RPS3, PDPK1, PRKCA, RPS17, MAPK1, RPS23, RPS6KA1, RPS28, RRAS, RAC1 |
| CTLA4 Signaling in Cytotoxic T Lymphocytes | 8.88 | 0.364 | | | NaN | PIK3R5, PTPN22, AP1S2, FCER1G, PIK3R1, PIK3CB, LCP2, CD8A, PIK3CD, PPP2R5A, GRB2, LAT, ZAP70, PPP2CA, AP1G1, CD3E, CD3G, ATM, CLTC, AP2B1, B2M, AKT2, AP1G2, HLA-A, TRAT1, PPP2R5D, AP2A1, PLCG1, HLA-C, HLA-B, AP2M1, CD247 |
| protein Ubiquitination Pathway | 8.87 | 0.251 | | | NaN | UBE2J1, HSPA1L, HSPA1A/HSPA1B, USP11, USP7, USP18, HSPA6, ANAPC5, PSMB7, UBE2A, PSMA1, USP48, DNAJB14, UBE2L3, USP22, UBE2H, USP3, DNAJB1, HSP90B1, PSMA6, UBE2G1, DNAJC3, USP15, USP9X, TAP2, PSMB4, BIRC6, UBB, HLA-C, HLA-B, RBX1, PSMB9, USP19, PSMB6, PSMD7, PSMA3, PSME2, CBL, TAP1, PSMB1, USP36, USP8, HSPA8, CDC34, PSMC1, DNAJB6, PSMB3, HSP90AA1, USP4, UBC, USP16, B2M, USP10, UBE2D3, UBE2B, HLA-A, UBE2L6, USP25, HSPA5, PSMD13, MDM2, PSMD8, HSP90AB1, UBE2D1 |
| NF-ÎºB Signaling | 8.80 | 0.283 | | | −0.577 | EIF2AK2, TLR5, TLR1, TNFRSF1A, CSNK2A2, PIK3R5, IL1R2, FCER1G, PRKACB, NFKBIA, PELI1, PIK3CB, TLR4, TLR8, PIK3CD, BCL10, TLR2, HDAC1, ZAP70, TNFSF13B, IL1B, TRAF3, IGF1R, NRAS, BRAF, MAP3K1, AZI2, GSK3B, TDP2, TAB2, PIK3R1, IRAK3, CREBBP, MAPK8, TLR6, KRAS, SIGIRR, CSNK2B, TGFBR3, ATM, EP300, AKT2, IGF2R, PLCG2, RELA, IRAK4, TLR10, RRAS, CASP8 |
| Huntington’s Disease Signaling | 8.69 | 0.258 | | | −0.333 | HSPA1L, HSPA1A/HSPA1B, SOS2, GNG11, POLR2A, GNG7, GNB1, PIK3R5, HSPA6, PIK3CB, PSME4, SDHB, SNCA, GNAQ, PIK3CD, PRKCH, POLR2J2/POLR2J3, ATP5B, HDAC1, DNAJB1, CLTC, CASP1, IGF1R, PRKCD, UBB, BCL2L1, TGM2, ATF2, APAF1, STX16, PIK3R1, PSME2, TP53, VTI1A, HTT, HSPA8, GRB2, CREBBP, MAPK8, CAPNS1, UBC, MAPK3, GNB2L1, ATM, CAPN1, EP300, SDHA, SP1, AKT2, POLR2H, PDPK1, ATF4, PRKCA, HSPA5, MAPK1, NAPG, VTI1B, GNG2, CASP8 |
| PI3K/AKT Signaling | 8.66 | 0.317 | | | −0.845 | GSK3B, SOS2, PTEN, MCL1, JAK3, PIK3R1, NFKBIA, YWHAE, PIK3CB, YWHAH, TP53, INPP5B, INPP5D, PIK3CD, PPP2R5A, GRB2, HSP90AA1, RPS6KB1, PPP2CA, MAPK3, KRAS, HSP90B1, JAK1, AKT2, RELA, NRAS, PDPK1, GAB2, BCL2, MAPK1, PPP2R5D, ITGA4, MDM2, BCL2L1, HSP90AB1, LIMS1, RRAS, ITGA5, IKBKE |
| phagosome maturation | 8.44 | 0.317 | | | NaN | ATP6V1D, VPS11, ATP6V0E2, ATP6V0D1, HLA-DRB3, TUBB1, STX16, PRDX1, TAP1, RAB7A, TUBA1A, ATP6V1F, VTI1A, ATP6V1B2, HLA-DRB5, CANX, LAMP2, PRDX2, M6PR, TSG101, DYNC1H1, TUBB, DYNC1LI1, RAB5A, HLA-DRB4, B2M, TUBB4B, ATP6V0C, LAMP1, HLA-A, PRDX5, TUBA1B, NAPG, VPS28, VTI1B, TUBA4A, HLA-C, HLA-B |
| Hypoxia Signaling in the Cardiovascular System | 8.36 | 0.400 | | | 0.000 | UBE2J1, ATF2, PTEN, NFKBIA, TP53, CSNK1D, CDC34, CREBBP, UBE2A, HSP90AA1, UBE2L3, UBE2H, HSP90B1, ATM, EP300, UBE2D3, UBE2G1, UBE2B, ATF4, UBE2L6, BIRC6, LDHA, MDM2, HSP90AB1, UBE2D1, P4HB |
| RhoGDI Signaling | 8.31 | 0.277 | | | 0.973 | RHOH, GNA13, ACTR2, GNG11, GNG7, LIMK1, GNB1, RHOQ, ARPC1B, GNAI3, ARHGDIB, ARPC5, GNAQ, GNAI2, CFL1, ARHGEF6, ACTB, ARPC4, CREBBP, ROCK1, DGKZ, WASF2, GNAZ, LIMK2, MYL4, ARPC3, GNB2L1, PPP1R12A, PAK1, MYL12B, EP300, EZR, MYL12A, MYL9, ACTR3, PI4KA, PIP4K2A, PPP1R12B, ARPC2, MYL6, PRKCA, ARHGEF1, ARHGEF2, ITGA4, GNG2, RAC1, ITGA5, ARHGAP9 |
| IL-8 Signaling | 8.29 | | | 0.272 | −1.155 | IQGAP1, GNA13, GNG11, LIMK1, GNG7, GNB1, PIK3R5, PIK3CB, GNAI3, PIK3CD, CXCL1, PRKCH, RPS6KB1, CCND2, RAB11FIP2, MYL9, PRKCD, NRAS, BRAF, CXCR2, BCL2L1, IKBKE, RHOH, DEFA1 (includes others), MMP9, RHOQ, PIK3R1, LASP1, RAC2, IRAK3, CCND3, GNAI2, ITGB3, ROCK1, MAPK8, MAPK3, KRAS, LIMK2, GNB2L1, ATM, MYL12B, AKT2, RELA, IRAK4, PRKCA, BCL2, MAPK1, GNG2, RRAS, RAC1 |
| Acute Myeloid Leukemia Signaling | 8.22 | | | 0.367 | NaN | FLT3LG, SOS2, PML, CSF2RA, CSF3R, PIK3R5, PIK3R1, PIK3CB, CSF1R, STAT3, PIK3CD, STAT5A, GRB2, TCF7L2, RPS6KB1, MAPK3, KRAS, ATM, LEF1, PIM2, SPI1, AKT2, RELA, NRAS, BRAF, PIM1, MAPK1, IDH1, RRAS |
| Regulation of IL-2 Expression in Activated and Anergic T Lymphocytes | 8.22 | | | 0.367 | NaN | SOS2, TGFB1, CALM1 (includes others), NFATC2, SMAD4, NFKBIA, BCL10, SMAD3, GRB2, MAPK8, LAT, ZAP70, MAPK3, CD3E, KRAS, CD3G, PPP3CA, VAV1, RELA, PLCG2, NFATC3, NRAS, MAP3K1, MAPK1, PLCG1, CD247, RRAS, RAC1, IKBKE |
| Systemic Lupus Erythematosus Signaling | 8.03 | | | 0.256 | NaN | FCGR1B, SOS2, NFATC2, FCGR1A, PIK3R5, HNRNPC, FCER1G, RNU1-1, LSM12, PIK3CB, FCGR2C, PIK3CD, PRPF38B, IGHM, SNRPA, LAT, CD79A, CD3E, TNFSF13B, IL1B, RNU2-1, RNU4-1, HLA-G, NRAS, HLA-C, HLA-B, LYN, IL6R, PIK3R1, CBL, HLA-F, SNRNP70, SNRNP200, INPP5D, PRPF8, GRB2, MAPK3, KRAS, HLA-E, CD3G, ATM, PIM2, AKT2, PLCG2, NFATC3, HLA-A, MAPK1, PRPF40A, PLCG1, CD247, LSM3, ZCRB1, RRAS, SNRPB, FCGR3A/FCGR3B |
| PDGF Signaling | 7.86 | | | 0.364 | 1.134 | EIF2AK2, SOS2, CSNK2A2, JAK3, PIK3R5, PIK3R1, PIK3CB, STAT3, INPP5B, INPP5D, PIK3CD, STAT1, GRB2, CRK, MAPK8, MAPK3, KRAS, CSNK2B, CRKL, ATM, JAK1, PLCG2, NRAS, PRKCA, MAP3K1, MAPK1, PLCG1, RRAS |
| Prolactin Signaling | 7.78 | | | 0.370 | 1.225 | IRF1, SOS2, PIK3R5, PIK3R1, PIK3CB, STAT3, PIK3CD, STAT1, STAT5A, GRB2, CREBBP, PRKCH, MAPK3, KRAS, TCF7, ATM, EP300, SP1, PLCG2, NRAS, PRKCD, PDPK1, PRKCA, CEBPB, MAPK1, PLCG1, RRAS |
| Thrombin Signaling | 7.71 | | | 0.262 | −0.603 | GNA13, GNG11, GNG7, GNB1, PIK3R5, PIK3CB, GNAI3, GATA3, GNAQ, ARHGEF6, PIK3CD, PRKCH, RPS6KB1, GNAZ, MYL4, CAMK1D, MYL9, PPP1R12B, PRKCD, NRAS, ARHGEF2, PPP1CB, RHOH, MAPK14, RHOQ, PIK3R1, GNAI2, GRB2, ROCK1, MAPK3, KRAS, GNB2L1, PPP1R12A, ADCY7, ATM, MYL12B, MYL12A, AKT2, GATA1, PLCG2, RELA, MYL6, PDPK1, ITPR3, PRKCA, ARHGEF1, MAPK1, PLCG1, GNG2, RRAS |
| PI3K Signaling in B Lymphocytes | 7.58 | | | 0.297 | −0.667 | ATF2, PTEN, CALM1 (includes others), NFATC2, PLEKHA2, PIK3R1, NFKBIA, CBL, PIK3CB, TLR4, INPP5D, PIK3CD, BCL10, PIK3AP1, CD180, BLNK, CD79A, CD81, MAPK3, KRAS, PPP3CA, ATF6, AKT2, VAV1, RELA, PLCG2, NFATC3, NRAS, PDPK1, ATF4, ITPR3, MAPK1, PLCG1, RRAS, RAC1, ATF6B, IKBKE, LYN |
| PTEN Signaling | 7.55 | | 0.305 | | 0.174 | GSK3B, SOS2, PTEN, BCL2L11, CSNK2A2, PIK3R5, FOXO4, PIK3R1, RAC2, CBL, PIK3CB, YWHAH, INPP5B, INPP5D, PIK3CD, GRB2, RPS6KB1, MAPK3, KRAS, CSNK2B, TGFBR3, AKT2, IGF2R, RELA, IGF1R, NRAS, PDPK1, SHARPIN, BCL2, MAPK1, ITGA4, BCL2L1, RRAS, RAC1, ITGA5, IKBKE |
| Signaling by Rho Family GTPases | 7.48 | | 0.244 | | −1.342 | IQGAP1, GNA13, GNG11, SEPT14, LIMK1, GNG7, GNB1, PIK3R5, PIK3CB, ARPC1B, GNAI3, ARPC5, GNAQ, ARHGEF6, PIK3CD, ACTB, GNAZ, MYL4, ARPC3, EZR, MYL9, PIP4K2A, PI4KA, PPP1R12B, ARPC2, SEPT7, ARHGEF2, ITGA4, ITGA5, RHOH, ACTR2, RHOQ, WIPF1, PIK3R1, VIM, GNAI2, CFL1, CLIP1, ARPC4, ROCK1, MAPK8, MAPK3, LIMK2, GNB2L1, PPP1R12A, PAK1, ATM, MYL12B, MYL12A, ACTR3, SEPT2, RELA, MYL6, ARHGEF1, MAPK1, GNG2, RAC1 |
| IL-3 Signaling | 7.42 | | 0.366 | | NaN | PIK3R5, PIK3R1, PIK3CB, STAT3, INPP5D, PIK3CD, STAT1, STAT5A, GRB2, PRKCH, MAPK3, KRAS, CRKL, PAK1, ATM, JAK1, PPP3CA, AKT2, NRAS, PRKCD, GAB2, PRKCA, STAT6, MAPK1, RRAS, RAC1 |
| Tec Kinase Signaling | 7.34 | | 0.274 | | 1.414 | RHOH, HCK, GNA13, GNG11, GNG7, JAK3, PIK3R5, GNB1, FGR, RHOQ, FCER1G, PIK3R1, PIK3CB, GNAI3, TLR4, GNAQ, STAT3, GNAI2, PIK3CD, STAT1, ACTB, STAT5A, ITK, MAPK8, PRKCH, GNAZ, GNB2L1, PAK1, ATM, STAT2, JAK1, VAV1, RELA, PLCG2, PRKCD, PRKCA, STAT6, TNFSF10, ITGA4, PLCG1, GNG2, ITGA5, LYN |
| CXCR4 Signaling | 7.30 | | 0.276 | | −2.596 | RHOH, GNA13, GNG11, GNG7, PIK3R5, GNB1, RHOQ, PIK3R1, PIK3CB, GNAI3, CXCR4, GNAQ, GNAI2, PIK3CD, CD4, ROCK1, CRK, MAPK8, PRKCH, ELMO1, MAPK3, KRAS, GNAZ, MYL4, GNB2L1, ADCY7, PAK1, MYL12B, ATM, MYL12A, MYL9, AKT2, MYL6, NRAS, PRKCD, PRKCA, ITPR3, MAPK1, GNG2, RRAS, RAC1, LYN |
| Remodeling of Epithelial Adherens Junctions | 7.20 | | 0.368 | | −2.887 | IQGAP1, ACTR2, TUBB1, MAPRE1, ARPC1B, APC, ZYX, ARPC5, RAB7A, TUBA1A, CLIP1, ACTB, ARPC4, ACTN4, CTNNA1, ARPC3, TUBB, RAB5A, ACTN1, ACTR3, TUBB4B, ARPC2, TUBA1B, TUBA4A, ARF6 |
| ERK/MAPK Signaling | 7.15 | | 0.257 | | 1.896 | PPP1CC, SOS2, ATF2, PPP1R7, PIK3R5, MKNK2, PIK3R1, PRKACB, RAC2, PIK3CB, YWHAH, STAT3, PIK3CD, PPP2R5A, DUSP1, STAT1, GRB2, CREBBP, CRK, PPP2CA, MAPK3, KSR1, KRAS, TLN1, PPP1R12A, CRKL, PAK1, ATM, EP300, H3F3A/H3F3B, PLCG2, PRKAR1A, NRAS, PRKCD, BRAF, ATF4, PRKCA, ELF4, MAPK1, PPP1CB, PPP2R5D, RPS6KA1, ITGA4, PLCG1, ETS1, RRAS, RAC1, ITGA5 |
| FAK Signaling | 7.14 | | 0.333 | | NaN | SOS2, PTEN, PIK3R5, PIK3R1, PIK3CB, ARHGEF6, PIK3CD, ACTB, GRB2, CRK, ARHGAP26, CAPNS1, MAPK3, KRAS, TLN1, PAK1, ATM, CAPN1, GIT2, AKT2, PLCG2, NRAS, PDPK1, MAPK1, ITGA4, PLCG1, RRAS, RAC1, ITGA5 |
| NGF Signaling | 7.11 | | 0.308 | | −0.354 | SOS2, ATF2, PIK3R5, PIK3R1, PIK3CB, TP53, PIK3CD, GRB2, CREBBP, ROCK1, CRK, MAPK8, RPS6KB1, MAPK3, KRAS, RPS6KA3, ATM, EP300, AKT2, RELA, PLCG2, NRAS, PRKCD, PDPK1, ATF4, MAP3K2, MAP3K1, MAPK1, RPS6KA1, PLCG1, RRAS, RAC1, IKBKE |
| Actin Nucleation by ARP-WASP Complex | 7.01 | | 0.393 | | −2.524 | KRAS, RHOH, SOS2, ACTR2, ARPC3, PPP1R12A, WIPF1, RHOQ, ARPC1B, ACTR3, PPP1R12B, ARPC2, ARPC5, NRAS, NCK2, ARPC4, GRB2, ROCK1, ITGA4, RRAS, RAC1, ITGA5 |
| EGF Signaling | 7.01 | | 0.393 | | 0.426 | SOS2, MAPK14, CSNK2B, ATM, CSNK2A2, PIK3R5, JAK1, PIK3R1, PIK3CB, AKT2, STAT3, PIK3CD, ITPR3, PRKCA, STAT1, MAP3K1, MAPK1, GRB2, PLCG1, MAPK8, RPS6KB1, MAPK3 |
| Colorectal Cancer Metastasis Signaling | 6.95 | | 0.237 | | −1.54 | SOS2, TLR5, TGFB1, TLR1, GNG11, TNFRSF1A, GNG7, JAK3, GNB1, PIK3R5, SMAD4, PRKACB, PIK3CB, TLR4, TLR8, IFNGR1, STAT3, PIK3CD, SMAD3, LRP1, TLR2, LEF1, MLH1, JAK1, MSH3, PRKAR1A, NRAS, BRAF, MMP8, BCL2L1, GSK3B, RHOH, MMP9, IL6R, RHOQ, PIK3R1, TP53, APC, ARRB1, STAT1, GRB2, MAPK8, TLR6, TCF7L2, MAPK3, KRAS, GNB2L1, ADCY7, ATM, AKT2, RELA, MAPK1, GNG2, TLR10, RRAS, RAC1 |
| Molecular Mechanisms of Cancer | 6.93 | | 0.211 | | NaN | SOS2, GNA13, TGFB1, JAK3, PIK3R5, SMAD4, PRKACB, NFKBIA, PIK3CB, RASGRP1, GNAI3, RALB, CFLAR, GNAQ, ARHGEF6, PIK3CD, SMAD3, LRP1, PRKCH, NF1, GNAZ, CCND2, LEF1, JAK1, PRKAR1A, PRKCD, NRAS, BRAF, PSENEN, ARHGEF2, RASGRF2, ITGA4, BCL2L1, ITGA5, GSK3B, RHOH, MAPK14, APAF1, BCL2L11, TAB2, RHOQ, PIK3R1, RAC2, CBL, TP53, APC, CCND3, GNAI2, E2F2, GRB2, CREBBP, RAP2B, CRK, MAPK8, MAPK3, CTNNA1, KRAS, NOTCH1, ADCY7, PAK1, ATM, EP300, AKT2, RELA, NAIP, GAB2, PRKCA, ARHGEF1, BCL2, MAPK1, MDM2, HIPK2, TFDP1, RRAS, RAC1, CASP8, CDC25B |
| p70S6K Signaling | 6.92 | | 0.294 | | −1.219 | SOS2, F2RL1, PIK3R5, PIK3R1, YWHAE, PIK3CB, YWHAH, EEF2, IL2RG, GNAI3, GNAQ, GNAI2, PIK3CD, PPP2R5A, GRB2, PRKCH, CD79A, RPS6KB1, PPP2CA, MAPK3, KRAS, RPS6, ATM, JAK1, AKT2, PLCG2, NRAS, PRKCD, PDPK1, PRKCA, MAPK1, PPP2R5D, PLCG1, RRAS, LYN |
| Rac Signaling | 6.89 | | 0.308 | | −0.354 | IQGAP1, ACTR2, LIMK1, PIK3R5, PIK3R1, PIK3CB, ARPC1B, ARPC5, CFL1, PIK3CD, ARPC4, MAPK8, CYFIP2, RPS6KB1, MAPK3, KRAS, LIMK2, ARPC3, PAK1, ATM, ACTR3, PI4KA, PIP4K2A, RELA, ARPC2, NRAS, MAP3K1, MAPK1, ITGA4, RRAS, RAC1, ITGA5 |
| Telomerase Signaling | 6.88 | | 0.313 | | −0.392 | SOS2, PIK3R5, IL2RB, PIK3R1, PIK3CB, TP53, IL2RG, PIK3CD, TERF1, PPP2R5A, GRB2, HSP90AA1, HDAC1, TPP1, PPP2CA, MAPK3, KRAS, TEP1, HSP90B1, ATM, SP1, AKT2, NRAS, PDPK1, ELF4, MAPK1, PPP2R5D, HSP90AB1, ETS1, RRAS, TERF2IP |
| IL-4 Signaling | 6.86 | | | 0.347 | NaN | SOS2, NFATC2, JAK3, PIK3R5, PIK3R1, PIK3CB, IL2RG, INPP5B, INPP5D, PIK3CD, HLA-DMA, HLA-DRB5, IRF4, GRB2, RPS6KB1, KRAS, HLA-DQB1, ATM, JAK1, HLA-DOB, AKT2, NFATC3, FCER2, NRAS, STAT6, RRAS |
| Ephrin Receptor Signaling | 6.84 | | | 0.259 | NaN | SOS2, ATF2, GNA13, ACTR2, GNG11, GNG7, LIMK1, GNB1, WIPF1, RAC2, ARPC1B, GNAI3, ARPC5, CXCR4, GNAQ, STAT3, GNAI2, CFL1, ARPC4, GRB2, CREBBP, ROCK1, CRK, SDCBP, MAPK3, KRAS, GNAZ, LIMK2, ARPC3, GNB2L1, CRKL, PAK1, EP300, ACTR3, AKT2, ARPC2, NRAS, ATF4, NCK2, MAPK1, ITGA4, GNG2, RRAS, RAC1, ITGA5 |
| Production of Nitric Oxide and Reactive Oxygen Species in Macrophages | 6.82 | | | 0.256 | 0.447 | IRF1, RHOH, PPP1CC, MAPK14, TNFRSF1A, PPP1R7, NCF1, JAK3, PIK3R5, RHOQ, PIK3R1, NFKBIA, PIK3CB, TLR4, IFNGR1, PIK3CD, PPP2R5A, STAT1, CREBBP, MAPK8, PRKCH, SIRPA, TLR2, PPP2CA, MAPK3, NCF4, PPP1R12A, ATM, JAK1, SPI1, AKT2, RELA, PLCG2, LYZ, PRKCD, PRKCA, MAP3K2, MAP3K1, MAPK1, PPP1CB, SERPINA1, PPP2R5D, PLCG1, CYBA, RAC1, IKBKE |
| IL-2 Signaling | 6.79 | | | 0.396 | −0.471 | KRAS, SOS2, CSNK2B, ATM, CSNK2A2, JAK3, PIK3R5, IL2RB, JAK1, PIK3R1, PIK3CB, AKT2, IL2RG, NRAS, PIK3CD, STAT5A, MAPK1, GRB2, MAPK8, RRAS, MAPK3 |
| PEDF Signaling | 6.77 | | | 0.352 | 0.408 | MAPK14, PIK3R5, PIK3R1, NFKBIA, PIK3CB, TP53, CFLAR, PIK3CD, PNPLA2, ROCK1, WASF2, MAPK3, KRAS, TCF7, ATM, AKT2, RELA, NRAS, BCL2, MAPK1, BCL2L1, RRAS, RAC1, CASP8, IKBKE |
| GM-CSF Signaling | 6.75 | | | 0.371 | −1.528 | KRAS, SOS2, HCK, GNB2L1, CSF2RA, ATM, PIK3R5, PIK3R1, PPP3CA, PIK3CB, AKT2, NRAS, STAT3, PIK3CD, STAT1, PIM1, MAPK1, GRB2, BCL2L1, ETS1, RRAS, MAPK3, LYN |
| Androgen Signaling | 6.69 | | | 0.297 | 0 | KAT2B, NCOA4, GNA13, CALM1 (includes others), POLR2A, GNG11, GNG7, GNB1, PRKACB, GNAI3, GNAQ, GNAI2, SMAD3, CREBBP, PRKCH, POLR2J2/POLR2J3, HSP90AA1, MAPK3, GNAZ, DNAJB1, NCOA1, GNB2L1, TAF2, EP300, GTF2A1, RELA, PRKAR1A, POLR2H, PRKCD, PRKCA, MAPK1, GNG2, NCOA2 |
| Erythropoietin Signaling | 6.69 | | | 0.358 | NaN | KRAS, SOS2, ATM, PIK3R5, PIK3R1, NFKBIA, CBL, PIK3CB, AKT2, RELA, PLCG2, NRAS, PRKCD, PDPK1, PIK3CD, PRKCA, STAT5A, MAPK1, GRB2, PLCG1, PRKCH, RRAS, RPS6KB1, MAPK3 |
| Regulation of Actin-based Motility by Rho | 6.67 | | 0.319 | | −1.512 | RHOH, ACTR2, LIMK1, WIPF1, RHOQ, RAC2, ARPC1B, ARPC5, CFL1, ACTB, ARPC4, ROCK1, MYL4, ARPC3, PPP1R12A, PAK1, MYL12B, MYL12A, MYL9, ACTR3, PI4KA, PIP4K2A, PPP1R12B, ARPC2, MYL6, PPP1CB, ITGA4, RAC1, ITGA5 |
| NF-ÎºB Activation by Viruses | 6.50 | | 0.342 | | NaN | EIF2AK2, ITGAL, PIK3R5, PIK3R1, NFKBIA, PIK3CB, PIK3CD, CD4, ITGB3, PRKCH, MAPK3, KRAS, ATM, ITGA6, AKT2, RELA, NRAS, PRKCD, PRKCA, MAP3K1, MAPK1, ITGA4, RRAS, ITGA5, IKBKE |
| G Beta Gamma Signaling | 6.44 | | 0.318 | | −0.962 | SOS2, GNA13, GNG11, GNG7, GNB1, PRKACB, GNAI3, GNAQ, GNAI2, ARHGEF6, GRB2, PRKCH, MAPK3, KRAS, GNAZ, GNB2L1, PAK1, AKT2, PLCG2, PRKAR1A, NRAS, PRKCD, PDPK1, PRKCA, MAPK1, PLCG1, GNG2, RRAS |
| UVA-Induced MAPK Signaling | 6.44 | | 0.318 | | 0.577 | MAPK14, PIK3R5, PARP9, PIK3R1, PARP8, PIK3CB, TP53, TIPARP, PIK3CD, STAT1, PARP14, MAPK8, RPS6KB1, MAPK3, PARP12, KRAS, PARP4, RPS6KA3, ATM, PLCG2, NRAS, PRKCA, ZC3HAV1, MAPK1, RPS6KA1, PLCG1, BCL2L1, RRAS |
| Clathrin-mediated Endocytosis Signaling | 6.44 | | 0.249 | | NaN | GAK, RAB11A, ACTR2, CSNK2A2, PIK3R5, PIK3R1, CBL, PIK3CB, ARPC1B, RAB4B, ARRB1, ARPC5, RAB7A, PIK3CD, ACTB, HSPA8, ARPC4, GRB2, ITGB3, RAB11B, UBC, SH3KBP1, TSG101, ARPC3, CSNK2B, ATM, CLTC, RAB5A, AP2B1, AAK1, PPP3CA, ACTR3, AP1G2, ARPC2, NUMB, LYZ, USP9X, UBB, SERPINA1, AP2A1, MDM2, DAB2, AP2M1, ARF6, RAC1, ITGA5 |
| NRF2-mediated Oxidative Stress Response | 6.39 | | 0.250 | | −3.162 | GSK3B, MAPK14, FTL, NQO2, PIK3R5, PIK3R1, PRDX1, PIK3CB, JUND, FKBP5, PIK3CD, ACTB, GSR, CDC34, CREBBP, MAPK8, PRKCH, DNAJB6, GSTP1, CUL3, DNAJB14, MGST3, MAPK3, KRAS, DNAJB1, TXN, ATM, EP300, PPIB, DNAJC3, NRAS, PRKCD, ATF4, DNAJA2, PRKCA, MAP3K1, UBB, MAPK1, UBE2K, FTH1, RRAS, RBX1, NFE2L2, HERPUD1, JUNB |
| Renin-Angiotensin Signaling | 6.37 | | 0.294 | | 0.186 | SOS2, ATF2, MAPK14, PIK3R5, PIK3R1, PRKACB, PIK3CB, GNAQ, STAT3, PIK3CD, STAT1, GRB2, MAPK8, PRKCH, MAPK3, KRAS, CCL5, ADCY7, PAK1, ATM, RELA, PLCG2, PRKAR1A, NRAS, PRKCD, PRKCA, ITPR3, MAP3K1, MAPK1, PLCG1, RRAS, RAC1 |
| Leukocyte Extravasation Signaling | 6.34 | | 0.242 | | −0.302 | RHOH, ITGAL, F11R, MAPK14, MMP9, NCF1, PIK3R5, WIPF1, PIK3R1, RAC2, PIK3CB, RASGRP1, GNAI3, CXCR4, GNAI2, PIK3CD, ACTB, ICAM3, ITK, ITGB3, ROCK1, CRK, MAPK8, PRKCH, ACTN4, CTNNA1, NCF4, CRKL, ATM, EZR, ITGA6, ACTN1, VAV1, PLCG2, MYL6, PRKCD, PRKCA, SELPLG, MAPK1, MMP8, ITGA4, PLCG1, TIMP1, CYBA, RAC1, ITGA5, PECAM1, ARHGAP9 |
| SAPK/JNK Signaling | 6.33 | | 0.309 | | −1.671 | SOS2, ATF2, GNA13, GNG11, MAP4K5, GNG7, PIK3R5, GNB1, FCER1G, PIK3R1, RAC2, PIK3CB, TP53, PIK3CD, GRB2, CRK, MAPK8, KRAS, HNRNPK, CRKL, ATM, NFATC3, NRAS, MAP3K2, MAP3K1, GNG2, RRAS, RAC1, MINK1 |
| PAK Signaling | 6.33 | | 0.315 | | −1.347 | SOS2, LIMK1, PIK3R5, PIK3R1, PIK3CB, CFL1, ARHGEF6, PIK3CD, GRB2, GIT1, MAPK8, MAPK3, KRAS, LIMK2, MYL4, PAK1, MYL12B, ATM, MYL12A, MYL9, MYL6, NRAS, NCK2, MAPK1, ITGA4, RRAS, RAC1, ITGA5 |
| Apoptosis Signaling | 6.33 | | 0.315 | | −1.512 | MCL1, APAF1, BCL2L11, TNFRSF1A, NFKBIA, TP53, ROCK1, MAPK8, CAPNS1, MAPK3, KRAS, CAPN1, ACIN1, RELA, PLCG2, NAIP, NRAS, PRKCA, BCL2, BIRC6, MAPK1, RPS6KA1, PLCG1, BCL2L1, RRAS, CASP8, IKBKE, SPTAN1 |
| Endometrial Cancer Signaling | 6.25 | | 0.385 | | NaN | CTNNA1, GSK3B, KRAS, SOS2, PTEN, LEF1, ATM, PIK3R5, MLH1, PIK3R1, PIK3CB, TP53, AKT2, NRAS, PDPK1, PIK3CD, MAPK1, GRB2, RRAS, MAPK3 |
| Dendritic Cell Maturation | 6.19 | | 0.249 | | 0.316 | FCGR1B, ATF2, MAPK14, FCGR1A, TNFRSF1A, HLA-DRB3, PIK3R5, FCER1G, PIK3R1, NFKBIA, PIK3CB, FCGR2C, IFNAR1, TLR4, PIK3CD, HLA-DMA, STAT1, HLA-DRB5, CREBBP, MAPK8, TLR2, MAPK3, IL32, IL1B, HLA-DQB1, CD58, ATM, STAT2, EP300, HLA-DRB4, HLA-DOB, B2M, LY75, AKT2, RELA, PLCG2, HLA-A, ATF4, MAPK1, PLCG1, HLA-C, HLA-B, FCGR3A/FCGR3B, IKBKE |
| IL-15 Signaling | 6.19 | | 0.348 | | NaN | KRAS, MAPK14, ATM, JAK3, PIK3R5, IL2RB, JAK1, PIK3R1, PIK3CB, AKT2, IL2RG, RELA, NRAS, STAT3, PIK3CD, STAT5A, BCL2, STAT6, MAPK1, PLCG1, BCL2L1, RRAS, MAPK3 |
| Paxillin Signaling | 6.13 | | 0.297 | | −0.962 | SOS2, ITGAL, MAPK14, PIK3R5, PIK3R1, PIK3CB, ARHGEF6, PIK3CD, ACTB, GRB2, ITGB3, CRK, MAPK8, ACTN4, KRAS, TLN1, PAK1, ATM, ITGA6, GIT2, ACTN1, ARF1, NRAS, NCK2, MAPK1, ITGA4, ARF6, RRAS, RAC1, ITGA5 |
| JAK/Stat Signaling | 6.03 | | 0.333 | | 0.408 | KRAS, SOS2, STAT2, ATM, JAK3, PIK3R5, JAK1, PIK3R1, PIK3CB, AKT2, RELA, NRAS, GNAQ, STAT3, PIK3CD, STAT1, STAT5A, CEBPB, STAT6, MAPK1, GRB2, BCL2L1, RRAS, MAPK3 |
| ILK Signaling | 5.96 | | 0.242 | | 0.469 | FLNB, GSK3B, RHOH, ATF2, PTEN, MMP9, TNFRSF1A, PIK3R5, RHOQ, PIK3R1, TESK1, PIK3CB, MYH9, VIM, CFL1, ARHGEF6, PIK3CD, PPP2R5A, ACTB, ITGB3, CREBBP, MAPK8, ACTN4, PPP2CA, MAPK3, MYL4, PPP1R12A, FLNA, ATM, LEF1, EP300, ACTN1, MYL9, AKT2, RELA, KRT18, MYL6, PDPK1, ATF4, NCK2, MAPK1, PPP2R5D, LIMS1, TMSB10/TMSB4X, NACA |
| Epithelial Adherens Junction Signaling | 5.96 | | 0.260 | | NaN | IQGAP1, PTEN, ACTR2, TUBB1, ARPC1B, NOTCH2, APC, MYH9, ZYX, ARPC5, TUBA1A, CLIP1, ACTB, ARPC4, CRK, TCF7L2, ACTN4, CTNNA1, KRAS, MYL4, ARPC3, NOTCH1, TUBB, TGFBR3, LEF1, ACTN1, MYL9, ACTR3, AKT2, TUBB4B, ARPC2, MYL6, NRAS, TUBA1B, CLINT1, TUBA4A, RRAS, RAC1 |
| Unfolded protein response | 5.94 | | 0.370 | | NaN | HSPA1L, HSPA1A/HSPA1B, CEBPD, HSP90B1, HSPA6, ATF6, DNAJC3, ATF4, DNAJA2, HSPA8, UBXN4, CEBPB, HSPA5, BCL2, XBP1, CANX, SCAP, MAPK8, P4HB, NFE2L2 |
| Macropinocytosis Signaling | 5.93 | | 0.338 | | 0.688 | KRAS, CD14, PAK1, ATM, RAB5A, PIK3R5, PIK3R1, PIK3CB, ANKFY1, CSF1R, PLCG2, NRAS, PRKCD, PIK3CD, PRKCA, PLCG1, ITGB3, PRKCH, ACTN4, ARF6, RRAS, RAC1, ITGA5 |
| P2Y Purigenic Receptor Signaling Pathway | 5.92 | | 0.277 | | 0.707 | ATF2, GNG11, GNG7, PIK3R5, GNB1, PIK3R1, PRKACB, PIK3CB, GNAI3, GNAQ, GNAI2, PIK3CD, ITGB3, CREBBP, PRKCH, MAPK3, KRAS, GNB2L1, ADCY7, ATM, EP300, AKT2, RELA, PLCG2, PRKAR1A, NRAS, PRKCD, ATF4, PRKCA, MAPK1, PLCG1, GNG2, RRAS |
| Ephrin B Signaling | 5.90 | | 0.329 | | −1.732 | GNAZ, GNA13, GNB2L1, HNRNPK, GNG11, PAK1, GNG7, LIMK1, GNB1, RAC2, CBL, VAV1, GNAI3, CXCR4, GNAQ, GNAI2, CFL1, NCK2, ITSN2, MAPK1, ROCK1, GNG2, RAC1, MAPK3 |
| Chronic Myeloid Leukemia Signaling | 5.90 | | 0.301 | | NaN | SOS2, TGFB1, PIK3R5, PIK3R1, SMAD4, PIK3CB, TP53, PIK3CD, SMAD3, E2F2, STAT5A, GRB2, CRK, HDAC1, MAPK3, KRAS, CRKL, ATM, AKT2, RELA, NRAS, GAB2, MAPK1, MDM2, BCL2L1, TFDP1, RRAS, IKBKE |
| Neuregulin Signaling | 5.89 | | 0.307 | | −0.229 | SOS2, PTEN, PIK3R1, ADAM17, STAT5A, GRB2, CRK, PRKCH, HSP90AA1, RPS6KB1, MAPK3, KRAS, RPS6, CRKL, HSP90B1, AKT2, PLCG2, NRAS, PRKCD, PDPK1, PRKCA, MAPK1, ITGA4, PLCG1, HSP90AB1, RRAS, ITGA5 |
| Calcium-induced T Lymphocyte Apoptosis | 5.83 | | 0.344 | | 1.807 | CD3E, ATP2A3, CALM1 (includes others), NFATC2, HLA-DQB1, CD3G, EP300, HLA-DOB, FCER1G, PPP3CA, PRKCD, CABIN1, ITPR3, PRKCA, HLA-DMA, HLA-DRB5, CD4, PLCG1, PRKCH, CD247, HDAC1, ZAP70 |
| Thrombopoietin Signaling | 5.80 | | 0.364 | | 0.894 | KRAS, ATM, PIK3R5, PIK3R1, PIK3CB, PLCG2, NRAS, STAT3, PRKCD, PIK3CD, GAB2, PRKCA, STAT1, STAT5A, MAPK1, GRB2, PLCG1, PRKCH, RRAS, MAPK3 |
| Mitochondrial Dysfunction | 5.79 | | 0.246 | | NaN | NDUFB9, VDAC3, MT-CYB, MT-ND5, COX5A, CPT1A, MT-CO3, MT-ND2, MT-ND1, SDHB, SNCA, COX6B1, GSR, TXN2, GPX7, SDHD, NDUFS5, MAPK8, UQCRFS1, ATP5C1, NDUFV2, ATP5B, UCP2, PINK1, COX4I1, PARK7, SDHA, APP, COX5B, NDUFA2, ATP5D, ATP5G1, PRDX5, PSENEN, VDAC2, BCL2, ACO1, MT-CO2, LRRK2, NDUFB5, ATPAF2, CASP8 |
| Melanocyte Development and Pigmentation Signaling | 5.77 | | 0.310 | | −0.392 | SOS2, ATF2, PIK3R5, PIK3R1, PRKACB, PIK3CB, PIK3CD, GRB2, CREBBP, CRK, RPS6KB1, MAPK3, KRAS, ADCY7, RPS6KA3, ATM, EP300, PLCG2, PRKAR1A, NRAS, ATF4, BCL2, MAPK1, RPS6KA1, PLCG1, RRAS |
| Germ Cell-Sertoli Cell Junction Signaling | 5.76 | | 0.250 | | NaN | IQGAP1, RHOH, MAPK14, TGFB1, TNFRSF1A, LIMK1, TUBB1, PIK3R5, RHOQ, PIK3R1, RAC2, PIK3CB, ZYX, TUBA1A, CFL1, PIK3CD, ACTB, MAPK8, ACTN4, MAPK3, CTNNA1, KRAS, LIMK2, TUBB, PAK1, ATM, ITGA6, ACTN1, RAB8B, TUBB4B, NRAS, PDPK1, MAP3K2, MAP3K1, TUBA1B, MAPK1, CLINT1, TUBA4A, RRAS, RAC1 |
| HGF Signaling | 5.74 | | 0.286 | | 0.378 | SOS2, ATF2, PIK3R5, PIK3R1, PIK3CB, STAT3, PIK3CD, GRB2, MAPK8, PRKCH, MAPK3, KRAS, CRKL, PAK1, ATM, AKT2, PLCG2, NRAS, PRKCD, PRKCA, MAP3K2, MAP3K1, ELF4, MAPK1, ITGA4, PLCG1, ETS1, RRAS, RAC1, ITGA5 |
| Non-Small Cell Lung Cancer Signaling | 5.70 | | 0.338 | | −0.447 | KRAS, SOS2, FHIT, ATM, PIK3R5, PIK3R1, PIK3CB, TP53, AKT2, NRAS, PDPK1, PIK3CD, ITPR3, PRKCA, MAPK1, GRB2, RXRA, PLCG1, TFDP1, RRAS, STK4, MAPK3 |
| TREM1 Signaling | 5.67 | | 0.320 | | NaN | SIGIRR, TLR5, NLRP12, IL1B, TLR1, NLRC5, CASP1, AKT2, RELA, PLCG2, TLR4, TLR8, STAT3, STAT5A, MAPK1, GRB2, TREM1, PLCG1, TLR6, TLR10, ITGA5, TLR2, NLRC4, MAPK3 |
| Role of NFAT in Cardiac Hypertrophy | 5.64 | 0.240 | | | 0 | GSK3B, CSNK1A1, SOS2, MAPK14, TGFB1, CALM1 (includes others), GNG11, GNG7, PIK3R5, GNB1, PIK3R1, PRKACB, PIK3CB, GNAI3, GNAQ, GNAI2, PIK3CD, GRB2, MAPK8, PRKCH, HDAC1, MAPK3, KRAS, GNB2L1, ADCY7, ATM, EP300, CAMK1D, PPP3CA, AKT2, PLCG2, PRKAR1A, IGF1R, NRAS, PRKCD, CABIN1, PRKCA, ITPR3, MAP3K1, MAPK1, PLCG1, GNG2, RRAS |
| 14-3-3-mediated Signaling | 5.62 | 0.274 | | | 0 | GSK3B, TNFRSF1A, TUBB1, PIK3R5, PIK3R1, YWHAE, CBL, PIK3CB, YWHAH, VIM, TUBA1A, SNCA, PIK3CD, GRB2, MAPK8, PRKCH, MAPK3, KRAS, TUBB, ATM, AKT2, TUBB4B, PLCG2, NRAS, PRKCD, PRKCA, TUBA1B, MAPK1, RPS6KA1, TUBA4A, PLCG1, RRAS |
| CCR3 Signaling in Eosinophils | 5.62 | 0.274 | | | NaN | MAPK14, CALM1 (includes others), GNG11, GNG7, LIMK1, PIK3R5, GNB1, PIK3R1, PIK3CB, GNAI3, GNAI2, CFL1, PIK3CD, ROCK1, PRKCH, MAPK3, KRAS, LIMK2, GNB2L1, PPP1R12A, PAK1, ATM, PPP1R12B, NRAS, PRKCD, PRKCA, ITPR3, MAPK1, PPP1CB, GNG2, RRAS, RAC1 |
| Role of Macrophages, Fibroblasts and Endothelial Cells in Rheumatoid Arthritis | 5.60 | 0.209 | | | NaN | TLR5, TGFB1, NFATC2, FCGR1A, TLR1, TNFRSF1A, PIK3R5, IL1R2, NFKBIA, PIK3CB, TLR4, TLR8, STAT3, GNAQ, PIK3CD, LRP1, PRKCH, TLR2, TNFSF13B, IL32, CCL5, IL1B, LEF1, TRAF3, PRKCD, IL17RA, NRAS, IKBKE, GSK3B, CSNK1A1, ATF2, MAPK14, F2RL1, CALM1 (includes others), IL6R, PIK3R1, APC, IRAK3, CREBBP, ROCK1, TLR6, TCF7L2, MAPK3, KRAS, CEBPD, ATM, EP300, PPP3CA, AKT2, PLCG2, RELA, IRAK4, NFATC3, ATF4, PRKCA, CEBPB, MAPK1, PLCG1, TLR10, RRAS, RAC1, FCGR3A/FCGR3B |
| Caveolar-mediated Endocytosis Signaling | 5.56 | 0.324 | | | NaN | FLNB, FLOT1, ITGAL, FLNA, CD48, ITGA6, RAB5A, B2M, HLA-A, PRKCA, ACTB, COPA, MAP3K2, COPE, COPB1, CD55, ITGB3, HLA-C, ITGA4, COPZ1, HLA-B, ITGA5, ARCN1 |
| Renal Cell Carcinoma Signaling | 5.56 | 0.324 | | | −1.213 | KRAS, SOS2, TGFB1, PAK1, ATM, EP300, PIK3R5, PIK3R1, PIK3CB, AKT2, NRAS, PIK3CD, UBB, MAPK1, GRB2, CREBBP, CRK, ETS1, RRAS, RAC1, RBX1, UBC, MAPK3 |
| Chemokine Signaling | 5.56 | 0.324 | | | 0.209 | KRAS, MAPK14, LIMK2, CCL5, CALM1 (includes others), PPP1R12A, LIMK1, CAMK1D, GNAI3, PPP1R12B, PLCG2, NRAS, CXCR4, GNAQ, GNAI2, CFL1, PRKCA, MAPK1, PPP1CB, PLCG1, MAPK8, RRAS, MAPK3 |
| Cardiac Hypertrophy Signaling | 5.51 | 0.224 | | | −1.18 | GNA13, TGFB1, GNG11, GNG7, GNB1, PIK3R5, PRKACB, PIK3CB, GNAI3, GNAQ, PIK3CD, RPS6KB1, GNAZ, MYL4, MYL9, ATF6, PRKAR1A, IGF1R, NRAS, MAP3K1, GSK3B, RHOH, ATF2, MAPK14, CALM1 (includes others), IL6R, RHOQ, PIK3R1, GNAI2, GRB2, CREBBP, ROCK1, MAPK8, MAPK3, KRAS, GNB2L1, ADCY7, ATM, MYL12B, EP300, MYL12A, PPP3CA, PLCG2, MYL6, MAP3K2, MAPK1, RPS6KA1, PLCG1, GNG2, RRAS |
| Fc Epsilon RI Signaling | 5.46 | 0.278 | | | −1.826 | SOS2, MAPK14, PIK3R5, FCER1G, PIK3R1, RAC2, PIK3CB, LCP2, INPP5B, INPP5D, PIK3CD, GRB2, MAPK8, LAT, PRKCH, MAPK3, KRAS, ATM, AKT2, VAV1, PLCG2, NRAS, PRKCD, PDPK1, PRKCA, MAPK1, PLCG1, RRAS, RAC1, LYN |
| iNOS Signaling | 5.44 | 0.386 | | | −0.775 | IRF1, MAPK14, CALM1 (includes others), CD14, JAK3, JAK1, NFKBIA, IRAK3, RELA, IRAK4, TLR4, IFNGR1, LY96, STAT1, MAPK1, CREBBP, IKBKE |
| p53 Signaling | 5.40 | 0.286 | | | −0.218 | KAT2B, GSK3B, MAPK14, PML, PTEN, APAF1, CCNG1, PIK3R5, PIK3R1, TP53INP1, PIK3CB, TP53, CSNK1D, PIK3CD, MAPK8, HDAC1, MDM4, CCND2, ATM, EP300, RRM2B, AKT2, BCL2, PPP1R13B, MDM2, BCL2L1, ADCK3, HIPK2 |
| CREB Signaling in Neurons | 5.39 | 0.240 | | | 0.186 | SOS2, ATF2, GNA13, CALM1 (includes others), POLR2A, GNG11, GNG7, PIK3R5, GNB1, PIK3R1, PRKACB, PIK3CB, GNAI3, GNAQ, GNAI2, PIK3CD, GRB2, CREBBP, PRKCH, POLR2J2/POLR2J3, MAPK3, KRAS, GNAZ, GNB2L1, ADCY7, ATM, EP300, AKT2, PLCG2, PRKAR1A, POLR2H, NRAS, PRKCD, ATF4, PRKCA, ITPR3, MAPK1, RPS6KA1, PLCG1, GNG2, RRAS |
| Role of PKR in Interferon Induction and Antiviral Response | 5.38 | 0.400 | | | NaN | EIF2S1, IRF1, EIF2AK2, ATF2, MAPK14, APAF1, TNFRSF1A, FCGR1A, TAB2, TRAF3, NFKBIA, TP53, RELA, STAT1, CASP8, IKBKE |
| Role of JAK1 and JAK3 in Î³c Cytokine Signaling | 5.35 | 0.333 | | | NaN | KRAS, ATM, JAK3, PIK3R5, IL2RB, JAK1, PIK3R1, PIK3CB, IL2RG, NRAS, STAT3, PIK3CD, STAT1, STAT5A, IL7R, STAT6, MAPK1, GRB2, BLNK, RRAS, MAPK3 |
| Role of Pattern Recognition Receptors in Recognition of Bacteria and Viruses | 5.33 | 0.262 | | | 0.577 | EIF2S1, EIF2AK2, TLR5, TGFB1, TLR1, PIK3R5, PIK3R1, PIK3CB, C3AR1, TLR4, TLR8, PIK3CD, OAS3, MAPK8, TLR6, PRKCH, TLR2, MAPK3, OAS1, CCL5, IL1B, ATM, CASP1, OAS2, DDX58, RELA, PLCG2, PRKCD, PRKCA, IFIH1, CLEC6A, MAPK1, NLRC4 |
| LPS-stimulated MAPK Signaling | 5.33 | 0.315 | | | −0.626 | KRAS, ATF2, MAPK14, CD14, PAK1, ATM, PIK3R5, PIK3R1, NFKBIA, PIK3CB, RELA, TLR4, NRAS, PRKCD, PIK3CD, PRKCA, MAPK1, MAPK8, PRKCH, RRAS, RAC1, IKBKE, MAPK3 |
| Type I Diabetes Mellitus Signaling | 5.28 | | 0.273 | | NaN | IRF1, MAPK14, APAF1, TNFRSF1A, FCER1G, NFKBIA, HLA-F, IFNGR1, HLA-DMA, STAT1, HLA-DRB5, MAPK8, CD3E, HLA-E, IL1B, HLA-DQB1, CD3G, PRF1, JAK1, HLA-G, HLA-DOB, RELA, HLA-A, BCL2, MAPK1, HLA-C, HLA-B, CD247, CASP8, IKBKE |
| Gap Junction Signaling | 5.28 | | 0.245 | | NaN | CSNK1A1, SOS2, TUBB1, PIK3R5, PIK3R1, PRKACB, PIK3CB, GNAI3, CSNK1D, GNAQ, GNAI2, TUBA1A, PIK3CD, ACTB, GRB2, PRKCH, MAPK3, KRAS, TUBB, ADCY7, ATM, PPP3CA, SP1, AKT2, TUBB4B, PLCG2, PRKAR1A, NRAS, PRKCD, PRKCA, ITPR3, MAP3K2, TUBA1B, MAPK1, SP3, TUBA4A, PLCG1, RRAS |
| OX40 Signaling Pathway | 5.25 | | 0.292 | | −0.905 | HLA-DRB3, FCER1G, NFKBIA, HLA-F, HLA-DMA, HLA-DRB5, HLA-DPA1, CD4, MAPK8, HLA-DPB1, CD3E, HLA-E, HLA-DQB1, CD3G, HLA-G, HLA-DRB4, HLA-DOB, B2M, TRAF3, RELA, HLA-A, BCL2, HLA-C, HLA-B, BCL2L1, CD247 |
| CCR5 Signaling in Macrophages | 5.22 | | 0.319 | | NaN | CD3E, MAPK14, CCL5, CALM1 (includes others), GNB2L1, CD3G, GNG11, GNG7, GNB1, FCER1G, GNAI3, PLCG2, GNAI2, PRKCD, PRKCA, MAPK1, CD4, PLCG1, GNG2, MAPK8, PRKCH, CD247 |
| FcÎ³RIIB Signaling in B Lymphocytes | 5.22 | | 0.390 | | −1.5 | KRAS, ATM, PIK3R5, PIK3R1, PIK3CB, PLCG2, NRAS, PDPK1, INPP5D, PIK3CD, GRB2, MAPK8, BLNK, CD79A, RRAS, LYN |
| Toll-like Receptor Signaling | 5.22 | | 0.311 | | −2.324 | EIF2AK2, SIGIRR, MAPK14, TLR5, IL1B, TLR1, CD14, TAB2, NFKBIA, IRAK3, RELA, IRAK4, TLR4, TLR8, LY96, MAP3K1, UBB, MAPK1, MAPK8, TLR6, TLR10, TLR2, UBC |
| RhoA Signaling | 5.20 | | 0.262 | | −1.414 | LPAR6, GNA13, ACTR2, SEPT14, LIMK1, ARPC1B, ARPC5, CFL1, ACTB, ARPC4, ROCK1, RAPGEF2, LIMK2, MYL4, ARPC3, PPP1R12A, MYL12B, EZR, MYL12A, MYL9, ACTR3, SEPT2, PI4KA, PIP4K2A, PPP1R12B, IGF1R, ARPC2, MYL6, SEPT7, ARHGEF1, PPP1CB, ARHGAP9 |
| Glioma Signaling | 5.18 | | 0.284 | | −0.392 | SOS2, PTEN, CALM1 (includes others), PIK3R5, PIK3R1, PIK3CB, TP53, PIK3CD, E2F2, GRB2, PRKCH, MAPK3, KRAS, ATM, CAMK1D, AKT2, IGF2R, PLCG2, IGF1R, NRAS, PRKCD, PRKCA, MAPK1, PLCG1, MDM2, TFDP1, RRAS |
| ErbB4 Signaling | 5.13 | 0.333 | | | −0.229 | KRAS, SOS2, ATM, PIK3R5, PIK3R1, PIK3CB, PLCG2, ADAM17, NRAS, PRKCD, PDPK1, PIK3CD, PRKCA, PSENEN, MAPK1, GRB2, PLCG1, PRKCH, RRAS, MAPK3 |
| VEGF Signaling | 4.97 | 0.283 | | | −0.426 | EIF2S1, SOS2, PIK3R5, PIK3R1, YWHAE, PIK3CB, PIK3CD, ACTB, GRB2, ROCK1, ACTN4, MAPK3, KRAS, EIF1AX, ATM, ACTN1, AKT2, PLCG2, NRAS, EIF2S3, PRKCA, BCL2, MAPK1, PLCG1, BCL2L1, RRAS |
| Î±-Adrenergic Signaling | 4.94 | 0.287 | | | 0.894 | CALM1 (includes others), GNG11, GNG7, GNB1, PRKACB, GNAI3, GNAQ, GNAI2, PYGB, PRKCH, MAPK3, KRAS, PYGL, GNB2L1, ADCY7, PLCG2, PRKAR1A, NRAS, PRKCD, PRKCA, ITPR3, MAPK1, PLCG1, GNG2, RRAS |
| phagosome formation | 4.90 | 0.266 | | | NaN | RHOH, FCGR1B, TLR5, FCGR1A, TLR1, PIK3R5, RHOQ, FCER1G, PIK3R1, PIK3CB, FCGR2C, TLR4, TLR8, INPP5D, PIK3CD, TLR6, PRKCH, TLR2, ATM, PLCG2, FCAR, FCER2, PRKCD, PRKCA, ITGA4, PLCG1, TLR10, ITGA5, FCGR3A/FCGR3B |
| ErbB2-ErbB3 Signaling | 4.90 | 0.333 | | | 0.688 | GSK3B, KRAS, SOS2, PTEN, ATM, JAK3, PIK3R5, PIK3R1, SP1, PIK3CB, NRAS, STAT3, PDPK1, PIK3CD, STAT5A, MAPK1, GRB2, RRAS, MAPK3 |
| Docosahexaenoic Acid (DHA) Signaling | 4.84 | 0.385 | | | NaN | GSK3B, APAF1, IL1B, ATM, PIK3R5, APP, PIK3R1, PIK3CB, AKT2, PDPK1, PIK3CD, PNPLA2, BCL2, BCL2L1, ALOX15 |
| Myc Mediated Apoptosis Signaling | 4.78 | 0.328 | | | NaN | KRAS, SOS2, APAF1, ATM, PIK3R5, PIK3R1, YWHAE, PIK3CB, TP53, AKT2, YWHAH, IGF1R, NRAS, PIK3CD, BCL2, GRB2, MAPK8, RRAS, CASP8 |
| Activation of IRF by Cytosolic Pattern Recognition Receptors | 4.77 | 0.317 | | | 1.342 | DHX58, IRF9, ATF2, STAT2, DDX58, IFIT2, PPIB, TRAF3, NFKBIA, IFNAR1, RELA, SIKE1, STAT1, IFIH1, ADAR, CREBBP, ZBP1, MAPK8, PIN1, IKBKE |
| Estrogen-Dependent Breast Cancer Signaling | 4.77 | | 0.317 | | 0.688 | KRAS, ATF2, ATM, EP300, PIK3R5, PIK3R1, SP1, PIK3CB, AKT2, RELA, IGF1R, NRAS, ATF4, PIK3CD, STAT5A, HSD17B4, MAPK1, CREBBP, RRAS, MAPK3 |
| Axonal Guidance Signaling | 4.77 | | 0.184 | | NaN | SOS2, GNA13, NFATC2, GNG11, LIMK1, GNG7, TUBB1, GNB1, PIK3R5, PRKACB, PIK3CB, ARPC1B, GNAI3, ARPC5, CXCR4, GNAQ, TUBA1A, ARHGEF6, PIK3CD, SDCBP, PRKCH, ADAM9, GNAZ, MYL4, ARPC3, CRKL, ADAM19, MYL9, TUBB4B, PRKAR1A, ARPC2, PRKCD, NRAS, NCK2, TUBA1B, MMP8, ITGA4, TUBA4A, ITGA5, GSK3B, SEMA4D, ACTR2, MMP9, WIPF1, PIK3R1, RAC2, ADAM17, LNPEP, GNAI2, CFL1, PLXNB2, ARPC4, GRB2, GIT1, ROCK1, CRK, MAPK3, KEL, KRAS, LIMK2, GNB2L1, TUBB, ADAM8, PAK1, ATM, MYL12B, MYL12A, PPP3CA, AKT2, ACTR3, PLCG2, NFATC3, MYL6, PRKCA, MAPK1, PLCG1, GNG2, SRGAP2, RRAS, RAC1 |
| Insulin Receptor Signaling | 4.72 | | 0.246 | | −0.522 | GSK3B, PPP1CC, SOS2, PTEN, PPP1R7, PIK3R5, FOXO4, RHOQ, PIK3R1, PRKACB, CBL, PIK3CB, INPP5B, INPP5D, PIK3CD, GRB2, CRK, MAPK8, RPS6KB1, MAPK3, KRAS, PPP1R12A, CRKL, ATM, JAK1, AKT2, PRKAR1A, NRAS, PDPK1, MAPK1, PPP1CB, ACLY, RRAS |
| Pancreatic Adenocarcinoma Signaling | 4.69 | | 0.264 | | 0.626 | TGFB1, MMP9, JAK3, PIK3R5, PIK3R1, SMAD4, PIK3CB, TP53, STAT3, PIK3CD, STAT1, SMAD3, E2F2, GRB2, MAPK8, MAPK3, KRAS, NOTCH1, ATM, JAK1, AKT2, RELA, BCL2, MAPK1, MDM2, BCL2L1, TFDP1, RAC1 |
| Lymphotoxin Î² Receptor Signaling | 4.68 | | 0.333 | | 0 | APAF1, ATM, EP300, PIK3R5, PIK3R1, TRAF3, NFKBIA, PIK3CB, AKT2, RELA, PDPK1, PIK3CD, CXCL1, MAPK1, CREBBP, BCL2L1, IKBKE, MAPK3 |
| IL-17A Signaling in Airway Cells | 4.66 | | 0.312 | | 1.213 | GSK3B, MAPK14, PTEN, ATM, JAK3, PIK3R5, JAK1, PIK3R1, NFKBIA, PIK3CB, AKT2, RELA, STAT3, IL17RA, PIK3CD, CXCL1, MAPK1, MAPK8, IKBKE, MAPK3 |
| ErbB Signaling | 4.53 | | 0.279 | | NaN | GSK3B, KRAS, SOS2, MAPK14, PAK1, ATM, PIK3R5, PIK3R1, PIK3CB, PLCG2, NRAS, PRKCD, PDPK1, PIK3CD, PRKCA, NCK2, MAPK1, GRB2, PLCG1, MAPK8, PRKCH, RRAS, RPS6KB1, MAPK3 |
| protein Kinase A Signaling | 4.53 | | 0.187 | | 1.778 | AKAP13, PTEN, GNA13, TGFB1, NFATC2, MPPE1, GNG11, GNG7, GNB1, SMAD4, PRKACB, YWHAE, NFKBIA, GNAI3, PTPN18, ANAPC5, GNAQ, SMAD3, PRKCH, MYL4, FLNA, LEF1, H3F3A/H3F3B, MYL9, PRKAR1A, PRKCD, BRAF, MAP3K1, PPP1CB, H1F0, GSK3B, FLNB, PPP1CC, TDP2, ATF2, CALM1(includesthers), DUSP11, PPP1R7, HIST1H1E, PTPN22, HIST1H1B, YWHAH, GNAI2, PYGB, DUSP1, PDE6D, CREBBP, ROCK1, TCF7L2, SIRPA, MAPK3, PYGL, GNB2L1, PPP1R12A, ADCY7, MYL12B, EP300, MYL12A, PPP3CA, PLCG2, RELA, NFATC3, MYL6, ATF4, ITPR3, KDELR2, PRKCA, MAPK1, PLCG1, GNG2, APEX1, CDC25B |
| Estrogen Receptor Signaling | 4.39 | | 0.244 | | NaN | KAT2B, SOS2, POLR2A, HNRNPD, CCNC, MED14, GRB2, CREBBP, POLR2J2/POLR2J3, CARM1, TRRAP, MAPK3, KRAS, MED13L, NCOA1, TAF2, EP300, H3F3A/H3F3B, MED13, TAF7, GTF2A1, SPEN, POLR2H, NRAS, GTF2F2, MAPK1, PHB2, MED12, TAF10, NCOA2, RRAS |
| Melanoma Signaling | 4.39 | | 0.357 | | NaN | KRAS, PTEN, ATM, PIK3R5, PIK3R1, PIK3CB, TP53, AKT2, NRAS, BRAF, PIK3CD, MAPK1, MDM2, RRAS, MAPK3 |
| IL-6 Signaling | 4.35 | | 0.250 | | −1.134 | SOS2, MAPK14, MCL1, TNFRSF1A, IL6R, CSNK2A2, PIK3R5, IL1R2, PIK3R1, NFKBIA, PIK3CB, STAT3, PIK3CD, GRB2, MAPK8, MAPK3, KRAS, IL1B, CSNK2B, CD14, ATM, AKT2, RELA, NRAS, TNFAIP6, CEBPB, MAPK1, RRAS, IKBKE |
| IL-17 Signaling | 4.35 | | 0.292 | | NaN | GSK3B, KRAS, ATF2, MAPK14, ATM, PIK3R5, JAK1, PIK3R1, PIK3CB, AKT2, RELA, NRAS, IL17RA, PIK3CD, CEBPB, CXCL1, MAPK1, MAPK8, TIMP1, RRAS, MAPK3 |
| Neurotrophin/TRK Signaling | 4.33 | | 0.299 | | 0 | KRAS, SOS2, ATF2, ATM, EP300, PIK3R5, PIK3R1, PIK3CB, NRAS, PDPK1, ATF4, PIK3CD, MAPK1, GRB2, RPS6KA1, CREBBP, PLCG1, MAPK8, RRAS, MAPK3 |
| CNTF Signaling | 4.33 | | 0.327 | | −0.243 | KRAS, RPS6KA3, ATM, PIK3R5, JAK1, PIK3R1, PIK3CB, NRAS, STAT3, PIK3CD, STAT1, MAPK1, GRB2, RPS6KA1, RRAS, RPS6KB1, MAPK3 |
| IL-12 Signaling and Production in Macrophages | 4.31 | | 0.239 | | NaN | IRF1, MAPK14, TGFB1, PIK3R5, PIK3R1, PIK3CB, TLR4, IFNGR1, RAB7A, PIK3CD, STAT1, RXRA, MAPK8, PRKCH, TLR2, MAPK3, ALOX15, REL, NCOA1, ATM, EP300, SPI1, AKT2, RELA, LYZ, PRKCD, PRKCA, CEBPB, STAT6, MAPK1, SERPINA1, IKBKE |
| Communication between Innate and Adaptive Immune Cells | 4.27 | | 0.270 | | NaN | TLR5, HLA-E, TNFSF13B, CCL5, IL1B, TLR1, HLA-DRB3, HLA-G, HLA-DRB4, B2M, FCER1G, IGHA1, HLA-F, TLR4, TLR8, HLA-A, CD8A, HLA-DRB5, CD4, HLA-C, HLA-B, TLR6, TLR10, TLR2 |
| IL-9 Signaling | 4.24 | | 0.382 | | 1.387 | ATM, JAK3, PIK3R5, JAK1, BCL3, PIK3R1, PIK3CB, IL2RG, RELA, STAT3, PIK3CD, STAT1, STAT5A |
| Oncostatin M Signaling | 4.24 | | 0.382 | | 0.577 | KRAS, MT2A, JAK3, JAK1, NRAS, STAT3, CHI3L1, STAT1, STAT5A, MAPK1, GRB2, RRAS, MAPK3 |
| FGF Signaling | 4.14 | | 0.271 | | 0.209 | SOS2, ATF2, MAPK14, CRKL, ATM, EP300, PIK3R5, PIK3R1, PIK3CB, AKT2, STAT3, ATF4, PIK3CD, PRKCA, MAP3K1, MAPK1, GRB2, CREBBP, PLCG1, MAPK8, CRK, RAC1, MAPK3 |
| Growth Hormone Signaling | 4.13 | | 0.290 | | 1.606 | RPS6KA3, ATM, PIK3R5, PIK3R1, PIK3CB, PLCG2, IGF1R, STAT3, PRKCD, PDPK1, PIK3CD, PRKCA, STAT1, STAT5A, MAPK1, RPS6KA1, PLCG1, PRKCH, RPS6KB1, MAPK3 |
| Role of PI3K/AKT Signaling in the Pathogenesis of Influenza | 4.11 | | 0.297 | | 0.258 | GSK3B, CCL5, CRKL, ATM, PIK3R5, MLH1, PIK3R1, NFKBIA, PIK3CB, AKT2, PLAC8, IFNAR1, GNAI3, RELA, GNAI2, PIK3CD, MAPK1, CRK, MAPK3 |
| IGF-1 Signaling | 4.07 | | 0.258 | | −1.043 | SOS2, CSNK2A2, PIK3R5, PIK3R1, PRKACB, YWHAE, PIK3CB, YWHAH, STAT3, PIK3CD, GRB2, MAPK8, RPS6KB1, MAPK3, KRAS, CSNK2B, ATM, JAK1, AKT2, PRKAR1A, IGF1R, NRAS, PDPK1, MAPK1, RRAS |
| Death Receptor Signaling | 4.02 | | 0.261 | | 0.816 | PARP12, PARP4, APAF1, TNFRSF1A, LIMK1, PARP9, PARP8, NFKBIA, ACIN1, RELA, ARHGDIB, TIPARP, NAIP, CFLAR, ACTB, ZC3HAV1, BCL2, TNFSF10, ROCK1, MAPK8, PARP14, CASP8, IKBKE, SPTAN1 |
| RAR Activation | 3.90 | | 0.211 | | NaN | KAT2B, MAPK14, PML, PTEN, DHRS9, TGFB1, CSNK2A2, PIK3R1, SMAD4, PRKACB, PIK3CB, PNRC1, PIK3CD, DUSP1, ACTB, SMAD3, STAT5A, RXRA, CREBBP, MAPK8, PRKCH, PHF10, CARM1, REL, NCOA1, CSNK2B, ADCY7, EP300, SMARCC2, AKT2, CITED2, RELA, PRKAR1A, PRKCD, PDPK1, PRKCA, MAP3K1, MAPK1, ARID1A, RAC1 |
| Cytotoxic T Lymphocyte-mediated Apoptosis of Target Cells | 3.86 | 0.375 | | | −1.414 | CD3E, HLA-A, APAF1, CD3G, BCL2, PRF1, HLA-C, B2M, HLA-B, FCER1G, CD247, CASP8 |
| PPARÎ±/RXRÎ± Activation | 3.81 | 0.212 | | | −1.291 | SOS2, MAPK14, TGFB1, ADIPOR1, BCL3, IL1R2, SMAD4, PRKACB, NFKBIA, NR2C2, GNAQ, ACOX1, SMAD3, NCOA6, GRB2, RXRA, CREBBP, MAPK8, HSP90AA1, MAPK3, KRAS, IL1B, ADCY7, TGFBR3, HSP90B1, EP300, RELA, PLCG2, PRKAR1A, CD36, NRAS, PRKCA, MAPK1, PLCG1, MED12, HSP90AB1, RRAS, IKBKE |
| Crosstalk between Dendritic Cells and Natural Killer Cells | 3.81 | 0.258 | | | NaN | KIR3DL2, ITGAL, HLA-E, KIR2DL2, TLN1, HLA-DRB3, KIR2DL1/KIR2DL3, PRF1, IL2RB, HLA-G, HLA-DRB4, HLA-F, IL2RG, RELA, TLR4, HLA-A, ACTB, ICAM3, HLA-DRB5, TNFSF10, HLA-C, HLA-B, KLRC4-KLRK1/KLRK1 |
| Mouse Embryonic Stem Cell Pluripotency | 3.79 | 0.253 | | | −0.408 | GSK3B, KRAS, SOS2, MAPK14, LEF1, ATM, JAK3, PIK3R5, JAK1, PIK3R1, SMAD4, PIK3CB, TP53, AKT2, APC, NRAS, STAT3, PIK3CD, MAPK1, GRB2, CREBBP, TCF7L2, RRAS, MAPK3 |
| Nur77 Signaling in T Lymphocytes | 3.77 | 0.298 | | | NaN | CD3E, APAF1, CALM1 (includes others), HLA-DQB1, CD3G, EP300, HLA-DOB, FCER1G, PPP3CA, CABIN1, HLA-DMA, MAP3K2, BCL2, HLA-DRB5, RXRA, CD247, HDAC1 |
| IL-22 Signaling | 3.74 | 0.417 | | | 0.000 | MAPK14, STAT3, IL10RB, STAT1, STAT5A, MAPK1, JAK1, MAPK8, AKT2, MAPK3 |
| HIF1Î± Signaling | 3.69 | 0.245 | | | NaN | MAPK14, MMP9, PIK3R5, PIK3R1, PIK3CB, TP53, PIK3CD, CREBBP, MAPK8, HSP90AA1, MAPK3, KRAS, NCOA1, ATM, EP300, AKT2, NRAS, LDHA, MAPK1, MMP8, MDM2, APEX1, RRAS, RBX1, LDHB |
| IL-1 Signaling | 3.66 | 0.253 | | | −1.414 | GNAZ, MAPK14, GNA13, GNB2L1, GNG11, ADCY7, GNG7, GNB1, TAB2, PRKACB, NFKBIA, IRAK3, GNAI3, RELA, IRAK4, PRKAR1A, GNAQ, GNAI2, MAP3K1, MAPK1, GNG2, MAPK8, IKBKE |
| UVB-Induced MAPK Signaling | 3.65 | | 0.302 | | −0.258 | MAPK14, RPS6KA3, ATM, PIK3R5, H3F3A/H3F3B, PIK3R1, PIK3CB, TP53, PRKCD, PIK3CD, PRKCA, MAPK1, MAPK8, PRKCH, RPS6KB1, MAPK3 |
| Primary Immunodeficiency Signaling | 3.64 | | 0.312 | | NaN | CD3E, JAK3, IGLL1/IGLL5, IGHD, IGHA1, TAP1, IL2RG, CD8A, TAP2, IL7R, IGHM, CD4, BLNK, CD79A, ZAP70 |
| Role of IL-17A in Arthritis | 3.54 | | 0.296 | | NaN | ATF2, MAPK14, CCL5, ATM, PIK3R5, PIK3R1, NFKBIA, PIK3CB, RELA, IL17RA, PIK3CD, CXCL1, MAPK1, RPS6KA1, MAPK8, MAPK3 |
| Sertoli Cell-Sertoli Cell Junction Signaling | 3.54 | | 0.208 | | NaN | GSK3B, ATF2, F11R, MAPK14, PTEN, TNFRSF1A, YBX3, TUBB1, PRKACB, SPTBN1, TUBA1A, EPB41, ACTB, MAPK8, ACTN4, MAPK3, CTNNA1, KRAS, TUBB, TGFBR3, ACTN1, AKT2, RAB8B, TUBB4B, PRKAR1A, NRAS, MAP3K2, MAP3K1, TUBA1B, MAPK1, CLINT1, ITGA4, TUBA4A, RRAS, RAC1, ITGA5, SPTAN1 |
| Endoplasmic Reticulum Stress Pathway | 3.53 | | 0.429 | | NaN | EIF2S1, DNAJC3, ATF4, HSP90B1, HSPA5, XBP1, MAPK8, TAOK3, ATF6 |
| GÎ ± q Signaling | 3.51 | | 0.218 | | 0.365 | GSK3B, RHOH, CALM1 (includes others), NFATC2, GNG11, GNG7, PIK3R5, GNB1, RHOQ, PIK3R1, NFKBIA, PIK3CB, GNAQ, PIK3CD, RGS2, ROCK1, PRKCH, MAPK3, GNB2L1, ATM, PPP3CA, AKT2, RELA, PLCG2, NFATC3, PRKCD, PRKCA, ITPR3, MAPK1, PLCG1, GNG2, IKBKE |
| GÎ ± 12/13 Signaling | 3.48 | | 0.231 | | −1.569 | LPAR6, F2RL1, GNA13, PIK3R5, PIK3R1, NFKBIA, PIK3CB, PIK3CD, ROCK1, MAPK8, MAPK3, KRAS, MYL4, MYL12B, ATM, MYL12A, MYL9, AKT2, VAV1, RELA, MYL6, NRAS, ARHGEF1, MAP3K1, MAPK1, RRAS, IKBKE |
| Lipid Antigen Presentation by CD1 | 3.40 | | 0.385 | | NaN | AP1G2, CD3E, AP2B1, AP2A1, CANX, PSAP, B2M, FCER1G, AP2M1, ARF6 |
| Superpathway of Inositol Phosphate Compounds | 3.34 | | 0.200 | | NaN | PPP1CC, MTMR4, NUDT3, PTEN, ATP1A1, PPM1F, TMEM55A, DUSP11, PPP4R1, PPP1R7, PIK3R5, PTPN22, PIK3R1, FIG4, PIK3CB, INPP5B, INPP5D, PIK3CD, PPTC7, PPP2R5A, DUSP1, NUDT5, SIRPA, INPP5A, PPP1R12A, ATM, IPMK, PPP3CA, PI4KA, PIP4K2A, PLCG2, ALPL, ITPK1, PI4K2A, IP6K1, PPP2R5D, PPP1R13B, PLCG1, CDC25B |
| Amyloid Processing | 3.32 | | 0.294 | | −1.342 | GSK3B, CSNK1A1, MAPK14, CSNK2B, CAPN1, CSNK2A2, APP, PRKACB, AKT2, PRKAR1A, CSNK1D, PSENEN, MAPK1, CAPNS1, MAPK3 |
| G- protein Coupled Receptor Signaling | 3.29 | | 0.188 | | NaN | SOS2, TDP2, ATF2, MPPE1, GABBR1, PIK3R5, PIK3R1, FPR1, PRKACB, NFKBIA, PIK3CB, RASGRP1, GNAI3, GNAQ, STAT3, GNAI2, PIK3CD, DUSP1, P2RY13, PDE6D, RGS2, GRB2, CREBBP, HCAR2, PTGDR, MAPK3, KRAS, ADCY7, HCAR3, ATM, EP300, FPR2, AKT2, RELA, PRKAR1A, NRAS, PDPK1, BRAF, ATF4, PRKCA, CXCR2, MAPK1, RPS6KA1, RGS10, APEX1, RRAS, HRH2, IKBKE |
| Reelin Signaling in Neurons | 3.27 | | 0.253 | | NaN | GSK3B, ITGAL, HCK, CRKL, ATM, ITGA6, PIK3R5, APP, FGR, PIK3R1, PIK3CB, ARHGEF6, PIK3CD, ARHGEF1, ARHGEF2, ITGB3, ITGA4, MAPK8, ITGA5, LYN |
| Glioma Invasiveness Signaling | 3.25 | | 0.281 | | −0.500 | KRAS, RHOH, MMP9, ATM, PLAUR, PIK3R5, RHOQ, PIK3R1, PIK3CB, NRAS, PIK3CD, MAPK1, ITGB3, TIMP1, RRAS, MAPK3 |
| GDNF Family Ligand-Receptor Interactions | 3.25 | | 0.265 | | −1.000 | KRAS, SOS2, ATM, PIK3R5, PIK3R1, PIK3CB, PLCG2, NRAS, PIK3CD, ITPR3, PDLIM7, MAPK1, GRB2, PLCG1, MAPK8, RRAS, RAC1, MAPK3 |
| Ceramide Signaling | 3.19 | | 0.250 | | 0.000 | KSR1, KRAS, TNFRSF1A, ATM, PIK3R5, PIK3R1, PIK3CB, AKT2, RELA, S1PR5, NRAS, PIK3CD, PPP2R5A, MAP3K1, BCL2, PPP2R5D, MAPK8, RRAS, PPP2CA, MAPK3 |
| Relaxin Signaling | 3.14 | | 0.215 | | −0.229 | TDP2, GNA13, MMP9, MPPE1, GNG11, GNG7, PIK3R5, GNB1, PIK3R1, PRKACB, NFKBIA, PIK3CB, GNAI3, GNAQ, GNAI2, PIK3CD, PDE6D, MAPK3, GNAZ, GNB2L1, ADCY7, ATM, AKT2, RELA, PRKAR1A, BRAF, MAPK1, GNG2, APEX1 |
| GNRH Signaling | 3.13 | | 0.217 | | −1.134 | SOS2, ATF2, MAPK14, PRKACB, GNAI3, GNAQ, GNAI2, GRB2, CREBBP, MAPK8, PRKCH, MAPK3, KRAS, ADCY7, PAK1, EP300, RELA, PRKAR1A, NRAS, PRKCD, ATF4, PRKCA, ITPR3, MAP3K2, MAP3K1, MAPK1, RRAS, RAC1 |
| eNOS Signaling | 3.11 | | 0.211 | | 1.225 | HSPA1L, LPAR6, HSPA1A/HSPA1B, CALM1 (includes others), PIK3R5, PIK3R1, PRKACB, HSPA6, PIK3CB, AQP3, GNAQ, PIK3CD, HSPA8, PRKCH, HSP90AA1, SLC7A1, ADCY7, HSP90B1, ATM, AKT2, PLCG2, PRKAR1A, PRKCD, PDPK1, PRKCA, ITPR3, HSPA5, PLCG1, HSP90AB1, CASP8 |
| Graft-versus-Host Disease Signaling | 3.10 | | 0.292 | | NaN | HLA-E, IL1B, HLA-DQB1, KIR2DL1/KIR2DL3, PRF1, HLA-G, HLA-DOB, FCER1G, HLA-F, HLA-A, HLA-DMA, HLA-DRB5, HLA-C, HLA-B |
| ATM Signaling | 3.07 | | 0.271 | | 0.832 | ATF2, MAPK14, TLK2, CBX5, SMC1A, ATM, EP300, TLK1, NFKBIA, TP53, TRIM28, ATF4, CREBBP, MAPK8, MDM2, MDM4 |
| HER-2 Signaling in Breast Cancer | 3.06 | | 0.250 | | NaN | GSK3B, KRAS, SOS2, ATM, PIK3R5, PIK3R1, PIK3CB, TP53, AKT2, NRAS, PRKCD, PIK3CD, PRKCA, GRB2, PLCG1, ITGB3, MDM2, PRKCH, RRAS |
| PPAR Signaling | 3.03 | | 0.234 | | 1.279 | KRAS, SOS2, NCOA1, IL1B, TNFRSF1A, HSP90B1, EP300, IL1R2, NFKBIA, CITED2, RELA, NRAS, STAT5A, MAPK1, GRB2, RXRA, CREBBP, HSP90AB1, HSP90AA1, RRAS, IKBKE, MAPK3 |
| Small Cell Lung Cancer Signaling | 3.01 | | 0.254 | | NaN | FHIT, PTEN, APAF1, ATM, PIK3R5, PIK3R1, TRAF3, NFKBIA, PIK3CB, TP53, AKT2, RELA, PIK3CD, BCL2, RXRA, BCL2L1, TFDP1, IKBKE |
